# Endemic diversity and distribution of the Iranian vascular flora across phytogeographical regions, biodiversity hotspots and areas of endemism

**DOI:** 10.1038/s41598-019-49417-1

**Published:** 2019-09-10

**Authors:** Jalil Noroozi, Amir Talebi, Moslem Doostmohammadi, Sara Manafzadeh, Zahra Asgarpour, Gerald M. Schneeweiss

**Affiliations:** 1 Department of Botany and Biodiversity Research, University of Vienna, Vienna, Austria; 2 Department of Plant Science, University of Tehran, Tehran, Iran

**Keywords:** Biodiversity, Conservation biology, Plant ecology, Biogeography

## Abstract

Endemism is one of the most important concepts in biogeography and is of high relevance for conservation biology. Nevertheless, our understanding of patterns of endemism is still limited in many regions of high biodiversity. This is also the case for Iran, which is rich in biodiversity and endemism, but there is no up-to-date account of diversity and distribution of its endemic species. In this study, a comprehensive list of all endemic vascular plant species of Iran, their taxonomic composition and their geographical distribution are presented. To this end, a total of 2,597 (sub)endemic vascular plant species of Iran were documented and their distribution in three phytogeographical regions, two biodiversity hotspots and five areas of endemism were analysed. The Irano-Turanian phytogeographical region harbours 88% of the Iranian endemics, the majority of which are restricted to the Irano-Anatolian biodiversity hotspot (84%). Nearly three quarters of the endemic species are restricted to mountain ranges. The rate of endemism increases along an elevational gradient, causing the alpine zone to harbour a disproportionally high number of endemics. With increasing pastoralism, urbanization, road construction and ongoing climate change, the risk of biodiversity loss in the Iranian mountains is very high, and these habitats need to be more effectively protected.

## Introduction

The concept of endemism, which describes that a taxon is restricted in its distribution to a distinct area, is central in biogeography^1^. It is also considered a significant criterion for biodiversity conservation at the global, national and local scales^2–4^. Biodiversity is unevenly distributed both around the Earth and among the different lineages of the tree of life^5,6^. Areas with high concentration of narrowly distributed species are of high priority to preserve biodiversity^3,7^, and identification of areas with high priority for conservation is a fundamental task of conservation biogeography^8,9^. The number of endemic species in a biogeographic region is a first step for assessing the conservation situation of that region^10^. Documenting endemic richness in a biodiversity hotspot or area of endemism is important not only for setting their conservation priorities^11^, but also for understanding the evolutionary and ecological processes that have shaped the biodiversity hotspots in general and areas of endemism in particular^12,13^.

**Figure 1:**
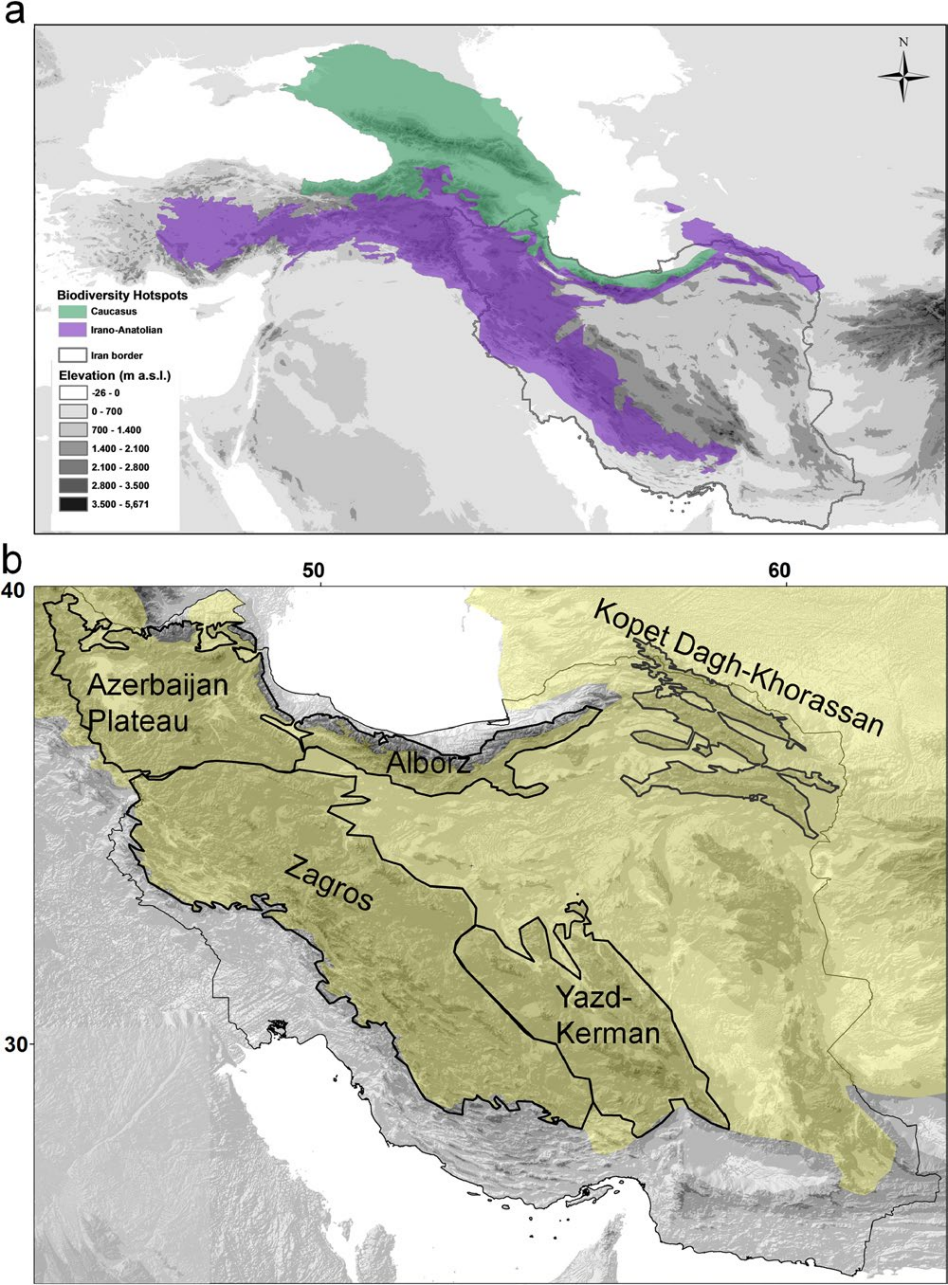
Global biodiversity hotspots, phytogeographic regions and areas of endemism in Iran. (**a**) Topographic map of Iran and adjacent regions showing the Irano-Anatolian and the Caucasus biodiversity hotspots. (**b**) Topographic map of Iran indicating phytogeographical regions (Irano-Turanian region indicated as yellow shaded area, the Saharo-Sindian region and the Euro-Siberian region indicated as unshaded areas south and north, respectively, of the Irano-Turanian region) and areas of endemism, well associated with high mountain ranges of Iran (indicated by outlines and their names).

This study focuses on Iran, a vast country in Southwest Asia (Fig. 1a) with very diverse landscapes^14–16^ comprising a large number of arid to semi-arid mountain ranges. It is home to a high plant and animal diversity with more than 8,000 vascular plant taxa, approximately 30% of which are endemics^17^, and more than 1,000 species of mainland vertebrates^14^. Moreover, Iran is at the crossroads of three major phytogeographic regions (the Irano-Turanian, the Saharo-Sindian and the Euro-Siberian regions sensu White and Léonard^18^), covers parts of two global biodiversity hotspots, i.e. Irano-Anatolian and Caucasus^7^, and harbours five areas of endemism^19^. These areas of endemism are clearly associated with the major mountain ranges of the Iranian Plateau, harbouring the majority of the Iranian endemic flora^19^. The Iranian Plateau is one of the hotspots of evolutionary and biological diversity of the Old World and serves as a bridge for migration of many plants, connecting the eastern and western floras of Eurasia^20^. Despite its outstanding species richness, geographic extent, and evolutionary importance, and although it has been the object of several studies on endemism, diversity and chorology of plants^17,21–32^, there is no updated and comprehensive work available summarizing the diversity and distribution of endemic vascular flora in Iran as a whole, in its phytogeographical regions, in biodiversity hotspots and also in areas of endemism.

In this study, we provide a complete and updated list of all endemic vascular plant species of Iran with their geographical distributions. Documenting all vascular plant species endemic to Iran, we can (1) uncover the taxonomic composition of the Iranian endemic flora, (2) estimate the number of Iranian endemics per phytogeographic region, biodiversity hotspot and area of endemism, (3) assess the floristic connections between areas of endemism, (4) quantify the contribution of large taxonomic groups to each area of endemism, (5) record the elevational distribution of endemic species, and finally (6) construct life form spectra in the biogeographical regions of Iran.

## Results and Discussion

### Taxonomic distribution of endemic diversity

**Figure 2:**
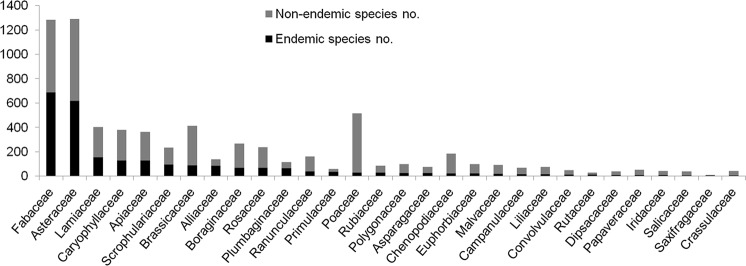
Number of endemic and non-endemic species in the 30 most endemic-rich families of the Iranian vascular flora (sorted by number of endemic species).

**Figure 3:**
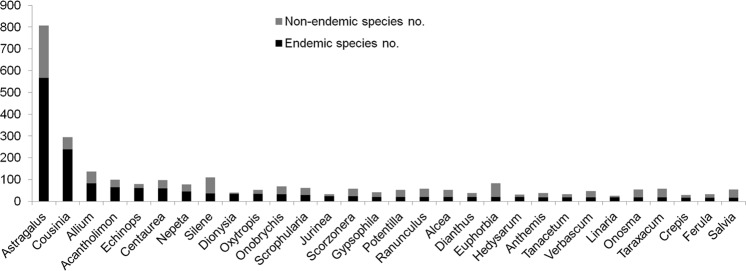
Number of endemic and non-endemic species of the 30 most endemic-rich genera of the Iranian vascular flora (sorted by number of endemic species).

The Iranian vascular flora includes a total of 2,597 endemic or subendemic species (32% of all native species), belonging to 359 genera within 65 families. There are no endemic families, but 26 endemic and subendemic genera (Table 1). Of those (sub)endemic genera, 19 are monotypic, two are ditypic and the remaining five multitypic. The highest number of (sub-)endemic genera is found in Apiaceae and Brassicaceae with 13 and 7 genera, respectively. However, data on endemic genera need to be viewed with caution because of uncertainty in taxonomic circumscription, for example generic re-alignments in Brassicaceae^33–36^ or addition of species to *Parrotia*^37^. Dicots contain 2,421 endemic species in Iran (93% of all vascular plant endemics) out of 6,889 species, monocots contain 175 endemics (7% of all vascular plant endemics) out of 1,164 species, and gymnosperms have only one endemic out of 10 species. None of the 53 pteridophyte species occurring in Iran is endemic. The percentage of endemism in dicots (35%) is much higher than in monocots (15%) and gymnosperms (10%). This likely is due to the high number of endemic species in a few eudicot genera, such as *Astragalus* (Fabaceae) and *Cousinia* (Astercaeae). The 10 largest families in terms of number of endemic species comprise 82% of the total Iranian endemic vascular flora (Fig. 2). Fabaceae is the number one in terms of endemic species (687 endemic species), which is due to the hyperdiverse genus *Astragalus* with ca. 800 species (70% endemics; Fig. 3), thus covering ca. 21% of the Iranian endemic vascular flora. The second largest family in terms of endemics is Asteraceae (Fig. 2), comprising 618 endemic species (48% of Asteraceae species). *Cousinia* is by far the largest genus of this family in Iran with 294 species (81% endemics; Fig. 3), encompassing 9% of the Iranian endemic species. Further families with high numbers of endemics are Lamiaceae (155), Apiaceae (127), Caryophyllaceae (127), Scrophulariaceae (96), Brassicaceae (88), Alliaceae (84), Boraginaceae (70), Rosaceae (69), and Plumbaginaceae (65; Fig. 2). Whereas these are all large families with respect to number of species present in Iran, Poaceae, which is the third biggest family in terms of total number of species (comprising 519 species) has only 28 endemic species (5%; Fig. 2). Although these numbers may decrease due to taxonomic revision^27,38^, new endemic species continue to be described^17^, rendering it unlikely that the overall pattern will change in the future.

**Table 1:** Endemic genera of Iran. See text for details.

Genera	Family	Distribution	AE^a^	El. (m)^b^	Phyto. region^c^
*Alococarpum* Riedl & Kuber	Apiaceae	W/C Iran	lo^d^	1000–1500	IT^e^
*Azilia* Hedge & Lamond	Apiaceae	W Iran	za^f^	1800–2300	IT
*Brossardia* Boiss.	Brassicaceae	N Iraq, Iran	za	1700–2500	IT
*Clastopus* Boiss.	Brassicaceae	W/N Iran	al^g^, az^h^, za	2500–3700	IT
*Demavendia* Pimenov	Apiaceae	W/N/E/S Iran, S Turkmenistan	al, za, ke^j^, ko^k^	1000–2500	IT
*Dicyclophora* Boiss.	Apiaceae	W/S Iran	lo	50–1800	SS^l^
*Diplotaenia* Boiss.	Apiaceae	E Turkey, NW/N Iran,	al, az	2500–3500	IT
*Elburzia* Hedge	Brassicaceae	N Iran	al	2000–3000	IT
*Ergocarpon* C.C. Towns.	Apiaceae	E Iraq, Iran	lo	200–700	SS
*Haussknechtia* Boiss.	Apiaceae	W Iran	za	1700–2500	IT
*Hymenocephalus* Jaub. & Spach	Asteraceae	W Iran	za	?	IT
*Jurinella* Jaub. & Spach	Asteraceae	NE Iraq, E Turkey, S Transcaucasia, Caucasus, NW/N/NE Iran,	al, az, ko	2500–4000	IT
*Kalakia* Alava	Apiaceae	NW/N Iran	al, az	1000–2500	IT
*Mozaffariania* Pimenov & Maassoumi	Apiaceae	SE Iran	lo	300–600	SS
*Myopordon* Boiss.	Asteraceae	W/N/S Iran	al, za, ke, ko	3500–4000	IT
*Opoidia* Lindl.	Apiaceae	NE Iran	ko	1500	IT
*Opsicarpium* Mozaff.	Apiaceae	W/NW Iran	az, za	500–2300	IT
*Phuopsis* (Griseb.) Hook. f.	Rubiaceae	Talish, NW/N Iran	al, az	500–2700	ES^m^
*Physoptychis* Boiss.	Brassicaceae	E Turkey, Transcaucasia, Iran	al, az, za	2500–4500	IT
*Pseudocamelina* (Boiss.) N. Busch	Brassicaceae	W/NW/N/S Iran	al, az, za, ke	1500–2500	IT
*Pseudofortuynia* Hedge	Brassicaceae	W/S Iran	za, ke	1500–2500	IT
*Sclerochorton* Boiss.	Apiaceae	W Iran	za	3000–3500	IT
*Stenotaenia* Boiss.	Apiaceae	W/NW/N Iran	al, az, za	1800–3200	IT
*Zerdana* Boiss.	Brassicaceae	W/S Iran	za, ke	3000–4000	IT
*Zeugandra* P.H. Davis	Campanulaceae	W Iran	za	1500–2200	IT
*Zhumeria* Rech.f. & Wendelbo	Lamiaceae	S Iran	lo	200–1500	SS

^a^Area of endemism; ^b^Elevation ranges (meter above sea level); ^c^Phytogeographical regions; ^d^Lowland; ^e^Irano-Turanian; ^f^Zagros; ^g^Alborz; ^h^Azerbaijan Plateau; ^j^Yazd-Kerman; ^k^Kopet Dagh-Khorassan; ^l^Shaharo-Sindian; ^m^Euro-Siberian.

The number of endemic species and the degree of endemism in the Iranian vascular flora is similar to Turkey, but twice that of Greece and the Iberian Peninsula (Table 2). Considering the smaller size of Turkey (about half of Iran), Turkey is proportionally richer than Iran. Asteraceae, Fabaceae, Lamiaceae, Caryophyllaceae and Brassicaceae are among the ten richest families in the Mediterranean countries and in Iran, but differ in their order (Table 3). In all five Mediterranean regions, Asteraceae contains the highest number of endemic species (Table 3), as is typical for non-tropical regions^39^, but in Iran the high diversity of *Astragalus* renders Fabaceae the most endemic-rich family.

**Table 2:** Comparison of area size, total species richness and degree of endemism in the vascular flora of Iran versus other Mediterranean regions (Turkey, Greece, Italy, Iberian Peninsula, Morocco; data from Buira, *et al*.^40^).

Family	Iran	Turkey	Greece	Italy	Iberian Peninsula	Morocco
Area (km^2^)	1,648,195	783,562	131,957	301,338	588,824	446,550
Endemic genera	26	15	8	7	27	14
Total native species	8112	8575	5502	5935	5537	3913
Endemic species	2597(32%)	2651(31%)	1278(24%)	1050(18%)	1328(24%)	640(16%)
No of families	65	63	53	52	63	54
No. of genera	359	371	239	260	321	255
Species/Genus	5.3	7.1	5.3	4	4.1	2.5

**Table 3:** The eleven most endemic-rich families in the vascular flora of Iran and other Mediterranean regions (Turkey, Greece, Italy, Iberian Peninsula, Morocco; data from Buira, *et al*.^40^).

Family	Iran	Turkey	Greece	Italy	Iberian Peninsula	Morocco
Fabaceae	687	375	35	53	104	78
Asteraceae	618	430	204	221	203	131
Lamiaceae	155	240	68	20	108	80
Caryophyllaceae	127	187	96	72	92	35
Apiaceae	127	117	52	24	43	30
Scrophulariaceae	96	207	26	8	19	12
Brassicaceae	88	194	68	47	78	42
Alliaceae	84	50	47	18	8	2
Boraginaceae	70	108	35	21	22	12
Rosaceae	69	46	9	34	47	8
Plumbaginaceae	65	21	86	100	129	15

The ten richest genera in terms of endemic species account for 47% of the total number of endemics of Iran (Fig. 3). The largest genera of the vascular flora usually also comprise the largest number of endemic species (e.g., *Astragalus, Cousinia, Allium*). Exceptions are smaller genera with high proportion of endemics, such as *Dionysia* (Primulaceae) with 39 species and about 90% endemism (Fig. 3), and species-rich genera with few endemics, such as *Carex, Trifolium*, and *Vicia*, whose species tend to be widespread rendering those genera species-rich but endemic-poor also in other Mediterranean areas such as the Iberian Peninsula^40^.

### Richness across the territories

**Figure 4:**
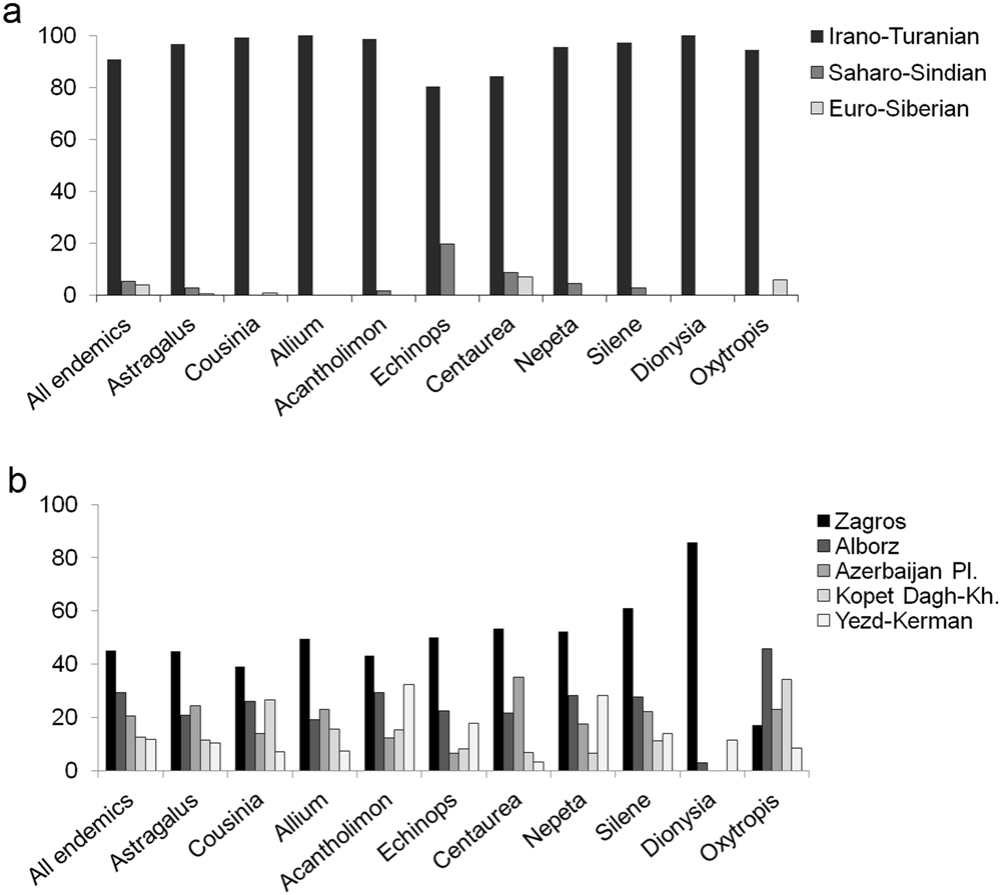
Distribution of endemic vascular plant species (total and in the 10 most endemic-rich genera) across (**a**) phytogeographical regions and (**b**) areas of endemism.

The majority of the endemic vascular plant species of Iran (88%) are restricted to the Irano-Turanian region, whereas only 5% and 4% are restricted to the Saharo-Sindian and the Euro-Siberian regions, respectively; 3% is shared between the regions (Table 4). The Irano-Turanian region is richer than the two others, which is due to the size of this region as well as its topography, as it contains numerous mountain ranges, which generally are rich in endemics^41,42^. Some of the species-rich genera more strongly represented in this region are *Astragalus* (Fabaceae), *Cousinia* (Asteraceae), *Acantholimon* (Plumbaginaceae) and *Allium* (Alliaceae; Fig. 4a). A typical representative of the Euro-Siberian region is *Alchemilla* (Rosaceae), with nearly 90% of its endemics being found in this region. Akhani, *et al*.^43^ suggests for the Hyrcanian forests (within the Euro-Siberian region) a number of ca. 280 endemic and subendemic species, which is almost three times more than we found (Table 4). As they do not provide any species list, we cannot assess the source for this discrepancy, but contributing factors may include the geographic coverage of the study by Akhani, *et al*.^43^ also including areas outside of Iran (southeastern Azerbaijan) or taxonomic differences, but still the discrepancy is remarkable. In the Saharo-Sindian region, the proportion of *Echinops* (Asteraceae) species is high compared to other regions (Fig. 4a).

**Table 4:** Vascular plant endemism in the phytogeographical regions of Iran.

Phytogeographical region	Region size (km^2^)	Iranian endemic species	Range-restricted endemic species	Iranian endemic genera
Irano-Turanian	1,769,457	2289(88%)	1239(89%)	21
Saharo-Sindian	451,052	133(5%)	88(6%)	4
Euro-Siberian	80,317	101(4%)	56(4%)	1
Shared	—	73(3%)	—	—

Patterns of endemism in global biodiversity hotspots present in Iran reflect those observed for biogeographic regions. Specifically, 84% of the Iranian vascular plant endemics are restricted to the Irano-Anatolian hotspot, which is inside the Irano-Turanian region, whereas only 4% of the Iranian vascular plant endemics are restricted to the Caucasian hotspot inside the Euro-Siberian region.

**Figure 5:**
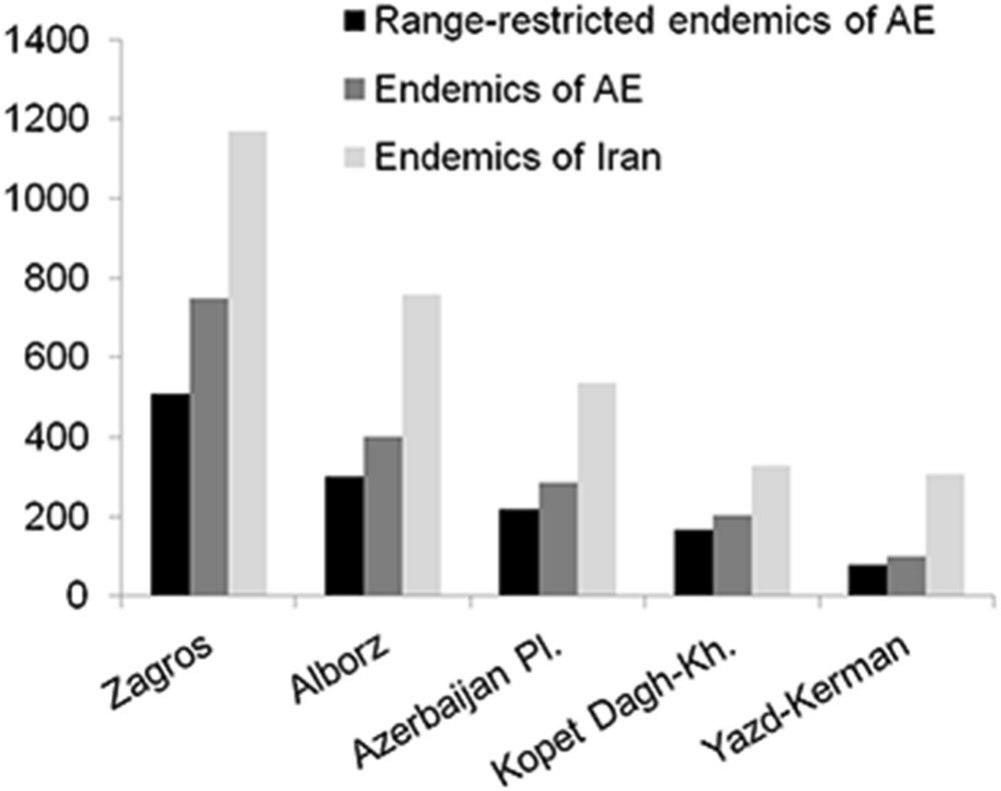
Distribution of endemic vascular plant species in general and of range-restricted endemic vascular plant species in Iran and its areas of endemism.

**Figure 6:**
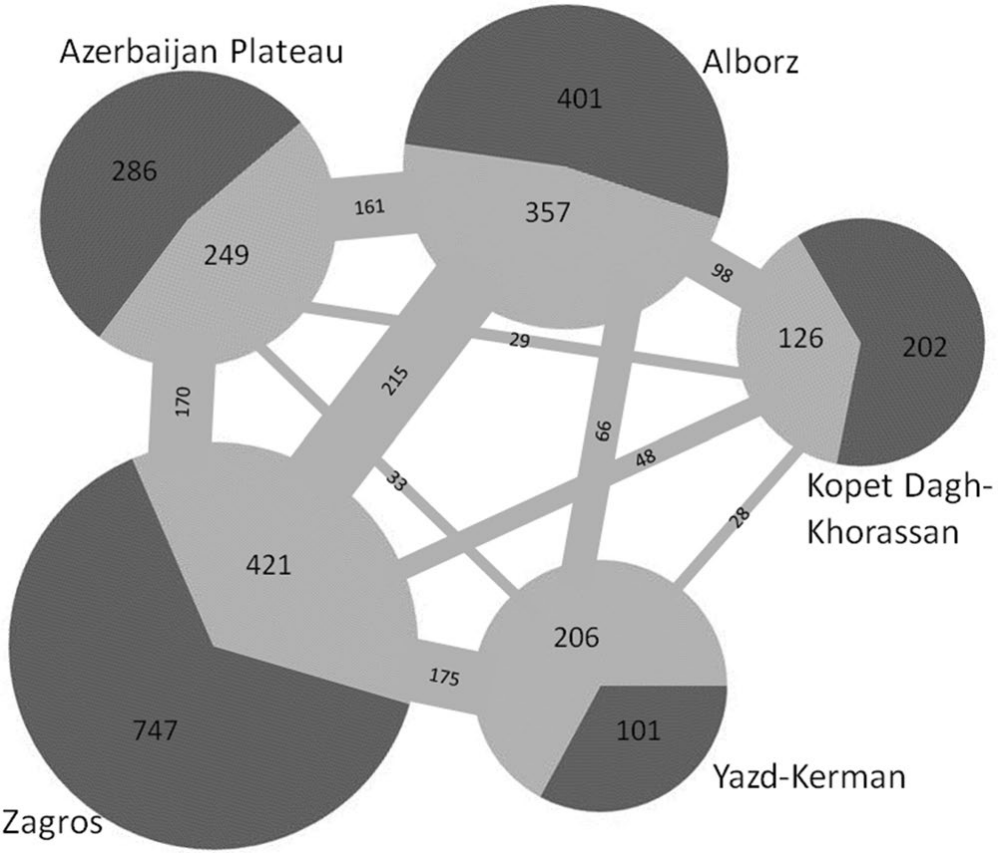
Endemic vascular plant species of Iran in areas of endemism. Endemic vascular plant species restricted to an area of endemism are indicated in dark grey, whereas those shared with other areas of endemism are indicated in light grey, their numbers being given in the bars connecting pie charts corresponding to areas of endemism.

With respect to areas of endemism, Zagros is the richest, harbouring 45% of the Iranian vascular plant endemics, the majority of which is restricted to this region (Figs 5, 6 and Table 5). Zagros is a continuous mountain range connecting the Azerbaijan Plateau and Alborz in the north to the Yazd-Kerman massifs in the south (Fig. 1). However, the connectivity is more contiguous in the montane zone and diminishes and eventually ceases with increasing elevation (Fig. 1). This increased isolation coincides with increased endemism, resulting in high endemic richness in areas with high elevational range (Noroozi *et al*. 2018), and an overall uneven distribution of endemic richness across Zagros. All of the ten most endemic-rich genera of Iran except *Oxytropis* (Fabaceae) are well-represented in Zagros (Fig. 4b).

**Table 5:** Vascular plant endemism in the areas of endemism in Iran.

AE	AE size (km^2^)	Iranian endemic species	AE endemic species	Range-restricted endemic species	Iranian endemic genera	AE endemic genera
Zagros	393,799	1,167(45%)	746(29%)	490(35%)	15	6
Alborz	62,571	758(29%)	401(15%)	260(19%)	11	1
Azerbaijan Plateau	179,693	535(21%)	286(11%)	204(15%)	8	0
Kopet Dagh-Khorassan	64,698	328(13%)	202(8%)	150(11%)	4	1
Yazd-Kerman	154,123	307(12%)	101(4%)	69(5%)	5	0

The second-richest area is Alborz, which harbours 29% of all Iranian vascular plant endemics nearly half of them being restricted to Alborz (Figs 5, 6 and Table 5). If taking area size into account, Alborz is richer than other regions. This is particularly evident for range-restricted endemics (here species which are only in maximally three grid cells of 0.5° × 0.5°), where, compared to Zagros, Alborz has half the number of endemics on one fifth the area (Fig. 5; Table 5). Generally, Central Alborz has the highest concentration of endemics in the Iranian Plateau (Noroozi *et al*. 2018). Alborz is a narrow, but very high and contiguous east-west oriented mountain range (Fig. 1) that borders on lowland deserts in the south and on the Caspian Sea in the north. A wide elevational range, high topographic complexity and strong environmental heterogeneity explain its high plant diversity and great richness of endemics^19^. Alborz connects Kopet Dagh-Khorassan mountains in the east with the Azerbaijan Plateau and Zagros in the west (evident also in the high numbers of endemic species shared between Alborz and at least one of these regions; Fig. 6), thus acting as a corridor between Central Asia and Caucasus plus Anatolian mountains. Therefore, Alborz not only harbours a high number of local endemics, but also many elements showing biogeographic connections east- and/or westwards.

In the Azerbaijan Plateau, 21% of Iranian vascular plant endemics are found, half of which are endemic to this area (Figs 5, 6 and Table 5). The Azerbaijan Plateau has a fragmented orography caused by different tectonic and volcanic activities^44^. Although three times larger than Alborz, the Azerbaijan Plateau contains fewer endemics and range-restricted endemics (Fig. 5; Table 5). This area is close to the border of Iran and is connected to eastern Turkey and the Armenian mountains. Consequently, there are many species in this area shared with eastern Turkey, Transcaucasus and Caucasus^22,45^. Moreover, it is floristically linked to Alborz in the east and to Zagros in the south (Fig. 6). Nevertheless, it is diverse in local endemics, especially at higher elevations, which likely is due to the strong level of geographic isolation of single high summits (e.g., Sahand, Sabalan, Kiamaki). The Azerbaijan Plateau is the centre of diversification of *Astragalus* (Fabaceae; Fig. 4b)^25,26^.

The Kopet Dagh-Khorassan harbours 13% of Iranian vascular plant endemics, two thirds of which are restricted to this area (Figs 5, 6 and Table 5). Although of similar size to Alborz, Kopet Dagh-Khorassan harbours only about half as many endemics. This might be due to the lower topographic complexity and the smaller elevational range. Most of the endemic species of Kopet Dagh-Khorassan are range-restricted and rare. A high proportion of those endemics is from *Cousinia* (Asteraceae; Fig. 4b), which has its centre of diversification in these mountains^38,46,47^. Floristically, Kopet Dagh-Khorassan is most closely linked to Alborz among Iranian areas of endemism (Fig. 6).

The smallest proportion of vascular plant endemics is found in the Yazd-Kerman comprising 12% of Iranian vascular plant endemics, only one third of which is restricted to this area (Figs 5, 6 and Table 5). This area comprises several high elevation areas in southern Iran, topographically and thereupon floristically well connected to southern Zagros (Fig. 6). This spatial proximity to Zagros probably explains the relatively low proportion of endemics restricted to this region. At high elevations, the number of local endemics increases considerably, especially in Hezar and Lalezar Mts.^48,49^ and Shirkuh Mts. (Noroozi *et al*., unpublished data). A typical representative of the local endemic flora is *Acantholimon* (Plumbaginaceae; Fig. 4b), which is highly diverse in this area^50,51^. Additionally, the Yazd-Kerman harbours isolated occurrences of species otherwise distributed in Hindukush and Central Asian mountains, especially in alpine regions^30,49,52,53^.

Considerable differences can be observed among areas of endemism with respect to the richness of the 10 largest genera of the Iranian endemic vascular flora (Fig. 4b), as shall be illustrated with the following examples. *Astragalus* (Fabaceae), *Allium* (Alliaceae) and *Centaurea* (Asteraceae) are well represented in the Azerbaijan Plateau, but *Acantholimon* (Plumbaginaceae), *Cousinia* (Asteraceae) and *Echinops* (Asteraceae) are underrepresented in this area. *Acantholimon*, *Echinops* and *Nepeta* (Lamiaceae) are very well represented in Yazd-Kerman and *Cousinia* is very diverse in Kopet Dagh-Khorassan. *Oxytropis* (Fabaceae) is diverse in both Alborz and Kopet Dagh-Khorassan, but very poor in Zagros. The proportion of *Silene* (Caryophyllaceae) is high in Zagros, and *Dionysia* (Primulaceae) is a Zagros element with 29 endemic species in this mountain range, three species in Yazd-Kerman and only one species in Alborz.

### Elevational distribution of endemics

**Figure 7:**
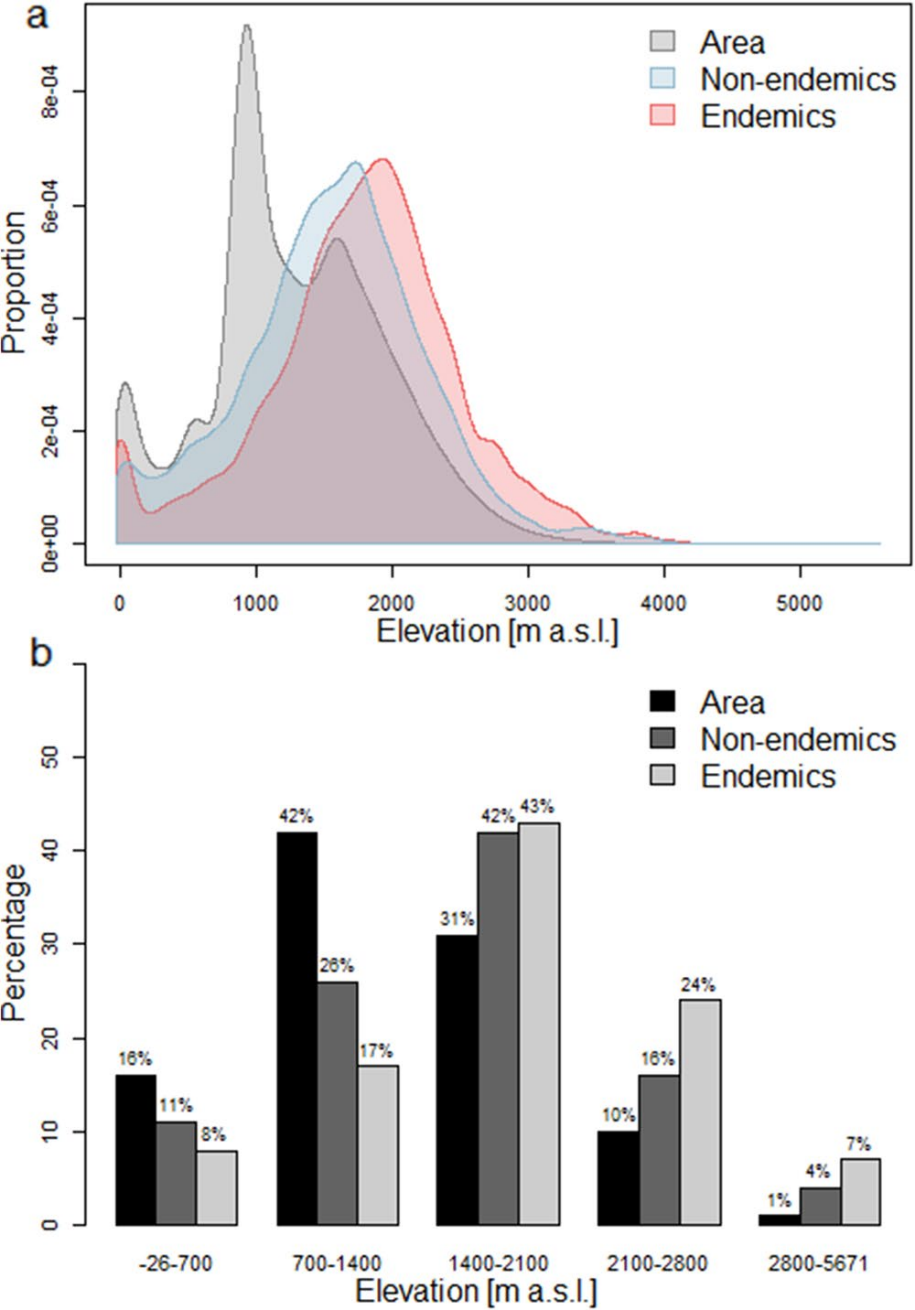
Elevational distribution of total endemic compared to non-endemic vascular flora of Iran. (**a**) Proportion of surface area, non-endemic and endemic species richness along the elevational gradient. (**b**) Percentage of surface area, non-endemic and endemic richness in different elevational zones.

The elevational distributions of surface area, non-endemic and endemic vascular plant species of Iran are displaced and peak at different elevations, as has already been found before on a taxonomically much smaller data set^19^. Despite a high proportion of surface area in lowlands (−26 to 1400 m), the proportions of non-endemics and especially of endemics in this elevation zone are low (Fig. 7). At mid elevations (1400 to 2800 m a.s.l.), the proportion of both non-endemics and endemics are high. At high elevations (2800 to 5671 m a.s.l.), although comprising only 1% of the surface area, the proportion of endemics becomes higher at the expense of the proportion of non-endemics. This confirms the importance of alpine habitats as endemism centres for vascular plants^17^.

### Life forms

**Figure 8:**
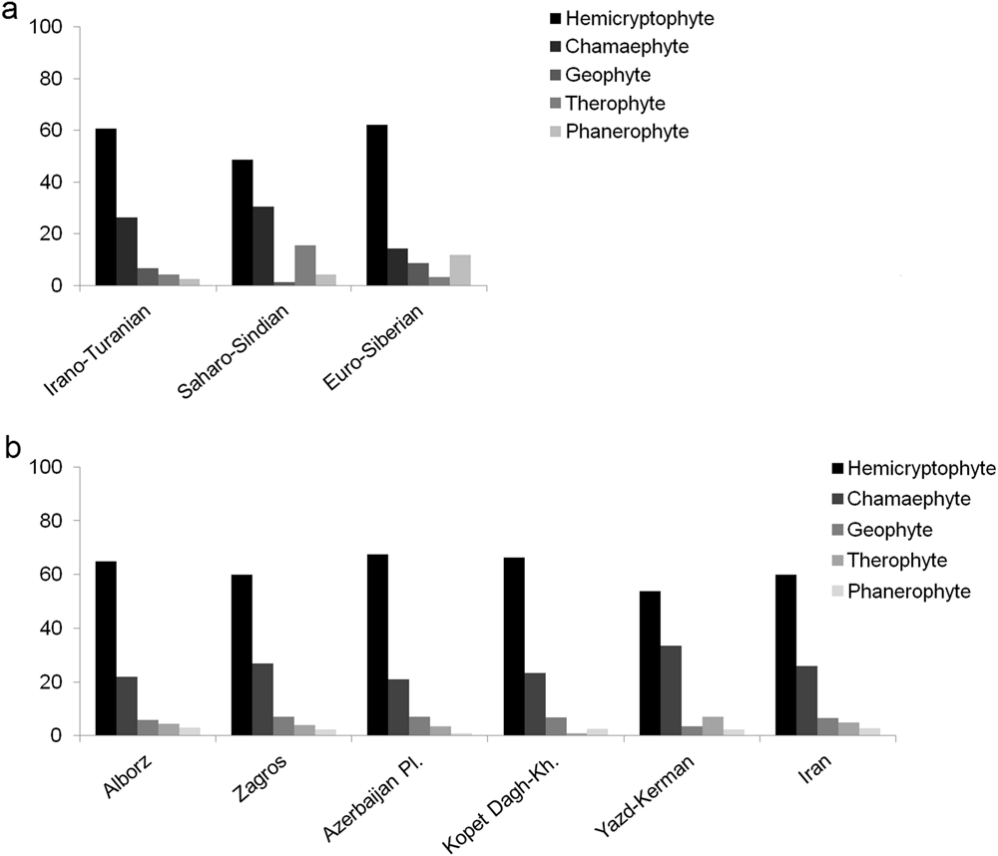
Life form spectra of the Iranian vascular flora in (**a**) phytogeographical regions and (**b**) areas of endemism.

Hemicryptophytes are the most dominant life form (60%) among the endemic vascular plant species, followed by chamaephytes (26%), geophytes (6%), therophytes (5%) and phanerophytes (3%). There are considerable differences in the proportion of life forms in the three phytogeographical regions (Fig. 8a). Hemicryptophytes are dominant to almost equal extents in all three regions, whereas chamaephytes are very poor in the Euro-Siberian region. The Euro-Siberian region with its high precipitation and temperate climate is covered by Hyrcanian forests, resulting in an overrepresentation of phanerophytes and an underrepresentation of chamaephytes, geophytes and therophytes compared to the other two regions. The majority of chamaephytes of the Iranian vascular flora are thorn-cushions, a life form adapted to windswept slopes in the regions with Mediterranean precipitation regimes^54^; such chamaephytes are dominant in the mountains of the Irano-Turanian region^54–58^. Geophytes are underrepresented and therophytes are prominent in the Saharo-Sindian region.

In areas of endemism, the life form spectra are roughly similar among the areas (Fig. 8b). Exceptions are the Yazd-Kerman area with a high proportion of therophytes, Kopet Dagh-Khorassan with a low proportion of therophytes, and the Azerbaijan Plateau with a low proportion of phanerophytes compared to the other areas or to the average for Iran (Fig. 8b). The higher proportion of therophytes in Yazd-Kerman might be due to the longer warm and dry season on these mountains, which allows penetration by elements from the Saharo-Sindian region, whose flora is rich in therophytes.

## Conclusion

Mountains influence the distribution and diversification of species and also maintain biodiversity over time^59^. Half of all the biodiversity hotspots are situated in mountains^3^, and our results indicate that 74% of all endemic vascular plant species of Iran are restricted to its mountains, which represent only about 42% of the country’s surface (elevations above 1400 m a.s.l.; Fig. 7). Generally, mountains with diverse micro-climates and topographic complexity promote high biodiversity and endemism^60,61^, which appears also to be the case for Iranian mountains^19^. The environmental heterogeneity provides diverse niche space allowing more species to coexist^62^, acts as trigger for diversification, resulting from isolation or adaptation to diverse environmental conditions^63^, and also enables the existence of particular habitats through longer time periods supporting relics^64^. The complex topography and the large elevational range potentially allowed Iranian plants to survive the Quaternary glaciations, as only the high elevations were covered with ice^65^ and lowlands could act as refugia for many relict elements such as *Parrotia* in the Hyrcanian Forests^66^. Additionally, Quaternary climatic fluctuations and associated shifts in habitats and vegetation zones may have triggered species diversification as, for instance, suggested for a group of steppe species within the hyperdiverse genus *Astragalus*^67^. However, understanding the origin of the biodiversity of the mountains of Iran requires molecular phylogenetic studies of their characteristic mega-genera. Until then, the evolutionary history of the taxa inhabiting these mountains remains one of the least understood fields of global biogeography, even though it is crucial for explaining the origin of plant diversity in mountains of Eurasia.

In Mediterranean mountain ranges, species richness has decreased during the past decade^68^. As the overall climate conditions in Iran are similar to those from Mediterranean regions, a decline of high-altitude habitats in the course of climate warming and reduced water availability can be expected in this region. Furthermore, pastoralism causes dramatic disturbance of mountain habitats of Iran. Pastoralism dates back to the Neolithic period^69,70^, since when it was extreme in several phases^71^. Pastoralism has already reduced the habitats of tree species like *Quercus macranthera* (Fagaceae) at the upper limit of the Hyrcanian forests and *Juniperus excelsa* (Cupressaceae) in the treeline zone of the southern slopes of Alborz^72^. Thus, with increasing anthropogenic pressure via pastoralism, urbanization, and road construction as well as ongoing global warming, mountain species are under increasing threat and need to be more strongly protected. According to the IUCN Red List, about one hundred species of the vertebrate fauna in Iran are considered vulnerable or already endangered^73^. For plants, nearly 60% of endemic vascular plant species of Iran are range-restricted and can be categorized as IUCN threatened species. Our knowledge about the centres of endemism in the Iranian Plateau is, however, still limited and future efforts will be needed to identify hotspots at a finer scale, “hotspots-within-hotspots”^8,74,75^, to aid practical conservation management.

## Materials and Methods

### Study area

Iran, with a surface area of c. 1.6 million km², is located between Central Asia and Himalaya in the east and Caucasus and Anatolia in the west. Iran displays considerable geologic and lithospheric heterogeneity, owing to its complex tectonic history^76^. One of the main tectonic events that influenced the geology and topography of Iran is the Arabia-Eurasia collision, which caused the uplift of numerous mountain ranges in the region, especially between the middle Miocene and the Pliocene^77^. The five major mountainous areas of Iran are the Azerbaijan Plateau, Alborz, Kopet Dagh-Khorassan, Zagros and the Yazd-Kerman massifs which are well associated with five areas of endemism (Fig. 1b). The elevation in Iran ranges from 26 m b.s.l. along the shore of the Caspian Sea up to 5,671 m a.s.l. at Damavand Mt. in Central Alborz. The climate is diverse and ranges from hot and dry deserts with precipitation of less than 25 mm/yr in central Iran to sub-tropical humid climates at the southern shore of the Caspian Sea with precipitation exceeding 1,800 mm/yr^78^. Nevertheless, major parts of Iran are characterised by continental climate with hot and dry summers, cold and harsh winters, and low precipitation^66,79^. Based on the Global Bioclimatic Classification System^80,81^ Iran is at the crossroad of three macrobioclimates (i.e. Mediterranean, tropical and temperate), correlating with the Irano-Turanian, Saharo-Sindian and Euro-Siberian biogeographical regions, respectively^79,82^.

Diverse climate and topography are paralleled by a multitude of vegetation types including desert and semidesert steppes, montane grasslands, wetlands, subalpine, alpine and subnival habitats, different types of shrublands and woodlands, deciduous temperate to subtropical forests, halophyte and even mangrove vegetation types^66^. These vegetation types are distributed in different elevational zones from 26 m b.s.l. up to 4,850 m a.s.l.^72^. Most of the biodiversity of Iran is centred within the two global biodiversity hotspots, i.e. the Irano-Anatolian and Caucasus hotspots (Fig. 1a), on five groups of mountain ranges (Fig. 1b). Iran covers 54% of the Irano-Anatolian hotspot and around 10% of the Caucasus hotspot (Fig. 1a). The species richness is not evenly distributed over the country and five areas of endemism have been identified, all of which are located in the Irano-Anatolian hotspot, and are well associated with major mountain ranges Fig. 1b;^19^. Azerbaijan Plateau, Alborz, Central Alborz, Zagros, the and Kopet Dagh-Khorassan were identified as areas of endemism in the Iranian Plateau based on data from Asteraceae^19^. Using the same approach (endemicity analysis) on the entire endemic vascular flora of Iran and a finer grid cell size, Yazd-Kerman is identified as an additional area of endemism (Noroozi *et al*., unpublished data), and is considered as such in this study. The Talysh mountains, which are located between Alborz and Azerbaijan, have a transitional situation, but their vascular flora is more linked to the Azerbaijan Plateau than to Alborz (Noroozi *et al*., unpublished data).

### Species distribution data

All endemic and subendemic vascular plant species of Iran were documented. A species was considered endemic if its range is restricted to Iran, and considered subendemic if its main distribution (>80% of the known range or occurrences) lies within this country. We considered only taxa at the species level, but not subspecies or varieties. The documentation of species and the characterization of their geographical and altitudinal ranges were based on Flora Iranica^83^, Flora of Iran^84^ and monographs published after these floras until the end of 2016 (see Appendix S1, S2). The Flora Iranica taxonomic system is followed for family and genus level. The localities of all species were geo-referenced with a precision of at least 0.25° using Google Earth. Presence of species was then recorded on the basis of a grid with cell size of 0.5° × 0.5°. Species present in maximally three grid cells were considered as range-restricted endemics even if the grid cells were non-adjacent. We compared area size of Iran, number of total vascular plant species, number of vascular plant endemic species and the ten largest vascular plant families of Iran with those from other countries and regions in the west with similar climate, i.e. Mediterranean (Turkey, Greece, Italy, Morocco and Iberian Peninsula). The number of endemic vascular plant species of Iran and their restriction to the three phytogeographical regions^18^, two biodiversity hotspots^7^, and five areas of endemism^19^ (but not distinguishing Central Alborz, as it is geographically nested within Alborz, but additionally recognizing Yazd-Kerman) were analysed. Using the life form system of Raunkiaer^85^, the following five categories were used: chamaephytes, geophytes, hemicryptophytes, phanerophytes, and therophytes.

## Supplementary information

Supplementary file

## References

[1] Anderson S (1994). Area and endemism. The Quarterly Review of Biology.

[2] Lamoreux JF (2006). Global tests of biodiversity concordance and the importance of endemism. Nature.

[3] Myers N, Mittermeier RA, Mittermeier CG, da Fonseca GAB, Kent J (2000). Biodiversity hotspots for conservation priorities. Nature.

[4] Riemann H, Ezcurra E (2005). Plant endemism and natural protected areas in the peninsula of Baja California, Mexico. Biological Conservation.

[5] Gotelli NJ (2009). Patterns and causes of species richness: a general simulation model for macroecology. Ecology Letters.

[6] Lévêque, C. & Mounolou, J.-C. *Biodiversity* (Wiley & Sons Ltd., 2007).

[7] Mittermeier, R. A. *et al*. *Hotspots Revisited: Earth’s Biologically Richest and Most Endangered Terrestrial Ecoregions* (Conservation International, 2005).

[8] Cañadas EM (2014). Hotspots within hotspots: Endemic plant richness, environmental drivers, and implications for conservation. Biological Conservation.

[9] Morrone JJ (2018). The spectre of biogeographical regionalization. Journal of Biogeography.

[10] Treurnicht M, Colville JF, Joppa LN, Huyser O, Manning J (2017). Counting complete? Finalising the plant inventory of a global biodiversity hotspot. PeerJ.

[11] Marshall CAM, Wieringa JJ, Hawthorne WD (2016). Bioquality hotspots in the tropical African flora. Current Biology.

[12] Harold AS, Mooi RD (1994). Areas of endemism: definition and recognition criteria. Systematic Biology.

[13] Morrone, J. J. *Evolutionary Biogeography: An Integrative Approach with Case**Studies* (Columbia University Press, 2008).

[14] Firouz, E. *The Complete Fauna of Iran* (Tauris, I. B., 2005).

[15] Frey, W., Kürschner, H. & Probst, W. In *Encyclopaedia* Iranica (ed Yarshater, E.) 43–63 (Mazda Publishers, Costa Mesa, 1999).

[16] Zohary, M. In *Plant Life of South-West Asia* (eds Davis, P. H., Harper. P. C., & Hedge, I. C.) 43–52 (Botanical Society of Edinburgh, 1971).

[17] Noroozi J, Moser D, Essl F (2016). Diversity, distribution, ecology and description rates of alpine endemic plant species from Iranian mountains. Alpine Botany.

[18] White F, Léonard J (1991). Phytogeographical links between Africa and Southwest Asia. Flora et Vegetatio Mundi.

[19] Noroozi J (2018). Hotspots within a global biodiversity hotspot - areas of endemism are associated with high mountain ranges. Scientific Reports.

[20] Manafzadeh S, Salvo G, Conti E (2014). A tale of migrations from east to west: the Irano-Turanian floristic region as a source of Mediterranean xerophytes. Journal of Biogeography.

[21] Akhani H (2004). A new spiny, cushion-like *Euphorbia* (Euphorbiaceae) from south-west Iran with special reference to the phytogeographic importance of local endemic species. Botanical Journal of the Linnean Society.

[22] Akhani H (2007). Diversity, biogeography, and photosynthetic pathways of *Argusia* and *Heliotropium* (Boraginaceae) in South-West Asia with an analysis of phytogeographical units. Botanical Journal of the Linnean Society.

[23] Freitag H (1986). Notes on the distribution, climate and flora of the sand deserts of Iran and Afghanistan. Proceedings of the Royal Society of Edinburgh, Section B: Biological Sciences.

[24] Hedge IC, Wendelbo P (1978). Patterns of distribution and endemism in Iran. Notes from the Royal Botanic Garden, Edinburgh.

[25] Mahmoodi M, Maassoumi AA, Hamzeh’ee B (2009). Geographical distribution of *Astragalus* (Fabaceae) in Iran. Rostaniha.

[26] Mahmoodi M, Maassoumi AA, Jalili A (2012). Distribution patterns of *Astragalus* in the Old World based on some selected sections. Rostaniha.

[27] Memariani F, Akhani H, Joharchi MR (2016). Endemic plants of Khorassan-Kopet Dagh floristic province in Irano-Turanian region: diversity, distribution patterns and conservation status. Phytotaxa.

[28] Memariani F, Zarrinpour V, Akhani H (2016). A review of plant diversity, vegetation, and phytogeography of the Khorassan-Kopet Dagh floristic province in the Irano-Turanian region (northeastern Iran–southern Turkmenistan). Phytotaxa.

[29] Noroozi J, Akhani H, Breckle S-W (2008). Biodiversity and phytogeography of the alpine flora of Iran. Biodiversity and Conservation.

[30] Noroozi J, Pauli H, Grabherr G, Breckle S-W (2011). The subnival–nival vascular plant species of Iran: a unique high-mountain flora and its threat from climate warming. Biodiversity and Conservation.

[31] Sales F, Hedge IC (2013). Generic endemism in South-West Asia: an overview. Rostaniha.

[32] Wendelbo, P. In *Plant Life of South-West Asia* (eds Davis, P. H., Harper, P. C., & Hedge, I. C.) 29–41 (Botanical Society of Edinburgh., 1971).

[33] Al-Shehbaz IA (2012). A generic and tribal synopsis of the Brassicaceae (Cruciferae). Taxon.

[34] Hedge, I. C. In *The Biology and Chemistry of the* Cruciferae (eds J.G. Vaughan, A. J. Mac Leod, & B. M. G. Jones) 1–45 (Academic Press, 1976).

[35] Moazzeni H, Zarre S, Al-Shehbaz IA, Mummenhoff K (2007). Seed-coat microsculpturing and its systematic application in *Isatis* (Brassicaceae) and allied genera in Iran. Flora.

[36] Mummenhoff K, Al-Shehbaz IA, Bakker FT, Linder HP, Mühlhausen A (2005). Phylogeny, morphological evolution, and speciation of endemic Brassicaceae genera in the Cape flora of southern Africa. Annals of the Missouri Botanical Garden.

[37] Li J, Del Tredici P (2008). The Chinese *Parrotia*: a sibling species of the Persian *Parrotia*. Arnoldia.

[38] Mehregan I, Kadereit JW (2008). Taxonomic revision of *Cousinia* sect. *Cynaroideae* (Asteraceae, Cardueae). Willdenowia.

[39] Hobohm, C. *Endemism in Vascular**Plants*. (Springer, 2014).

[40] Buira A, Aedo C, Medina L (2017). Spatial patterns of the Iberian and Balearic endemic vascular flora. Biodiversity and Conservation.

[41] Hobohm, C. & Tucker, C. M. In *E*nd*emi*sm in *Va*scula*r Plants* (ed Carsten Hobohm) 3–9 (Springer, 2014).

[42] Spehn EM, Rudmann-Maurer K, Körner C (2011). Mountain biodiversity. Plant Ecology & Diversity.

[43] Akhani H, Djamali M, Ghorbanalizadeh A, Ramezani E (2010). Plant biodiversity of Hyrcanian relict forests, N Iran: An overview of the flora, vegetation, palaeoecology and conservation. Pakistan Journal of Botany.

[44] Innocenti, F., Manetti, P., Mazzuoli, R., Pasquaré, G. & Villari, L. In *Andesites: Orogenic Andesites and Related* Rocks (ed. Thorpe, R. S.) 327–349 (John Wiley & Sons, 1982).

[45] Noroozi J, Willner W, Pauli H, Grabherr G (2014). Phytosociology and ecology of the high-alpine to subnival scree vegetation of N and NW Iran (Alborz and Azerbaijan Mts.). Applied Vegetation Science.

[46] Knapp HD (1987). On the distribution of the genus *Cousinia* (Compositae). Plant Systematics and Evolution.

[47] López-Vinyallonga S, Mehregan I, Garcia-Jacas N, Kadereit JW (2009). Phylogeny and evolution of the *Arctium*-*Cousinia* complex (Compositae,Cardueae-Carduinae). Taxon.

[48] Noroozi J, Ajani Y, Nordenstam B (2010). A new annual species of *Senecio* (Compositae-Senecioneae) from subnival zone of southern Iran with comments on phytogeographical aspects of the area. Compositae Newsletter.

[49] Rajaei P, Maassoumi AA, Mozaffarian V, Nejad Sattari T, Pourmirzaei A (2011). Alpine flora of Hezar mountain (SE Iran). Rostaniha.

[50] Assadi M (2006). Distribution patterns of the genus *Acantholimon* (Plumbaginaceae) in Iran. Iranian Journal of Botany.

[51] Moharrek F, Kazempour-Osaloo S, Assadi M, Feliner GN (2017). Molecular phylogenetic evidence for a wide circumscription of a characteristic Irano-Turanian element: *Acantholimon* (Plumbaginaceae: Limonioideae). Botanical Journal of the Linnean Society.

[52] Ajani Y, Noroozi J, Levichev IG (2010). *Gagea alexii* (Liliaceae), a new record from subnival zone of southern Iran with key and notes on sect. Incrustatae. Pakistan Journal of Botany.

[53] Doostmohammadi M, Kilian N (2017). *Lactuca pumila* (Asteraceae, Cichorieae) revisited—additional evidence for a phytogeographical link between SE Zagros and Hindu Kush. Phytotaxa.

[54] Kürschner H (1986). The subalpine thorn-cushion formations of western South-West Asia: ecology, structure and zonation. Proceedings of the Royal Society of Edinburgh. Section B. Biological Sciences.

[55] Klein JC (1987). Les pelouses xérophiles d’‏altitude du flanc sud de l’Alborz central (Iran). Phytocoenologia.

[56] Klein JC (1988). Les groupements à grandes ombellifères et à xérophytes orophiles: Essai de synthèse à l’échelle de la région irano-touranienne. Phytocoenologia.

[57] Noroozi J, Akhani H, Wıllner W (2010). Phytosociological and ecological study of the high alpine vegetation of Tuchal Mountains (Central Alborz, Iran). Phytocoenologia.

[58] Noroozi J, Hülber K, Willner W (2017). Phytosociological and ecological description of the high alpine vegetation of NW Iran. Phytocoenologia.

[59] Hoorn, C., Perrigo, A. & Antonelli, A. *Mountains, Climate and Biodiversity* (Wiley-Blackwell, 2018).

[60] Irl SDH (2015). Climate vs. topography – spatial patterns of plant species diversity and endemism on a high-elevation island. Journal of Ecology.

[61] Steinbauer MJ (2016). Topography-driven isolation, speciation and a global increase of endemism with elevation (Forthcoming). Global Ecology and Biogeography.

[62] Tews J (2004). Animal species diversity driven by habitat heterogeneity/diversity: the importance of keystone structures. Journal of Biogeography.

[63] Hughes C, Eastwood R (2006). Island radiation on a continental scale: Exceptional rates of plant diversification after uplift of the Andes. Proceedings of the National Academy of Sciences.

[64] Fjeldså J, Bowie RCK, Rahbek C (2012). The role of mountain ranges in the diversification of birds. Annual Review of Ecology, Evolution, and Systematics.

[65] Becker, D., Verheul, J., Zickel, M. & Willmes, C. LGM paleoenvironment of Europe - Map. CRC806-Database, 10.5880/SFB806.15 (2015).

[66] Zohary, M. *Geobotanical Foundations of the Middle East 2*. (Gustav Fischer, 1973).

[67] Bagheri A, Maassoumi AA, Rahiminejad MR, Brassac J, Blattner FR (2017). Molecular phylogeny and divergence times of *Astragalus* section *Hymenostegis*: An analysis of a rapidly diversifying species group in Fabaceae. Scientific Reports.

[68] Pauli H (2012). Recent plant diversity changes on Europe’s mountain summits. Science.

[69] Diamond J (2002). Evolution, consequences and future of plant and animal domestication. Nature.

[70] Zeder MA, Hesse B (2000). The initial domestication of goats (*Capra hircus*) in the Zagros Mountains 10,000 years ago. Science.

[71] Djamali M (2009). A late Holocene pollen record from Lake Almalou in NW Iran: evidence for changing land-use in relation to some historical events during the last 3700 years. Journal of Archaeological Science.

[72] Noroozi J, Körner C (2018). A bioclimatic characterization of high elevation habitats in the Alborz mountains of Iran. Alpine Botany.

[73] Jowkar H, Ostrowski S, Tahbaz M, Zahler P (2016). The conservation of biodiversity in Iran: threats, challenges and hopes. Iranian Studies.

[74] Harris GM, Jenkins CN, Pimm SL (2005). Refining biodiversity conservation priorities. Conservation Biology.

[75] Murray-Smith C (2009). Plant diversity hotspots in the Atlantic coastal forests of Brazil. Conservation Biology.

[76] Stöcklin J (1968). Structural history and tectonics of Iran; a review. AAPG Bulletin.

[77] Berberian M, King GCP (1981). Towards a paleogeography and tectonic evolution of Iran. Canadian Journal of Earth Sciences.

[78] Djamali, M. *Palaeoenvironmental Changes in Iran During the Last Two Climatic Cycles (Vegetation-Climate-Anthropisation). Unpublished PhD thesis*. (Aix-Marseille, 2008).

[79] Djamali M, Brewer S, Breckle SW, Jackson ST (2012). Climatic determinism in phytogeographic regionalization: A test from the Irano-Turanian region, SW and Central. Asia. Flora.

[80] Rivas-Martínez S (1997). Syntaxonomical synopsis of the potential natural plant communities of North America, I:(Compedio sintaxonómico de la vegetación natural potencial de Norteamérica, I). Itinera Geobotanica.

[81] Rivas-Martínez S, Sánchez-Mata D, Costa M (1999). Boreal and western Temperate forest vegetation (syntaxonomical synopsis of the potential natural plant communities of North America II). Itinera Geobotanica.

[82] Djamali M (2011). Application of the Global Bioclimatic Classification to Iran: implications for understanding the modern vegetation and biogeography. Ecologia Mediterranea.

[83] Rechinger, K. H. *Flora Iranica*. Vol. 1–181 (Akad. Druck- Verlagsanstalt. & Naturhist. Mus. Wien, 1963–2015).

[84] Assadi, M., Khatamsaz, M., Maassoumi, A. A. & Mozaffarian, V. *Flora of Iran*. (Research Institute of Forests & Rangelands, 1989–2018).

[85] Raunkiaer, C. *The Life Forms of Plants and Statistical Plant**Geography*. (Clarendon Press, 1934).

