# Supplementary material for: Endemic diversity and distribution of the Iranian vascular flora across phytogeographical regions, biodiversity hotspots and areas of endemism
Source: Sci Rep. 2019 Sep 10;9:12991. doi: 10.1038/s41598-019-49417-1 (PMC6737179; doi:10.1038/s41598-019-49417-1)
Supplement: Supplementary file 1 — Supplementary file [file 41598_2019_49417_MOESM1_ESM.pdf]

Electronic Supplementary Material

Article title: **Endemic diversity and distribution of the Iranian vascular flora across phytogeographical regions, biodiversity hotspots and areas of endemism**

Jalil Noroozi[1]*, Amir Talebi[2], Moslem Doostmohammadi[2], Sara Manafzadeh[1], Zahra Asgarpour[1], Gerald M. Schneeweiss[1]

[1]Department of Botany and Biodiversity Research, University of Vienna, Vienna, Austria.

[2]Department of Plant Science, University of Tehran, Tehran, Iran.

*Correspondence: jalil.noroozi@univie.ac.at

Table S1. A total of 2,597 endemic vascular plant species of Iran, their presence in three phytogrographical regions and in five areas of endemism, their life forms, and range-restricted species. Species without entry in the column ñAreas of endemismò are found exclusively outside any of the five areas of endemism.

Nr	Species	Family	Phytogeographic region	Area of endemism	Life form	Range-restricted sp.
1	Acantholimon acmostegium Boiss. & Buhse	Plumbaginaceae	Irano-Turanian	Al, Ko	Chamaephyte	NO
2	Acantholimon alavae Rech.f. & Schiman-Czeika	Plumbaginaceae	Irano-Turanian	Ko	Chamaephyte	YES
3	Acantholimon albocalycinum Assadi & Mirtadz.	Plumbaginaceae	Irano-Turanian	Ke	Chamaephyte	YES
4	Acantholimon aspadanum Bunge	Plumbaginaceae	Irano-Turanian	Za	Chamaephyte	NO
5	Acantholimon asphodelinum Mobayen	Plumbaginaceae	Irano-Turanian	Za	Chamaephyte	NO
6	Acantholimon atropatanum Bunge	Plumbaginaceae	Irano-Turanian	Az	Chamaephyte	NO
7	Acantholimon austro-iranicum Rech.f. & Schiman-Czeika	Plumbaginaceae	Irano-Turanian	Ke	Chamaephyte	NO
8	Acantholimon bakhtiaricum Assadi	Plumbaginaceae	Irano-Turanian	Za	Chamaephyte	YES
9	Acantholimon bodeanum Bunge	Plumbaginaceae	Irano-Turanian	Al, Ko	Chamaephyte	NO
10	Acantholimon brachystachyum Boiss. ex Bunge	Plumbaginaceae	Irano-Turanian	Al, Za	Chamaephyte	NO
11	Acantholimon bromifolium Boiss. ex Bunge	Plumbaginaceae	Irano-Turanian	Za	Chamaephyte	NO
12	Acantholimon cephalotoides Rech.f.	Plumbaginaceae	Irano-Turanian	Al	Chamaephyte	NO
13	Acantholimon chlorostegium Rech.f. & Schiman-Czeika	Plumbaginaceae	Irano-Turanian	Ke	Chamaephyte	NO
14	Acantholimon collare Köie & Rech.f.	Plumbaginaceae	Irano-Turanian	Al, Lo	Chamaephyte	NO
15	Acantholimon cupreo-olivascens Rech.f. & Schiman-Czeika	Plumbaginaceae	Irano-Turanian	Ke	Chamaephyte	YES
16	Acantholimon curviflorum Bunge	Plumbaginaceae	Irano-Turanian	Za	Chamaephyte	NO
17	Acantholimon cymosum Bunge	Plumbaginaceae	Irano-Turanian	Al	Chamaephyte	YES
18	Acantholimon demavendicum Bornm.	Plumbaginaceae	Irano-Turanian	Al	Chamaephyte	NO
19	Acantholimon densiflorum Assadi	Plumbaginaceae	Irano-Turanian	Az	Chamaephyte	YES
20	Acantholimon eschkerense Boiss. & Hausskn.	Plumbaginaceae	Irano-Turanian	Za	Chamaephyte	NO
21	Acantholimon festucaceum (Jaub. & Spach) Boiss.	Plumbaginaceae	Irano-Turanian	Al, Za, Az	Chamaephyte	NO
22	Acantholimon flabellum Assadi	Plumbaginaceae	Irano-Turanian	Za, Ko	Chamaephyte	YES
23	Acantholimon flexuosum Boiss. & Hausskn. ex Bunge	Plumbaginaceae	Irano-Turanian	Al, Za, Ke	Chamaephyte	NO
24	Acantholimon gadukense Mobayen	Plumbaginaceae	Irano-Turanian	Al	Chamaephyte	YES

25	Acantholimon gilliatii Turrill	Plumbaginaceae	Irano-Turanian	Al, Az	Chamaephyte	NO
26	Acantholimon glabratum Assadi	Plumbaginaceae	Irano-Turanian	Za	Chamaephyte	YES
27	Acantholimon gorganense Mobayen	Plumbaginaceae	Irano-Turanian	Ko	Chamaephyte	NO
28	Acantholimon gulistanum Bunge	Plumbaginaceae	Irano-Turanian	Ko	Chamaephyte	YES
29	Acantholimon haesarensis Bornm. ex Rech.f. & Schiman-Czeika	Plumbaginaceae	Irano-Turanian	Ke	Chamaephyte	YES
30	Acantholimon hormozganense Assadi	Plumbaginaceae	Saharo-Sindian	Ke	Chamaephyte	YES
31	Acantholimon horridum Bunge	Plumbaginaceae	Irano-Turanian	Ke	Chamaephyte	YES
32	Acantholimon hystrix Stapf	Plumbaginaceae	Irano-Turanian	Za	Chamaephyte	YES
33	Acantholimon incomptum Boiss. & Buhse	Plumbaginaceae	Irano-Turanian	Al, Ke, Lo	Chamaephyte	NO
34	Acantholimon kermanense Assadi & Mirtadz.	Plumbaginaceae	Irano-Turanian	Ke	Chamaephyte	NO
35	Acantholimon melananthum Boiss.	Plumbaginaceae	Irano-Turanian	Za	Chamaephyte	NO
36	Acantholimon mirtadzadinii Assadi	Plumbaginaceae	Irano-Turanian	Ke	Chamaephyte	YES
37	Acantholimon mishaudaghense Mobayen	Plumbaginaceae	Irano-Turanian	Az	Chamaephyte	YES
38	Acantholimon mobayenii Assadi & Ghahr.	Plumbaginaceae	Irano-Turanian	Za	Chamaephyte	YES
39	Acantholimon modestum Bornm. ex Rech.f. & Schiman-Czeika	Plumbaginaceae	Irano-Turanian	Ke	Chamaephyte	NO
40	Acantholimon moradii Assadi	Plumbaginaceae	Irano-Turanian	Za	Chamaephyte	YES
41	Acantholimon nigricans Mobayen	Plumbaginaceae	Irano-Turanian	Za, Ke	Chamaephyte	YES
42	Acantholimon oliganthum Boiss.	Plumbaginaceae	Irano-Turanian	Za, Ke	Chamaephyte	NO
43	Acantholimon olivieri (Jaub. & Spach) Boiss.	Plumbaginaceae	Irano-Turanian	Za	Chamaephyte	NO
44	Acantholimon ophiocladus Rech.f. & Schiman-Czeika	Plumbaginaceae	Irano-Turanian	Al, Za	Chamaephyte	YES
45	Acantholimon pterostegium Bunge	Plumbaginaceae	Irano-Turanian	Ko	Chamaephyte	NO
46	Acantholimon quinquelobum Bunge	Plumbaginaceae	Irano-Turanian	Al, Ko	Chamaephyte	NO
47	Acantholimon restiaceum Bunge	Plumbaginaceae	Irano-Turanian	Ko	Chamaephyte	NO
48	Acantholimon rhodopolium Rech.f. & Schiman-Czeika	Plumbaginaceae	Irano-Turanian	Za	Chamaephyte	YES
49	Acantholimon scabrellum Boiss. & Hausskn.	Plumbaginaceae	Irano-Turanian	Za	Chamaephyte	NO
50	Acantholimon schahrudicum Bunge	Plumbaginaceae	Irano-Turanian	Al, Za, Ke	Chamaephyte	NO
51	Acantholimon schirazianum Boiss.	Plumbaginaceae	Irano-Turanian	Za	Chamaephyte	YES
52	Acantholimon scirpinum Bunge	Plumbaginaceae	Irano-Turanian	Ke	Chamaephyte	NO
53	Acantholimon scorpius (Jaub. & Spach) Boiss.	Plumbaginaceae	Irano-Turanian	Al, Za, Ke, Az	Chamaephyte	NO
54	Acantholimon senganense Bunge	Plumbaginaceae	Irano-Turanian	Al	Chamaephyte	YES
55	Acantholimon serotinum Rech.f. & Schiman-Czeika	Plumbaginaceae	Irano-Turanian	Za, Ke	Chamaephyte	NO

56	Acantholimon sirchense Assadi & Mirtadz.	Plumbaginaceae	Irano-Turanian	Ke	Chamaephyte	YES
57	Acantholimon spinicalyx Köie & Rech.f.	Plumbaginaceae	Irano-Turanian	Ko, Ke	Chamaephyte	NO
58	Acantholimon talagonicum Boiss.	Plumbaginaceae	Irano-Turanian	Al, Za	Chamaephyte	NO
59	Acantholimon termei Rech.f. & Schiman-Czeika	Plumbaginaceae	Irano-Turanian	Al	Chamaephyte	YES
60	Acantholimon tomentellum Boiss.	Plumbaginaceae	Irano-Turanian	Za	Chamaephyte	NO
61	Acantholimon tragacanthinum (Jaub. & Spach) Boiss.	Plumbaginaceae	Irano-Turanian	Az	Chamaephyte	YES
62	Acantholimon viscidulum Boiss.	Plumbaginaceae	Irano-Turanian		Chamaephyte	YES
63	Acantholimon wendelboi Rech.f. & Schiman-Czeika	Plumbaginaceae	Irano-Turanian	Za	Chamaephyte	NO
64	Acantholimon wilhelminae Rech.f. & Schiman-Czeika	Plumbaginaceae	Irano-Turanian	Az	Chamaephyte	YES
65	Acantholimon zaeifii Assadi	Plumbaginaceae	Irano-Turanian	Ke	Chamaephyte	NO
66	Acanthophyllum andersenii Schiman-Czeika	Caryophyllaceae	Irano-Turanian	Ko	Chamaephyte	YES
67	Acanthophyllum chloroleucum Rech.f. & Aell.	Caryophyllaceae	Irano-Turanian	Ko	Chamaephyte	YES
68	Acanthophyllum crassifolium Boiss.	Caryophyllaceae	Irano-Turanian	Al, Za, Az	Chamaephyte	NO
69	Acanthophyllum ejtehadii Mahmoudi & Vaezi	Caryophyllaceae	Irano-Turanian	Ko	Chamaephyte	YES
70	Acanthophyllum leucostegium Schiman-Czeika	Caryophyllaceae	Irano-Turanian, Saharo-Sindian	Za, Ke	Chamaephyte	NO
71	Acanthophyllum pachycephalum Schiman-Czeika	Caryophyllaceae	Irano-Turanian	Al, Za, Ko	Chamaephyte	NO
72	Acanthophyllum yasamin-nassehiae Joharchi & Pirani	Caryophyllaceae	Irano-Turanian	Ko	Chamaephyte	YES
73	Acer mazandaranicum Amini, H.Zare & Assadi	Aceraceae	Euro-Siberian	Al	Phanerophyte	YES
74	Achillea aucheri Boiss.	Asteraceae	Irano-Turanian	Al, Az	Hemicryptophyte	NO
75	Achillea callichroa Boiss.	Asteraceae	Irano-Turanian	Za	Hemicryptophyte	YES
76	Achillea eriophora DC.	Asteraceae	Irano-Turanian, Saharo-Sindian	Za, Ke	Hemicryptophyte	NO
77	Achillea kellalensis Boiss. & Hausskn.	Asteraceae	Irano-Turanian	Za	Hemicryptophyte	YES
78	Achillea oxyodonta Boiss.	Asteraceae	Irano-Turanian	Al, Za	Hemicryptophyte	NO
79	Achillea pachycephala Rech.f.	Asteraceae	Irano-Turanian, Euro-Siberian	Ko, Al	Hemicryptophyte	NO
80	Achillea talagonica Boiss.	Asteraceae	Irano-Turanian	Al, Za, Az, Ke	Hemicryptophyte	NO
81	Aconitum iranshahrii Riedl	Ranunculaceae	Irano-Turanian	Al	Geophyte	YES
82	Adonis globosa C.H.Steinb. ex Rech.f.	Ranunculaceae	Irano-Turanian	Za	Hemicryptophyte	NO
83	Aeluropus laciniatus Khodash.	Poaceae	Irano-Turanian	Ko	Geophyte	YES
84	Aeluropus peterganicus Khodash.	Poaceae	Irano-Turanian		Geophyte	YES

85	Aethionema cephalanthum (Bornm.) Bornm.	Brassicaceae	Irano-Turanian	Za	Hemicryptophyte	YES
86	Aethionema sabzevaricum Khosravi & Joharchi	Brassicaceae	Irano-Turanian		Hemicryptophyte	YES
87	Aethionema semnanensis Mozaff.	Brassicaceae	Irano-Turanian	Al	Chamaephyte	YES
88	Aethionema stenopterum Boiss.	Brassicaceae	Irano-Turanian	Al, Za	Hemicryptophyte	NO
89	Aethionema umbellatum (Boiss.) Bornm.	Brassicaceae	Irano-Turanian	Za, Ke	Hemicryptophyte	NO
90	Agropyron afghanicum Melderis	Poaceae	Irano-Turanian	Za	Hemicryptophyte	YES
91	Agropyron brachyphyllum Boiss. & Hausskn. ex Boiss.	Poaceae	Irano-Turanian	Za	Hemicryptophyte	YES
92	Agropyron bulbosum Boiss.	Poaceae	Irano-Turanian	Al	Hemicryptophyte	YES
93	Agropyron gentryi Melderis	Poaceae	Irano-Turanian	Za	Hemicryptophyte	YES
94	Agrostis gariana Taheri	Poaceae	Irano-Turanian	Za	Hemicryptophyte	YES
95	Ajuga chamaecistus Ging. ex Benth.	Lamiaceae	Irano-Turanian	Al, Za, Ke, Az	Chamaephyte	NO
96	Ajuga saxicola Assadi & Jamzad	Lamiaceae	Saharo-Sindian		Chamaephyte	YES
97	Albraunia foveopilosa Speta	Scrophulariaceae	Saharo-Sindian		Therophyte	NO
98	Alcea arbelensis Boiss. & Hausskn.	Malvaceae	Irano-Turanian	Al, Az	Hemicryptophyte	NO
99	Alcea assadii Pakravan	Malvaceae	Irano-Turanian	Az	Hemicryptophyte	YES
100	Alcea flavovirens (Boiss. & Buhse) Iljin	Malvaceae	Irano-Turanian	Az, Za	Hemicryptophyte	NO
101	Alcea ghahremanii Pakravan & Assadi	Malvaceae	Irano-Turanian	Az	Hemicryptophyte	YES
102	Alcea glabrata Alef.	Malvaceae	Irano-Turanian	Al, Az, Za	Hemicryptophyte	NO
103	Alcea gorganica (Rech.f., Aellen & Esfand.) Zohary	Malvaceae	Euro-Siberian	Al	Hemicryptophyte	NO
104	Alcea ilamica Pakravan	Malvaceae	Saharo-Sindian		Hemicryptophyte	YES
105	Alcea iranshahrii Pakravan, Ghahr. & Assadi	Malvaceae	Irano-Turanian	Za	Hemicryptophyte	YES
106	Alcea koelzii Riedl	Malvaceae	Irano-Turanian	Az, Za	Hemicryptophyte	NO
107	Alcea kurdica Alef.	Malvaceae	Irano-Turanian	Az, Za	Hemicryptophyte	NO
108	Alcea lenkoranica Iljin	Malvaceae	Euro-Siberian	Al, Lo	Hemicryptophyte	YES
109	Alcea loftusii (Baker f.) Zohary	Malvaceae	Saharo-Sindian		Hemicryptophyte	YES
110	Alcea mazandaranica Pakravan & Ghahr.	Malvaceae	Euro-Siberian	Al	Hemicryptophyte	YES
111	Alcea mozaffarianii Ghahr., Pakravan & Assadi	Malvaceae	Irano-Turanian	Az	Hemicryptophyte	YES
112	Alcea persarum Bornm.	Malvaceae	Irano-Turanian	Za, Ko, Ke	Hemicryptophyte	YES
113	Alcea schirazana Alef.	Malvaceae	Irano-Turanian	Za	Hemicryptophyte	NO
114	Alcea semnanica Pakravan	Malvaceae	Irano-Turanian	Al	Hemicryptophyte	YES
115	Alcea tarica Pakravan & Ghahr.	Malvaceae	Irano-Turanian	Al, Za	Hemicryptophyte	NO

116	Alcea teheranica Parsa	Malvaceae	Irano-Turanian	Al, Za	Hemicryptophyte	YES
117	Alcea wilhelminae Riedl	Malvaceae	Irano-Turanian	Az, Lo	Hemicryptophyte	NO
118	Alchemilla amardica Rothm.	Rosaceae	Euro-Siberian	Al	Hemicryptophyte	YES
119	Alchemilla citrina S.E.Fröhner	Rosaceae	Euro-Siberian	Al, Lo	Hemicryptophyte	NO
120	Alchemilla condensa S.E.Fröhner	Rosaceae	Euro-Siberian	Al, Lo	Hemicryptophyte	YES
121	Alchemilla farinosa S.E.Fröhner	Rosaceae	Euro-Siberian	Al, Az	Hemicryptophyte	YES
122	Alchemilla fluminea S.E.Fröhner	Rosaceae	Irano-Turanian, Euro-Siberian	Al, Az	Hemicryptophyte	YES
123	Alchemilla gigantodus S.E.Fröhner	Rosaceae	Euro-Siberian	Al	Hemicryptophyte	YES
124	Alchemilla hessii Rothm.	Rosaceae	Irano-Turanian, Euro-Siberian	Al, Az	Hemicryptophyte	YES
125	Alchemilla kurdica Rothm.	Rosaceae	Irano-Turanian	Za	Hemicryptophyte	YES
126	Alchemilla melancholica S.E.Fröhner	Rosaceae	Euro-Siberian	Al	Hemicryptophyte	NO
127	Alchemilla microscopica S.E.Fröhner	Rosaceae	Euro-Siberian	Al, Lo	Hemicryptophyte	YES
128	Alchemilla pectiniloba S.E.Fröhner	Rosaceae	Euro-Siberian	Al, Lo	Hemicryptophyte	YES
129	Alchemilla plicatissima S.E.Fröhner	Rosaceae	Euro-Siberian	Al, Lo	Hemicryptophyte	YES
130	Alchemilla rechingeri Rothm.	Rosaceae	Euro-Siberian	Al	Hemicryptophyte	YES
131	Alchemilla surculosa S.E.Fröhner	Rosaceae	Euro-Siberian	Al	Hemicryptophyte	YES
132	Alkanna bracteosa Boiss.	Boraginaceae	Irano-Turanian	Al, Za, Az	Hemicryptophyte	NO
133	Alkanna frigida Boiss.	Boraginaceae	Irano-Turanian	Al, Za	Hemicryptophyte	NO
134	Allium abbasii R.M.Fritsch	Alliaceae	Irano-Turanian	Za	Geophyte	YES
135	Allium akaka S.G.Gmel. ex Schult. & Schult.f.	Alliaceae	Irano-Turanian	Az	Geophyte	NO
136	Allium aladaghense Memariani & Joharchi	Alliaceae	Irano-Turanian	Ko	Geophyte	YES
137	Allium alamutense Razyfard, Zarre & R.M.Fritsch	Alliaceae	Irano-Turanian	Al	Geophyte	YES
138	Allium altissimum Regel	Alliaceae	Irano-Turanian	Ko	Geophyte	YES
139	Allium assadii Seisums	Alliaceae	Irano-Turanian	Za	Geophyte	YES
140	Allium austroiranicum R.M.Fritsch	Alliaceae	Irano-Turanian	Za, Ke	Geophyte	NO
141	Allium autumniflorum F.O.Khass. & Akhani	Alliaceae	Irano-Turanian	Al	Geophyte	YES
142	Allium aznavense R.M.Fritsch	Alliaceae	Irano-Turanian	Az	Geophyte	YES
143	Allium bakhtiaricum Regel	Alliaceae	Irano-Turanian	Za, Az	Geophyte	NO
144	Allium bisotunense R.M.Fritsch	Alliaceae	Irano-Turanian	Za	Geophyte	NO
145	Allium brachyodon Boiss.	Alliaceae	Irano-Turanian	Za	Geophyte	YES

146	Allium brachyscapum Vved.	Alliaceae	Irano-Turanian	Ko	Geophyte	YES
147	Allium breviscapum Stapf	Alliaceae	Irano-Turanian	Az, Za	Geophyte	NO
148	Allium bungei Boiss.	Alliaceae	Irano-Turanian	Al, Za. Ke	Geophyte	YES
149	Allium capitellatum Boiss.	Alliaceae	Irano-Turanian	Al, Za	Geophyte	NO
150	Allium cardiostemon Fisch. & C.A.Mey.	Alliaceae	Irano-Turanian	Az	Geophyte	NO
151	Allium caspium (Pall.) M.Bieb.	Alliaceae	Irano-Turanian	Ko, Al, Lo	Geophyte	NO
152	Allium cathodicarpum Wendelbo	Alliaceae	Irano-Turanian	Ke	Geophyte	NO
153	Allium chloroneurum Boiss.	Alliaceae	Irano-Turanian	Ke	Geophyte	YES
154	Allium chlorotepalum R.M.Fritsch & M.Jaeger	Alliaceae	Irano-Turanian	Za	Geophyte	YES
155	Allium chrysantherum Boiss. & Reut.	Alliaceae	Irano-Turanian	Za	Geophyte	NO
156	Allium clivorum R.M.Fritsch	Alliaceae	Irano-Turanian	Al	Geophyte	YES
157	Allium cristophii Trautv.	Alliaceae	Irano-Turanian	Al, Ko	Geophyte	NO
158	Allium derderianum Regel	Alliaceae	Irano-Turanian	Al	Geophyte	NO
159	Allium dolichovaginatum R.M.Fritsch	Alliaceae	Irano-Turanian	Ko	Geophyte	YES
160	Allium egorovae M.V.Agab. & Ogan.	Alliaceae	Irano-Turanian	Az	Geophyte	NO
161	Allium elburzense Wendelbo	Alliaceae	Irano-Turanian	Al	Geophyte	NO
162	Allium ellisii Hook.f.	Alliaceae	Irano-Turanian	Ko	Geophyte	NO
163	Allium esfahanicum R.M.Fritsch	Alliaceae	Irano-Turanian	Za	Geophyte	NO
164	Allium esfandiarii Matin	Alliaceae	Irano-Turanian	Az	Geophyte	YES
165	Allium fedtschenkoi Nábelek	Alliaceae	Irano-Turanian	Za, Az	Geophyte	NO
166	Allium grande Lipsky	Alliaceae	Irano-Turanian	Al	Geophyte	NO
167	Allium graveolens (R.M.Fritsch) R.M.Fritsch	Alliaceae	Irano-Turanian	Za	Geophyte	NO
168	Allium haemanthoides Boiss. & Reut. ex Regel	Alliaceae	Irano-Turanian	Za	Geophyte	NO
169	Allium hamedanense R.M.Fritsch	Alliaceae	Irano-Turanian	Za	Geophyte	YES
170	Allium helicophyllum Vved.	Alliaceae	Irano-Turanian	Ko	Geophyte	NO
171	Allium hooshidaryae Mashayekhi, Zarre & R.M.Fritsch	Alliaceae	Irano-Turanian	Za	Geophyte	YES
172	Allium iranshahrii R.M.Fritsch	Alliaceae	Irano-Turanian	Az	Geophyte	YES
173	Allium jaegeri R.M.Fritsch	Alliaceae	Irano-Turanian	Za	Geophyte	YES
174	Allium jesdianum Boiss. & Buhse	Alliaceae	Irano-Turanian	Za, Ke	Geophyte	NO
175	Allium joharchii F.O.Khass. & Memariani	Alliaceae	Irano-Turanian	Ko	Geophyte	YES
176	Allium kazerouni Parsa	Alliaceae	Irano-Turanian	Za	Geophyte	NO

177	Allium keusgenii R.M.Fritsch	Alliaceae	Irano-Turanian	Za	Geophyte	YES
178	Allium kirindicum Bornm.	Alliaceae	Irano-Turanian	Za	Geophyte	YES
179	Allium koelzii (Wendelbo) Perss. & Wendelbo	Alliaceae	Irano-Turanian	Za	Geophyte	NO
180	Allium kotschyi Boiss.	Alliaceae	Irano-Turanian	Za	Geophyte	YES
181	Allium kuhrangense Akhavan, Saeidi & R.M.Fritsch	Alliaceae	Irano-Turanian	Za	Geophyte	YES
182	Allium kuhsorkhense R.M.Fritsch & Joharchi	Alliaceae	Irano-Turanian	Ko	Geophyte	NO
183	Allium kurdistanicum Maroofi & R.M.Fritsch	Alliaceae	Irano-Turanian	Za	Geophyte	YES
184	Allium lalesaricum Freyn & Bornm.	Alliaceae	Irano-Turanian	Za, Ke	Geophyte	YES
185	Allium latifolium Jaub. & Spach	Alliaceae	Irano-Turanian	Az, Za	Geophyte	NO
186	Allium longipapillatum R.M.Fritsch & Matin	Alliaceae	Irano-Turanian	Za	Geophyte	YES
187	Allium longivaginatum Wendelbo	Alliaceae	Irano-Turanian	Za	Geophyte	YES
188	Allium mahneshanense Razyfard, Zarre & R.M.Fritsch	Alliaceae	Irano-Turanian	Az	Geophyte	YES
189	Allium materculae Bordz.	Alliaceae	Irano-Turanian	Az	Geophyte	NO
190	Allium minutiflorum Regel	Alliaceae	Irano-Turanian	Za	Geophyte	NO
191	Allium moderense R.M.Fritsch	Alliaceae	Irano-Turanian	Za	Geophyte	YES
192	Allium monophyllum Vved. ex Czerniak.	Alliaceae	Irano-Turanian	Ko	Geophyte	NO
193	Allium montelburzense R.M.Fritsch, Salmaki & Zarre	Alliaceae	Irano-Turanian	Al	Geophyte	YES
194	Allium mozaffarianii Maroofi & R.M.Fritsch	Alliaceae	Irano-Turanian	Za	Geophyte	YES
195	Allium najafdaricum R.M.Fritsch	Alliaceae	Irano-Turanian	Al	Geophyte	YES
196	Allium orientoiranicum Neshati, Zarre & R.M.Fritsch	Alliaceae	Irano-Turanian	Ko	Geophyte	NO
197	Allium pseudobodeanum R.M.Fritsch & Matin	Alliaceae	Irano-Turanian	Al	Geophyte	NO
198	Allium pseudohollandicum R.M.Fritsch	Alliaceae	Irano-Turanian	Az	Geophyte	NO
199	Allium regelii Trautv.	Alliaceae	Irano-Turanian	Ko	Geophyte	NO
200	Allium remediorum (R.M.Fritsch) R.M.Fritsch	Alliaceae	Irano-Turanian	Za	Geophyte	NO
201	Allium sabalense R.M.Fritsch	Alliaceae	Irano-Turanian	Az	Geophyte	YES
202	Allium sahandicum R.M.Fritsch	Alliaceae	Irano-Turanian	Az	Geophyte	NO
203	Allium sanandajense Maroofi & R.M.Fritsch	Alliaceae	Irano-Turanian	Az, Za	Geophyte	YES
204	Allium saralicum R.M.Fritsch	Alliaceae	Irano-Turanian	Al	Geophyte	NO
205	Allium scotostemon Wendelbo	Alliaceae	Irano-Turanian	Za	Geophyte	NO
206	Allium shatakiense Rech.f.	Alliaceae	Irano-Turanian	Za	Geophyte	YES
207	Allium shelkovnikovii Grossh.	Alliaceae	Irano-Turanian	Az	Geophyte	NO

208	Allium straussii Bornm.	Alliaceae	Irano-Turanian	Za	Geophyte	YES
209	Allium subakaka Razyfard & Zarre	Alliaceae	Irano-Turanian	Az	Geophyte	NO
210	Allium subvineale Wendelbo	Alliaceae	Irano-Turanian	Al	Geophyte	YES
211	Allium subnotabile Wendelbo	Alliaceae	Irano-Turanian	Za	Geophyte	YES
212	Allium tuchalense F.O.Khass. & Noroozi	Alliaceae	Irano-Turanian	Al	Geophyte	YES
213	Allium ubipetrense R.M.Fritsch	Alliaceae	Irano-Turanian	Za	Geophyte	NO
214	Allium vescum Wendelbo	Alliaceae	Irano-Turanian		Geophyte	YES
215	Allium wendelboi Matin	Alliaceae	Irano-Turanian		Geophyte	YES
216	Allium zagricum R.M.Fritsch	Alliaceae	Irano-Turanian	Za	Geophyte	NO
217	Allochrusa lutea Falat. & Mahmoodi	Caryophyllaceae	Irano-Turanian		Hemicryptophyte	YES
218	Allochrusa persica (Boiss.) Boiss.	Caryophyllaceae	Irano-Turanian	Az	Chamaephyte	YES
219	Alnus djavanshirii H.Zare	Betulaceae	Euro-Siberian	Al	Phanerophyte	YES
220	Alnus dolichocarpa H.Zare, Amini & Assadi	Betulaceae	Euro-Siberian		Phanerophyte	YES
221	Alococarpum erianthum (DC.) Riedl & Kuber	Apiaceae	Irano-Turanian	Al, Az	Hemicryptophyte	NO
222	Alrawia bellii (Baker) Perss. & Wendelbo	Asparagaceae	Irano-Turanian	Za, Ke	Geophyte	NO
223	Alyssum bracteatum Boiss. & Bushe	Brassicaceae	Irano-Turanian	Al, ze, Az	Hemicryptophyte	NO
224	Alyssum hezarmasjedense Kavousi & Nazary	Brassicaceae	Irano-Turanian	Ko	Chamaephyte	YES
225	Alyssum lanigerum DC.	Brassicaceae	Irano-Turanian	Al, Az, Za	Chamaephyte	NO
226	Alyssum mozaffarianii Kavousi	Brassicaceae	Irano-Turanian	Al	Chamaephyte	YES
227	Alyssum muelleri Boiss. & Bushe	Brassicaceae	Irano-Turanian	Al, Za, Ke	Hemicryptophyte	YES
228	Alyssum persicum Boiss.	Brassicaceae	Irano-Turanian	Za	Chamaephyte	YES
229	Alyssum polycladum Rech.f.	Brassicaceae	Irano-Turanian	Za, Al, Za	Chamaephyte	NO
230	Alyssum stipitatum Kavousi & T.R.Dudley	Brassicaceae	Irano-Turanian	Al	Chamaephyte	YES
231	Alyssum turgidum Dudley	Brassicaceae	Irano-Turanian	Za	Hemicryptophyte	YES
232	Amberboa lippii (L.) DC.	Asteraceae	Saharo-Sindian		Therophyte	YES
233	Amberboa maroofii Negaresh	Asteraceae	Irano-Turanian	Za	Therophyte	YES
234	Amberboa sosnovskyi Iljin	Asteraceae	Irano-Turanian	Az	Therophyte	YES
235	Amberboa zanjanica Ranjbar & Negaresh	Asteraceae	Irano-Turanian		Therophyte	YES
236	Amygdalus eburnea Spach	Rosaceae	Irano-Turanian, Saharo-Sindian	Za, Ke, Lo	Phanerophyte	NO
237	Amygdalus elaeagnifolia Spach	Rosaceae	Irano-Turanian	Za, Ke	Phanerophyte	NO

238	Amygdalus glauca Browicz	Rosaceae	Irano-Turanian	Za	Phanerophyte	YES
239	Amygdalus haussknechtii (C.K.Schneid.) Bornm.	Rosaceae	Irano-Turanian	Za	Phanerophyte	NO
240	Amygdalus kurdistanica Attar, Maroofi & Vafadar	Rosaceae	Irano-Turanian	Za	Phanerophyte	YES
241	Amygdalus orazii Attar, Maroofi & Vafadar	Rosaceae	Irano-Turanian	Za	Phanerophyte	YES
242	Amygdalus paboti Browicz	Rosaceae	Irano-Turanian	Za	Phanerophyte	YES
243	Amygdalus reticulata Runemark ex Khat.	Rosaceae	Irano-Turanian	Za	Phanerophyte	YES
244	Amygdalus wendelboi Freitag	Rosaceae	Irano-Turanian	Ke, Lo	Phanerophyte	YES
245	Anabasis calcarea (Charif & Aellen) Bokhari & Wendelbo	Chenopodiaceae	Irano-Turanian		Chamaephyte	NO
246	Anabasis firouzii Akhani	Chenopodiaceae	Irano-Turanian	Ko	Hemicryptophyte	YES
247	Anabasis haussknechtii Bunge ex Boiss.	Chenopodiaceae	Irano-Turanian		Chamaephyte	NO
248	Anchonium elichrysifolium (DC.) Boiss.	Brassicaceae	Irano-Turanian	Al, Az, Lo	Hemicryptophyte	NO
249	Andrachne fruticulosa Boiss.	Euphorbiaceae	Irano-Turanian	Za, Ke, Lo	Chamaephyte	NO
250	Angelica urumiensis Mozaff.	Apiaceae	Irano-Turanian	Al	Hemicryptophyte	YES
251	Anthemis atropatana Iranshahr	Asteraceae	Irano-Turanian	Az	Therophyte	NO
252	Anthemis austroiranica Rech.f., Aellen & Esfand.	Asteraceae	Irano-Turanian, Saharo-Sindian		Therophyte	NO
253	Anthemis brachystephana Bornm. & Gauba	Asteraceae	Irano-Turanian		Therophyte	NO
254	Anthemis bushehrica Iranshahr	Asteraceae	Saharo-Sindian		Therophyte	YES
255	Anthemis fungosa Boiss. & Hausskn.	Asteraceae	Saharo-Sindian		Therophyte	YES
256	Anthemis gayana Boiss.	Asteraceae	Irano-Turanian	Za, Ke	Therophyte	NO
257	Anthemis gilanica Bornm. & Gauba	Asteraceae	Irano-Turanian	Al, Za	Therophyte	NO
258	Anthemis gillettii Iranshahr	Asteraceae	Saharo-Sindian		Therophyte	YES
259	Anthemis gracilis Iranshahr	Asteraceae	Irano-Turanian	Za	Therophyte	YES
260	Anthemis hemistephana Boiss.	Asteraceae	Irano-Turanian		Therophyte	YES
261	Anthemis leptophylla Eig	Asteraceae	Saharo-Sindian		Therophyte	YES
262	Anthemis lorestanica Iranshahr	Asteraceae	Irano-Turanian, Saharo-Sindian	Za	Therophyte	NO
263	Anthemis mazandaranica Iranshahr	Asteraceae	Euro-Siberian	Al	Therophyte	YES
264	Anthemis mirheydari Iranshahr	Asteraceae	Saharo-Sindian		Therophyte	YES
265	Anthemis moghanica Iranshahr	Asteraceae	Irano-Turanian	Az	Therophyte	NO
266	Anthemis persica Boiss.	Asteraceae	Saharo-Sindian		Therophyte	NO
267	Anthemis schizostephana Boiss. & Hausskn.	Asteraceae	Irano-Turanian	Za	Therophyte	NO

268	Anthemis susiana Nábělek	Asteraceae	Saharo-Sindian		Therophyte	NO
269	Anthemis talyschensis Fed.	Asteraceae	Irano-Turanian	Az	Hemicryptophyte	YES
270	Anthochlamys multinervis Rech.f.	Chenopodiaceae	Irano-Turanian		Therophyte	NO
271	Aphanopleura breviseta (Boiss.) Heywood & Jury	Apiaceae	Irano-Turanian		Therophyte	NO
272	Arabis ottonis-schulzii Bornm. & Gauba	Brassicaceae	Irano-Turanian	Al	Hemicryptophyte	YES
273	Arabis rimarum Rech.f.	Brassicaceae	Irano-Turanian	Al	Hemicryptophyte	YES
274	Arenaria assadii Fadaie	Caryophyllaceae	Irano-Turanian	Az	Hemicryptophyte	YES
275	Arenaria bulica Stapf ex F.N.Williams	Caryophyllaceae	Irano-Turanian	Za, Ke	Chamaephyte	YES
276	Arenaria kandavanensis Fadaie, Sheidai & Assadi	Caryophyllaceae	Euro-Siberian	Al	Hemicryptophyte	YES
277	Arenaria longibracteata Fadaie	Caryophyllaceae	Irano-Turanian	Az	Hemicryptophyte	YES
278	Arenaria minutissima Rech.f. & Esfand.	Caryophyllaceae	Irano-Turanian	Za, Ke	Chamaephyte	YES
279	Arenaria persica Boiss.	Caryophyllaceae	Irano-Turanian	Za, Ke	Chamaephyte	NO
280	Arenaria polycnemifolia Boiss.	Caryophyllaceae	Irano-Turanian	Al, Za	Chamaephyte	NO
281	Arenaria semiromica Fadaie	Caryophyllaceae	Irano-Turanian	Za	Hemicryptophyte	YES
282	Arenaria szowitsii Boiss.	Caryophyllaceae	Irano-Turanian	Az	Chamaephyte	NO
283	Arenaria tetrasticha Boiss.	Caryophyllaceae	Irano-Turanian	Za	Chamaephyte	YES
284	Arenaria zargariana Parsa	Caryophyllaceae	Irano-Turanian	Al, Za	Chamaephyte	NO
285	Argyrolobium trigonelloides Jaub. & Spach	Fabaceae	Irano-Turanian	Al, Za, Ke, Az	Hemicryptophyte	NO
286	Aristolochia hyrcana P.H.Davis & M.S.Khan	Aristolochiaceae	Irano-Turanian, Euro-Siberian	Al	Geophyte	YES
287	Aristolochia olivieri Colleg. ex Boiss.	Aristolochiaceae	Irano-Turanian	Al, Za	Hemicryptophyte	NO
288	Arnebia violascens Riedl	Boraginaceae	Saharo-Sindian		Therophyte	YES
289	Artemisia gypsacea Krasch., Popov & Lincz. ex Poljakov	Asteraceae	Irano-Turanian	Ko	Hemicryptophyte	YES
290	Artemisia kermanensis Podlech	Asteraceae	Irano-Turanian	Ke, Lo	Chamaephyte	NO
291	Artemisia khorassanica Podlech	Asteraceae	Irano-Turanian	Al, Ko	Hemicryptophyte	NO
292	Artemisia kulbadica Boiss. & Buhse	Asteraceae	Euro-Siberian		Hemicryptophyte	YES
293	Artemisia melanolepis Boiss.	Asteraceae	Irano-Turanian	Al, Az	Chamaephyte	NO
294	Artemisia quettensis Podlech	Asteraceae	Irano-Turanian	Ko	Hemicryptophyte	YES
295	Artemisia splendens Willd.	Asteraceae	Irano-Turanian	Al, Az	Hemicryptophyte	NO
296	Arum giganteum Ghahr.	Araceae	Irano-Turanian	Za	Geophyte	NO
297	Asparagus khorasanensis Hamdi & Assadi	Asparagaceae	Irano-Turanian	Ko	Chamaephyte	YES

298	Asparagus touranensis Hamdi & Assadi	Asparagaceae	Irano-Turanian, Saharo-Sindian		Chamaephyte	NO
299	Asperula fragillima Boiss. & Hausskn.	Rubiaceae	Irano-Turanian	Za	Hemicryptophyte	NO
300	Asperula gorganica Schönb.-Tem. & Ehrend.	Rubiaceae	Euro-Siberian	Al	Chamaephyte	YES
301	Asperula mazanderanica Ehrend.	Rubiaceae	Euro-Siberian	Al	Chamaephyte	NO
302	Asperula microphylla Boiss.	Rubiaceae	Euro-Siberian	Al	Hemicryptophyte	NO
303	Asperula oppositifolia Regel & Schmalh.	Rubiaceae	Irano-Turanian	Ko	Hemicryptophyte	YES
304	Asperula rechingeri Ehrend. & Schönb.-Tem.	Rubiaceae	Irano-Turanian	Za	Chamaephyte	NO
305	Asperula rezaiyensis Schönb.-Tem.	Rubiaceae	Irano-Turanian	Az	Chamaephyte	YES
306	Asperula seticornis Boiss.	Rubiaceae	Irano-Turanian	Za	Therophyte	YES
307	Asperula sherardioides Jaub. & Spach	Rubiaceae	Irano-Turanian		Therophyte	YES
308	Aster bachtiaricus Mozaff.	Asteraceae	Irano-Turanian	Za	Hemicryptophyte	YES
309	Astragalus abadehensis Maassoumi & Podlech	Fabaceae	Irano-Turanian	Za	Hemicryptophyte	YES
310	Astragalus abharensis Maassoumi & Podlech	Fabaceae	Irano-Turanian	Za	Hemicryptophyte	YES
311	Astragalus abnormalis Rech.f.	Fabaceae	Irano-Turanian	Za	Hemicryptophyte	NO
312	Astragalus absconditus Zarre & Podlech	Fabaceae	Irano-Turanian	Za	Hemicryptophyte	YES
313	Astragalus absentivus Maassoumi	Fabaceae	Irano-Turanian	Al	Hemicryptophyte	YES
314	Astragalus acutifolius Bunge	Fabaceae	Irano-Turanian		Chamaephyte	YES
315	Astragalus adulterinus Podlech	Fabaceae	Irano-Turanian	Za	Hemicryptophyte	YES
316	Astragalus aestimabilis Podlech	Fabaceae	Irano-Turanian	Za	Chamaephyte	YES
317	Astragalus aestivorum Podlech	Fabaceae	Irano-Turanian	Al	Hemicryptophyte	YES
318	Astragalus affinis Podlech & Zarre	Fabaceae	Irano-Turanian	Ko	Hemicryptophyte	YES
319	Astragalus ahangarensis Zarre & Podlech	Fabaceae	Irano-Turanian		Hemicryptophyte	YES
320	Astragalus aharicus Maassoumi & Podlech	Fabaceae	Irano-Turanian	Az	Hemicryptophyte	NO
321	Astragalus ahmad-parsae Maassoumi	Fabaceae	Irano-Turanian	Za	Chamaephyte	YES
322	Astragalus ahmed-adlii Bornm. & Gauba	Fabaceae	Irano-Turanian		Chamaephyte	YES
323	Astragalus akhanii Podlech	Fabaceae	Saharo-Sindian		Hemicryptophyte	YES
324	Astragalus alamouticus Maassoumi	Fabaceae	Irano-Turanian	Al	Hemicryptophyte	YES
325	Astragalus alavaanus Podlech	Fabaceae	Irano-Turanian		Hemicryptophyte	YES
326	Astragalus albispinus Širj. & Bornm.	Fabaceae	Irano-Turanian	Za, Ke	Chamaephyte	NO
327	Astragalus alienus Podlech	Fabaceae	Irano-Turanian	Za	Hemicryptophyte	YES

328	Astragalus aliomranii Maassoumi	Fabaceae	Irano-Turanian	Az	Hemicryptophyte	YES
329	Astragalus altimontanus Podlech & Maassoumi	Fabaceae	Irano-Turanian	Al	Hemicryptophyte	YES
330	Astragalus altiusculus Maassoumi & Ghahrem.	Fabaceae	Irano-Turanian	Al	Chamaephyte	YES
331	Astragalus alyssiformis Maassoumi, Ghahrem. & Javadi	Fabaceae	Irano-Turanian	Al	Hemicryptophyte	YES
332	Astragalus ammodendroides Bornm.	Fabaceae	Irano-Turanian		Chamaephyte	YES
333	Astragalus anacamptus Bunge	Fabaceae	Irano-Turanian	Al	Hemicryptophyte	YES
334	Astragalus andabaddensis Maassoumi, Bagheri & F.Ghahrem.	Fabaceae	Irano-Turanian	Az	Hemicryptophyte	YES
335	Astragalus andabilensis Ranjbar & Mahmoudian	Fabaceae	Irano-Turanian	Az	Hemicryptophyte	YES
336	Astragalus anguranensis Podlech & Maassoumi	Fabaceae	Irano-Turanian	Az	Chamaephyte	YES
337	Astragalus angustistipulatus Podlech	Fabaceae	Irano-Turanian	Ke	Hemicryptophyte	YES
338	Astragalus anodiophilus Zarre & Podlech	Fabaceae	Irano-Turanian	Za	Hemicryptophyte	YES
339	Astragalus anserinifolius Boiss.	Fabaceae	Irano-Turanian, Saharo-Sindian	Za, Ke, Bl	Hemicryptophyte	NO
340	Astragalus arasbaranensis Maassoumi & Ranjbar	Fabaceae	Irano-Turanian	Az	Hemicryptophyte	YES
341	Astragalus archibaldii Podlech	Fabaceae	Irano-Turanian	Za	Hemicryptophyte	YES
342	Astragalus ardahalicus Parsa	Fabaceae	Irano-Turanian	Za	Chamaephyte	NO
343	Astragalus argyrostachys Boiss.	Fabaceae	Irano-Turanian	Za	Chamaephyte	NO
344	Astragalus ashtianensis Podlech & Maassoumi	Fabaceae	Irano-Turanian	Za	Hemicryptophyte	YES
345	Astragalus askius Bunge	Fabaceae	Irano-Turanian	Al	Hemicryptophyte	NO
346	Astragalus aspadanus Bunge	Fabaceae	Irano-Turanian	Za	Hemicryptophyte	NO
347	Astragalus aspreticola Podlech	Fabaceae	Irano-Turanian	Ko	Hemicryptophyte	YES
348	Astragalus assadabadensis Ghahrem.-Nejad & Podlech	Fabaceae	Irano-Turanian	Za	Hemicryptophyte	YES
349	Astragalus assadii Maassoumi & Podlech	Fabaceae	Irano-Turanian	Ko	Hemicryptophyte	YES
350	Astragalus atricapillus Bornm.	Fabaceae	Irano-Turanian	Al	Hemicryptophyte	NO
351	Astragalus attarae Podlech	Fabaceae	Irano-Turanian	Ko	Hemicryptophyte	YES
352	Astragalus austrokhorasanicus Podlech	Fabaceae	Irano-Turanian	Ko	Chamaephyte	YES
353	Astragalus austromahneshanensis F.Ghahrem., Maassoumi & Bagheri	Fabaceae	Irano-Turanian	Az	Chamaephyte	YES
354	Astragalus avajensis Podlech	Fabaceae	Irano-Turanian	Za	Hemicryptophyte	YES
355	Astragalus avicennicus Parsa	Fabaceae	Irano-Turanian	Za	Hemicryptophyte	NO
356	Astragalus azizii Maassoumi	Fabaceae	Irano-Turanian	Al	Hemicryptophyte	YES
357	Astragalus aznaicus Podlech & Maassoumi	Fabaceae	Irano-Turanian	Za	Hemicryptophyte	YES

358	Astragalus baba-alliar Parsa	Fabaceae	Saharo-Sindian		Chamaephyte	NO
359	Astragalus babakhanloui Maassoumi & Podlech	Fabaceae	Irano-Turanian	Za	Hemicryptophyte	NO
360	Astragalus badelehensis Maassoumi & Taheri	Fabaceae	Irano-Turanian	Al	Hemicryptophyte	YES
361	Astragalus baftensis Ranjbar & Maassoumi	Fabaceae	Irano-Turanian	Ke	Hemicryptophyte	YES
362	Astragalus baharensis Ghahrem.	Fabaceae	Irano-Turanian	Ko	Chamaephyte	YES
363	Astragalus bajgiranensis Podlech	Fabaceae	Irano-Turanian	Ko	Hemicryptophyte	YES
364	Astragalus baraftabensis Maassoumi & Podlech	Fabaceae	Irano-Turanian	Za	Hemicryptophyte	YES
365	Astragalus barnasariformis Maassoumi, F.Ghahrem. & Bagheri	Fabaceae	Irano-Turanian		Hemicryptophyte	YES
366	Astragalus bashmaghensis Maassoumi & Podlech	Fabaceae	Irano-Turanian	Al	Chamaephyte	YES
367	Astragalus bashmensis Maassoumi	Fabaceae	Irano-Turanian	Al	Hemicryptophyte	YES
368	Astragalus basilicus Podlech & Maassoumi	Fabaceae	Irano-Turanian	Az	Hemicryptophyte	NO
369	Astragalus bavanatensis Maassoumi, Nowroozi & Podlech	Fabaceae	Irano-Turanian	Za	Hemicryptophyte	YES
370	Astragalus bavanaticus Maassoumi, Nowroozi & Podlech	Fabaceae	Irano-Turanian	Za	Hemicryptophyte	YES
371	Astragalus bazarganii Podlech & Zarre	Fabaceae	Irano-Turanian	Ko, Lo	Chamaephyte	NO
372	Astragalus bazmanicus Podlech	Fabaceae	Irano-Turanian		Chamaephyte	YES
373	Astragalus beckii Bornm.	Fabaceae	Irano-Turanian	Al, Az	Hemicryptophyte	NO
374	Astragalus bejourensis Podlech & Maassoumi	Fabaceae	Irano-Turanian	Az	Hemicryptophyte	YES
375	Astragalus belgheisicoides Podlech & Maassoumi	Fabaceae	Irano-Turanian	Az	Hemicryptophyte	YES
376	Astragalus belgheisicus Maassoumi	Fabaceae	Irano-Turanian	Al, Za	Hemicryptophyte	NO
377	Astragalus biarjmandicus Podlech & Zarre	Fabaceae	Irano-Turanian		Chamaephyte	YES
378	Astragalus bijarensis Podlech & Sytin	Fabaceae	Irano-Turanian	Az	Hemicryptophyte	YES
379	Astragalus birangiae Maassoumi	Fabaceae	Irano-Turanian	Az	Hemicryptophyte	YES
380	Astragalus biserrula Bunge	Fabaceae	Irano-Turanian	Az, Za, Al, Ko	Hemicryptophyte	NO
381	Astragalus bodeanus Fisch.	Fabaceae	Irano-Turanian	Za	Chamaephyte	NO
382	Astragalus bojnurdensis Podlech	Fabaceae	Irano-Turanian	Ko	Hemicryptophyte	YES
383	Astragalus bordschensis Bornm.	Fabaceae	Irano-Turanian	Za	Hemicryptophyte	YES
384	Astragalus borujenensis Ranjbar & Maassoumi	Fabaceae	Irano-Turanian	Za	Chamaephyte	YES
385	Astragalus botryophorus Maassoumi & Podlech	Fabaceae	Irano-Turanian	Za	Chamaephyte	YES
386	Astragalus bounophilus Boiss.	Fabaceae	Irano-Turanian	Al	Chamaephyte	YES
387	Astragalus bozghoushensis Maassoumi, Mozaff. & Ramezani	Fabaceae	Irano-Turanian	Az	Hemicryptophyte	YES
388	Astragalus brachyodontus Boiss.	Fabaceae	Irano-Turanian	Az, Za, Al	Hemicryptophyte	NO

389	Astragalus bradosticus Maassoumi & Podlech	Fabaceae	Irano-Turanian	Az	Chamaephyte	YES
390	Astragalus brevicalycinus Maassoumi	Fabaceae	Saharo-Sindian		Phanerophyte	YES
391	Astragalus brevipedunculatus Ranjbar	Fabaceae	Irano-Turanian	Az	Hemicryptophyte	YES
392	Astragalus brevirhachis Tietz & Zarre	Fabaceae	Irano-Turanian	Ke	Chamaephyte	YES
393	Astragalus brevitomentosus Podlech	Fabaceae	Irano-Turanian	Za	Hemicryptophyte	YES
394	Astragalus brunsianus Bornm.	Fabaceae	Irano-Turanian	Al	Chamaephyte	YES
395	Astragalus bukanensis Maassoumi & Podlech	Fabaceae	Irano-Turanian	Az	Hemicryptophyte	YES
396	Astragalus caespititius Podlech	Fabaceae	Irano-Turanian	Ke	Hemicryptophyte	YES
397	Astragalus callainus Podlech	Fabaceae	Irano-Turanian	Ko	Hemicryptophyte	NO
398	Astragalus calliphysa Bunge	Fabaceae	Irano-Turanian	Za, Ke	Chamaephyte	NO
399	Astragalus campylanthoides Bornm.	Fabaceae	Irano-Turanian	Za, Az	Chamaephyte	YES
400	Astragalus campylanthus Boiss.	Fabaceae	Irano-Turanian	Za, Ke	Chamaephyte	NO
401	Astragalus canosus Maassoumi	Fabaceae	Irano-Turanian	Az	Hemicryptophyte	YES
402	Astragalus capax Maassoumi	Fabaceae	Irano-Turanian	Al	Chamaephyte	NO
403	Astragalus capito Boiss.	Fabaceae	Irano-Turanian	Al	Hemicryptophyte	YES
404	Astragalus carmanicus Bornm.	Fabaceae	Irano-Turanian	Ke	Chamaephyte	NO
405	Astragalus catacamptus Bunge	Fabaceae	Irano-Turanian	Al, Ko, Za	Hemicryptophyte	NO
406	Astragalus cemerinus Beck	Fabaceae	Irano-Turanian	Za	Chamaephyte	NO
407	Astragalus cephalanthus DC.	Fabaceae	Irano-Turanian	Za, Ke	Chamaephyte	NO
408	Astragalus chaetopodus Bunge	Fabaceae	Irano-Turanian	Za	Hemicryptophyte	YES
409	Astragalus chahartaghensis Maassoumi & Podlech	Fabaceae	Irano-Turanian	Za	Hemicryptophyte	NO
410	Astragalus chalaranthus Boiss. & Hausskn.	Fabaceae	Irano-Turanian	Za	Chamaephyte	YES
411	Astragalus chaldoranicus Podlech & Maassoumi	Fabaceae	Irano-Turanian	Az	Hemicryptophyte	YES
412	Astragalus chartostegius Boiss. & Hausskn.	Fabaceae	Irano-Turanian	Za	Chamaephyte	NO
413	Astragalus chehregani Zarre & Podlech	Fabaceae	Irano-Turanian	Az	Chamaephyte	YES
414	Astragalus chichesticus Podlech & Maassoumi	Fabaceae	Irano-Turanian	Az	Hemicryptophyte	YES
415	Astragalus chrysanthus Boiss. & Hohen.	Fabaceae	Irano-Turanian	Al	Hemicryptophyte	NO
416	Astragalus chrysotrichus Boiss.	Fabaceae	Irano-Turanian	Za	Hemicryptophyte	NO
417	Astragalus ciceropsis Hamzehee & Maassoumi	Fabaceae	Irano-Turanian	Za	Hemicryptophyte	YES
418	Astragalus circumlacustris Podlech & Sytin	Fabaceae	Irano-Turanian	Az	Hemicryptophyte	YES
419	Astragalus clivicola Podlech & Maassoumi	Fabaceae	Irano-Turanian	Za	Hemicryptophyte	YES

420	Astragalus coluteopsis Parsa	Fabaceae	Irano-Turanian	Za	Chamaephyte	YES
421	Astragalus confertiformis Širj. & Rech.f.	Fabaceae	Irano-Turanian	Za, Ke	Hemicryptophyte	NO
422	Astragalus confusus Bunge	Fabaceae	Irano-Turanian	Al	Hemicryptophyte	NO
423	Astragalus controversus Maassoumi & Podlech	Fabaceae	Irano-Turanian	Ko	Hemicryptophyte	YES
424	Astragalus craccinopsis Maassoumi	Fabaceae	Irano-Turanian	Za	Hemicryptophyte	YES
425	Astragalus crispus Ghahrem.	Fabaceae	Irano-Turanian		Hemicryptophyte	YES
426	Astragalus culminatus Maassoumi, Kaz.Osaloo & Joharchi	Fabaceae	Irano-Turanian	Ko	Hemicryptophyte	YES
427	Astragalus curviflorus Boiss.	Fabaceae	Irano-Turanian	Za	Chamaephyte	NO
428	Astragalus cyclophyllon Beck	Fabaceae	Irano-Turanian	Az, Za	Hemicryptophyte	NO
429	Astragalus cystosus Zarre & Podlech	Fabaceae	Irano-Turanian	Ko	Chamaephyte	YES
430	Astragalus daenensis Boiss.	Fabaceae	Irano-Turanian	Za, Ke	Hemicryptophyte	NO
431	Astragalus daghdaghabadensis Maassoumi	Fabaceae	Irano-Turanian	Az, Za	Hemicryptophyte	NO
432	Astragalus damghanensis Podlech	Fabaceae	Irano-Turanian	Al	Hemicryptophyte	YES
433	Astragalus darlingtonii Podlech	Fabaceae	Irano-Turanian	Za	Hemicryptophyte	NO
434	Astragalus darrehbidensis Podlech & Zarre	Fabaceae	Irano-Turanian	Ke	Hemicryptophyte	YES
435	Astragalus delutulus Maassoumi	Fabaceae	Irano-Turanian	Za	Hemicryptophyte	NO
436	Astragalus demavendicola Bornm. & Gauba	Fabaceae	Irano-Turanian	Za, Al	Chamaephyte	NO
437	Astragalus demavendicus Boiss. & Buhse	Fabaceae	Irano-Turanian	Al	Hemicryptophyte	NO
438	Astragalus demonstratus Maassoumi	Fabaceae	Irano-Turanian	Az	Chamaephyte	YES
439	Astragalus dengolanensis Podlech	Fabaceae	Irano-Turanian	Za	Hemicryptophyte	YES
440	Astragalus denticulatus Podlech	Fabaceae	Irano-Turanian	Ko	Hemicryptophyte	YES
441	Astragalus dianat-nejadii Ghahrem.	Fabaceae	Irano-Turanian	Az	Chamaephyte	YES
442	Astragalus dictyolobus Bunge	Fabaceae	Irano-Turanian	Az, Al	Hemicryptophyte	NO
443	Astragalus distans Fisch.	Fabaceae	Irano-Turanian	Za	Chamaephyte	YES
444	Astragalus divandarrehensis Podlech	Fabaceae	Irano-Turanian	Za	Hemicryptophyte	NO
445	Astragalus diversipilosus Podlech	Fabaceae	Irano-Turanian	Za	Hemicryptophyte	YES
446	Astragalus diversus Podlech & Maassoumi	Fabaceae	Irano-Turanian	Az	Hemicryptophyte	YES
447	Astragalus doghrunensis Maassoumi & Podlech	Fabaceae	Irano-Turanian	Az	Chamaephyte	YES
448	Astragalus dopolanicus Podlech	Fabaceae	Irano-Turanian	Za	Hemicryptophyte	YES
449	Astragalus dorudensis Zarre & Podlech	Fabaceae	Irano-Turanian	Za	Hemicryptophyte	YES
450	Astragalus doshman-ziariensis Maassoumi & Podlech	Fabaceae	Irano-Turanian	Za	Hemicryptophyte	YES

451	Astragalus dschuparensis Freyn & Bornm.	Fabaceae	Irano-Turanian	Ke	Chamaephyte	NO
452	Astragalus durandianus Aitch. & Baker	Fabaceae	Irano-Turanian	Al, Ko, Lo	Chamaephyte	NO
453	Astragalus dysbatophilus Zarre & Podlech	Fabaceae	Irano-Turanian	Az	Hemicryptophyte	YES
454	Astragalus ebenoides Boiss.	Fabaceae	Irano-Turanian	Az, Za	Chamaephyte	NO
455	Astragalus ebrahimabadensis Zarre & Podlech	Fabaceae	Irano-Turanian	Za	Hemicryptophyte	YES
456	Astragalus eburneus Bornm. & Gauba	Fabaceae	Saharo-Sindian		Hemicryptophyte	NO
457	Astragalus ecbatanus Bunge	Fabaceae	Irano-Turanian	Za	Chamaephyte	NO
458	Astragalus echidna Bunge	Fabaceae	Irano-Turanian	Ko, Lo	Chamaephyte	NO
459	Astragalus edmondsonii Podlech	Fabaceae	Irano-Turanian	Ko	Hemicryptophyte	YES
460	Astragalus elezgensis Maassoumi & Kaz.Osaloo	Fabaceae	Irano-Turanian	Za	Hemicryptophyte	NO
461	Astragalus elwendicus Bornm.	Fabaceae	Irano-Turanian	Za	Hemicryptophyte	NO
462	Astragalus erinifolius Pau	Fabaceae	Irano-Turanian	Za	Chamaephyte	YES
463	Astragalus eriopodus Boiss.	Fabaceae	Irano-Turanian	Az, Za, Al	Hemicryptophyte	NO
464	Astragalus eriostomus Bornm.	Fabaceae	Irano-Turanian	Za	Chamaephyte	NO
465	Astragalus erubescens Podlech	Fabaceae	Irano-Turanian	Ko	Hemicryptophyte	NO
466	Astragalus erwinii-gaubae Širj. & Rech.f.	Fabaceae	Irano-Turanian	Al	Chamaephyte	NO
467	Astragalus erythrolepis Boiss.	Fabaceae	Irano-Turanian	Za	Chamaephyte	YES
468	Astragalus esferayenicus Podlech & Maassoumi	Fabaceae	Irano-Turanian	Ko	Hemicryptophyte	YES
469	Astragalus estahbanensis Maassoumi & Podlech	Fabaceae	Irano-Turanian	Za	Hemicryptophyte	YES
470	Astragalus eusarathron I.Deml & Podlech	Fabaceae	Irano-Turanian	Za	Hemicryptophyte	YES
471	Astragalus evanensis Maassoumi & Podlech	Fabaceae	Irano-Turanian	Al	Hemicryptophyte	YES
472	Astragalus expetitus Maassoumi	Fabaceae	Irano-Turanian	Az, Za	Chamaephyte	NO
473	Astragalus expectatus Maassoumi	Fabaceae	Irano-Turanian	Za	Hemicryptophyte	YES
474	Astragalus facetus Maassoumi & Podlech	Fabaceae	Irano-Turanian	Za	Hemicryptophyte	YES
475	Astragalus fagh-soleimanensis Maassoumi & Podlech	Fabaceae	Irano-Turanian	Az, Za	Hemicryptophyte	NO
476	Astragalus farsicus Širj. & Rech.f.	Fabaceae	Irano-Turanian, Saharo-Sindian	Ke	Chamaephyte	NO
477	Astragalus fasciculifolius Boiss.	Fabaceae	Irano-Turanian	Za	Chamaephyte	NO
478	Astragalus fausicola Podlech ex Bagheri, Massoumi & F.Ghahrem.	Fabaceae	Irano-Turanian	Za	Hemicryptophyte	YES
479	Astragalus ferruminatus Maassoumi	Fabaceae	Irano-Turanian	Za	Chamaephyte	YES
480	Astragalus filifoliolatus Maassoumi	Fabaceae	Irano-Turanian	Za	Chamaephyte	YES

481	Astragalus firuzkuhensis Podlech	Fabaceae	Irano-Turanian	Al	Hemicryptophyte	YES
482	Astragalus fischeri Buhse	Fabaceae	Irano-Turanian	Za	Chamaephyte	NO
483	Astragalus flexilipes Bornm.	Fabaceae	Irano-Turanian	Za	Chamaephyte	YES
484	Astragalus floccosus Boiss	Fabaceae	Irano-Turanian	Za, Al, Ko, Ke	Chamaephyte	NO
485	Astragalus foliosus Podlech, Maassoumi & Ranjbar	Fabaceae	Irano-Turanian	Az	Hemicryptophyte	YES
486	Astragalus fortuitus Maassoumi	Fabaceae	Irano-Turanian	Za	Hemicryptophyte	YES
487	Astragalus fragiferus Bunge	Fabaceae	Irano-Turanian	Za, Ke	Chamaephyte	NO
488	Astragalus fridae Rech.f.	Fabaceae	Irano-Turanian	Al	Hemicryptophyte	YES
489	Astragalus fuliginosus Beck	Fabaceae	Irano-Turanian	Az, Al	Hemicryptophyte	NO
490	Astragalus gagnieui Maassoumi & Podlech	Fabaceae	Irano-Turanian	Za	Hemicryptophyte	YES
491	Astragalus gamasiabensis Maassoumi, Zarre & Podlech	Fabaceae	Irano-Turanian	Za	Chamaephyte	YES
492	Astragalus gandomanicus Podlech	Fabaceae	Irano-Turanian	Za	Hemicryptophyte	YES
493	Astragalus garmashubensis Maassoumi & Khorrami	Fabaceae	Irano-Turanian	Za	Hemicryptophyte	YES
494	Astragalus gaubae Bornm.	Fabaceae	Irano-Turanian, Saharo-Sindian	Za	Hemicryptophyte	YES
495	Astragalus ghahremanii Maassoumi & Podlech	Fabaceae	Saharo-Sindian		Hemicryptophyte	NO
496	Astragalus ghamishluensis Dastpak, Maassoumi & Kaz.Osaloo	Fabaceae	Irano-Turanian	Za	Chamaephyte	YES
497	Astragalus ghashghaicus Tietz & Zarre	Fabaceae	Irano-Turanian	Za	Chamaephyte	NO
498	Astragalus ghouchanensis Souzani, Zarre & Maassoumi	Fabaceae	Irano-Turanian	Ko	Chamaephyte	YES
499	Astragalus gifanicus Maassoumi & Podlech	Fabaceae	Irano-Turanian	Ko	Hemicryptophyte	YES
500	Astragalus gigantifoliolatus Maassoumi & Maroofi	Fabaceae	Irano-Turanian	Za	Hemicryptophyte	YES
501	Astragalus gigantirostratus Maassoumi, Ghahr., Ghahrem. & F.Matin.	Fabaceae	Irano-Turanian	Al	Chamaephyte	YES
502	Astragalus gilvanensis Ranjbar & Nouri	Fabaceae	Irano-Turanian		Hemicryptophyte	YES
503	Astragalus glaucacanthos Fisch.	Fabaceae	Irano-Turanian	Za, Al, Ke	Chamaephyte	NO
504	Astragalus glaucops Bornm.	Fabaceae	Irano-Turanian	Za	Chamaephyte	NO
505	Astragalus glaucopsiformis Maassoumi	Fabaceae	Irano-Turanian	Za	Chamaephyte	YES
506	Astragalus glochidiatus Maassoumi	Fabaceae	Irano-Turanian	Za	Hemicryptophyte	YES
507	Astragalus glumaceus Boiss.	Fabaceae	Irano-Turanian	Az, Za	Chamaephyte	NO
508	Astragalus griseus Boiss.	Fabaceae	Irano-Turanian	Za, Ke, Lo	Hemicryptophyte	NO
509	Astragalus gueldenstaedtiae Bunge	Fabaceae	Irano-Turanian	Al	Hemicryptophyte	YES
510	Astragalus gululsaranii Podlech	Fabaceae	Irano-Turanian	Ko	Hemicryptophyte	YES

511	Astragalus gypsaceus Beck	Fabaceae	Irano-Turanian	Az, Za	Hemicryptophyte	NO
512	Astragalus gypsocola Maassoumi & Mozaff.	Fabaceae	Saharo-Sindian		Hemicryptophyte	YES
513	Astragalus hajiabadensis Podlech & Maassoumi	Fabaceae	Irano-Turanian		Hemicryptophyte	YES
514	Astragalus hajijafanensis Maassoumi	Fabaceae	Irano-Turanian	Az	Hemicryptophyte	YES
515	Astragalus hamadanus Boiss.	Fabaceae	Irano-Turanian	Al, Za	Hemicryptophyte	NO
516	Astragalus hamadryadis Podlech	Fabaceae	Irano-Turanian	Al	Hemicryptophyte	YES
517	Astragalus harazensis Zarre & Podlech	Fabaceae	Irano-Turanian	Al, Ko	Hemicryptophyte	YES
518	Astragalus hecatae Zarre & Podlech	Fabaceae	Irano-Turanian	Za	Hemicryptophyte	YES
519	Astragalus heinzianus Maassoumi & Mozaff.	Fabaceae	Saharo-Sindian		Hemicryptophyte	YES
520	Astragalus hekmat-Safaviae Ghahrem.	Fabaceae	Irano-Turanian	Ko	Chamaephyte	YES
521	Astragalus herbertii Maassoumi	Fabaceae	Irano-Turanian	Al	Hemicryptophyte	YES
522	Astragalus hermannii Freitag & Podlech	Fabaceae	Irano-Turanian	Al, Ko	Hemicryptophyte	YES
523	Astragalus heterodoxus Bunge	Fabaceae	Irano-Turanian	Ke	Hemicryptophyte	NO
524	Astragalus heterophyllus Podlech	Fabaceae	Irano-Turanian	Za	Hemicryptophyte	NO
525	Astragalus hirticalyx Boiss.	Fabaceae	Irano-Turanian	Az	Chamaephyte	NO
526	Astragalus hirtus Bunge	Fabaceae	Irano-Turanian	Za	Hemicryptophyte	YES
527	Astragalus holopsilus Bunge	Fabaceae	Irano-Turanian	Za	Hemicryptophyte	NO
528	Astragalus holosemius Bunge	Fabaceae	Irano-Turanian	Az	Hemicryptophyte	NO
529	Astragalus homandicus Maassoumi & Podlech	Fabaceae	Irano-Turanian	Al	Hemicryptophyte	YES
530	Astragalus horridus Boiss.	Fabaceae	Irano-Turanian	Za, Ke	Chamaephyte	NO
531	Astragalus hotkanensis Maassoumi & Mirtadz.	Fabaceae	Irano-Turanian	Ke	Chamaephyte	YES
532	Astragalus huthianus Freyn & Bornm.	Fabaceae	Irano-Turanian	Ke	Hemicryptophyte	NO
533	Astragalus hymenocalyx Boiss.	Fabaceae	Irano-Turanian	Za	Hemicryptophyte	NO
534	Astragalus hymenostegis Fisch. & C.A. Mey.	Fabaceae	Irano-Turanian	Az	Chamaephyte	NO
535	Astragalus hypsogeton Bunge	Fabaceae	Irano-Turanian	Ko	Chamaephyte	YES
536	Astragalus ibicinus Boiss. & Hausskn.	Fabaceae	Irano-Turanian	Za	Hemicryptophyte	NO
537	Astragalus icmadophilus Hand.-Mazz.	Fabaceae	Irano-Turanian	Az, Al	Chamaephyte	YES
538	Astragalus ignotus Podlech	Fabaceae	Irano-Turanian	Az	Hemicryptophyte	YES
539	Astragalus ilachchiensis Ranjbar & Zarin	Fabaceae	Irano-Turanian	Az	Hemicryptophyte	YES
540	Astragalus imbecillus Maassoumi & Podlech	Fabaceae	Irano-Turanian	Ke	Hemicryptophyte	YES
541	Astragalus impexus Podlech	Fabaceae	Irano-Turanian	Za, Ke	Hemicryptophyte	NO

542	Astragalus inchebroonensis Maassoumi	Fabaceae	Euro-Siberian		Chamaephyte	YES
543	Astragalus indistinctus Podlech & Maassoumi	Fabaceae	Irano-Turanian	Za	Hemicryptophyte	NO
544	Astragalus inexpectatus Maassoumi & Podlech	Fabaceae	Irano-Turanian	Za	Hemicryptophyte	YES
545	Astragalus inexpectatus Maassoumi & Podlech	Fabaceae	Irano-Turanian	Za	Hemicryptophyte	NO
546	Astragalus innotabilis Podlech	Fabaceae	Irano-Turanian	Az	Hemicryptophyte	YES
547	Astragalus inquilinus Maassoumi	Fabaceae	Irano-Turanian	Za	Hemicryptophyte	YES
548	Astragalus insularis-ashkii Maassoumi & Podlech	Fabaceae	Irano-Turanian	Za	Hemicryptophyte	YES
549	Astragalus iodotropis Boiss. & Hohen.	Fabaceae	Irano-Turanian	Al	Chamaephyte	NO
550	Astragalus iranicus Bunge	Fabaceae	Irano-Turanian	Az, Za, Al, Ko	Hemicryptophyte	NO
551	Astragalus iranshahrii Maassoumi & Podlech	Fabaceae	Irano-Turanian	Za	Hemicryptophyte	NO
552	Astragalus ischredensis Bunge	Fabaceae	Irano-Turanian	Za	Hemicryptophyte	NO
553	Astragalus issatissensis Maassoumi & Mahmoodi	Fabaceae	Irano-Turanian	Ke	Hemicryptophyte	YES
554	Astragalus jacobsii Podlech	Fabaceae	Irano-Turanian	Az, Za	Hemicryptophyte	NO
555	Astragalus jamzadiae Maassoumi	Fabaceae	Irano-Turanian	Za	Chamaephyte	YES
556	Astragalus jaskensis Maassoumi	Fabaceae	Saharo-Sindian		Phanerophyte	YES
557	Astragalus jesdianus Boiss. & Buhse	Fabaceae	Irano-Turanian	Ke, Lo	Chamaephyte	NO
558	Astragalus jessenii Bunge	Fabaceae	Irano-Turanian	Al	Hemicryptophyte	NO
559	Astragalus johannis Boiss.	Fabaceae	Irano-Turanian	Za, Ke	Hemicryptophyte	NO
560	Astragalus joharchii Ghahrem.-Nejad & Gaskin	Fabaceae	Irano-Turanian	Ko	Chamaephyte	NO
561	Astragalus juladakensis Maassoumi	Fabaceae	Irano-Turanian	Al	Chamaephyte	YES
562	Astragalus kabutarlanensis Dehshiri & Maassoumi	Fabaceae	Irano-Turanian	Za	Hemicryptophyte	YES
563	Astragalus kadschoroides Ranjbar	Fabaceae	Irano-Turanian	Az	Chamaephyte	YES
564	Astragalus kalatehensis Maassoumi & Kaz.Osaloo	Fabaceae	Irano-Turanian	Ko	Hemicryptophyte	YES
565	Astragalus karl-heinzii Maassoumi	Fabaceae	Irano-Turanian	Az	Chamaephyte	YES
566	Astragalus kashafensis Podlech	Fabaceae	Irano-Turanian	Ko	Hemicryptophyte	YES
567	Astragalus kashmarensis Maassoumi & Podlech	Fabaceae	Irano-Turanian	Ko	Hemicryptophyte	NO
568	Astragalus kaswinensis Bornm.	Fabaceae	Irano-Turanian	Al	Hemicryptophyte	YES
569	Astragalus kavirensis Freitag	Fabaceae	Irano-Turanian		Phanerophyte	YES
570	Astragalus kentrophyllus Podlech	Fabaceae	Saharo-Sindian		Chamaephyte	NO
571	Astragalus keredjensis (Bornm. & Gauba) Podl.	Fabaceae	Irano-Turanian	Al	Hemicryptophyte	NO
572	Astragalus kermanschahensis Bornm.	Fabaceae	Irano-Turanian	Za	Hemicryptophyte	YES

573	Astragalus khadem-kandicus Maassoumi & Podlech	Fabaceae	Irano-Turanian	Az	Hemicryptophyte	YES
574	Astragalus khajehensis Ghahrem.-Nejad	Fabaceae	Irano-Turanian	Az	Hemicryptophyte	YES
575	Astragalus khajiboulaghensis Maassoumi	Fabaceae	Irano-Turanian	Al	Hemicryptophyte	YES
576	Astragalus khaneradarensis Sirj. & Rech.f.	Fabaceae	Saharo-Sindian		Chamaephyte	YES
577	Astragalus kharvanensis Ranjbar	Fabaceae	Irano-Turanian	Az	Chamaephyte	YES
578	Astragalus khatamsaziae Maassoumi	Fabaceae	Irano-Turanian	Az	Chamaephyte	YES
579	Astragalus khongensis Maassoumi, Joharchi & Podlech	Fabaceae	Irano-Turanian		Hemicryptophyte	YES
580	Astragalus khonikensis Nasseh & Joharchi	Fabaceae	Irano-Turanian		Chamaephyte	YES
581	Astragalus khoshjailensis Sirj. & Rech.f.	Fabaceae	Irano-Turanian	Al, Ko	Hemicryptophyte	NO
582	Astragalus khosrowabadensis Ranjbar & Karamian	Fabaceae	Irano-Turanian	Za	Hemicryptophyte	YES
583	Astragalus khunsarensis Zarre & Podlech	Fabaceae	Irano-Turanian	Za	Hemicryptophyte	YES
584	Astragalus kiamaky-daghensis Maassoumi & Podlech	Fabaceae	Irano-Turanian	Az	Hemicryptophyte	YES
585	Astragalus kiviensis Ranjbar & Rahimin.	Fabaceae	Irano-Turanian	Az	Chamaephyte	YES
586	Astragalus koelzii Barneby	Fabaceae	Irano-Turanian	Za	Hemicryptophyte	YES
587	Astragalus kohrudicus Bunge	Fabaceae	Irano-Turanian	Az, Al, Za	Chamaephyte	NO
588	Astragalus kordloricus Zarre	Fabaceae	Irano-Turanian	Za	Chamaephyte	NO
589	Astragalus kuhidashtehensis Podlech	Fabaceae	Irano-Turanian	Al	Hemicryptophyte	YES
590	Astragalus lacus-valashti Maassoumi, Podlech & Jalili	Fabaceae	Irano-Turanian	Al	Hemicryptophyte	YES
591	Astragalus lalesarensis Bornm.	Fabaceae	Irano-Turanian	Ke	Chamaephyte	YES
592	Astragalus lambinonii Podlech	Fabaceae	Irano-Turanian	Za	Hemicryptophyte	NO
593	Astragalus laricus Boiss. & Hohen.	Fabaceae	Irano-Turanian	Al	Therophyte	YES
594	Astragalus laristanicus Bornm. & Gauba	Fabaceae	Irano-Turanian, Saharo-Sindian		Hemicryptophyte	NO
595	Astragalus lasiocalycinus Podlech & Maassoumi	Fabaceae	Irano-Turanian	Az	Hemicryptophyte	YES
596	Astragalus lateritiiformis Zarre, Maassoumi & Podlech	Fabaceae	Irano-Turanian	Za	Chamaephyte	NO
597	Astragalus lateritius Boiss. & Hausskn. ex Boiss.	Fabaceae	Irano-Turanian	Za	Chamaephyte	NO
598	Astragalus latianicus Maassoumi & Ranjbar	Fabaceae	Irano-Turanian	Al	Hemicryptophyte	YES
599	Astragalus ledinghamii Barneby	Fabaceae	Irano-Turanian	Za, Ke	Hemicryptophyte	NO
600	Astragalus leonardii Maassoumi	Fabaceae	Irano-Turanian	Za	Hemicryptophyte	YES
601	Astragalus lepidus Podlech	Fabaceae	Irano-Turanian	Za	Hemicryptophyte	YES
602	Astragalus leptynticus Maassoumi	Fabaceae	Irano-Turanian	Al	Chamaephyte	NO

603	Astragalus leucocerciformis Ranjbar & Maassoumi	Fabaceae	Irano-Turanian	Za	Chamaephyte	YES
604	Astragalus leucophanus Bornm.	Fabaceae	Irano-Turanian	Za	Hemicryptophyte	YES
605	Astragalus leucoptilus Boiss. & Hausskn. ex Boiss.	Fabaceae	Irano-Turanian	Za	Chamaephyte	YES
606	Astragalus lilacinus Boiss.	Fabaceae	Irano-Turanian	Az, Al	Hemicryptophyte	NO
607	Astragalus longicuspis Bunge	Fabaceae	Irano-Turanian	Az	Hemicryptophyte	NO
608	Astragalus longirostratus Pau	Fabaceae	Irano-Turanian	Za	Hemicryptophyte	NO
609	Astragalus longistylus Bunge	Fabaceae	Irano-Turanian	Al, Za, Ke, Bl	Chamaephyte	NO
610	Astragalus lurorum Bornm.	Fabaceae	Irano-Turanian	Za	Chamaephyte	NO
611	Astragalus lycioides Boiss.	Fabaceae	Irano-Turanian	Al, Ke, Ko	Chamaephyte	NO
612	Astragalus maabudii Ranjbar	Fabaceae	Irano-Turanian	Za	Hemicryptophyte	YES
613	Astragalus maarofii Podlech & Maassoumi	Fabaceae	Irano-Turanian	Az	Hemicryptophyte	YES
614	Astragalus maassoumii Podlech	Fabaceae	Irano-Turanian	Za	Hemicryptophyte	NO
615	Astragalus macrosemius Boiss. & Hohen.	Fabaceae	Irano-Turanian	Al	Chamaephyte	YES
616	Astragalus magistratus Maassoumi, Ghahr. & Mozaff.	Fabaceae	Irano-Turanian	Al	Chamaephyte	NO
617	Astragalus magnibracteatus Maassoumi & Maroofi	Fabaceae	Irano-Turanian	Za	Chamaephyte	YES
618	Astragalus maharluensis Bornm. & Gauba	Fabaceae	Irano-Turanian	Za	Therophyte	YES
619	Astragalus mahneshanensis Maassoumi & Moussavi	Fabaceae	Irano-Turanian	Az	Hemicryptophyte	YES
620	Astragalus makuensis Maassoumi, Bagheri & Rahimin.	Fabaceae	Irano-Turanian	Az	Chamaephyte	YES
621	Astragalus managettae Sirj. & Rech.f.	Fabaceae	Irano-Turanian	Za	Hemicryptophyte	NO
622	Astragalus marandicus Podlech	Fabaceae	Irano-Turanian	Za	Hemicryptophyte	YES
623	Astragalus margonensis Ranjbar, Rahimin. & Raufi	Fabaceae	Irano-Turanian	Za	Hemicryptophyte	YES
624	Astragalus markasicus Podlech & Maassoumi	Fabaceae	Irano-Turanian	Az	Hemicryptophyte	YES
625	Astragalus masulehensis Ranjbar & Assadi	Fabaceae	Euro-Siberian		Hemicryptophyte	YES
626	Astragalus megalocystis Bunge	Fabaceae	Irano-Turanian	Al	Chamaephyte	NO
627	Astragalus mehranensis Maassoumi & Mozaff.	Fabaceae	Saharo-Sindian		Hemicryptophyte	YES
628	Astragalus mehrizianus Podlech & Maassoumi	Fabaceae	Irano-Turanian	Ke	Hemicryptophyte	YES
629	Astragalus meimandicus Maassoumi & Vakili	Fabaceae	Irano-Turanian	Ke	Chamaephyte	YES
630	Astragalus melanocalyx Boiss. & Buhse	Fabaceae	Irano-Turanian	Za, Ke	Hemicryptophyte	YES
631	Astragalus melanodon Boiss.	Fabaceae	Irano-Turanian	Za, Ke	Hemicryptophyte	NO
632	Astragalus membranostipulus Maassoumi	Fabaceae	Irano-Turanian	Al	Chamaephyte	YES
633	Astragalus memnonius Maassoumi & Podlech	Fabaceae	Irano-Turanian	Za	Hemicryptophyte	YES

634	Astragalus memoriosus Pakravan, Nasseh & Maassoumi	Fabaceae	Irano-Turanian	Az	Phanerophyte	YES
635	Astragalus meshkinensis Podlech	Fabaceae	Irano-Turanian	Az	Hemicryptophyte	YES
636	Astragalus microphysa Boiss.	Fabaceae	Irano-Turanian	Az	Chamaephyte	NO
637	Astragalus minutulus Maassoumi	Fabaceae	Irano-Turanian	Ko	Hemicryptophyte	YES
638	Astragalus mishodaghmontanus Ranjbar, Karamian & Nouri	Fabaceae	Irano-Turanian	Az	Hemicryptophyte	YES
639	Astragalus modestus Boiss. & Hohen.	Fabaceae	Irano-Turanian	Al	Hemicryptophyte	NO
640	Astragalus monanthemus Boiss.	Fabaceae	Irano-Turanian	Al	Hemicryptophyte	YES
641	Astragalus monozyx Bornm.	Fabaceae	Irano-Turanian	Za	Hemicryptophyte	YES
642	Astragalus montis-bakhtiari Maassoumi & Sardari	Fabaceae	Irano-Turanian	Za	Hemicryptophyte	YES
643	Astragalus montismishoudaghi Sheikh Akbari Mehr, Ghorbani & Maassoumi	Fabaceae	Irano-Turanian	Az	Hemicryptophyte	YES
644	Astragalus montis-nacarouzii Maassoumi & Maroofi	Fabaceae	Irano-Turanian	Za	Chamaephyte	YES
645	Astragalus montis-parrowii Maassoumi & Nemati	Fabaceae	Irano-Turanian	Za	Hemicryptophyte	YES
646	Astragalus montis-varvashti Podlech	Fabaceae	Irano-Turanian	Al	Hemicryptophyte	YES
647	Astragalus montosus Maassoumi	Fabaceae	Irano-Turanian	Za	Chamaephyte	YES
648	Astragalus moussavii Maassoumi, Ghahr.-Nejad & Ghahr.	Fabaceae	Irano-Turanian	Al	Hemicryptophyte	YES
649	Astragalus mozaffarianii Maassoumi	Fabaceae	Irano-Turanian	Az	Hemicryptophyte	YES
650	Astragalus mucronifolius Boiss.	Fabaceae	Irano-Turanian	Za, Al, Ke	Chamaephyte	NO
651	Astragalus murinus Boiss.	Fabaceae	Irano-Turanian	Za	Chamaephyte	NO
652	Astragalus musaianus Maassoumi & Joharchi	Fabaceae	Irano-Turanian		Hemicryptophyte	YES
653	Astragalus mutus Podlech	Fabaceae	Irano-Turanian	Az	Hemicryptophyte	YES
654	Astragalus myriacanthus Boiss.	Fabaceae	Irano-Turanian	Za, Ke, Bl	Chamaephyte	NO
655	Astragalus naftabensis Sirj. & Rech.f.	Fabaceae	Irano-Turanian	Al	Chamaephyte	YES
656	Astragalus nahavandicus Maassoumi	Fabaceae	Irano-Turanian	Za	Hemicryptophyte	NO
657	Astragalus nalbandanicus Podlech	Fabaceae	Irano-Turanian		Hemicryptophyte	YES
658	Astragalus neoassadianus Ranjbar	Fabaceae	Irano-Turanian	Ko	Hemicryptophyte	YES
659	Astragalus neochaldoranicus Podlech & Maassoumi	Fabaceae	Irano-Turanian	Az	Hemicryptophyte	NO
660	Astragalus neoiranshahrii Maassoumi & Amini Rad	Fabaceae	Irano-Turanian	Az	Hemicryptophyte	YES
661	Astragalus neomaassoumianus Ranjbar	Fabaceae	Irano-Turanian	Za	Hemicryptophyte	YES
662	Astragalus neomobayenii Maassoumi	Fabaceae	Irano-Turanian	Az	Hemicryptophyte	YES
663	Astragalus neomozaffarianii Maassoumi	Fabaceae	Irano-Turanian	Az	Chamaephyte	YES

664	Astragalus neopodlechii Maassoumi	Fabaceae	Irano-Turanian	Za	Hemicryptophyte	YES
665	Astragalus neosytinii Ranjbar	Fabaceae	Irano-Turanian	Ke	Hemicryptophyte	YES
666	Astragalus neyshaburensis Podlech	Fabaceae	Irano-Turanian	Ko	Chamaephyte	YES
667	Astragalus nezva-montis Podlech & Zarre	Fabaceae	Irano-Turanian	Al	Hemicryptophyte	YES
668	Astragalus nigrohirsutus (Tietz & Zarre) Borjian	Fabaceae	Irano-Turanian	Za	Chamaephyte	YES
669	Astragalus nowroozii Podlech & Zarre	Fabaceae	Irano-Turanian	Ke	Chamaephyte	YES
670	Astragalus nubicola Podlech	Fabaceae	Irano-Turanian	Al	Hemicryptophyte	YES
671	Astragalus nurabadensis Maassoumi & Podlech	Fabaceae	Irano-Turanian	Za	Hemicryptophyte	YES
672	Astragalus nurensis Boiss. & Buhse	Fabaceae	Irano-Turanian, Euro-Siberian	Al	Hemicryptophyte	NO
673	Astragalus ochreatus Bunge	Fabaceae	Irano-Turanian	Al, Ko	Hemicryptophyte	NO
674	Astragalus ochrochlorus Boiss. & Hohen.	Fabaceae	Irano-Turanian	Al	Chamaephyte	NO
675	Astragalus olangensis Maassoumi & Joharchi	Fabaceae	Irano-Turanian	Al	Hemicryptophyte	YES
676	Astragalus oligoflorus Maassoumi, Ghahrem. & Javadi	Fabaceae	Irano-Turanian	Az	Hemicryptophyte	YES
677	Astragalus orientopersicus F.Ghahrem., Joharchi, Fereid. & Hoseini	Fabaceae	Irano-Turanian	Ko	Chamaephyte	YES
678	Astragalus orthocarpoides Sirj. & Rech.f.	Fabaceae	Irano-Turanian	Ko	Chamaephyte	YES
679	Astragalus ovigerus Boiss.	Fabaceae	Irano-Turanian	Za	Chamaephyte	YES
680	Astragalus ovoideus Sirj. & Rech.f.	Fabaceae	Irano-Turanian, Saharo-Sindian	Za, Ke, Lo	Chamaephyte	NO
681	Astragalus pakravaniae Podlech & Maassoumi	Fabaceae	Irano-Turanian	Al	Hemicryptophyte	YES
682	Astragalus paralurges Bunge	Fabaceae	Irano-Turanian	Az, Za	Chamaephyte	NO
683	Astragalus parvarensis Podlech & Sytin	Fabaceae	Irano-Turanian	Al	Hemicryptophyte	YES
684	Astragalus parvulus Bornm.	Fabaceae	Irano-Turanian	Az	Hemicryptophyte	YES
685	Astragalus passargadensis Maassoumi	Fabaceae	Irano-Turanian	Za	Chamaephyte	YES
686	Astragalus patrius Maassoumi	Fabaceae	Irano-Turanian	Az, Za, Al	Hemicryptophyte	NO
687	Astragalus paucifoliolatus Podlech	Fabaceae	Irano-Turanian	Za	Hemicryptophyte	NO
688	Astragalus pauperiflorus Bornm.	Fabaceae	Irano-Turanian	Az	Hemicryptophyte	NO
689	Astragalus pauxillis Maassoumi & Ghahrem.	Fabaceae	Irano-Turanian	Az	Chamaephyte	NO
690	Astragalus pediculariformis Maassoumi	Fabaceae	Irano-Turanian	Az	Chamaephyte	YES
691	Astragalus pellitus Bunge	Fabaceae	Irano-Turanian	Al, Ko, Ke	Hemicryptophyte	NO
692	Astragalus pendulipodus Ranjbar & Karamian	Fabaceae	Irano-Turanian	Az	Chamaephyte	YES
693	Astragalus penetratus Maassoumi	Fabaceae	Irano-Turanian	Za	Hemicryptophyte	YES

694	Astragalus pentanthus Boiss.	Fabaceae	Irano-Turanian	Za	Hemicryptophyte	NO
695	Astragalus perdurans Podlech	Fabaceae	Irano-Turanian	Al, Ko	Hemicryptophyte	NO
696	Astragalus pereshkhoranicus Maassoumi & F.Ghahrem.	Fabaceae	Irano-Turanian	Az	Chamaephyte	YES
697	Astragalus persicus (DC.) Fisch. & C.A.Mey.	Fabaceae	Irano-Turanian	Za	Chamaephyte	NO
698	Astragalus peymanii Maassoumi	Fabaceae	Euro-Siberian	Za	Chamaephyte	YES
699	Astragalus pileh-khasehensis Podlech & Maassoumi	Fabaceae	Irano-Turanian	Az	Hemicryptophyte	YES
700	Astragalus piranshahricus Maassoumi & Podlech	Fabaceae	Irano-Turanian	Za, Az	Hemicryptophyte	NO
701	Astragalus plagiophacos Maassoumi & Podlech	Fabaceae	Irano-Turanian	Al, Za	Hemicryptophyte	NO
702	Astragalus platyfoliolatus Maassoumi	Fabaceae	Irano-Turanian	Za	Hemicryptophyte	YES
703	Astragalus platysematus Bunge	Fabaceae	Irano-Turanian	Al	Hemicryptophyte	NO
704	Astragalus plebejus Boiss.	Fabaceae	Irano-Turanian	Za	Hemicryptophyte	NO
705	Astragalus podoloboides Maassoumi	Fabaceae	Irano-Turanian		Chamaephyte	YES
706	Astragalus podosphaerus Boiss. & Hausskn.	Fabaceae	Irano-Turanian	Za	Hemicryptophyte	YES
707	Astragalus poliotrichus Bornm.	Fabaceae	Irano-Turanian	Za	Hemicryptophyte	YES
708	Astragalus polyanthus Bunge	Fabaceae	Irano-Turanian	Az	Chamaephyte	YES
709	Astragalus polystachys Maassoumi	Fabaceae	Irano-Turanian	Az	Chamaephyte	YES
710	Astragalus porphyrophysa Bornm. & Gauba	Fabaceae	Irano-Turanian, Saharo-Sindian		Chamaephyte	NO
711	Astragalus pravitzii Podlech	Fabaceae	Irano-Turanian	Za	Hemicryptophyte	NO
712	Astragalus protectus Maassoumi & Podlech	Fabaceae	Irano-Turanian	Za	Hemicryptophyte	YES
713	Astragalus pseudocapito Podlech	Fabaceae	Irano-Turanian	Az	Hemicryptophyte	YES
714	Astragalus pseudocomosus Maassoumi, F.Ghahrem. & Bagheri	Fabaceae	Irano-Turanian	Az	Hemicryptophyte	YES
715	Astragalus pseudocyclophyllus Rech.f.	Fabaceae	Irano-Turanian	Al	Hemicryptophyte	YES
716	Astragalus pseudofragiferus Tietz	Fabaceae	Saharo-Sindian		Chamaephyte	YES
717	Astragalus pseudoibicinus Maassoumi & Podlech	Fabaceae	Irano-Turanian	Za	Hemicryptophyte	NO
718	Astragalus pseudoindurascens Sirj. & Rech.f.	Fabaceae	Irano-Turanian	Ko	Hemicryptophyte	NO
719	Astragalus pseudojohannis Maassoumi & Podlech	Fabaceae	Irano-Turanian	Ke	Hemicryptophyte	YES
720	Astragalus pseudokurrumensis Sirj. & Rech.f.	Fabaceae	Irano-Turanian	Ko	Hemicryptophyte	YES
721	Astragalus pseudomacrostachys Maassoumi	Fabaceae	Irano-Turanian	Az	Hemicryptophyte	YES
722	Astragalus pseudonigrescens Maassoumi	Fabaceae	Irano-Turanian		Chamaephyte	YES
723	Astragalus pseudo-orthocarpus Ranjbar & Maassoumi	Fabaceae	Irano-Turanian	Al	Chamaephyte	YES

724	Astragalus pseudopellitus Podlech	Fabaceae	Irano-Turanian	Za	Hemicryptophyte	YES
725	Astragalus pseudopersicus Podlech & Maassoumi	Fabaceae	Irano-Turanian	Az	Chamaephyte	YES
726	Astragalus pseudorobustus Podlech & Maassoumi	Fabaceae	Irano-Turanian	Za	Hemicryptophyte	NO
727	Astragalus pseudoshebarensis Podlech	Fabaceae	Irano-Turanian	Za, Ke	Chamaephyte	NO
728	Astragalus pseudotortuosus Tietz & Zarre	Fabaceae	Irano-Turanian	Za	Chamaephyte	YES
729	Astragalus pseudozagrosicus Maassoumi & Podlech	Fabaceae	Irano-Turanian	Za	Hemicryptophyte	NO
730	Astragalus psilostylus Bunge	Fabaceae	Irano-Turanian	Az	Chamaephyte	NO
731	Astragalus ptychophyllus Boiss.	Fabaceae	Irano-Turanian	Za	Chamaephyte	NO
732	Astragalus punctatus Bunge	Fabaceae	Irano-Turanian	Az	Hemicryptophyte	NO
733	Astragalus qaratchaicus Maassoumi, Ghahrem. & Javadi	Fabaceae	Irano-Turanian	Az	Hemicryptophyte	YES
734	Astragalus qeydarnabiensis Bagheri, F.Ghahrem. & Maassoumi	Fabaceae	Irano-Turanian	Az	Chamaephyte	YES
735	Astragalus qohestanicus Nasseh & Maassoumi	Fabaceae	Irano-Turanian		Chamaephyte	YES
736	Astragalus qorvehensis Podlech	Fabaceae	Irano-Turanian	Za	Chamaephyte	YES
737	Astragalus qoturensis Podlech	Fabaceae	Irano-Turanian	Az	Chamaephyte	YES
738	Astragalus quinquefoliolatus Bunge	Fabaceae	Irano-Turanian	Za	Hemicryptophyte	NO
739	Astragalus rahiminejadii Ranjbar	Fabaceae	Irano-Turanian	Za	Hemicryptophyte	YES
740	Astragalus ranjbarii Podlech	Fabaceae	Irano-Turanian	Ko	Chamaephyte	YES
741	Astragalus rassulovae Podlech	Fabaceae	Irano-Turanian	Ko, Lo	Hemicryptophyte	YES
742	Astragalus raswendicus Hausskn. & Bornm.	Fabaceae	Irano-Turanian	Za	Chamaephyte	NO
743	Astragalus razensis Nasseh & Joharchi	Fabaceae	Irano-Turanian	Ko	Hemicryptophyte	YES
744	Astragalus recognitus Fisch.	Fabaceae	Irano-Turanian, Euro-Siberian	Az	Chamaephyte	NO
745	Astragalus reconditus Podlech & Maassoumi	Fabaceae	Irano-Turanian	Za	Hemicryptophyte	YES
746	Astragalus recurvatus Podlech	Fabaceae	Irano-Turanian	Za	Hemicryptophyte	YES
747	Astragalus regestus Maassoumi	Fabaceae	Irano-Turanian	Za	Hemicryptophyte	NO
748	Astragalus remotiflorus Boiss.	Fabaceae	Irano-Turanian	Za, Ke	Chamaephyte	NO
749	Astragalus remotijugus Boiss. & Hohen.	Fabaceae	Irano-Turanian	Al	Hemicryptophyte	NO
750	Astragalus remotispicatus Bagheri & Maassoumi	Fabaceae	Irano-Turanian	Az	Chamaephyte	YES
751	Astragalus renzianus Podlech	Fabaceae	Irano-Turanian	Ko	Hemicryptophyte	NO
752	Astragalus repentinus Ekici & Podlech	Fabaceae	Irano-Turanian	Al, Ko	Hemicryptophyte	NO
753	Astragalus reticulato-venosus Maassoumi & Podlech	Fabaceae	Irano-Turanian	Ko	Hemicryptophyte	YES

754	Astragalus reuterianus Boiss.	Fabaceae	Irano-Turanian	Za, Ke	Chamaephyte	NO
755	Astragalus rhabdophorus Bornm.	Fabaceae	Irano-Turanian	Za	Hemicryptophyte	NO
756	Astragalus rhodosemius Boiss. & Hausskn.	Fabaceae	Irano-Turanian	Az, Za, Ke	Chamaephyte	NO
757	Astragalus rimarum Bornm.	Fabaceae	Irano-Turanian	Al	Hemicryptophyte	YES
758	Astragalus riouxii Rech.f.	Fabaceae	Irano-Turanian	Za	Hemicryptophyte	YES
759	Astragalus rollovii Grossh.	Fabaceae	Irano-Turanian	Az	Hemicryptophyte	NO
760	Astragalus rosellus Sirj. & Rech.f.	Fabaceae	Irano-Turanian	Al	Chamaephyte	NO
761	Astragalus rubriflorus Bunge	Fabaceae	Irano-Turanian	Al	Chamaephyte	NO
762	Astragalus rubriphysa Maassoumi & Khorrami	Fabaceae	Irano-Turanian	Za	Chamaephyte	YES
763	Astragalus rubrocalycinus Maassoumi & Podlech	Fabaceae	Irano-Turanian	Az	Hemicryptophyte	NO
764	Astragalus rubrolineatus Sirj. & Rech.f.	Fabaceae	Irano-Turanian	Al	Chamaephyte	YES
765	Astragalus rubrostriatus Bunge	Fabaceae	Irano-Turanian	Az, Za	Chamaephyte	NO
766	Astragalus rudimentus Maassoumi	Fabaceae	Irano-Turanian	Az	Hemicryptophyte	NO
767	Astragalus rufescens Freyn	Fabaceae	Irano-Turanian	Za, Ke	Hemicryptophyte	NO
768	Astragalus runemarkii Maassoumi & Podlech	Fabaceae	Irano-Turanian	Az	Hemicryptophyte	YES
769	Astragalus ruscifolius Boiss.	Fabaceae	Irano-Turanian	Za, Ke, Al	Hemicryptophyte	NO
770	Astragalus saadatabadensis Podlech	Fabaceae	Irano-Turanian	Za	Hemicryptophyte	YES
771	Astragalus sabetii Podlech & Maassoumi	Fabaceae	Irano-Turanian	Az	Hemicryptophyte	YES
772	Astragalus saccatus Boiss.	Fabaceae	Irano-Turanian	Za	Hemicryptophyte	NO
773	Astragalus saetiger Becht	Fabaceae	Irano-Turanian	Za	Hemicryptophyte	YES
774	Astragalus safavii Podlech & Maassoumi	Fabaceae	Irano-Turanian	Az	Hemicryptophyte	NO
775	Astragalus sahendi Buhse	Fabaceae	Irano-Turanian	Az	Chamaephyte	YES
776	Astragalus salavatabadensis Podlech	Fabaceae	Irano-Turanian	Za	Chamaephyte	YES
777	Astragalus salehabadensis Ranjbar & Zarin	Fabaceae	Irano-Turanian		Hemicryptophyte	YES
778	Astragalus sanandajianus Tietz	Fabaceae	Irano-Turanian	Za	Chamaephyte	YES
779	Astragalus sarabensis Maassoumi & Podlech	Fabaceae	Irano-Turanian	Az	Hemicryptophyte	YES
780	Astragalus saremii Maassoumi	Fabaceae	Irano-Turanian	Za	Hemicryptophyte	YES
781	Astragalus sarzehensis Ranjbar	Fabaceae	Irano-Turanian	Ke	Hemicryptophyte	YES
782	Astragalus savanatensis Ranjbar, Vitek & Mahmoudian	Fabaceae	Irano-Turanian	Za	Hemicryptophyte	YES
783	Astragalus savellanicus Podlech	Fabaceae	Irano-Turanian	Az	Hemicryptophyte	NO
784	Astragalus scapiger Ranjbar & Maassoumi	Fabaceae	Irano-Turanian	Az, Ko	Hemicryptophyte	NO

785	Astragalus schmidii Podlech	Fabaceae	Irano-Turanian	Ko	Hemicryptophyte	NO
786	Astragalus sciureus Boiss. & Hohen.	Fabaceae	Irano-Turanian	Al	Chamaephyte	NO
787	Astragalus sclerocladus Bunge	Fabaceae	Irano-Turanian	Za	Chamaephyte	YES
788	Astragalus segregatus Zarre & Podlech	Fabaceae	Irano-Turanian	Za	Hemicryptophyte	YES
789	Astragalus seidabadensis Bunge	Fabaceae	Irano-Turanian	Za	Chamaephyte	NO
790	Astragalus semiglabricarpus Maassoumi	Fabaceae	Irano-Turanian	Ko	Chamaephyte	YES
791	Astragalus semilunatus Podlech	Fabaceae	Irano-Turanian	Az	Hemicryptophyte	YES
792	Astragalus semiromensis Podlech & Maassoumi	Fabaceae	Irano-Turanian	Za	Hemicryptophyte	NO
793	Astragalus semitarius Zarre & Podlech	Fabaceae	Irano-Turanian	Az	Hemicryptophyte	YES
794	Astragalus semnanensis Bornm. & Rech.f.	Fabaceae	Irano-Turanian	Al	Chamaephyte	YES
795	Astragalus senilis Bornm.	Fabaceae	Irano-Turanian	Az, Al	Hemicryptophyte	NO
796	Astragalus shabilensis Podlech & Maassoumi	Fabaceae	Irano-Turanian	Az	Hemicryptophyte	YES
797	Astragalus shahbazanicus Podlech	Fabaceae	Irano-Turanian	Za	Hemicryptophyte	NO
798	Astragalus shahinii Podlech & Maassoumi	Fabaceae	Irano-Turanian	Za	Hemicryptophyte	YES
799	Astragalus shahsavarii Maassoumi & Podlech	Fabaceae	Irano-Turanian	Za	Hemicryptophyte	YES
800	Astragalus shehbazii Zarre & Podlech	Fabaceae	Irano-Turanian	Za	Hemicryptophyte	YES
801	Astragalus shuturunkuhensis Podlech	Fabaceae	Irano-Turanian	Za	Hemicryptophyte	YES
802	Astragalus siahbishehensis Rahimin. & Ranjbar	Fabaceae	Irano-Turanian	Al	Hemicryptophyte	YES
803	Astragalus siahcheshmehensis Maassoumi & Podlech	Fabaceae	Irano-Turanian	Az	Hemicryptophyte	YES
804	Astragalus simakanensis Maassoumi & Hatami	Fabaceae	Irano-Turanian	Za	Chamaephyte	YES
805	Astragalus sirjaevii Zarre, Maassoumi & Podlech	Fabaceae	Irano-Turanian	Za	Chamaephyte	YES
806	Astragalus sisakhtianus Podlech & Maassoumi	Fabaceae	Irano-Turanian	Za	Hemicryptophyte	YES
807	Astragalus sitiens Bunge	Fabaceae	Irano-Turanian	Za, Az, Al, Ko	Hemicryptophyte	NO
808	Astragalus sivendicus Podlech & Maassoumi	Fabaceae	Irano-Turanian	Za	Hemicryptophyte	YES
809	Astragalus sojakii Podlech	Fabaceae	Irano-Turanian	Za, Ke	Hemicryptophyte	NO
810	Astragalus spachianiformis Podlech & Maassoumi	Fabaceae	Irano-Turanian	Ke	Hemicryptophyte	YES
811	Astragalus spachianus Boiss. & Buhse	Fabaceae	Irano-Turanian	Za, Ke, Ko	Hemicryptophyte	NO
812	Astragalus speciosus Boiss. & Hohen.	Fabaceae	Irano-Turanian	Za	Hemicryptophyte	NO
813	Astragalus sphaeranthus Boiss.	Fabaceae	Irano-Turanian	Za	Hemicryptophyte	NO
814	Astragalus stenolepis Fisch.	Fabaceae	Irano-Turanian	Al	Chamaephyte	NO
815	Astragalus stenostegius Boiss. & Hausskn.	Fabaceae	Irano-Turanian	Za	Chamaephyte	NO

816	Astragalus stepporum Podlech	Fabaceae	Saharo-Sindian		Hemicryptophyte	YES
817	Astragalus straussii Bornm.	Fabaceae	Irano-Turanian	Za, Al	Chamaephyte	NO
818	Astragalus strictissimus Podlech & Zarre	Fabaceae	Irano-Turanian	Az	Chamaephyte	YES
819	Astragalus subalpinus Boiss. & Buhse	Fabaceae	Irano-Turanian	Al	Hemicryptophyte	NO
820	Astragalus subaspadanus Maassoumi, F.Ghahrem. & Bagheri	Fabaceae	Irano-Turanian	Az	Hemicryptophyte	YES
821	Astragalus subbrevidens Maassoumi	Fabaceae	Irano-Turanian	Ko	Hemicryptophyte	YES
822	Astragalus subglaberrimus Podlech & Maassoumi	Fabaceae	Irano-Turanian	Al	Hemicryptophyte	YES
823	Astragalus subkohrudicus Maassoumi, F.Ghahrem. & Bagheri	Fabaceae	Irano-Turanian	Az	Chamaephyte	YES
824	Astragalus submitis Boiss. & Hohen.	Fabaceae	Irano-Turanian	Az	Chamaephyte	NO
825	Astragalus subpentanthus Maassoumi & Podlech	Fabaceae	Irano-Turanian	Za	Hemicryptophyte	YES
826	Astragalus subrecognitus Bagheri, Maassoumi & F.Ghahrem.	Fabaceae	Irano-Turanian	Az	Chamaephyte	YES
827	Astragalus subrosulariformis Sirj. & Rech.f.	Fabaceae	Irano-Turanian	Ko	Hemicryptophyte	YES
828	Astragalus subsecundus Boiss. & Hohen.	Fabaceae	Irano-Turanian	Za, Al	Hemicryptophyte	NO
829	Astragalus sumarensis Maassoumi	Fabaceae	Saharo-Sindian		Chamaephyte	YES
830	Astragalus susianus Boiss.	Fabaceae	Irano-Turanian	Za	Chamaephyte	NO
831	Astragalus sympileicalycinus Maassoumi & Nasseh	Fabaceae	Irano-Turanian	Ko	Chamaephyte	YES
832	Astragalus sympileicarpus Rech.f.	Fabaceae	Irano-Turanian	Ko	Hemicryptophyte	NO
833	Astragalus tabrizianus Buhse	Fabaceae	Irano-Turanian	Az, Za	Chamaephyte	NO
834	Astragalus taebiae Zarre & Podlech	Fabaceae	Irano-Turanian	Az	Hemicryptophyte	YES
835	Astragalus tahbaziae Zarre & Podlech	Fabaceae	Irano-Turanian	Za	Hemicryptophyte	YES
836	Astragalus taleshensis Bidarlord, F.Ghahrem. & Maassoumi	Fabaceae	Irano-Turanian	Az	Hemicryptophyte	YES
837	Astragalus talimansurensis Sirj. & Rech.f.	Fabaceae	Saharo-Sindian		Chamaephyte	NO
838	Astragalus tarumensis Sirj. & Rech.f.	Fabaceae	Irano-Turanian	Za, Ke	Chamaephyte	NO
839	Astragalus teheranicus Boiss & Hohen.	Fabaceae	Irano-Turanian	Az, Al, Za, Ke	Hemicryptophyte	NO
840	Astragalus tekabensis Maassoumi & Maroofi	Fabaceae	Irano-Turanian	Az	Hemicryptophyte	YES
841	Astragalus tenellus Bunge	Fabaceae	Irano-Turanian	Za	Hemicryptophyte	NO
842	Astragalus tenuiramosus Podlech & Zarre	Fabaceae	Irano-Turanian		Chamaephyte	NO
843	Astragalus tenuiscapus Freyn & Bornm.	Fabaceae	Irano-Turanian	Za, Ke	Hemicryptophyte	NO
844	Astragalus tenuissimus Zarre & Podlech	Fabaceae	Irano-Turanian	Al	Hemicryptophyte	YES
845	Astragalus termeanus Maassoumi & Podlech	Fabaceae	Saharo-Sindian		Hemicryptophyte	NO
846	Astragalus tietziae Ghahr. & Zarre	Fabaceae	Irano-Turanian	Al	Chamaephyte	YES

847	Astragalus torbathaydariyehensis Ranjbar & Zarin	Fabaceae	Irano-Turanian	Ko	Hemicryptophyte	YES
848	Astragalus touranicus Freitag & Podlech	Fabaceae	Irano-Turanian	Al, Ko	Hemicryptophyte	NO
849	Astragalus townsendii Zarre, Maassoumi & Podlech	Fabaceae	Irano-Turanian	Za	Chamaephyte	YES
850	Astragalus trachyacanthos Fisch.	Fabaceae	Irano-Turanian	Az, Al, Ke	Chamaephyte	NO
851	Astragalus tricholobus DC.	Fabaceae	Irano-Turanian	Az, Za, Al	Chamaephyte	NO
852	Astragalus turgidus Podlech	Fabaceae	Irano-Turanian	Za	Hemicryptophyte	YES
853	Astragalus tuyehensis Ghahr., Maassoumi & Ghahr.-Nejad	Fabaceae	Irano-Turanian	Al, Ko	Hemicryptophyte	NO
854	Astragalus typhaeformis Maassoumi	Fabaceae	Irano-Turanian	Al	Hemicryptophyte	YES
855	Astragalus urbanus Podlech & Maassoumi	Fabaceae	Irano-Turanian	Al	Hemicryptophyte	YES
856	Astragalus vanillae Boiss.	Fabaceae	Irano-Turanian	Za, Ke	Chamaephyte	NO
857	Astragalus vegetus Bunge	Fabaceae	Irano-Turanian	Az, Za, Al	Hemicryptophyte	NO
858	Astragalus veiskaramii Zarre, Podlech & Sabaii	Fabaceae	Irano-Turanian	Za	Hemicryptophyte	YES
859	Astragalus vereskensis Maassoumi & Podlech	Fabaceae	Irano-Turanian, Euro-Siberian	Al	Hemicryptophyte	NO
860	Astragalus vernaculus Podlech	Fabaceae	Irano-Turanian	Az, Za	Chamaephyte	NO
861	Astragalus vessalae Maassoumi & Podlech	Fabaceae	Irano-Turanian		Hemicryptophyte	YES
862	Astragalus vicinalis Zarre & Podlech	Fabaceae	Irano-Turanian	Za	Hemicryptophyte	YES
863	Astragalus vulcanicus Bornm.	Fabaceae	Irano-Turanian	Al	Hemicryptophyte	YES
864	Astragalus xiphidiopsis Bornm.	Fabaceae	Irano-Turanian	Za	Hemicryptophyte	NO
865	Astragalus yazdii (Vassilcz.) Podlech & Maassoumi	Fabaceae	Irano-Turanian	Za, Ke	Hemicryptophyte	YES
866	Astragalus yushensis Sabaii, Zarre & Podlech	Fabaceae	Irano-Turanian	Al	Chamaephyte	YES
867	Astragalus zagrosicus Boiss. & Hausskn.	Fabaceae	Irano-Turanian	Az, Za	Hemicryptophyte	NO
868	Astragalus zangooeianus Maassoumi, Safavi & Nasseh	Fabaceae	Irano-Turanian	Ko	Hemicryptophyte	YES
869	Astragalus zanjanensis Podlech & Maassoumi	Fabaceae	Irano-Turanian	Az	Hemicryptophyte	NO
870	Astragalus zarjabadensis Ranjbar	Fabaceae	Irano-Turanian	Az	Hemicryptophyte	YES
871	Astragalus zarreanus Ranjbar	Fabaceae	Irano-Turanian	Za	Chamaephyte	YES
872	Astragalus zerdanus Boiss.	Fabaceae	Irano-Turanian	Za	Hemicryptophyte	NO
873	Astragalus zoshkensis Ghahr.-Nejad	Fabaceae	Irano-Turanian	Ko	Chamaephyte	YES
874	Astragalus zourabadensis Zarre & Podlech	Fabaceae	Irano-Turanian	Az	Chamaephyte	YES
875	Astrodaucus persicus (Boiss.) Drude	Apiaceae	Irano-Turanian	Az, Za, Al, Ko	Hemicryptophyte	NO
876	Asyneuma multicaule (Boiss.) Rech.f. & Schiman-Czeika	Campanulaceae	Irano-Turanian	Za	Hemicryptophyte	NO

877	Atractylis delvarii Mozaff.	Asteraceae	Saharo-Sindian		Hemicryptophyte	YES
878	Atraphaxis aucheri Jaub. & Spach	Polygonaceae	Irano-Turanian	Al	Phanerophyte	YES
879	Atraphaxis binaludensis S. Tavakkoli, Mozaff. & Kaz.Osaloo	Polygonaceae	Irano-Turanian	Ko	Phanerophyte	YES
880	Atraphaxis intricata Mozaff.	Polygonaceae	Irano-Turanian	Ko	Phanerophyte	YES
881	Azilia eryngioides (Pau) Hedge & Lamond	Apiaceae	Irano-Turanian	Za	Hemicryptophyte	NO
882	Ballota platyloma Rech.f.	Lamiaceae	Irano-Turanian, Euro-Siberian	Za	Chamaephyte	NO
883	Bellevalia assadii Wendelbo	Asparagaceae	Irano-Turanian	Az	Geophyte	YES
884	Bellevalia cyanopoda Wendelbo	Asparagaceae	Irano-Turanian	Za	Geophyte	YES
885	Bellevalia decolorans Bornm.	Asparagaceae	Irano-Turanian	Za, Al	Geophyte	NO
886	Bellevalia heweri Wendelbo	Asparagaceae	Irano-Turanian	Za	Geophyte	YES
887	Bellevalia koeiei Rech.f.	Asparagaceae	Irano-Turanian	Za	Geophyte	YES
888	Bellevalia multicolor Wendelbo	Asparagaceae	Irano-Turanian	Az	Geophyte	YES
889	Bellevalia shiraziana Parsa	Asparagaceae	Irano-Turanian	Za	Geophyte	YES
890	Bellevalia tabriziana Turrill	Asparagaceae	Irano-Turanian	Az	Geophyte	YES
891	Bellevalia tristis Bornm.	Asparagaceae	Irano-Turanian	Za	Geophyte	YES
892	Bellevalia wendelboi Maassoumi & Jafari	Asparagaceae	Irano-Turanian	Za	Geophyte	YES
893	Biarum platyspathum Bornm.	Araceae	Irano-Turanian	Za	Geophyte	YES
894	Biarum straussii Engl.	Araceae	Irano-Turanian	Za	Geophyte	NO
895	Bienertia kavirense Akhani	Chenopodiaceae	Irano-Turanian		Therophyte	YES
896	Bromus frigidus Boiss. & Hausskn.	Poaceae	Irano-Turanian	Za	Hemicryptophyte	YES
897	Brossardia papyracea Boiss.	Brassicaceae	Irano-Turanian	Za	Hemicryptophyte	NO
898	Bufonia calycina Boiss. & Hausskn.	Brassicaceae	Irano-Turanian	Za	Hemicryptophyte	YES
899	Bufonia capitata Bornm.	Caryophyllaceae	Irano-Turanian	Za	Chamaephyte	YES
900	Bufonia capsularis Boiss. & Hausskn.	Caryophyllaceae	Irano-Turanian	Za, Al	Chamaephyte	NO
901	Bufonia enervis Boiss.	Caryophyllaceae	Irano-Turanian	Az, Za, Al	Chamaephyte	NO
902	Bufonia hebecalyx Boiss.	Caryophyllaceae	Irano-Turanian	Al	Chamaephyte	YES
903	Bufonia koelzii Rech.f.	Caryophyllaceae	Irano-Turanian	Al	Chamaephyte	NO
904	Bufonia kotschyana Boiss.	Caryophyllaceae	Irano-Turanian	Az, Za, Al	Chamaephyte	NO
905	Bufonia macrocarpa Ser.	Caryophyllaceae	Irano-Turanian	Za	Chamaephyte	NO
906	Bufonia micrantha Boiss. & Hausskn.	Caryophyllaceae	Irano-Turanian	Za	Chamaephyte	YES

907	Bufonia stapfii Bornm.	Caryophyllaceae	Irano-Turanian	Za	Chamaephyte	YES
908	Bunium luristanicum Rech.f.	Caryophyllaceae	Irano-Turanian	Za, Al, Ko	Geophyte	NO
909	Bunium wolffii Klyuikov	Apiaceae	Irano-Turanian	Al, Ko	Geophyte	NO
910	Bupleurum flexile Bornm. & Gauba	Apiaceae	Irano-Turanian	Al	Hemicryptophyte	NO
911	Bupleurum ghahremanii Mozaff.	Apiaceae	Irano-Turanian	Al	Hemicryptophyte	YES
912	Bupleurum gilanicum Mozaff.	Apiaceae	Irano-Turanian	Al	Hemicryptophyte	YES
913	Bupleurum wolffianum Bornm. ex H.Wolff	Apiaceae	Irano-Turanian	Al	Hemicryptophyte	YES
914	Caccinia actinobole Bunge	Apiaceae	Irano-Turanian	Al	Hemicryptophyte	NO
915	Caccinia kotschyi Boiss.	Boraginaceae	Irano-Turanian	Za	Hemicryptophyte	NO
916	Caccinia strigosa Boiss.	Boraginaceae	Irano-Turanian	Al	Hemicryptophyte	NO
917	Calendula aurantiaca Kotschy ex Boiss.	Boraginaceae	Irano-Turanian	Za	Therophyte	YES
918	Calligonum alatosetosum Maassoumi & Kazempour	Asteraceae	Saharo-Sindian		Phanerophyte	YES
919	Calligonum bungei Boiss.	Polygonaceae	Irano-Turanian		Phanerophyte	NO
920	Calligonum denticulatum Bunge ex Boiss.	Polygonaceae	Irano-Turanian		Phanerophyte	NO
921	Calligonum laristanicum Rech.f. & Schiman-Czeika	Polygonaceae	Saharo-Sindian		Phanerophyte	NO
922	Calligonum persicum (Boiss. & Buhse) Boiss.	Polygonaceae	Irano-Turanian, Euro-Siberian		Phanerophyte	NO
923	Calligonum schizopterum Rech.f. & Schiman-Czeika	Polygonaceae	Irano-Turanian, Saharo-Sindian		Phanerophyte	NO
924	Calligonum spinosetosum Maassoumi & Batooli	Polygonaceae	Irano-Turanian		Phanerophyte	YES
925	Calligonum stenopterum Bunge ex Boiss.	Polygonaceae	Irano-Turanian		Phanerophyte	YES
926	Campanula candida A.DC.	Polygonaceae	Irano-Turanian	Za	Hemicryptophyte	NO
927	Campanula escalerae Rech.f. & Schiman-Czeika	Campanulaceae	Irano-Turanian	Za	Hemicryptophyte	YES
928	Campanula gilliatii Milne-Redh. & Turrill	Campanulaceae	Irano-Turanian	Az	Hemicryptophyte	YES
929	Campanula hermannii Rech.f.	Campanulaceae	Irano-Turanian	Za	Hemicryptophyte	YES
930	Campanula humillima A.DC.	Campanulaceae	Irano-Turanian	Za	Hemicryptophyte	NO
931	Campanula hystricula Pau	Campanulaceae	Irano-Turanian	Za	Chamaephyte	YES
932	Campanula kermanica (Rech.f., Aellen & Esfand.) Rech.f.	Campanulaceae	Irano-Turanian	Za	Chamaephyte	NO
933	Campanula kurdistanica Advay & Maroofi	Campanulaceae	Irano-Turanian	Za	Hemicryptophyte	YES
934	Campanula lamondiae Rech.f.	Campanulaceae	Irano-Turanian	Az	Hemicryptophyte	YES
935	Campanula lourica Boiss.	Campanulaceae	Irano-Turanian	Al	Chamaephyte	NO
936	Campanula luristanica Freyn	Campanulaceae	Irano-Turanian	Za	Hemicryptophyte	NO

937	Campanula persepolitana Kotschy ex Boiss.	Campanulaceae	Irano-Turanian	Za, Az	Hemicryptophyte	NO
938	Campanula savalanica Fed.	Campanulaceae	Irano-Turanian	Az	Hemicryptophyte	YES
939	Centaurea albonitens Turrill	Campanulaceae	Irano-Turanian	Az	Hemicryptophyte	NO
940	Centaurea amadanensis Sch.Bip.	Asteraceae	Irano-Turanian	Az, Za	Hemicryptophyte	NO
941	Centaurea ardabilica Ranjbar & Heydari	Asteraceae	Irano-Turanian	Az	Hemicryptophyte	YES
942	Centaurea aucheri (DC.) Wagenitz	Asteraceae	Irano-Turanian	Az, Al, Za	Hemicryptophyte	NO
943	Centaurea aziziana Rech.f.	Asteraceae	Irano-Turanian	Az, Al	Hemicryptophyte	NO
944	Centaurea bachtiarica Hayek & Bornm.	Asteraceae	Irano-Turanian	Za	Hemicryptophyte	YES
945	Centaurea bavegehensis Ranjbar & Negaresh	Asteraceae	Irano-Turanian	Za	Hemicryptophyte	YES
946	Centaurea congesta Wagenitz	Asteraceae	Irano-Turanian	Az	Hemicryptophyte	NO
947	Centaurea elbursensis Boiss. & Buhse	Asteraceae	Irano-Turanian	Az, Al	Hemicryptophyte	YES
948	Centaurea elymaitica Mozaff.	Asteraceae	Saharo-Sindian		Hemicryptophyte	YES
949	Centaurea gabrielae (Bornm.) Wagenitz	Asteraceae	Irano-Turanian		Hemicryptophyte	NO
950	Centaurea galactochroa Rech.f.	Asteraceae	Irano-Turanian	Ko	Hemicryptophyte	YES
951	Centaurea gaubae (Bornm.) Wagenitz	Asteraceae	Irano-Turanian	Al, Za	Hemicryptophyte	NO
952	Centaurea geluensis Boiss. & Hausskn. ex Boiss.	Asteraceae	Irano-Turanian	Za	Hemicryptophyte	YES
953	Centaurea ghahremanii Wagenitz & Esfand.	Asteraceae	Irano-Turanian	Za	Hemicryptophyte	YES
954	Centaurea gilanica Bornm.	Asteraceae	Irano-Turanian	Az, Za, Al	Hemicryptophyte	NO
955	Centaurea golestanica Akhani & Wagenitz	Asteraceae	Euro-Siberian	Al	Hemicryptophyte	YES
956	Centaurea hyrcanica Bornm.	Asteraceae	Euro-Siberian	Al	Hemicryptophyte	NO
957	Centaurea iljinii Czerniak.	Asteraceae	Irano-Turanian	Ko	Hemicryptophyte	YES
958	Centaurea imperialis Hausskn. ex Bornm.	Asteraceae	Irano-Turanian	Za, Az	Hemicryptophyte	NO
959	Centaurea incanescens (DC.) Sch.Bip.	Asteraceae	Irano-Turanian	Az	Hemicryptophyte	NO
960	Centaurea intricata Boiss.	Asteraceae	Irano-Turanian	Za	Hemicryptophyte	NO
961	Centaurea irritans Wagenitz	Asteraceae	Irano-Turanian	Za	Hemicryptophyte	NO
962	Centaurea ispahanica Boiss.	Asteraceae	Irano-Turanian	Za, Ke	Hemicryptophyte	NO
963	Centaurea kabirkuhensis Mozaff., F.Ghahrem. & Fereid.	Asteraceae	Saharo-Sindian		Hemicryptophyte	YES
964	Centaurea kamyaranensis Ranjbar & Negaresh	Asteraceae	Irano-Turanian	Za	Hemicryptophyte	YES
965	Centaurea kandavanensis Wagenitz	Asteraceae	Irano-Turanian, Euro-Siberian	Al	Hemicryptophyte	NO
966	Centaurea karamianiae Negaresh	Asteraceae	Irano-Turanian	Za	Hemicryptophyte	YES

967	Centaurea khuzistanica Mozaff.	Asteraceae	Saharo-Sindian	Za	Hemicryptophyte	YES
968	Centaurea koeieana Bornm.	Asteraceae	Irano-Turanian	Za	Hemicryptophyte	NO
969	Centaurea lachnopus Rech.f.	Asteraceae	Irano-Turanian	Al	Hemicryptophyte	YES
970	Centaurea leuzeoides (Jaub. & Spech) Walp.	Asteraceae	Irano-Turanian	Za, Az, Al	Hemicryptophyte	NO
971	Centaurea luristanica Rech.f.	Asteraceae	Irano-Turanian, Saharo-Sindian	Za	Hemicryptophyte	NO
972	Centaurea microlonchoides Boiss.	Asteraceae	Irano-Turanian	Az, Za, Ke	Hemicryptophyte	NO
973	Centaurea nemecii Nábělek	Asteraceae	Irano-Turanian	Za	Therophyte	NO
974	Centaurea ochrocephala Wagenitz	Asteraceae	Irano-Turanian	Az	Hemicryptophyte	YES
975	Centaurea orumiehensis Ranjjbar & Negaresh	Asteraceae	Irano-Turanian	Az	Hemicryptophyte	YES
976	Centaurea pabotii Wagenitz	Asteraceae	Saharo-Sindian		Hemicryptophyte	NO
977	Centaurea paradoxa Mozaff.	Asteraceae	Irano-Turanian	Za	Hemicryptophyte	YES
978	Centaurea persica Boiss.	Asteraceae	Irano-Turanian	Za	Hemicryptophyte	NO
979	Centaurea phaeopappoides Bordz.	Asteraceae	Irano-Turanian	Az	Hemicryptophyte	YES
980	Centaurea phlomoides Boiss. & Hausskn. ex Boiss. & Hausskn.	Asteraceae	Irano-Turanian	Za	Hemicryptophyte	YES
981	Centaurea rahiminejadii Negaresh	Asteraceae	Irano-Turanian	Az	Hemicryptophyte	YES
982	Centaurea ravansarensis Ranjbar & Negaresh	Asteraceae	Irano-Turanian	Za	Hemicryptophyte	YES
983	Centaurea salmasensis Ranjbar & Heydari	Asteraceae	Irano-Turanian	Az	Hemicryptophyte	YES
984	Centaurea sanandajensis Ranjbar & Negaresh	Asteraceae	Irano-Turanian	Za	Hemicryptophyte	YES
985	Centaurea schmidii Wagenitz	Asteraceae	Irano-Turanian	Ko	Hemicryptophyte	YES
986	Centaurea shahuensis Ranjbar & Negaresh	Asteraceae	Irano-Turanian	Za	Hemicryptophyte	YES
987	Centaurea shehbazii Ranjbar & Negaresh	Asteraceae	Irano-Turanian	Za	Hemicryptophyte	YES
988	Centaurea sintenisiana Gand.	Asteraceae	Irano-Turanian	Al, Ko	Hemicryptophyte	NO
989	Centaurea solitaria Ranjbar & Negaresh	Asteraceae	Irano-Turanian	Za	Hemicryptophyte	YES
990	Centaurea sosnowskyi Grossh.	Asteraceae	Irano-Turanian	Az, Za	Hemicryptophyte	NO
991	Centaurea tabriziana Ranjbar & Heydari	Asteraceae	Irano-Turanian	Az	Hemicryptophyte	YES
992	Centaurea urvillei DC.	Asteraceae	Irano-Turanian	Az	Hemicryptophyte	NO
993	Centaurea ustulata DC.	Asteraceae	Irano-Turanian	Az, Za	Hemicryptophyte	NO
994	Centaurea wendelboi Wagenitz	Asteraceae	Saharo-Sindian		Hemicryptophyte	YES
995	Centaurea xeranthemoides Rech.f.	Asteraceae	Irano-Turanian	Za	Hemicryptophyte	NO
996	Centaurea zagrosmontana Ranjbar & Heydari	Asteraceae	Irano-Turanian	Za	Hemicryptophyte	YES

997	Centaurea zangulensis Ranjbar & Negaresh	Asteraceae	Euro-Siberian	Al	Hemicryptophyte	YES
998	Centaurea zuvandica (Sosn.) Sosn.	Asteraceae	Euro-Siberian	Al	Hemicryptophyte	NO
999	Cephalaria bojnordensis Ranjbar & Z.Ranjbar	Asteraceae	Irano-Turanian	Ko	Hemicryptophyte	YES
1000	Cephalaria juncea Boiss.	Dipsacaceae	Irano-Turanian	Az, Za	Hemicryptophyte	NO
1001	Cephalorrhynchus brassicifolius (Boiss.) Tuisl	Dipsacaceae	Irano-Turanian	Al, Ko	Hemicryptophyte	NO
1002	Cephalorrhynchus gorganicus (Rech.f. & Esfand.) Tuisl	Asteraceae	Euro-Siberian	Al	Hemicryptophyte	YES
1003	Cephalorrhynchus kossinskyi (Krasch.) Kirp.	Asteraceae	Irano-Turanian	Ko	Hemicryptophyte	NO
1004	Cephalorrhynchus microcephalus (DC.) Schchian	Asteraceae	Irano-Turanian	Az, Za, Al	Hemicryptophyte	NO
1005	Cerastium persicum Boiss.	Asteraceae	Irano-Turanian	Al, Za	Hemicryptophyte	YES
1006	Cerasus brachypetala Boiss.	Caryophyllaceae	Irano-Turanian	Az, Za	Phanerophyte	NO
1007	Cerasus chorossanica Pojark.	Rosaceae	Irano-Turanian	Ko	Phanerophyte	YES
1008	Cerasus paradoxa Dehshiri & Mozaff.	Rosaceae	Irano-Turanian	Za	Phanerophyte	YES
1009	Chaenorhinum foroughii Speta	Rosaceae	Irano-Turanian	Za	Hemicryptophyte	YES
1010	Chaenorhinum grossecostatum Speta	Scrophulariaceae	Irano-Turanian	Ke	Therophyte	YES
1011	Chaerophyllum khorossanicum Czerniak. ex Schischk.	Scrophulariaceae	Irano-Turanian	Al, Ko	Hemicryptophyte	NO
1012	Chaerophyllum nivale Hedge & Lamond	Apiaceae	Irano-Turanian	Za	Hemicryptophyte	YES
1013	Chamaegeron asterellus (Bornm.) Botsch.	Apiaceae	Irano-Turanian	Za, Ke	Hemicryptophyte	NO
1014	Chamaegeron keredjensis (Bornm. & Gauba) Grierson	Asteraceae	Irano-Turanian	Al	Hemicryptophyte	YES
1015	Chesneya kotschyi Boiss.	Asteraceae	Saharo-Sindian		Hemicryptophyte	YES
1016	Chorispora persica Boiss.	Fabaceae	Irano-Turanian	Az, Za	Therophyte	NO
1017	Cicer spiroceras Jaub. & Spach	Brassicaceae	Irano-Turanian	Za, Ke, Bl	Hemicryptophyte	NO
1018	Cicer stapfianum Rech.f.	Fabaceae	Irano-Turanian	Za	Hemicryptophyte	YES
1019	Cicer subaphyllum Boiss.	Fabaceae	Irano-Turanian	Za	Hemicryptophyte	YES
1020	Cicer tragacanthoides Jaub. & Spach	Fabaceae	Irano-Turanian	Al, Za, Ko	Hemicryptophyte	NO
1021	Cicerbita polyclada (Boiss.) Beauverd	Fabaceae	Irano-Turanian	Za	Hemicryptophyte	YES
1022	Cirsium bracteosum DC.	Asteraceae	Irano-Turanian	Za	Hemicryptophyte	YES
1023	Cirsium gadukense Petr.	Asteraceae	Irano-Turanian	Al	Hemicryptophyte	YES
1024	Cirsium iranicum Petr.	Asteraceae	Irano-Turanian	Az	Hemicryptophyte	YES
1025	Cirsium lappaceum (M.Bieb.) Fisch.	Asteraceae	Irano-Turanian	Al, Az	Hemicryptophyte	NO
1026	Cirsium pyramidale Bornm.	Asteraceae	Irano-Turanian	Ke	Hemicryptophyte	YES
1027	Cirsium spectabile DC.	Asteraceae	Irano-Turanian	Za, Ke	Hemicryptophyte	NO

1028	Cistanche eremodoxa Bornm.	Asteraceae	Irano-Turanian	Az, Za	Geophyte	YES
1029	Clastopus erubescens Hausskn.	Orobanchaceae	Irano-Turanian	Za	Hemicryptophyte	YES
1030	Clastopus vestitus (Desv.) Boiss.	Brassicaceae	Irano-Turanian	Al, Za	Chamaephyte	NO
1031	Clematis iranica Habibi, Ghorbani & Azizian	Brassicaceae	Irano-Turanian	Al	Hemicryptophyte	YES
1032	Cleome foliolosa DC.	Ranunculaceae	Irano-Turanian	Al, Za, Ke	Therophyte	NO
1033	Codonocephalum stenocalathium Rech.f.	Cleomaceae	Irano-Turanian	Az	Hemicryptophyte	YES
1034	Colchicum bakhtiaricum Matin & Iranshahr	Asteraceae	Irano-Turanian	Za, Ke	Geophyte	YES
1035	Colchicum varians (Freyn & Bornm.) Dyer	Colchicaceae	Irano-Turanian	Za, Ke	Geophyte	NO
1036	Colchicum wendelboi K.Perss.	Colchicaceae	Irano-Turanian	Az	Geophyte	NO
1037	Colpodium gillettii Bor	Colchicaceae	Irano-Turanian	Az	Hemicryptophyte	YES
1038	Colpodium violaceum (Boiss.) Griseb.	Poaceae	Irano-Turanian	Za, Al	Hemicryptophyte	NO
1039	Colutea gifana Parsa	Poaceae	Irano-Turanian	Ko	Phanerophyte	YES
1040	Colutea persica Boiss.	Fabaceae	Irano-Turanian	Al, Za, Ke	Phanerophyte	NO
1041	Colutea porphyrogramma Rech.f.	Fabaceae	Irano-Turanian	Al, Ko	Phanerophyte	NO
1042	Colutea uniflora Beck	Fabaceae	Euro-Siberian	Al	Phanerophyte	YES
1043	Consolida linarioides (Boiss.) Munz	Fabaceae	Irano-Turanian	Za	Therophyte	YES
1044	Consolida lorestanica Iranshahr	Ranunculaceae	Irano-Turanian	Za	Therophyte	YES
1045	Consolida teheranica (Boiss.) Rech.f.	Ranunculaceae	Irano-Turanian	Al	Therophyte	NO
1046	Consolida trigonelloides (Boiss.) Munz	Ranunculaceae	Irano-Turanian	Za, Ke	Therophyte	NO
1047	Convolvulus ammocharis Boiss. & Hausskn.	Ranunculaceae	Saharo-Sindian		Hemicryptophyte	YES
1048	Convolvulus argyracanthus Rech. f., Aellen & Esfand.	Convolvulaceae	Saharo-Sindian		Chamaephyte	YES
1049	Convolvulus cephalophorus Boiss.	Convolvulaceae	Saharo-Sindian		Hemicryptophyte	YES
1050	Convolvulus elymaiticus Mozaff.	Convolvulaceae	Saharo-Sindian		Chamaephyte	YES
1051	Convolvulus eremophilus Boiss. & Buhse	Convolvulaceae	Irano-Turanian		Chamaephyte	NO
1052	Convolvulus gonocladus Boiss.	Convolvulaceae	Saharo-Sindian		Hemicryptophyte	NO
1053	Convolvulus gracillimus Rech.f.	Convolvulaceae	Irano-Turanian	Al	Chamaephyte	YES
1054	Convolvulus iranicus J.R.I.Wood & Scotland	Convolvulaceae	Irano-Turanian	Za	Chamaephyte	YES
1055	Convolvulus koieanus Bornm.	Convolvulaceae	Irano-Turanian		Chamaephyte	YES
1056	Convolvulus oxysepalus Boiss.	Convolvulaceae	Irano-Turanian, Saharo-Sindian	Ke	Chamaephyte	NO
1057	Convolvulus schirazianus Boiss.	Convolvulaceae	Irano-Turanian	Za, Az	Hemicryptophyte	NO

1058	Convolvulus stapfii Rech.f.	Convolvulaceae	Saharo-Sindian		Hemicryptophyte	NO
1059	Convolvulus turrillianus Parsa	Convolvulaceae	Irano-Turanian, Saharo-Sindian		Chamaephyte	NO
1060	Convolvulus urosepalus Pau	Convolvulaceae	Irano-Turanian	Za	Chamaephyte	NO
1061	Corydalis firouzii Wendelbo	Convolvulaceae	Irano-Turanian	Al	Geophyte	YES
1062	Cotoneaster assadii Khat.	Fumariaceae	Euro-Siberian	Al	Phanerophyte	YES
1063	Cotoneaster esfandiarii Khat.	Rosaceae	Irano-Turanian	Al	Phanerophyte	YES
1064	Cotoneaster persicus Pojark.	Rosaceae	Irano-Turanian	Za, Ke	Phanerophyte	NO
1065	Cousinia adenostegia Rech.f.	Rosaceae	Irano-Turanian	Ko	Hemicryptophyte	YES
1066	Cousinia adenosticta Bornm.	Asteraceae	Irano-Turanian	Al	Chamaephyte	YES
1067	Cousinia aggregata DC.	Asteraceae	Irano-Turanian	Al, Za	Chamaephyte	NO
1068	Cousinia akredii Bornm. & Gauba	Asteraceae	Irano-Turanian	Al	Hemicryptophyte	YES
1069	Cousinia albescens Winkl. & Strauss ex Winkl.	Asteraceae	Irano-Turanian	Za	Hemicryptophyte	YES
1070	Cousinia albida DC.	Asteraceae	Irano-Turanian	Za	Hemicryptophyte	YES
1071	Cousinia alexeenkoana Bornm.	Asteraceae	Irano-Turanian	Za, Al	Hemicryptophyte	NO
1072	Cousinia alfredii Bornm. & Gauba	Asteraceae	Irano-Turanian	Al	Hemicryptophyte	YES
1073	Cousinia amicorum Tscherneva, Joharchi & Ghahrem.-Nejad	Asteraceae	Irano-Turanian	Ko	Hemicryptophyte	YES
1074	Cousinia amplissima (Boiss.) Boiss.	Asteraceae	Irano-Turanian	Az, Za, Al	Hemicryptophyte	NO
1075	Cousinia antonowii C.Winkl.	Asteraceae	Irano-Turanian	Ko	Hemicryptophyte	YES
1076	Cousinia arakensis Attar & Djavadi	Asteraceae	Irano-Turanian	Za	Hemicryptophyte	NO
1077	Cousinia araneosa DC.	Asteraceae	Irano-Turanian	Za, Ke	Hemicryptophyte	NO
1078	Cousinia archibaldii Rech.f.	Asteraceae	Irano-Turanian	Za	Chamaephyte	NO
1079	Cousinia arctotidifolia Bunge	Asteraceae	Irano-Turanian	Ko, Al	Hemicryptophyte	NO
1080	Cousinia ardalensis Attar & Djavadi	Asteraceae	Irano-Turanian	Za	Hemicryptophyte	YES
1081	Cousinia argentea Mehregan & Assadi	Asteraceae	Irano-Turanian	Ko	Chamaephyte	YES
1082	Cousinia assadii Attar	Asteraceae	Irano-Turanian	Za	Hemicryptophyte	YES
1083	Cousinia assyriaca Jaub. & Spach	Asteraceae	Irano-Turanian	Za, Az	Hemicryptophyte	NO
1084	Cousinia atrobracteata Attar	Asteraceae	Irano-Turanian	Az	Hemicryptophyte	YES
1085	Cousinia atropatana Bunge	Asteraceae	Irano-Turanian	Az	Hemicryptophyte	YES
1086	Cousinia attariae Assadi & Joharchi	Asteraceae	Irano-Turanian	Ko	Hemicryptophyte	YES
1087	Cousinia azerbaidjanica Djavadi, Attar & Najafi	Asteraceae	Irano-Turanian	Az	Hemicryptophyte	YES

1088	Cousinia bachtiarica Boiss. & Hausskn.	Asteraceae	Irano-Turanian	Za	Hemicryptophyte	NO
1089	Cousinia barbeyi C.Winkl.	Asteraceae	Irano-Turanian	Za	Hemicryptophyte	YES
1090	Cousinia barezica Assadi	Asteraceae	Irano-Turanian	Ke	Hemicryptophyte	YES
1091	Cousinia bazoftensis Attar	Asteraceae	Irano-Turanian	Za	Hemicryptophyte	YES
1092	Cousinia beauverdiana Bornm.	Asteraceae	Irano-Turanian	Ke	Hemicryptophyte	YES
1093	Cousinia belangeri DC.	Asteraceae	Irano-Turanian	Al, Za	Hemicryptophyte	NO
1094	Cousinia bienerti Bunge	Asteraceae	Irano-Turanian	Ko	Hemicryptophyte	YES
1095	Cousinia bijarensis Rech.f.	Asteraceae	Irano-Turanian	Az	Chamaephyte	YES
1096	Cousinia bobekii Rech.f.	Asteraceae	Irano-Turanian	Az	Hemicryptophyte	NO
1097	Cousinia boissieri Buhse	Asteraceae	Irano-Turanian	Az	Hemicryptophyte	YES
1098	Cousinia brevicaulis Attar, Mozaff. & Mirtadz.	Asteraceae	Irano-Turanian		Hemicryptophyte	YES
1099	Cousinia calcitrapa Boiss.	Asteraceae	Irano-Turanian	Az, Ke, Za	Hemicryptophyte	NO
1100	Cousinia calocephala Jaub. & Spach	Asteraceae	Irano-Turanian	Az, Al, Za	Hemicryptophyte	NO
1101	Cousinia calolepis Boiss.	Asteraceae	Irano-Turanian	Al	Hemicryptophyte	YES
1102	Cousinia candolleana Jaub. & Spach	Asteraceae	Irano-Turanian	Za	Hemicryptophyte	YES
1103	Cousinia canescens DC.	Asteraceae	Irano-Turanian	Az, Za	Hemicryptophyte	NO
1104	Cousinia cavarae Bornm.	Asteraceae	Irano-Turanian	Ko	Hemicryptophyte	YES
1105	Cousinia chaetocephala Kult.	Asteraceae	Irano-Turanian	Ko	Chamaephyte	YES
1106	Cousinia chamaepeuce Boiss.	Asteraceae	Irano-Turanian	Al	Hemicryptophyte	NO
1107	Cousinia chlorocephala C.A.Mey. ex DC.	Asteraceae	Irano-Turanian	Az	Hemicryptophyte	YES
1108	Cousinia chlorosphaera Bornm.	Asteraceae	Irano-Turanian	Za	Hemicryptophyte	NO
1109	Cousinia chrysacantha Jaub. & Spach	Asteraceae	Irano-Turanian	Az	Hemicryptophyte	NO
1110	Cousinia chrysandra Bornm. & Gauba	Asteraceae	Irano-Turanian	Ko	Hemicryptophyte	YES
1111	Cousinia commutata Bunge	Asteraceae	Irano-Turanian	Al	Hemicryptophyte	NO
1112	Cousinia concinna Boiss. & Hausskn.	Asteraceae	Irano-Turanian	Za	Hemicryptophyte	NO
1113	Cousinia concolor Bunge	Asteraceae	Irano-Turanian	Al, Ko	Hemicryptophyte	YES
1114	Cousinia contumax C.Winkl. & Bornm.	Asteraceae	Irano-Turanian	Za	Hemicryptophyte	YES
1115	Cousinia cordifolia Djavadi & Attar	Asteraceae	Irano-Turanian	Al	Hemicryptophyte	YES
1116	Cousinia crassipes Kult.	Asteraceae	Irano-Turanian	Ko	Hemicryptophyte	YES
1117	Cousinia crispa Jaub. & Spach	Asteraceae	Irano-Turanian	Al	Hemicryptophyte	NO
1118	Cousinia curvibracteata Mehregan	Asteraceae	Irano-Turanian	Za	Hemicryptophyte	YES

1119	Cousinia cylindracea Boiss.	Asteraceae	Irano-Turanian	Al, Za, Az	Hemicryptophyte	NO
1120	Cousinia cylindrocephala Jaub. & Spach	Asteraceae	Irano-Turanian		Hemicryptophyte	YES
1121	Cousinia czerniakowskae Kult.	Asteraceae	Irano-Turanian	Ko	Hemicryptophyte	YES
1122	Cousinia dalahuensis Attar & Ghahr.	Asteraceae	Irano-Turanian	Za	Hemicryptophyte	YES
1123	Cousinia dasylepis Kult.	Asteraceae	Irano-Turanian	Ko	Hemicryptophyte	YES
1124	Cousinia decipiens Boiss. & Buhse	Asteraceae	Irano-Turanian	Al	Hemicryptophyte	NO
1125	Cousinia decumbens Rech.f.	Asteraceae	Irano-Turanian	Al	Hemicryptophyte	YES
1126	Cousinia denaensis Attar & Djavadi	Asteraceae	Irano-Turanian	Za	Hemicryptophyte	NO
1127	Cousinia deserti Bunge	Asteraceae	Irano-Turanian	Ko	Hemicryptophyte	YES
1128	Cousinia diezii Rech.f.	Asteraceae	Irano-Turanian	Ko	Hemicryptophyte	YES
1129	Cousinia dipterocarpa Bornm. & Rech.f.	Asteraceae	Irano-Turanian	Ko	Hemicryptophyte	YES
1130	Cousinia discolor Bunge	Asteraceae	Irano-Turanian	Ko	Hemicryptophyte	YES
1131	Cousinia disfulensis Bornm.	Asteraceae	Irano-Turanian	Za	Hemicryptophyte	YES
1132	Cousinia eburnea Bornm.	Asteraceae	Irano-Turanian	Za	Hemicryptophyte	YES
1133	Cousinia ecbatanensis Bornm.	Asteraceae	Irano-Turanian	Za	Hemicryptophyte	YES
1134	Cousinia edmondsonii Rech.f.	Asteraceae	Irano-Turanian	Al	Chamaephyte	YES
1135	Cousinia ehrysandra Bornm. & Gauba	Asteraceae	Irano-Turanian	Ko	Hemicryptophyte	YES
1136	Cousinia elata Boiss. & Buhse	Asteraceae	Irano-Turanian	Al, Ko	Hemicryptophyte	NO
1137	Cousinia elburzensis Attar, Mahdigholi & Ghahrem.	Asteraceae	Irano-Turanian	Al	Hemicryptophyte	YES
1138	Cousinia erinacea Jaub. & Spach	Asteraceae	Irano-Turanian, Euro-Siberian	Al	Hemicryptophyte	YES
1139	Cousinia eriobasis Bunge	Asteraceae	Irano-Turanian	Za, Ke	Chamaephyte	NO
1140	Cousinia eriophylla (Kult.) Bornm.	Asteraceae	Irano-Turanian	Ko	Chamaephyte	YES
1141	Cousinia eriorrhiza Bornm.	Asteraceae	Irano-Turanian	Za	Hemicryptophyte	YES
1142	Cousinia eryngioides Boiss.	Asteraceae	Irano-Turanian	Al, Ke, Ko, Az	Hemicryptophyte	NO
1143	Cousinia esfandiarii Rech.f. & Aellen	Asteraceae	Irano-Turanian	Al	Hemicryptophyte	NO
1144	Cousinia euchlora Bornm. & Rech.f.	Asteraceae	Irano-Turanian	Ko	Hemicryptophyte	YES
1145	Cousinia fabrorum Rech.f.	Asteraceae	Irano-Turanian	Al, Ko	Hemicryptophyte	NO
1146	Cousinia falcinella Bornm.	Asteraceae	Irano-Turanian	Al	Hemicryptophyte	YES
1147	Cousinia farimanensis Assadi	Asteraceae	Irano-Turanian	Ko	Chamaephyte	YES
1148	Cousinia firuzkuhensis Rech.f.	Asteraceae	Irano-Turanian	Al	Hemicryptophyte	YES

1149	Cousinia fragilis C.Winkl. & Bornm.	Asteraceae	Irano-Turanian	Ke	Hemicryptophyte	YES
1150	Cousinia fragillima Rech.f.	Asteraceae	Irano-Turanian	Ko	Chamaephyte	YES
1151	Cousinia freynii Bornm. & Sint.	Asteraceae	Irano-Turanian	Ko	Hemicryptophyte	YES
1152	Cousinia gabrielae Bornm.	Asteraceae	Irano-Turanian		Hemicryptophyte	YES
1153	Cousinia gaharensis Attar & Djavadi	Asteraceae	Irano-Turanian	Za	Hemicryptophyte	YES
1154	Cousinia gatchsaranica Mehregan, Assadi & Attar	Asteraceae	Irano-Turanian	Za	Hemicryptophyte	YES
1155	Cousinia gaubae Bornm.	Asteraceae	Irano-Turanian	Al	Hemicryptophyte	YES
1156	Cousinia gedrosiaca Bornm. & Gauba	Asteraceae	Irano-Turanian	Ke, Lo	Hemicryptophyte	YES
1157	Cousinia ghahremanii Mirtadz. & Attar	Asteraceae	Irano-Turanian	Ke	Hemicryptophyte	YES
1158	Cousinia gilanica Bornm.	Asteraceae	Irano-Turanian, Euro-Siberian	Al	Hemicryptophyte	YES
1159	Cousinia gilliatii Rech.f.	Asteraceae	Irano-Turanian	Az	Hemicryptophyte	YES
1160	Cousinia glaucopsis Bornm. & Rech.f.	Asteraceae	Irano-Turanian	Al	Hemicryptophyte	YES
1161	Cousinia gmelini C.Winkl.	Asteraceae	Irano-Turanian	Al	Hemicryptophyte	NO
1162	Cousinia golestanica Attar	Asteraceae	Irano-Turanian		Chamaephyte	YES
1163	Cousinia gracilis Boiss.	Asteraceae	Irano-Turanian	Za	Hemicryptophyte	NO
1164	Cousinia grandis C.A.Mey. ex DC.	Asteraceae	Irano-Turanian	Az	Hemicryptophyte	NO
1165	Cousinia hablitzii C.A.Mey. ex DC.	Asteraceae	Irano-Turanian	Al	Hemicryptophyte	YES
1166	Cousinia hamosa C.A.Mey.	Asteraceae	Irano-Turanian	Az	Hemicryptophyte	YES
1167	Cousinia harazensis Rech.f.	Asteraceae	Irano-Turanian	Al	Hemicryptophyte	YES
1168	Cousinia hazarensis Mirtadz. & Attar	Asteraceae	Irano-Turanian	Ke	Hemicryptophyte	YES
1169	Cousinia heliantha Bunge	Asteraceae	Irano-Turanian	Ko	Hemicryptophyte	NO
1170	Cousinia hergtiana Bornm.	Asteraceae	Irano-Turanian	Za	Chamaephyte	NO
1171	Cousinia hololeuca Bunge	Asteraceae	Irano-Turanian	Al	Chamaephyte	YES
1172	Cousinia horrida Kult.	Asteraceae	Irano-Turanian	Ko	Hemicryptophyte	YES
1173	Cousinia hypochionea Bornm.	Asteraceae	Euro-Siberian	Al	Hemicryptophyte	YES
1174	Cousinia hypoleuca Boiss.	Asteraceae	Irano-Turanian	Al, Za, Ko	Hemicryptophyte	NO
1175	Cousinia hypopolia Bornm. & Sint.	Asteraceae	Irano-Turanian	Al, Ko	Hemicryptophyte	NO
1176	Cousinia ilicifolia Jaub. & Spach	Asteraceae	Irano-Turanian	Za	Hemicryptophyte	YES
1177	Cousinia incompta DC.	Asteraceae	Irano-Turanian	?	Hemicryptophyte	YES
1178	Cousinia inflata Boiss. & Hausskn. ex Boiss.	Asteraceae	Irano-Turanian	Za	Hemicryptophyte	YES

1179	Cousinia iranica Winkl. & Strauss ex Winkl.	Asteraceae	Irano-Turanian	Za	Hemicryptophyte	YES
1180	Cousinia iranshahriana Attar & Maroofi	Asteraceae	Irano-Turanian	Za	Chamaephyte	NO
1181	Cousinia iranshahrii Rech.f.	Asteraceae	Irano-Turanian	Ko	Hemicryptophyte	YES
1182	Cousinia irritans Rech.f.	Asteraceae	Irano-Turanian	Al, Ko	Hemicryptophyte	NO
1183	Cousinia isfahanica Assadi	Asteraceae	Irano-Turanian	Za	Hemicryptophyte	YES
1184	Cousinia Jacobsii Rech.f.	Asteraceae	Irano-Turanian	Za	Hemicryptophyte	YES
1185	Cousinia joharchii Assadi & Mehregan	Asteraceae	Irano-Turanian	Al	Hemicryptophyte	YES
1186	Cousinia kadereitii Mehregan & Assadi	Asteraceae	Irano-Turanian	Ko	Chamaephyte	YES
1187	Cousinia kandavanensis Attar	Asteraceae	Irano-Turanian	Al	Hemicryptophyte	YES
1188	Cousinia karkasensis Mehregan & Djavadi	Asteraceae	Irano-Turanian	Za	Hemicryptophyte	YES
1189	Cousinia kashanensis Rech.f. & Esfand.	Asteraceae	Irano-Turanian	Za	Hemicryptophyte	YES
1190	Cousinia keredjensis Bornm. & Gauba	Asteraceae	Irano-Turanian	Al	Hemicryptophyte	YES
1191	Cousinia kermanshahensis Attar, Ghahr. & Assadi	Asteraceae	Irano-Turanian	Za	Hemicryptophyte	YES
1192	Cousinia khorasanica Djavadi & Attar	Asteraceae	Irano-Turanian	Ko	Hemicryptophyte	YES
1193	Cousinia kilouyensis Djavadi & Attar	Asteraceae	Irano-Turanian	Za	Hemicryptophyte	YES
1194	Cousinia komarowii (Kuntze) C.Winkl.	Asteraceae	Irano-Turanian	Ko	Hemicryptophyte	YES
1195	Cousinia komidjanensis Mehregan	Asteraceae	Irano-Turanian	Za	Hemicryptophyte	YES
1196	Cousinia kornhuberi Heimerl	Asteraceae	Irano-Turanian	Za	Hemicryptophyte	YES
1197	Cousinia korowiakowi C.Winkl.	Asteraceae	Irano-Turanian	Za	Hemicryptophyte	YES
1198	Cousinia kotschyi Boiss.	Asteraceae	Irano-Turanian	Za, Ko, Ke	Hemicryptophyte	NO
1199	Cousinia kurdistanica Attar	Asteraceae	Irano-Turanian	Za	Hemicryptophyte	YES
1200	Cousinia labrorum Rech.f.	Asteraceae	Irano-Turanian		Hemicryptophyte	YES
1201	Cousinia lactiflora Rech.f.	Asteraceae	Irano-Turanian	Za	Hemicryptophyte	YES
1202	Cousinia lasiandra Bunge	Asteraceae	Irano-Turanian	Ko	Hemicryptophyte	NO
1203	Cousinia lasiolepis Boiss.	Asteraceae	Irano-Turanian	Za, Ke	Hemicryptophyte	YES
1204	Cousinia lepida Bunge ex Boiss.	Asteraceae	Irano-Turanian	Ko	Chamaephyte	NO
1205	Cousinia lignosissima Rech.f.	Asteraceae	Irano-Turanian		Chamaephyte	YES
1206	Cousinia linczewskii Juz.	Asteraceae	Irano-Turanian		Hemicryptophyte	YES
1207	Cousinia longibracteata Attar & Mirtadz.	Asteraceae	Irano-Turanian		Hemicryptophyte	YES
1208	Cousinia longifolia C.Winkl. & Bornm.	Asteraceae	Irano-Turanian	Za, Ke	Hemicryptophyte	NO
1209	Cousinia lordeganensis Mehregan	Asteraceae	Irano-Turanian	Za	Hemicryptophyte	YES

1210	Cousinia lucida DC.	Asteraceae	Irano-Turanian	Za, Al	Hemicryptophyte	YES
1211	Cousinia lurestanica Attar & Djavadi	Asteraceae	Irano-Turanian	Za	Hemicryptophyte	YES
1212	Cousinia lurorum Bornm.	Asteraceae	Irano-Turanian	Za	Hemicryptophyte	NO
1213	Cousinia maassoumii Assadi	Asteraceae	Irano-Turanian	Za	Hemicryptophyte	YES
1214	Cousinia macrocephala C.A.Mey.	Asteraceae	Irano-Turanian	Az	Hemicryptophyte	NO
1215	Cousinia macroptera C.A.Mey. ex DC.	Asteraceae	Irano-Turanian	Az	Hemicryptophyte	NO
1216	Cousinia manouchehrii Rech.f. & Esfan.	Asteraceae	Irano-Turanian	Za	Hemicryptophyte	YES
1217	Cousinia mehreganii Assadi	Asteraceae	Irano-Turanian	Ko	Chamaephyte	YES
1218	Cousinia meluarmanica Rech.f.	Asteraceae	Irano-Turanian	Al	Hemicryptophyte	YES
1219	Cousinia meshhedensis Bornm. & Rech.f.	Asteraceae	Irano-Turanian	Al, Ko	Hemicryptophyte	NO
1220	Cousinia microcephala C.A.Mey. ex DC.	Asteraceae	Irano-Turanian	Az	Hemicryptophyte	YES
1221	Cousinia mobayenii Ghahr. & Attar	Asteraceae	Irano-Turanian	Za	Hemicryptophyte	YES
1222	Cousinia monocephala Bunge	Asteraceae	Irano-Turanian		Hemicryptophyte	YES
1223	Cousinia mozdouranensis Djavadi & Attar	Asteraceae	Irano-Turanian	Ko	Hemicryptophyte	YES
1224	Cousinia mutehensis Rech.f.	Asteraceae	Irano-Turanian	Za	Hemicryptophyte	YES
1225	Cousinia nekarmanica Rech.f.	Asteraceae	Irano-Turanian	Al	Hemicryptophyte	YES
1226	Cousinia neurocentra Bunge	Asteraceae	Irano-Turanian	Al, Ke	Chamaephyte	NO
1227	Cousinia noeana Boiss.	Asteraceae	Irano-Turanian	Za	Hemicryptophyte	NO
1228	Cousinia nujianensis Attar, Ghahr., Saber & Zarre	Asteraceae	Irano-Turanian	Za	Chamaephyte	YES
1229	Cousinia oligocephala Hausskn. ex Boiss.	Asteraceae	Irano-Turanian	Za	Hemicryptophyte	YES
1230	Cousinia onopordioides Ledeb.	Asteraceae	Irano-Turanian	Al, Ko, Ke	Hemicryptophyte	NO
1231	Cousinia oreodoxa Bornm. & Sint.	Asteraceae	Irano-Turanian	Ko	Hemicryptophyte	NO
1232	Cousinia orthoclada Hausskn. & Bornm.	Asteraceae	Irano-Turanian	Za	Hemicryptophyte	NO
1233	Cousinia oshtorankuhensis Attar	Asteraceae	Irano-Turanian	Za	Hemicryptophyte	YES
1234	Cousinia ottonis Bornm.	Asteraceae	Irano-Turanian	Za	Hemicryptophyte	YES
1235	Cousinia papillosa Djavadi & Attar	Asteraceae	Irano-Turanian	Ko	Hemicryptophyte	YES
1236	Cousinia parsana Ghahr., Iranshahr & Attar	Asteraceae	Irano-Turanian	Za	Hemicryptophyte	YES
1237	Cousinia pasargardensis Attar	Asteraceae	Irano-Turanian	Za	Hemicryptophyte	YES
1238	Cousinia pergamacea Boiss. & Hausskn. ex Boiss. & Hausskn.	Asteraceae	Irano-Turanian	Az, Za	Hemicryptophyte	NO
1239	Cousinia persica Djavadi & Attar	Asteraceae	Irano-Turanian	Al	Hemicryptophyte	YES
1240	Cousinia perspolitana Attar & Ghahr.	Asteraceae	Irano-Turanian	Za	Hemicryptophyte	YES

1241	Cousinia pinarocephala Boiss.	Asteraceae	Irano-Turanian	Al	Hemicryptophyte	NO
1242	Cousinia piptocephala Bunge	Asteraceae	Irano-Turanian	Ko, Ke, Za	Hemicryptophyte	NO
1243	Cousinia platyacantha Bunge	Asteraceae	Irano-Turanian	Ko	Chamaephyte	YES
1244	Cousinia platyptera Bornm.	Asteraceae	Irano-Turanian	Za	Hemicryptophyte	YES
1245	Cousinia platyraphis Kult.	Asteraceae	Irano-Turanian	Ko	Hemicryptophyte	YES
1246	Cousinia pseudocandolleana Assadi	Asteraceae	Irano-Turanian	Ko	Hemicryptophyte	YES
1247	Cousinia pterocaulos (C.A.Mey.) Rech.f.	Asteraceae	Irano-Turanian	Az, Al, Za	Hemicryptophyte	NO
1248	Cousinia pugionifera Jaub. & Spach	Asteraceae	Irano-Turanian	Za	Hemicryptophyte	NO
1249	Cousinia qarehbilensis Rech.f.	Asteraceae	Irano-Turanian		Hemicryptophyte	YES
1250	Cousinia raphiocephala Rech f.	Asteraceae	Irano-Turanian	Za	Chamaephyte	YES
1251	Cousinia raphiostegia Rech.f.	Asteraceae	Irano-Turanian		Chamaephyte	YES
1252	Cousinia rechingerae Bornm.	Asteraceae	Irano-Turanian		Hemicryptophyte	NO
1253	Cousinia rechingerorum Bornm.	Asteraceae	Irano-Turanian	Ko	Chamaephyte	YES
1254	Cousinia recurvata DC.	Asteraceae	Irano-Turanian	Al, Za, Ko	Hemicryptophyte	NO
1255	Cousinia remingerorum Bornm.	Asteraceae	Irano-Turanian	Ko	Hemicryptophyte	YES
1256	Cousinia renominata Rech.f.	Asteraceae	Euro-Siberian		Hemicryptophyte	YES
1257	Cousinia rhabdodes Bornm. & Rech.f.	Asteraceae	Irano-Turanian	Al	Chamaephyte	YES
1258	Cousinia rhaphiocephala Rech.f.	Asteraceae	Irano-Turanian	Za	Chamaephyte	YES
1259	Cousinia sabalanica Attar, Ghahr. & Assadi	Asteraceae	Irano-Turanian	Az	Hemicryptophyte	YES
1260	Cousinia sabzevarensis Rech.f.	Asteraceae	Irano-Turanian	Ko, Za	Hemicryptophyte	YES
1261	Cousinia sagittata C.Winkl. & Strauss	Asteraceae	Irano-Turanian	Za	Hemicryptophyte	NO
1262	Cousinia sahandica Attar & Djavadi	Asteraceae	Irano-Turanian	Az	Hemicryptophyte	YES
1263	Cousinia sakawensis Boiss. & Hausskn. ex Boiss. & Hausskn.	Asteraceae	Irano-Turanian	Za	Hemicryptophyte	YES
1264	Cousinia saloukensis Mehregan	Asteraceae	Irano-Turanian	Ko	Chamaephyte	YES
1265	Cousinia sardashtensis Rech.f.	Asteraceae	Irano-Turanian	Az	Hemicryptophyte	YES
1266	Cousinia sarzehensis Attar, Ghahr. & Assadi	Asteraceae	Irano-Turanian	Ke	Hemicryptophyte	YES
1267	Cousinia schindleriana Bornm. & Gauba	Asteraceae	Irano-Turanian	Ko	Hemicryptophyte	YES
1268	Cousinia schiraziana Attar	Asteraceae	Irano-Turanian	Za	Hemicryptophyte	YES
1269	Cousinia seidlitzii Bunge	Asteraceae	Irano-Turanian	Az	Hemicryptophyte	NO
1270	Cousinia shahuensis Attar	Asteraceae	Irano-Turanian	Za	Chamaephyte	YES
1271	Cousinia shahvarica Rech.f.	Asteraceae	Irano-Turanian	Al	Hemicryptophyte	NO

1272	Cousinia shebliensis Ghahr., Iranshahr & Attar	Asteraceae	Irano-Turanian	Az	Hemicryptophyte	YES
1273	Cousinia sheidaii Attar, Ghahrem. & Mahdigholi	Asteraceae	Irano-Turanian	Za	Hemicryptophyte	YES
1274	Cousinia shulabadensis Attar & Ghahr.	Asteraceae	Irano-Turanian	Za	Hemicryptophyte	YES
1275	Cousinia sicigera C.Winkl. & Bornm.	Asteraceae	Irano-Turanian	Ke	Hemicryptophyte	NO
1276	Cousinia silvanica Attar	Asteraceae	Irano-Turanian	Az	Hemicryptophyte	YES
1277	Cousinia silyboides Jaub. & Spach	Asteraceae	Irano-Turanian	Za	Hemicryptophyte	NO
1278	Cousinia smirnowii Trautv.	Asteraceae	Irano-Turanian	Al, Ko	Hemicryptophyte	NO
1279	Cousinia spathulata Kult.	Asteraceae	Irano-Turanian	Ko	Hemicryptophyte	YES
1280	Cousinia sphaerocephala Jaub. & Spach	Asteraceae	Irano-Turanian	Al	Hemicryptophyte	YES
1281	Cousinia stahliana Bornm. & Gauba	Asteraceae	Irano-Turanian	Al, Ko	Hemicryptophyte	NO
1282	Cousinia straussii Hausskn. & Winkl. ex Winkl.	Asteraceae	Irano-Turanian	Za	Hemicryptophyte	YES
1283	Cousinia subinflata Bornm.	Asteraceae	Irano-Turanian	Za	Hemicryptophyte	YES
1284	Cousinia subpectinata Mirtadz., Attar & Assadi	Asteraceae	Irano-Turanian	Ke	Chamaephyte	YES
1285	Cousinia tabrisiana Bunge	Asteraceae	Irano-Turanian	Az	Chamaephyte	NO
1286	Cousinia taybadensis Djavadi & Attar	Asteraceae	Irano-Turanian		Chamaephyte	YES
1287	Cousinia tenuifolia C.A.Mey. ex DC.	Asteraceae	Irano-Turanian	Az	Hemicryptophyte	NO
1288	Cousinia tenuiramula Rech.f.	Asteraceae	Irano-Turanian	Za	Hemicryptophyte	YES
1289	Cousinia termei Rech.f.	Asteraceae	Irano-Turanian	Ko	Hemicryptophyte	YES
1290	Cousinia tetanocephala Bornm. & Gauba	Asteraceae	Irano-Turanian	Ko	Hemicryptophyte	YES
1291	Cousinia thamnodes Boiss. & Hausskn.	Asteraceae	Irano-Turanian	Za	Hemicryptophyte	YES
1292	Cousinia touchalensis Attar	Asteraceae	Irano-Turanian	Al	Hemicryptophyte	YES
1293	Cousinia trachylepis Bunge	Asteraceae	Irano-Turanian	Al	Hemicryptophyte	NO
1294	Cousinia trachyphyllaria Bornm. & Rech.f.	Asteraceae	Irano-Turanian	Ko	Hemicryptophyte	YES
1295	Cousinia triflora Schrenk	Asteraceae	Irano-Turanian	Ko	Hemicryptophyte	YES
1296	Cousinia turcomanica C.Winkl.	Asteraceae	Irano-Turanian	Al, Ko	Hemicryptophyte	NO
1297	Cousinia urumiensis Bornm.	Asteraceae	Irano-Turanian	Az	Hemicryptophyte	NO
1298	Cousinia verbascifolia Bunge	Asteraceae	Irano-Turanian	Ko	Hemicryptophyte	NO
1299	Cousinia wendelboi Rech.f.	Asteraceae	Irano-Turanian	Al	Chamaephyte	YES
1300	Cousinia wilhelminae Rech.f.	Asteraceae	Irano-Turanian	Az	Hemicryptophyte	YES
1301	Cousinia xiphiolepis Boiss.	Asteraceae	Irano-Turanian	Al	Hemicryptophyte	YES
1302	Cousinia yasujensis Attar	Asteraceae	Irano-Turanian	Za	Hemicryptophyte	YES

1303	Cousinia zardkuhensis Attar & Ghahr.	Asteraceae	Irano-Turanian	Za	Hemicryptophyte	YES
1304	Crataegus aminii Khat.	Rosaceae	Irano-Turanian	Za	Phanerophyte	YES
1305	Crataegus assadii Khat.	Rosaceae	Irano-Turanian	Ko	Phanerophyte	YES
1306	Crataegus babakhanloui Khat.	Rosaceae	Irano-Turanian	Al, Za	Phanerophyte	NO
1307	Crataegus grossidentata Sharifnia & K.I.Chr.	Rosaceae	Irano-Turanian	Al	Phanerophyte	YES
1308	Crataegus hatamii Hamzehee, K.I.Chr. & Attar	Rosaceae	Irano-Turanian	Za	Phanerophyte	YES
1309	Crataegus khatamsazae Hamzehee, K.I.Chr. & Attar	Rosaceae	Irano-Turanian	Za	Phanerophyte	YES
1310	Crataegus persica Pojark.	Rosaceae	Irano-Turanian	Za	Phanerophyte	YES
1311	Crataegus zagrica Khat.	Rosaceae	Irano-Turanian	Za	Phanerophyte	YES
1312	Crataegus zarrei Dönmez	Rosaceae	Irano-Turanian	Za	Phanerophyte	YES
1313	Crepis alfredii Bornm.	Asteraceae	Euro-Siberian		Hemicryptophyte	YES
1314	Crepis asadbarensis Bornm. ex Rech.f.	Asteraceae	Irano-Turanian	Al	Hemicryptophyte	NO
1315	Crepis ciliata K.Koch	Asteraceae	Irano-Turanian	Al	Hemicryptophyte	YES
1316	Crepis connexa Babc.	Asteraceae	Irano-Turanian	Za	Hemicryptophyte	YES
1317	Crepis demavendi Bornm.	Asteraceae	Irano-Turanian	Al	Hemicryptophyte	YES
1318	Crepis elbrusensis Boiss.	Asteraceae	Irano-Turanian	Az, Al	Hemicryptophyte	NO
1319	Crepis elymaitica Bornm.	Asteraceae	Irano-Turanian	Za	Hemicryptophyte	NO
1320	Crepis frigida (Boiss. & Balansa) Babc.	Asteraceae	Irano-Turanian	Al	Hemicryptophyte	YES
1321	Crepis gaubae Bornm.	Asteraceae	Irano-Turanian	Al	Hemicryptophyte	YES
1322	Crepis heterotricha DC.	Asteraceae	Irano-Turanian	Al, Za, Ke	Hemicryptophyte	NO
1323	Crepis khorassanica Boiss.	Asteraceae	Irano-Turanian	Ko	Hemicryptophyte	YES
1324	Crepis papposissima Babc.	Asteraceae	Irano-Turanian	Al	Therophyte	YES
1325	Crepis quercifolia Bornm. & Gauba	Asteraceae	Irano-Turanian	Al, Az, Za	Therophyte	NO
1326	Crepis sahendi Boiss. & Buhse	Asteraceae	Irano-Turanian	Az	Hemicryptophyte	YES
1327	Crepis semnanensis Heidarnia & Assadi	Asteraceae	Irano-Turanian	Al	Hemicryptophyte	YES
1328	Crepis straussii Bornm.	Asteraceae	Irano-Turanian	Za	Hemicryptophyte	NO
1329	Crepis willemetioides Boiss.	Asteraceae	Euro-Siberian	Al	Hemicryptophyte	NO
1330	Crocus almehensis C.D.Brickell & B.Mathew	Iridaceae	Irano-Turanian		Geophyte	YES
1331	Crocus gilanicus B.Mathew	Iridaceae	Euro-Siberian		Geophyte	YES
1332	Crocus gunae Rukšāns	Iridaceae	Euro-Siberian	Al	Geophyte	YES
1333	Crocus iranicus Rukšāns	Iridaceae	Irano-Turanian	Az	Geophyte	YES

1334	Crocus reinhardii Rukšāns	Iridaceae	Irano-Turanian	Az	Geophyte	YES
1335	Crotalaria assadii Zaeifi	Fabaceae	Saharo-Sindian		Hemicryptophyte	YES
1336	Crucianella gilanica Trin.	Rubiaceae	Irano-Turanian	Al, Za	Chamaephyte	NO
1337	Crucianella platyphylla Ehrend. & Schönb.-Tem.	Rubiaceae	Euro-Siberian	Al	Chamaephyte	YES
1338	Cuscuta haussknechtii Yunck.	Cuscutaceae	Irano-Turanian		Therophyte	YES
1339	Cyanus ouramanicus Ranjbar & Negaresh	Asteraceae	Irano-Turanian	Za	Hemicryptophyte	YES
1340	Cyanus persicus Ranjbar & Negaresh	Asteraceae	Irano-Turanian		Therophyte	YES
1341	Cyanus tabrizianus Ranjbar & Negaresh	Asteraceae	Irano-Turanian	Az	Hemicryptophyte	YES
1342	Cyclotrichium depauperatum (Bunge) Manden. & Scheng.	Lamiaceae	Irano-Turanian	Za	Chamaephyte	NO
1343	Cyclotrichium haussknechtii (Bunge) Manden. & Scheng.	Lamiaceae	Irano-Turanian	Za	Chamaephyte	YES
1344	Cyclotrichium straussii (Bornm.) Rech.f.	Lamiaceae	Irano-Turanian	Za	Chamaephyte	NO
1345	Cymbalaria bakhtiarica Podlech & Iranshahr	Scrophulariaceae	Irano-Turanian	Za	Therophyte	YES
1346	Cynoglossum semnanicum Khat.	Boraginaceae	Irano-Turanian	Al	Hemicryptophyte	YES
1347	Cyperus celans Kukkonen	Cyperaceae	Saharo-Sindian		Hemicryptophyte	YES
1348	Daucus littoralis Sm.	Apiaceae	Euro-Siberian, Saharo-Sindian		Hemicryptophyte	NO
1349	Delphinium aquilegifolium (Boiss.) Bornm.	Ranunculaceae	Irano-Turanian	Al, Ko	Hemicryptophyte	NO
1350	Delphinium elbursense Rech.f.	Ranunculaceae	Irano-Turanian	Az, Al	Hemicryptophyte	NO
1351	Delphinium jacobsii Iranshahr	Ranunculaceae	Irano-Turanian	Za	Hemicryptophyte	YES
1352	Delphinium lalesaricum Iranshahr	Ranunculaceae	Irano-Turanian	Ke	Hemicryptophyte	YES
1353	Delphinium lanigerum Boiss.	Ranunculaceae	Irano-Turanian	Al, Za, Az	Hemicryptophyte	NO
1354	Delphinium saniculifolium Boiss.	Ranunculaceae	Irano-Turanian, Saharo-Sindian	Al, Za, Ke	Hemicryptophyte	NO
1355	Delphinium tuberosum Aucher ex Boiss.	Ranunculaceae	Irano-Turanian	Al, Za, Az	Hemicryptophyte	NO
1356	Delphinium ursinum Rech.f.	Ranunculaceae	Irano-Turanian	Al	Hemicryptophyte	NO
1357	Demavendia pastinacifolia (Boiss. & Hausskn.) Pimenov	Apiaceae	Irano-Turanian	Al	Hemicryptophyte	NO
1358	Deyeuxia parsana Bor	Poaceae	Irano-Turanian	Al	Hemicryptophyte	YES
1359	Dianthus agrostolepis Rech.f.	Caryophyllaceae	Euro-Siberian		Chamaephyte	YES
1360	Dianthus austroiranicus Lemperg	Caryophyllaceae	Irano-Turanian	Za	Chamaephyte	NO
1361	Dianthus binaludensis Rech.f.	Caryophyllaceae	Irano-Turanian	Ko	Chamaephyte	YES
1362	Dianthus denaicus Assadi	Caryophyllaceae	Irano-Turanian	Za	Chamaephyte	YES
1363	Dianthus diversifolius Assadi	Caryophyllaceae	Irano-Turanian	Za	Chamaephyte	YES

1364	Dianthus elymaiticus Hausskn. & Bornm.	Caryophyllaceae	Irano-Turanian	Za	Chamaephyte	YES
1365	Dianthus erythrocoleus Boiss.	Caryophyllaceae	Irano-Turanian	Al	Hemicryptophyte	NO
1366	Dianthus hafezii Assadi	Caryophyllaceae	Irano-Turanian	Za	Chamaephyte	YES
1367	Dianthus hyrcanicus Rech.f.	Caryophyllaceae	Euro-Siberian	Al	Chamaephyte	YES
1368	Dianthus macranthoides Hausskn. ex Bornm.	Caryophyllaceae	Irano-Turanian	Za, Ke	Chamaephyte	NO
1369	Dianthus mazanderanicus Rech.f.	Caryophyllaceae	Euro-Siberian	Al	Chamaephyte	YES
1370	Dianthus pseudocrinitus Behrooz. & Joharchi	Caryophyllaceae	Irano-Turanian	Ko	Hemicryptophyte	YES
1371	Dianthus rudbaricus Assadi	Caryophyllaceae	Euro-Siberian		Chamaephyte	YES
1372	Dianthus sahandicus Assadi	Caryophyllaceae	Irano-Turanian	Az	Chamaephyte	YES
1373	Dianthus seidlitzii Boiss.	Caryophyllaceae	Irano-Turanian	Az	Chamaephyte	YES
1374	Dianthus stapfii Lemperg	Caryophyllaceae	Irano-Turanian	Za	Chamaephyte	YES
1375	Dianthus stenocephalus Boiss.	Caryophyllaceae	Irano-Turanian	Za	Chamaephyte	NO
1376	Dianthus subaphyllus (Lemperg) Rech.f.	Caryophyllaceae	Irano-Turanian, Saharo-Sindian	Za	Chamaephyte	NO
1377	Dianthus szowitisianus Boiss.	Caryophyllaceae	Irano-Turanian	Al, Az, Za	Chamaephyte	NO
1378	Dianthus tabrisianus Bien. ex Boiss.	Caryophyllaceae	Irano-Turanian	Az, Za	Chamaephyte	NO
1379	Diaphanoptera khorasanica Rech.f.	Caryophyllaceae	Irano-Turanian	Ko	Chamaephyte	YES
1380	Diaphanoptera stenocalycina Rech.f. & Schiman-Czeika	Caryophyllaceae	Euro-Siberian	Al	Chamaephyte	YES
1381	Diceratella canescens (Boiss.) Boiss.	Brassicaceae	Saharo-Sindian		Chamaephyte	NO
1382	Dicyclophora persica Boiss.	Apiaceae	Saharo-Sindian		Therophyte	NO
1383	Didymophysa aucheri Boiss.	Brassicaceae	Irano-Turanian	Al, Az, Za, Ko	Hemicryptophyte	NO
1384	Dielsiocharis kotschyi (Boiss.) O.E.Schulz	Brassicaceae	Irano-Turanian	Al, Za, Ko, Ke	Hemicryptophyte	NO
1385	Dionysia archibaldii Wendelbo	Primulaceae	Irano-Turanian	Za	Chamaephyte	YES
1386	Dionysia aretioides (Lehm.) Boiss.	Primulaceae	Irano-Turanian	Al	Chamaephyte	NO
1387	Dionysia assadii Borjian	Primulaceae	Irano-Turanian	Za	Hemicryptophyte	YES
1388	Dionysia aubrietioides Jamzad & Mozaff.	Primulaceae	Irano-Turanian	Za	Chamaephyte	YES
1389	Dionysia bazoftica Jamzad	Primulaceae	Irano-Turanian	Za	Chamaephyte	YES
1390	Dionysia bryoides Boiss.	Primulaceae	Irano-Turanian	Za	Chamaephyte	NO
1391	Dionysia caespitosa Boiss.	Primulaceae	Irano-Turanian	Za	Chamaephyte	NO
1392	Dionysia cristagalli Lidén	Primulaceae	Irano-Turanian	Za	Hemicryptophyte	YES
1393	Dionysia curviflora Bunge	Primulaceae	Irano-Turanian	Za, Ke	Chamaephyte	YES

1394	Dionysia diapensiifolia Boiss.	Primulaceae	Irano-Turanian	Za	Chamaephyte	NO
1395	Dionysia esfandiarii Wendelbo	Primulaceae	Irano-Turanian	Za	Chamaephyte	YES
1396	Dionysia gaubae Bornm.	Primulaceae	Irano-Turanian	Za	Chamaephyte	YES
1397	Dionysia haussknechtii Bornm. & Strauss	Primulaceae	Irano-Turanian	Za	Chamaephyte	NO
1398	Dionysia iranica Jamzad	Primulaceae	Irano-Turanian	Za	Chamaephyte	YES
1399	Dionysia iranshahrii Wendelbo	Primulaceae	Irano-Turanian	Za	Chamaephyte	YES
1400	Dionysia janthina Bornm.	Primulaceae	Irano-Turanian	Za	Chamaephyte	YES
1401	Dionysia khatamii Mozaff.	Primulaceae	Irano-Turanian	Ke	Hemicryptophyte	YES
1402	Dionysia khuzistanica Jamzad	Primulaceae	Irano-Turanian	Za	Chamaephyte	YES
1403	Dionysia lamingtonii Stapf	Primulaceae	Irano-Turanian	Za	Chamaephyte	YES
1404	Dionysia leucotricha Bornm.	Primulaceae	Irano-Turanian	Za	Chamaephyte	NO
1405	Dionysia lurorum Wendelbo	Primulaceae	Irano-Turanian	Za	Chamaephyte	YES
1406	Dionysia michauxii (Duby) Boiss.	Primulaceae	Irano-Turanian	Za	Chamaephyte	YES
1407	Dionysia mozaffarianii Lidén	Primulaceae	Irano-Turanian	Za	Chamaephyte	YES
1408	Dionysia oreodoxa Bornm.	Primulaceae	Irano-Turanian	Ke	Chamaephyte	YES
1409	Dionysia revoluta Boiss.	Primulaceae	Irano-Turanian	Za, Ke	Chamaephyte	NO
1410	Dionysia rhaptodes Bunge	Primulaceae	Irano-Turanian	Ke	Chamaephyte	NO
1411	Dionysia robusta Younesi	Primulaceae	Irano-Turanian	Za	Hemicryptophyte	YES
1412	Dionysia sarvestanica Jamzad & Grey-Wilson	Primulaceae	Irano-Turanian	Za	Chamaephyte	YES
1413	Dionysia sawyeri (Watt) Wendelbo	Primulaceae	Irano-Turanian	Za	Chamaephyte	YES
1414	Dionysia tacamahaca Lidén	Primulaceae	Irano-Turanian	Za	Hemicryptophyte	YES
1415	Dionysia termeana Wendelbo	Primulaceae	Irano-Turanian	Za	Chamaephyte	YES
1416	Dionysia viva Lidén & Zetterl.	Primulaceae	Irano-Turanian	Za	Hemicryptophyte	YES
1417	Dionysia zagrica Grey-Wilson	Primulaceae	Irano-Turanian	Za	Chamaephyte	YES
1418	Dionysia zetterlundii Lidén	Primulaceae	Irano-Turanian	Za	Hemicryptophyte	YES
1419	Dionysia zschummelii Lidén	Primulaceae	Irano-Turanian	Za	Hemicryptophyte	YES
1420	Diplotaenia cachrydifolia Boiss.	Apiaceae	Irano-Turanian	Az, Al	Hemicryptophyte	NO
1421	Diplotaenia damavandica Mozaff., Hedge & Lamond	Apiaceae	Irano-Turanian	Al	Hemicryptophyte	NO
1422	Dolichorrhiza persica (Boiss.) B.Nord.	Asteraceae	Irano-Turanian	Al	Hemicryptophyte	YES
1423	Dorema ammoniacum D.Don	Apiaceae	Irano-Turanian	Al, Za, Ke	Hemicryptophyte	NO
1424	Dorema aucheri Boiss.	Apiaceae	Irano-Turanian	Za, Ke	Hemicryptophyte	NO

1425	Doronicum bracteatum Edmondson	Asteraceae	Irano-Turanian	Az, Za	Hemicryptophyte	YES
1426	Doronicum wendelboi Edmondson	Asteraceae	Irano-Turanian, Euro-Siberian	Al	Hemicryptophyte	YES
1427	Draba pulchella Willd. ex DC.	Brassicaceae	Irano-Turanian	Az, Al	Hemicryptophyte	NO
1428	Dracocephalum aucheri Boiss.	Lamiaceae	Irano-Turanian	Az, Al	Hemicryptophyte	NO
1429	Dracocephalum ghahremanii Jamzad	Lamiaceae	Irano-Turanian	Al	Chamaephyte	YES
1430	Dracocephalum kotschyi Boiss.	Lamiaceae	Irano-Turanian	Al, Za	Chamaephyte	NO
1431	Dracocephalum oligadenium Bornm. & Gauba	Lamiaceae	Irano-Turanian	Al	Chamaephyte	YES
1432	Dracocephalum polychaetum Bornm.	Lamiaceae	Irano-Turanian	Ke	Chamaephyte	NO
1433	Dracocephalum surmandinum Rech.f.	Lamiaceae	Irano-Turanian	Za	Chamaephyte	NO
1434	Ducrosia assadii Alava	Apiaceae	Irano-Turanian	Za	Hemicryptophyte	NO
1435	Echinophora cinerea (Boiss.) Hedge & Lamond	Apiaceae	Irano-Turanian	Za	Hemicryptophyte	NO
1436	Echinophora platyloba DC.	Apiaceae	Irano-Turanian	Al, Za, Ko, Az	Hemicryptophyte	NO
1437	Echinops abazariae Mozaff.	Asteraceae	Irano-Turanian		Hemicryptophyte	YES
1438	Echinops arachniolepis Rech.f.	Asteraceae	Irano-Turanian	Ko	Hemicryptophyte	YES
1439	Echinops aucheri Boiss.	Asteraceae	Irano-Turanian	Ke	Hemicryptophyte	YES
1440	Echinops austro-iranicus Mozaff.	Asteraceae	Saharo-Sindian		Hemicryptophyte	NO
1441	Echinops avajensis Mozaff.	Asteraceae	Irano-Turanian	Za	Hemicryptophyte	NO
1442	Echinops bakhtiaricus Rech.f.	Asteraceae	Irano-Turanian, Saharo-Sindian		Hemicryptophyte	YES
1443	Echinops barezicus Montaz. & Mozaff.	Asteraceae	Saharo-Sindian		Hemicryptophyte	YES
1444	Echinops cephalotes DC.	Asteraceae	Irano-Turanian	Al, Za	Hemicryptophyte	NO
1445	Echinops ceratophorus Boiss.	Asteraceae	Irano-Turanian	Za, Ke	Hemicryptophyte	NO
1446	Echinops cervicornis Bornm.	Asteraceae	Irano-Turanian	Ke	Hemicryptophyte	YES
1447	Echinops chorassanicus Bunge	Asteraceae	Irano-Turanian	Al, Ko	Hemicryptophyte	NO
1448	Echinops cyanocephalus Boiss. & Hausskn.	Asteraceae	Irano-Turanian	Za	Hemicryptophyte	YES
1449	Echinops delicatus Mozaff.	Asteraceae	Saharo-Sindian		Therophyte	YES
1450	Echinops dichrous Boiss. & Hausskn.	Asteraceae	Saharo-Sindian		Hemicryptophyte	NO
1451	Echinops disfulensis Bornm.	Asteraceae	Saharo-Sindian		Hemicryptophyte	YES
1452	Echinops ecbatanus Bornm.	Asteraceae	Irano-Turanian	Za, Az, Al	Hemicryptophyte	NO
1453	Echinops elbursensis Rech.f.	Asteraceae	Irano-Turanian	Al	Hemicryptophyte	NO
1454	Echinops elymaiticus Bornm.	Asteraceae	Irano-Turanian	Za	Hemicryptophyte	NO

1455	Echinops endotrichus Rech.f.	Asteraceae	Irano-Turanian	Za	Hemicryptophyte	NO
1456	Echinops erioceras Bornm.	Asteraceae	Irano-Turanian	Za	Hemicryptophyte	NO
1457	Echinops eriophorus Steven ex DC.	Asteraceae	Irano-Turanian	Za	Hemicryptophyte	YES
1458	Echinops farsicus Rech.f.	Asteraceae	Irano-Turanian		Hemicryptophyte	NO
1459	Echinops gedrosiacus Bornm.	Asteraceae	Saharo-Sindian		Hemicryptophyte	NO
1460	Echinops glanduloso-punctatus Rech.f.	Asteraceae	Irano-Turanian	Ke	Hemicryptophyte	YES
1461	Echinops haussknechtii Boiss.	Asteraceae	Irano-Turanian	Za, Az	Hemicryptophyte	NO
1462	Echinops hebelepis DC.	Asteraceae	Irano-Turanian	Za	Hemicryptophyte	YES
1463	Echinops heteromorphus Bunge	Asteraceae	Irano-Turanian	Ko	Hemicryptophyte	NO
1464	Echinops ilicifolius Bunge	Asteraceae	Irano-Turanian	Ke	Hemicryptophyte	NO
1465	Echinops iranshahrii Rech.f.	Asteraceae	Irano-Turanian	Za	Hemicryptophyte	YES
1466	Echinops jesdianus Boiss. & Buhse	Asteraceae	Irano-Turanian	Ke	Hemicryptophyte	YES
1467	Echinops kazerunensis Mozaff.	Asteraceae	Saharo-Sindian		Hemicryptophyte	YES
1468	Echinops keredjensis Rech.f.	Asteraceae	Irano-Turanian	Al, Za	Hemicryptophyte	NO
1469	Echinops kermanshahanicus Mozaff.	Asteraceae	Irano-Turanian	Za	Hemicryptophyte	NO
1470	Echinops khansaricus Mozaff.	Asteraceae	Irano-Turanian	Za	Hemicryptophyte	NO
1471	Echinops khuzistanicus Mozaff.	Asteraceae	Irano-Turanian	Za	Hemicryptophyte	NO
1472	Echinops koelzii Rech.f.	Asteraceae	Irano-Turanian, Euro-Siberian	Az, Za	Hemicryptophyte	NO
1473	Echinops kotschyi Boiss.	Asteraceae	Irano-Turanian	Za	Hemicryptophyte	YES
1474	Echinops kurdicus Boiss. & Hausskh.	Asteraceae	Irano-Turanian	Za	Hemicryptophyte	NO
1475	Echinops lalesarensis Bornm.	Asteraceae	Irano-Turanian	Ke	Hemicryptophyte	NO
1476	Echinops laricus Mozaff.	Asteraceae	Saharo-Sindian		Hemicryptophyte	YES
1477	Echinops lasiolepis Bunge	Asteraceae	Irano-Turanian	Ke	Hemicryptophyte	NO
1478	Echinops leiopolyceroides Mozaff.	Asteraceae	Irano-Turanian	Ke, Al	Hemicryptophyte	NO
1479	Echinops longipenicillatus Mozaff. & Ghahr.	Asteraceae	Saharo-Sindian		Hemicryptophyte	NO
1480	Echinops macrophyllus Boiss. & Hausskn.	Asteraceae	Irano-Turanian	Al, Za	Hemicryptophyte	NO
1481	Echinops mosulensis Rech.f.	Asteraceae	Irano-Turanian	Za, Al	Hemicryptophyte	NO
1482	Echinops nizvanus Rech.f.	Asteraceae	Irano-Turanian	Al	Hemicryptophyte	NO
1483	Echinops pabotii Rech.f.	Asteraceae	Irano-Turanian	Ke	Hemicryptophyte	YES
1484	Echinops pachyphyllus Rech.f.	Asteraceae	Irano-Turanian	Za	Hemicryptophyte	YES

1485	Echinops persepolitanus Rech.f.	Asteraceae	Irano-Turanian	Za	Hemicryptophyte	NO
1486	Echinops polychromus Rech.f.	Asteraceae	Saharo-Sindian		Hemicryptophyte	YES
1487	Echinops polygamus Bunge	Asteraceae	Irano-Turanian	Al, Za	Hemicryptophyte	NO
1488	Echinops procerus Mozaff.	Asteraceae	Irano-Turanian	Ko	Hemicryptophyte	YES
1489	Echinops psammophilus Mozaff.	Asteraceae	Saharo-Sindian		Hemicryptophyte	NO
1490	Echinops quercetorum Mozaff.	Asteraceae	Irano-Turanian	Za	Hemicryptophyte	YES
1491	Echinops robustus Bunge	Asteraceae	Irano-Turanian	Az,Za, Al	Hemicryptophyte	NO
1492	Echinops sabzevarensis Mozaff.	Asteraceae	Irano-Turanian	Al, Ko, Za	Hemicryptophyte	NO
1493	Echinops shahrudensis Mozaff. & Ghahr.	Asteraceae	Irano-Turanian	Al	Hemicryptophyte	YES
1494	Echinops shulabadensis Mozaff.	Asteraceae	Irano-Turanian	Al, Za	Hemicryptophyte	YES
1495	Echinops sojakii Rech.f.	Asteraceae	Irano-Turanian	Za	Hemicryptophyte	YES
1496	Echinops taftanicus Mozaff. & Ghahr.	Asteraceae	Saharo-Sindian		Hemicryptophyte	YES
1497	Echinops tenuisectus Rech.f.	Asteraceae	Irano-Turanian	Ke, Za	Hemicryptophyte	NO
1498	Echinops viscidulus Mozaff.	Asteraceae	Irano-Turanian	Za	Hemicryptophyte	NO
1499	Echium khuzistanicum Mozaff.	Boraginaceae	Saharo-Sindian		Hemicryptophyte	YES
1500	Elaeosticta nodosa (Boiss.) Boiss.	Apiaceae	Irano-Turanian	Za	Hemicryptophyte	NO
1501	Elburzia fenestrata (Boiss.) Hedge	Brassicaceae	Irano-Turanian	Al	Chamaephyte	NO
1502	Elymus zagricus Assadi	Poaceae	Irano-Turanian	Za	Hemicryptophyte	YES
1503	Eminium jaegeri Bogner & P.C.Boyce	Araceae	Irano-Turanian	Za	Geophyte	YES
1504	Ephedra laristanica Assadi	Ephedraceae	Saharo-Sindian		Phanerophyte	NO
1505	Epilobium rechingeri P.H.Raven	Onagraceae	Irano-Turanian	Al	Hemicryptophyte	YES
1506	Epipactis rechingeri Renz	Orchidaceae	Euro-Siberian		Geophyte	NO
1507	Eremostachys lanata Jamzad	Lamiaceae	Euro-Siberian		Hemicryptophyte	YES
1508	Ergocarpon cryptanthum (Rech.f.) C.C.Towns.	Apiaceae	Saharo-Sindian		Therophyte	NO
1509	Erigeron hyrcanicus Bornm. & Vierh.	Asteraceae	Irano-Turanian	Al	Hemicryptophyte	NO
1510	Eriocycla ghafooriana Akhani	Apiaceae	Euro-Siberian	Al	Chamaephyte	YES
1511	Eriocycla olivieri (Boiss.) H.Wolff	Apiaceae	Irano-Turanian, Euro-Siberian	Al	Chamaephyte	NO
1512	Eritrichium gracillimum Rech.f.	Boraginaceae	Irano-Turanian	Al	Hemicryptophyte	YES
1513	Erodium dimorphum Wendelbo	Geraniaceae	Irano-Turanian	Al	Hemicryptophyte	YES
1514	Eryngium iranicum Mozaff.	Apiaceae	Irano-Turanian	Az	Hemicryptophyte	YES

1515	Erysimum caespitosum DC.	Brassicaceae	Irano-Turanian	Az, Al, Za	Hemicryptophyte	NO
1516	Erysimum elbrusense Boiss.	Brassicaceae	Irano-Turanian	Az, Za, Al, Ko	Hemicryptophyte	NO
1517	Erysimum elymaiticum Mozaff.	Brassicaceae	Saharo-Sindian		Chamaephyte	YES
1518	Erysimum frigidum Boiss. & Hausskn.	Brassicaceae	Irano-Turanian	Za	Hemicryptophyte	YES
1519	Erysimum gelidum Bunge	Brassicaceae	Irano-Turanian	Az	Hemicryptophyte	YES
1520	Erysimum hezarense Moazzeni	Brassicaceae	Irano-Turanian	Ke	Hemicryptophyte	YES
1521	Erysimum koelzii Polatschek & Rech.f.	Brassicaceae	Irano-Turanian	Za	Hemicryptophyte	YES
1522	Erysimum nanum Boiss. & Hohen.	Brassicaceae	Irano-Turanian	Az, Al	Hemicryptophyte	YES
1523	Erysimum nasturtioides Boiss. & Hausskn.	Brassicaceae	Irano-Turanian	Az, Za	Hemicryptophyte	YES
1524	Erysimum polatschekii Moazzeni, Assadi & Al-Shehbaz	Brassicaceae	Irano-Turanian	Ke	Hemicryptophyte	YES
1525	Euphorbia acanthodes Akhani	Euphorbiaceae	Saharo-Sindian		Chamaephyte	YES
1526	Euphorbia aellenii Rech.f.	Euphorbiaceae	Irano-Turanian	Ko	Hemicryptophyte	YES
1527	Euphorbia connata Boiss.	Euphorbiaceae	Irano-Turanian	Ke	Hemicryptophyte	NO
1528	Euphorbia decipiens Boiss. & Buhse	Euphorbiaceae	Irano-Turanian	Az, Za, Ke	Hemicryptophyte	NO
1529	Euphorbia elwendica Stapf	Euphorbiaceae	Irano-Turanian	Za	Hemicryptophyte	YES
1530	Euphorbia erythradenia Boiss.	Euphorbiaceae	Irano-Turanian	Za, Ke	Hemicryptophyte	NO
1531	Euphorbia ferdowsiana Pahlevani	Euphorbiaceae	Irano-Turanian	Ko	Hemicryptophyte	YES
1532	Euphorbia gorenflotii Mobayen	Euphorbiaceae	Irano-Turanian	Al	Hemicryptophyte	YES
1533	Euphorbia gypsicola Rech.f. & Aellen	Euphorbiaceae	Irano-Turanian	Al	Hemicryptophyte	YES
1534	Euphorbia halophila Bronm. & Gauba	Euphorbiaceae	Irano-Turanian		Hemicryptophyte	YES
1535	Euphorbia hebecarpa Boiss.	Euphorbiaceae	Irano-Turanian	Az, Za, Ke	Hemicryptophyte	NO
1536	Euphorbia iranshahrii Pahlevani	Euphorbiaceae	Irano-Turanian	Za	Hemicryptophyte	NO
1537	Euphorbia khorasanica Saeidi & Ghayorm.	Euphorbiaceae	Irano-Turanian	Ko	Hemicryptophyte	YES
1538	Euphorbia malleata Boiss.	Euphorbiaceae	Irano-Turanian	Za	Hemicryptophyte	YES
1539	Euphorbia mazandaranica Pahlevani	Euphorbiaceae	Euro-Siberian		Hemicryptophyte	NO
1540	Euphorbia plebeia Boiss.	Euphorbiaceae	Irano-Turanian	Za	Hemicryptophyte	YES
1541	Euphorbia sahendi Bornm.	Euphorbiaceae	Irano-Turanian	Az	Hemicryptophyte	YES
1542	Euphorbia spartiformis Mobayen	Euphorbiaceae	Saharo-Sindian		Hemicryptophyte	YES
1543	Euphorbia sulphurea Pahlevani	Euphorbiaceae	Saharo-Sindian		Hemicryptophyte	YES
1544	Euphorbia teheranica Boiss.	Euphorbiaceae	Irano-Turanian	Al, Za	Hemicryptophyte	NO
1545	Euphrasia juzepczukii Deniss.	Scrophulariaceae	Irano-Turanian	Az, Al	Therophyte	NO

1546	Farsetia assadii Kavousi	Scrophulariaceae	Saharo-Sindian		Chamaephyte	YES
1547	Ferula alliacea Boiss.	Apiaceae	Irano-Turanian	Ko	Hemicryptophyte	YES
1548	Ferula behboudiana (Rech.f. & Esfand.) D.F.Chamb.	Apiaceae	Saharo-Sindian		Hemicryptophyte	NO
1549	Ferula flabelliloba Rech.f. & Aellen	Apiaceae	Irano-Turanian	Ko, Za	Hemicryptophyte	YES
1550	Ferula gabrielii Rech.f.	Apiaceae	Irano-Turanian	Ko, Lo	Hemicryptophyte	YES
1551	Ferula hezarlalehzarica Ajani	Apiaceae	Irano-Turanian	Ke	Hemicryptophyte	YES
1552	Ferula hirtella Boiss.	Apiaceae	Irano-Turanian	Al, Za, Ke, Lo	Hemicryptophyte	NO
1553	Ferula kashanica Rech.f.	Apiaceae	Irano-Turanian	Za	Hemicryptophyte	NO
1554	Ferula lutensis Rech.f.	Apiaceae	Irano-Turanian, Saharo-Sindian	Ko, Lo	Hemicryptophyte	NO
1555	Ferula macrocolea Boiss.	Apiaceae	Irano-Turanian	Za, Al	Hemicryptophyte	NO
1556	Ferula microcolea (Boiss.) Boiss.	Apiaceae	Irano-Turanian	Az, Za, Al	Hemicryptophyte	NO
1557	Ferula persica Willd.	Apiaceae	Irano-Turanian	Al	Hemicryptophyte	NO
1558	Ferula pseudalliacea Rech.f.	Apiaceae	Irano-Turanian	Za	Hemicryptophyte	NO
1559	Ferula serpentinica Rech.f.	Apiaceae	Irano-Turanian	Al	Hemicryptophyte	YES
1560	Ferula sharifii Rech.f. & Esfand.	Apiaceae	Irano-Turanian, Saharo-Sindian	Ke, Lo	Hemicryptophyte	YES
1561	Ferula stenocarpa Boiss. & Hausskn.	Apiaceae	Saharo-Sindian		Hemicryptophyte	NO
1562	Ferula tabasensis Rech.f.	Apiaceae	Irano-Turanian	Ko, Lo	Hemicryptophyte	NO
1563	Ferula xylorhachis Rech.f.	Apiaceae	Irano-Turanian	Al, Za, Ko	Hemicryptophyte	NO
1564	Ferulago angulata (Schltdl.) Boiss.	Apiaceae	Irano-Turanian	Az, Za, Al, Ko	Hemicryptophyte	NO
1565	Ferulago carduchorum Boiss. & Hausskn. ex Boiss.	Apiaceae	Irano-Turanian	Az, Za, Ke	Hemicryptophyte	NO
1566	Ferulago contracta Boiss. & Hausskn.	Apiaceae	Irano-Turanian	Za, Ke	Hemicryptophyte	NO
1567	Ferulago phialocarpa Rech.f. & Riedl	Apiaceae	Irano-Turanian	Za	Hemicryptophyte	YES
1568	Ferulago trifida Boiss.	Apiaceae	Irano-Turanian	Al	Hemicryptophyte	YES
1569	Festuca akhanii Tzvelev	Poaceae	Irano-Turanian		Hemicryptophyte	YES
1570	Festuca iranica E.B.Alexeev	Poaceae	Irano-Turanian	Za	Hemicryptophyte	YES
1571	Festuca rechingeri E.B.Alexeev	Poaceae	Euro-Siberian		Hemicryptophyte	YES
1572	Festuca sabalanica E.B.Alexeev	Poaceae	Irano-Turanian	Az	Hemicryptophyte	YES
1573	Fibigia multicaulis (Boiss. & Hohen.) Boiss.	Brassicaceae	Irano-Turanian	Al, Za, Ke	Hemicryptophyte	NO
1574	Fibigia umbellata (Boiss.) Boiss.	Brassicaceae	Irano-Turanian	Al, Za, Ke	Hemicryptophyte	NO
1575	Fortuynia garcinii (Burm.f.) Shuttlew.	Brassicaceae	Irano-Turanian,		Chamaephyte	NO

			Saharo-Sindian			
1576	Fritillaria avromanica Advay & Tekşen	Liliaceae	Irano-Turanian	Za	Geophyte	YES
1577	Fritillaria chlorantha Hausskn. & Bornm.	Liliaceae	Irano-Turanian	Za	Geophyte	NO
1578	Fritillaria kotschyana Herb.	Liliaceae	Irano-Turanian	Az, Al	Geophyte	NO
1579	Fritillaria olivieri Baker	Liliaceae	Irano-Turanian	Az, Za	Geophyte	NO
1580	Fritillaria straussii Bornm.	Liliaceae	Irano-Turanian	Za	Geophyte	NO
1581	Fritillaria zagrica Stapf	Liliaceae	Irano-Turanian	Az, Za	Geophyte	NO
1582	Gagea calcicola Zarrei & Wilkin	Liliaceae	Irano-Turanian	Za	Geophyte	NO
1583	Gagea iranica Zarrei & Zarre	Liliaceae	Irano-Turanian	Al, Ko	Geophyte	NO
1584	Gagea robusta Zarrei & Wilkin	Liliaceae	Irano-Turanian	Al	Geophyte	YES
1585	Gagea uliginosa Siehe & Pascher	Liliaceae	Irano-Turanian	Al, Az	Geophyte	NO
1586	Gagea wendelboi Rech.f.	Liliaceae	Irano-Turanian	Al	Geophyte	YES
1587	Gaillonia dezfulensis Naanaie & Assadi	Rubiaceae	Saharo-Sindian		Chamaephyte	YES
1588	Gaillonia eriantha Jaub. & Spach	Rubiaceae	Irano-Turanian	Ke, Za	Chamaephyte	NO
1589	Galium aucheri Boiss.	Rubiaceae	Irano-Turanian	Al	Hemicryptophyte	NO
1590	Galium decumbens (Ehrend.) Ehrend. & Schönb.-Tem.	Rubiaceae	Irano-Turanian	Az, Al	Chamaephyte	NO
1591	Galium delicatulum Boiss. & Hohen.	Rubiaceae	Irano-Turanian	Al	Chamaephyte	YES
1592	Galium diploprion Boiss. & Hohen.	Rubiaceae	Irano-Turanian	Al	Hemicryptophyte	NO
1593	Galium elbursense Bornm. & Gauba	Rubiaceae	Irano-Turanian	Al	Chamaephyte	YES
1594	Galium iranicum Hausskn. ex Bornm.	Rubiaceae	Irano-Turanian	Za	Hemicryptophyte	YES
1595	Galium problematicum (Ehrend.) Ehrend. & Schönb.-Tem.	Rubiaceae	Irano-Turanian	Al	Chamaephyte	YES
1596	Galium pseudokurdicum (Ehrend.) Schönb.-Tem.	Rubiaceae	Irano-Turanian	Za, Az	Hemicryptophyte	NO
1597	Galium schoenbeck-Temesyae Ehrend.	Rubiaceae	Irano-Turanian	Za	Chamaephyte	YES
1598	Galium sojakii Ehrend. & Schönb.-Tem.	Rubiaceae	Irano-Turanian	Za	Therophyte	YES
1599	Galium tehranicum Moussavi, Ghahr. & Attar	Rubiaceae	Irano-Turanian	Al	Hemicryptophyte	YES
1600	Geranium persicum Schönb.-Tem.	Geraniaceae	Irano-Turanian	Az, Za, Al	Geophyte	NO
1601	Geum iranicum Khat.	Rosaceae	Irano-Turanian	Ko	Hemicryptophyte	YES
1602	Girgensohnia imbricata Bunge	Chenopodiaceae	Irano-Turanian	Ke, Lo	Therophyte	NO
1603	Gladiolus persicus Boiss.	Iridaceae	Irano-Turanian, Saharo-Sindian	Za, Ke	Geophyte	NO
1604	Glaucium calycinum Boiss.	Papaveraceae	Irano-Turanian	Za	Hemicryptophyte	YES

1605	Glaucium contortuplicatum Boiss.	Papaveraceae	Irano-Turanian	Al	Hemicryptophyte	NO
1606	Glaucium elegantissimum Mobayen	Papaveraceae	Irano-Turanian, Euro-Siberian		Therophyte	YES
1607	Glaucium golestanicum A.Gran & Sharifnia	Papaveraceae	Irano-Turanian		Hemicryptophyte	YES
1608	Glaucium mathiolifolium Mobayen	Papaveraceae	Saharo-Sindian		Hemicryptophyte	YES
1609	Graellsia integrifolia (Rech.f.) Rech.f.	Brassicaceae	Irano-Turanian	Ko	Hemicryptophyte	YES
1610	Graellsia saxifragifolia (DC.) Boiss.	Brassicaceae	Irano-Turanian	Za, Ko, Ke	Hemicryptophyte	NO
1611	Graellsia stylosa (Boiss. & Hohen.) Poulter	Brassicaceae	Irano-Turanian	Al	Hemicryptophyte	NO
1612	Grantia arachnoidea Boiss.	Asteraceae	Saharo-Sindian		Hemicryptophyte	YES
1613	Grantia discoidea Bunge ex Boiss.	Asteraceae	Irano-Turanian		Hemicryptophyte	YES
1614	Gypsophila acantholimoides Bornm.	Caryophyllaceae	Irano-Turanian	Za	Chamaephyte	YES
1615	Gypsophila adenophora Boiss. & Buhse	Caryophyllaceae	Irano-Turanian	Za	Hemicryptophyte	YES
1616	Gypsophila alvandica Falat., F.Ghahrem. & Assadi	Caryophyllaceae	Irano-Turanian	Za	Hemicryptophyte	YES
1617	Gypsophila bazorganica Rech.f.	Caryophyllaceae	Irano-Turanian	Az	Chamaephyte	YES
1618	Gypsophila caricifolia Boiss.	Caryophyllaceae	Irano-Turanian	Az, Za	Hemicryptophyte	NO
1619	Gypsophila elymaitica Mozaff.	Caryophyllaceae	Irano-Turanian, Saharo-Sindian		Hemicryptophyte	YES
1620	Gypsophila iranica Barkoudah	Caryophyllaceae	Irano-Turanian	Az	Therophyte	YES
1621	Gypsophila leioclada Rech.f.	Caryophyllaceae	Irano-Turanian	Za	Hemicryptophyte	YES
1622	Gypsophila lurorum Rech.f.	Caryophyllaceae	Irano-Turanian	Za	Hemicryptophyte	YES
1623	Gypsophila melampoda Bien. ex Boiss.	Caryophyllaceae	Irano-Turanian, Saharo-Sindian	Za	Therophyte	YES
1624	Gypsophila mucronifolia Rech.f.	Caryophyllaceae	Irano-Turanian	Al	Hemicryptophyte	YES
1625	Gypsophila persica Barkoudak	Caryophyllaceae	Irano-Turanian	Al, Za	Hemicryptophyte	NO
1626	Gypsophila platyphylla Boiss.	Caryophyllaceae	Irano-Turanian	Za	Hemicryptophyte	NO
1627	Gypsophila polyclada Fenzl ex Boiss.	Caryophyllaceae	Irano-Turanian	Za	Hemicryptophyte	YES
1628	Gypsophila pseudomelampoda Gauba & Rech.f.	Caryophyllaceae	Irano-Turanian	Al, Za	Hemicryptophyte	NO
1629	Gypsophila pseudopallida Falat., Assadi & F.Ghahrem.	Caryophyllaceae	Irano-Turanian	Az	Hemicryptophyte	YES
1630	Gypsophila rupestris Mozaff.	Caryophyllaceae	Irano-Turanian	Za	Hemicryptophyte	YES
1631	Gypsophila saponarioides Bornm. & Gauba	Caryophyllaceae	Irano-Turanian	Al	Chamaephyte	YES
1632	Gypsophila wilhelminae Rech.f.	Caryophyllaceae	Irano-Turanian	Az	Chamaephyte	YES
1633	Gypsophila xanthochlora Rech.f.	Caryophyllaceae	Irano-Turanian	Al	Hemicryptophyte	NO

1634	Gypsophila yazdiana Falat., F.Ghahrem. & Assadi	Caryophyllaceae	Irano-Turanian	Ke	Hemicryptophyte	YES
1635	Halanthium alaeflavum Assadi	Chenopodiaceae	Irano-Turanian	Az	Therophyte	YES
1636	Halimocnemis azarbaijanensis Assadi	Chenopodiaceae	Irano-Turanian	Az	Therophyte	YES
1637	Halimocnemis mamamensis (Bunge) Assadi	Chenopodiaceae	Irano-Turanian	Az, Al, Za	Therophyte	NO
1638	Halotis pedunculata Assadi	Amaranthaceae	Saharo-Sindian		Therophyte	YES
1639	Haplophyllum bakhteganicum Soltani & Khosravi	Rutaceae	Irano-Turanian	Za	Hemicryptophyte	YES
1640	Haplophyllum buhsei Boiss.	Rutaceae	Irano-Turanian	Al	Chamaephyte	YES
1641	Haplophyllum canaliculatum Boiss.	Rutaceae	Irano-Turanian, Saharo-Sindian	Za, Lo	Hemicryptophyte	NO
1642	Haplophyllum dasygynum C.C.Towns.	Rutaceae	Irano-Turanian	Az, Za	Hemicryptophyte	YES
1643	Haplophyllum furfuraceum Bunge	Rutaceae	Irano-Turanian	Ko, Lo	Hemicryptophyte	NO
1644	Haplophyllum glaberrimum Bunge	Rutaceae	Irano-Turanian		Hemicryptophyte	NO
1645	Haplophyllum laeviusculum C.C.Towns.	Rutaceae	Irano-Turanian	Az, Al	Chamaephyte	NO
1646	Haplophyllum laristanicum C.C.Towns.	Rutaceae	Saharo-Sindian		Hemicryptophyte	YES
1647	Haplophyllum lissonotum C.C.Towns.	Rutaceae	Irano-Turanian	Za	Chamaephyte	NO
1648	Haplophyllum rechingeri C.C.Towns.	Rutaceae	Irano-Turanian	Za	Hemicryptophyte	YES
1649	Haplophyllum rubro-tinctum C.C.Towns.	Rutaceae	Irano-Turanian	Al, Za	Hemicryptophyte	NO
1650	Haplophyllum stapfianum Hand.-Mazz.	Rutaceae	Irano-Turanian	Za	Hemicryptophyte	YES
1651	Haplophyllum virgatum Spach	Rutaceae	Irano-Turanian, Saharo-Sindian		Hemicryptophyte	NO
1652	Haplophyllum viridulum Soják	Rutaceae	Irano-Turanian	Za	Hemicryptophyte	NO
1653	Haussknechtia elymaitica Boiss.	Apiaceae	Irano-Turanian	Za	Hemicryptophyte	NO
1654	Hedysarum al-shehbazii Ranjbar	Fabaceae	Irano-Turanian	Za	Hemicryptophyte	YES
1655	Hedysarum bojnordense Ranjbar & Joharchi	Fabaceae	Irano-Turanian		Hemicryptophyte	YES
1656	Hedysarum callithrix Boiss.	Fabaceae	Irano-Turanian	Al, Ko, Ke	Hemicryptophyte	YES
1657	Hedysarum criniferum Boiss.	Fabaceae	Irano-Turanian	Za, Az	Hemicryptophyte	NO
1658	Hedysarum damghanicum Rech.f.	Fabaceae	Irano-Turanian	Al	Chamaephyte	YES
1659	Hedysarum elbursense Bornm. & Gauba	Fabaceae	Irano-Turanian	Al	Chamaephyte	YES
1660	Hedysarum glabrifoliolatum Ranjbar	Fabaceae	Irano-Turanian	Ko	Chamaephyte	YES
1661	Hedysarum gypsophilum Dehshiri	Fabaceae	Irano-Turanian	Za	Hemicryptophyte	YES
1662	Hedysarum halophilum Bornm. & Gauba	Fabaceae	Irano-Turanian	Al	Chamaephyte	YES
1663	Hedysarum hyrcanum Bornm. & Gauba	Fabaceae	Irano-Turanian	Al	Hemicryptophyte	YES

1664	Hedysarum johartchii Ranjbar	Fabaceae	Irano-Turanian	Ko	Hemicryptophyte	YES
1665	Hedysarum longipedunculatum Ranjbar & Karamian	Fabaceae	Irano-Turanian		Hemicryptophyte	YES
1666	Hedysarum marandense Mozaff.	Fabaceae	Irano-Turanian	Az	Chamaephyte	YES
1667	Hedysarum neyshaboricum Ranjbar	Fabaceae	Irano-Turanian	Ko	Hemicryptophyte	YES
1668	Hedysarum orumiehense Ranjbar	Fabaceae	Irano-Turanian	Az	Hemicryptophyte	YES
1669	Hedysarum papillosum Boiss.	Fabaceae	Irano-Turanian	Az	Hemicryptophyte	YES
1670	Hedysarum paucifoliolatum Ranjbar & Olanj	Fabaceae	Irano-Turanian	Ko	Hemicryptophyte	YES
1671	Hedysarum persicum Bidarlord, F.Ghahrem. & Mozaff.	Fabaceae	Irano-Turanian	Az	Hemicryptophyte	YES
1672	Hedysarum plumosum Boiss. & Hausskn.	Fabaceae	Irano-Turanian	Za	Hemicryptophyte	YES
1673	Hedysarum renzii Rech.f.	Fabaceae	Irano-Turanian		Chamaephyte	YES
1674	Heldreichia longifolia Boiss.	Brassicaceae	Irano-Turanian	Za	Hemicryptophyte	YES
1675	Helianthemum assadii Ghahrem.-Nejad & Gholamian	Cistaceae	Saharo-Sindian		Therophyte	NO
1676	Helianthemum sinuspersicum Gholamian & Ghahrem.-Nejad	Cistaceae	Saharo-Sindian		Chamaephyte	YES
1677	Helichrysum artemisioides Boiss. & Hausskn.	Asteraceae	Irano-Turanian	Za	Chamaephyte	NO
1678	Helichrysum athanaton Georgiadou & Rech.f.	Asteraceae	Irano-Turanian	Za	Chamaephyte	NO
1679	Helichrysum davisianum Rech.f.	Asteraceae	Irano-Turanian	Ke	Chamaephyte	YES
1680	Helichrysum globiferum Boiss.	Asteraceae	Irano-Turanian	Az, Al, Za	Chamaephyte	NO
1681	Helichrysum leucocephalum Boiss.	Asteraceae	Irano-Turanian, Saharo-Sindian	Ke, Lo	Chamaephyte	NO
1682	Helichrysum makranicum (Rech.f. & Esfand.) Rech.f.	Asteraceae	Saharo-Sindian		Hemicryptophyte	YES
1683	Helichrysum oligocephalum DC.	Asteraceae	Irano-Turanian	Za, Al	Chamaephyte	NO
1684	Helichrysum oocephalum Boiss.	Asteraceae	Irano-Turanian	Al, Za, Ko	Hemicryptophyte	NO
1685	Helichrysum persicum Ghahrem.-Nejad & Noori	Asteraceae	Irano-Turanian	Ko	Chamaephyte	YES
1686	Helichrysum psychrophilum Boiss.	Asteraceae	Irano-Turanian	Az, Al	Hemicryptophyte	NO
1687	Heliocarya monandra Bunge	Boraginaceae	Irano-Turanian	Za, Ke	Hemicryptophyte	YES
1688	Heliotropium agdense Bunge	Boraginaceae	Irano-Turanian	Ke	Therophyte	NO
1689	Heliotropium aucheri DC.	Boraginaceae	Irano-Turanian	Ke, Za	Hemicryptophyte	NO
1690	Heliotropium denticulatum Boiss. & Hausskn.	Boraginaceae	Irano-Turanian	Za	Hemicryptophyte	YES
1691	Heliotropium disciforme Akhani	Boraginaceae	Irano-Turanian	Ko	Therophyte	YES
1692	Heliotropium esfahanicum Khat.	Boraginaceae	Irano-Turanian	Za	Therophyte	YES
1693	Heliotropium esfandiarii Akhani & Riedl	Boraginaceae	Irano-Turanian	Al	Therophyte	NO

1694	Heliotropium gypsaceum Rech.f. & Riedl	Boraginaceae	Irano-Turanian	Az	Therophyte	YES
1695	Heliotropium kaserunense Bornm.	Boraginaceae	Saharo-Sindian		Hemicryptophyte	YES
1696	Heliotropium khayyamii Akhani	Boraginaceae	Irano-Turanian	Ko	Therophyte	YES
1697	Heliotropium samoliflorum Bunge	Boraginaceae	Irano-Turanian	Az, Za	Therophyte	NO
1698	Heliotropium shirazicum Mozaff.	Boraginaceae	Irano-Turanian	Za	Hemicryptophyte	YES
1699	Heliotropium ziegleri Akhani	Boraginaceae	Irano-Turanian	Al	Therophyte	YES
1700	Heracleum anisactis Boiss. & Hohen.	Apiaceae	Irano-Turanian	Az, Al	Hemicryptophyte	NO
1701	Heracleum gorganicum Rech.f.	Apiaceae	Euro-Siberian	Al	Hemicryptophyte	YES
1702	Heracleum rawianum C.C.Towns.	Apiaceae	Irano-Turanian	Az	Hemicryptophyte	YES
1703	Heracleum rechingeri Manden.	Apiaceae	Euro-Siberian	Al	Hemicryptophyte	YES
1704	Hertia angustifolia (DC.) Kuntze	Asteraceae	Irano-Turanian	Al, Za, Ke	Chamaephyte	NO
1705	Hesperis borbasii F.Dvořák	Brassicaceae	Irano-Turanian	Za	Hemicryptophyte	YES
1706	Hesperis leucoclada Boiss.	Brassicaceae	Irano-Turanian	Za, Ke	Hemicryptophyte	YES
1707	Hesperis luristanica F.Dvořák	Brassicaceae	Irano-Turanian	Za	Hemicryptophyte	YES
1708	Hesperis nivalis Boiss. & Hausskn.	Brassicaceae	Irano-Turanian	Az, Za	Hemicryptophyte	NO
1709	Heteroderis pusilla (Boiss.) Boiss.	Asteraceae	Irano-Turanian		Therophyte	NO
1710	Hieracium azerbaijanense Lack	Asteraceae	Irano-Turanian	Az	Hemicryptophyte	YES
1711	Hieracium cheirifolium Boiss. & Hausskn. ex Boiss.	Asteraceae	Irano-Turanian	Za	Hemicryptophyte	YES
1712	Hieracium piranshahricum Tavakkoli & Assadi	Asteraceae	Irano-Turanian	Az	Hemicryptophyte	YES
1713	Hyacinthella persica (Boiss. & Buhse) Chouard	Asparagaceae	Euro-Siberian		Geophyte	YES
1714	Hymenocephalus rigidus Jaub. & Spach	Asteraceae	Irano-Turanian	Za	Chamaephyte	YES
1715	Hymenocrater incanus Bunge	Lamiaceae	Irano-Turanian	Za	Chamaephyte	NO
1716	Hymenocrater oxyodontus Rech.f.	Lamiaceae	Irano-Turanian		Chamaephyte	NO
1717	Hymenocrater platystegius Rech.f.	Lamiaceae	Irano-Turanian	Ko	Chamaephyte	NO
1718	Hymenocrater yazdianus Rech.f.	Lamiaceae	Irano-Turanian	Za, Ke	Chamaephyte	NO
1719	Hyoscyamus bornmulleri Khat.	Solanaceae	Irano-Turanian	Za	Hemicryptophyte	YES
1720	Hyoscyamus kotschyanus Pojark.	Solanaceae	Irano-Turanian	Za	Therophyte	NO
1721	Hyoscyamus kurdicus Bornm.	Solanaceae	Irano-Turanian	Az, Al, Za	Hemicryptophyte	YES
1722	Hyoscyamus malekianus Parsa	Solanaceae	Irano-Turanian		Hemicryptophyte	YES
1723	Hyoscyamus tenuicaulis Schönb.-Tem.	Solanaceae	Saharo-Sindian		Hemicryptophyte	NO
1724	Hypericopsis persica Boiss.	Frankeniaceae	Irano-Turanian	Za	Chamaephyte	NO

1725	Hypericum dogonbadanicum Assadi	Hypericaceae	Saharo-Sindian		Chamaephyte	YES
1726	Hypericum fursei N.Robson	Hypericaceae	Irano-Turanian	Al	Hemicryptophyte	YES
1727	Indigofera sinuspersica Mozaff.	Fabaceae	Saharo-Sindian		Therophyte	YES
1728	Inula angustifolia DC.	Asteraceae	Irano-Turanian	?	Hemicryptophyte	YES
1729	Inula persica Ghahrem.-Nejad & Narimisa	Asteraceae	Irano-Turanian	Ke	Hemicryptophyte	YES
1730	Inula rajamandii Narimisa & F.Ghahrem.	Asteraceae	Irano-Turanian	Az	Hemicryptophyte	YES
1731	Iranecio elbrusensis (Boiss.) B.Nord.	Asteraceae	Irano-Turanian	Al	Hemicryptophyte	NO
1732	Iranecio oligolepis (Boiss.) B.Nord.	Asteraceae	Irano-Turanian	Al	Hemicryptophyte	YES
1733	Iranecio paucilobus (DC.) B.Nord.	Asteraceae	Irano-Turanian	Az, Al, Za, Ke	Hemicryptophyte	NO
1734	Iris barnumiae Foster & Baker	Iridaceae	Irano-Turanian	Al	Geophyte	NO
1735	Iris meda Stapf	Iridaceae	Irano-Turanian	Az, Za	Geophyte	NO
1736	Isatis campylocarpa Boiss.	Brassicaceae	Irano-Turanian	Za	Therophyte	NO
1737	Isatis gaubae Bornm.	Brassicaceae	Irano-Turanian	Al	Hemicryptophyte	NO
1738	Isatis koeiei Rech.f.	Brassicaceae	Saharo-Sindian		Therophyte	YES
1739	Isatis pachycarpa Rech. f., Aellen & Esfand.	Brassicaceae	Irano-Turanian		Hemicryptophyte	YES
1740	Isatis raphanifolia Boiss.	Brassicaceae	Irano-Turanian, Saharo-Sindian	Za, Al, Ke	Therophyte	NO
1741	Isatis rugulosa Bunge	Brassicaceae	Irano-Turanian	Za, Ke	Therophyte	NO
1742	Isatis zarrei Al-Shehbaz, Moazzeni & Mumm.	Brassicaceae	Irano-Turanian	Al	Therophyte	YES
1743	Johrenia golestanica Rech.f.	Apiaceae	Euro-Siberian	Al	Hemicryptophyte	YES
1744	Johrenia ramosissima Mozaff.	Apiaceae	Euro-Siberian	Al	Hemicryptophyte	YES
1745	Johreniopsis oligactis (Rech.f. & Riedl) Pimenov	Apiaceae	Irano-Turanian	Za	Hemicryptophyte	YES
1746	Johreniopsis scoparia (Boiss.) Pimenov	Apiaceae	Irano-Turanian	Za	Hemicryptophyte	NO
1747	Johreniopsis stricticaulis (Rech.f.) Pimen.	Apiaceae	Irano-Turanian	Ko	Hemicryptophyte	NO
1748	Jurinea bungei Boiss.	Asteraceae	Irano-Turanian	Za, Ke	Hemicryptophyte	NO
1749	Jurinea cartilaginea Mozaff.	Asteraceae	Saharo-Sindian		Chamaephyte	YES
1750	Jurinea catharinae Iljin	Asteraceae	Irano-Turanian	Ko	Chamaephyte	NO
1751	Jurinea cordata Boiss. & Hausskn. ex Boiss.	Asteraceae	Irano-Turanian	Za	Chamaephyte	YES
1752	Jurinea eriobasis DC.	Asteraceae	Irano-Turanian	Za	Hemicryptophyte	NO
1753	Jurinea gabrielae Bornm.	Asteraceae	Irano-Turanian		Chamaephyte	YES
1754	Jurinea gedrosiaca Bornm.	Asteraceae	Saharo-Sindian		Chamaephyte	YES

1755	Jurinea giviensis Mirtadz.	Asteraceae	Irano-Turanian	Az	Hemicryptophyte	YES
1756	Jurinea heterophylla (Jaub. & Spach) Boiss.	Asteraceae	Irano-Turanian	Az, Al, Za	Chamaephyte	NO
1757	Jurinea inuloides Boiss. & Hausskn. ex Boiss.	Asteraceae	Irano-Turanian	Za	Chamaephyte	YES
1758	Jurinea jedresiaca Bornm.	Asteraceae	Saharo-Sindian		Hemicryptophyte	YES
1759	Jurinea kopetensis Rech.f.	Asteraceae	Irano-Turanian	Ko	Chamaephyte	YES
1760	Jurinea leptoloba DC.	Asteraceae	Irano-Turanian	Az	Chamaephyte	NO
1761	Jurinea meda Bornm.	Asteraceae	Irano-Turanian	Za	Chamaephyte	NO
1762	Jurinea mobayenii Ghahr. & Mirtadz.	Asteraceae	Irano-Turanian	Ke	Hemicryptophyte	YES
1763	Jurinea monocephala Aitch. & Hemsl.	Asteraceae	Irano-Turanian	Ko	Chamaephyte	NO
1764	Jurinea multicaulis DC.	Asteraceae	Irano-Turanian	Az	Chamaephyte	YES
1765	Jurinea prasinophylla Rech.f.	Asteraceae	Irano-Turanian	Za	Chamaephyte	YES
1766	Jurinea proteoides Boiss. & Hausskn. ex Boiss.	Asteraceae	Irano-Turanian	Za	Chamaephyte	YES
1767	Jurinea radians Boiss.	Asteraceae	Irano-Turanian	Az, Al, Ko, Ke	Chamaephyte	NO
1768	Jurinea sharifiana Rech.f. & Esfand.	Asteraceae	Irano-Turanian	Al	Chamaephyte	YES
1769	Jurinea stenocalathia Rech.f.	Asteraceae	Irano-Turanian	Al, Ko, Ke	Chamaephyte	NO
1770	Jurinea viciosoi Pau	Asteraceae	Irano-Turanian	Za	Chamaephyte	YES
1771	Jurinella frigida (Boiss.) Wagenitz	Asteraceae	Irano-Turanian	Al	Chamaephyte	NO
1772	Jurinella microcephala (Boiss.) Wagenitz	Asteraceae	Irano-Turanian	Al, Ko	Hemicryptophyte	NO
1773	Kalakia marginata (Boiss.) Alava	Apiaceae	Irano-Turanian	Al	Therophyte	NO
1774	Karvandarina aphylla Rech.f., Aellen & Esfand.	Asteraceae	Saharo-Sindian		Hemicryptophyte	NO
1775	Kaviria zehzadii (Akhani) Akhani	Chenopodiaceae	Irano-Turanian		Therophyte	YES
1776	Kelussia odoratissima Mozaff.	Apiaceae	Irano-Turanian	Za	Chamaephyte	YES
1777	Klasea calcarea (Mozaff.) Ranjbar & Negaresh	Asteraceae	Irano-Turanian	Az	Hemicryptophyte	YES
1778	Klasea gracillima (Rech.f.) L.Martins	Asteraceae	Irano-Turanian	Al	Hemicryptophyte	YES
1779	Klasea melanocheila (Boiss. & Hausskn.) J.Holub	Asteraceae	Irano-Turanian	Za	Hemicryptophyte	YES
1780	Klasea nana Ranjbar & Negaresh	Asteraceae	Irano-Turanian	Ko	Hemicryptophyte	YES
1781	Klasea sanandajensis Ranjbar & Negaresh	Asteraceae	Irano-Turanian	Za	Hemicryptophyte	YES
1782	Klasea suffulta (Rech.f.) L.Martins	Asteraceae	Irano-Turanian	Za	Hemicryptophyte	YES
1783	Klasea viciifolia (Boiss. & Hausskn.) L.Martins	Asteraceae	Irano-Turanian	Az, Za	Hemicryptophyte	YES
1784	Lactuca azerbaijanica Rech.f.	Asteraceae	Irano-Turanian	Az, Al	Hemicryptophyte	YES
1785	Lactuca birjandica Mozaff.	Asteraceae	Irano-Turanian		Hemicryptophyte	YES

1786	Lactuca denaensis N. Kilian & Djavadi	Asteraceae	Irano-Turanian	Za	Hemicryptophyte	YES
1787	Lactuca gilanica Mozaff.	Asteraceae	Euro-Siberian		Hemicryptophyte	YES
1788	Lactuca hazaranensis Djavadi & N. Kilian	Asteraceae	Irano-Turanian	Ke	Hemicryptophyte	YES
1789	Lactuca polyclada Boiss.	Asteraceae	Irano-Turanian	Za	Hemicryptophyte	YES
1790	Lagochilus alutaceus Bunge	Lamiaceae	Irano-Turanian	Al, Ko	Chamaephyte	NO
1791	Lagochilus aucheri Boiss.	Lamiaceae	Irano-Turanian	Az, Al, Za	Chamaephyte	NO
1792	Lagochilus kotschyanus Boiss.	Lamiaceae	Irano-Turanian	Az, Al	Hemicryptophyte	NO
1793	Lagochilus lasiocalyx (Stapf) Jamzad	Lamiaceae	Irano-Turanian	Za	Chamaephyte	NO
1794	Lagochilus lorestanicus Dehshiri & Mozaff.	Lamiaceae	Irano-Turanian	Za	Chamaephyte	YES
1795	Lagochilus macracanthus Fisch. & C.A.Mey.	Lamiaceae	Irano-Turanian	Al, Za	Chamaephyte	NO
1796	Lagochilus quadridentatus Jamzad	Lamiaceae	Euro-Siberian	Al	Chamaephyte	YES
1797	Lamium bakhtiaricum Jamzad	Lamiaceae	Irano-Turanian	Za	Hemicryptophyte	YES
1798	Laser rechingeri Akhani	Apiaceae	Euro-Siberian	Al	Hemicryptophyte	YES
1799	Lathyrus alamutensis Mozaff., Ahavazi & Charkhch.	Fabaceae	Irano-Turanian	Al	Hemicryptophyte	YES
1800	Launaea acanthodes (Boiss.) Kuntze	Asteraceae	Irano-Turanian		Hemicryptophyte	NO
1801	Launaea bornmuelleri (Hausskn. ex Bornm.) Bornm.	Asteraceae	Saharo-Sindian		Hemicryptophyte	YES
1802	Launaea piestocarpa (Boiss.) Rech.f.	Asteraceae	Irano-Turanian	Ko	Chamaephyte	YES
1803	Lavandula sublepidota Rech.f.	Lamiaceae	Saharo-Sindian		Chamaephyte	YES
1804	Leontodon stenocalathius Rech.f.	Asteraceae	Euro-Siberian	Al	Hemicryptophyte	YES
1805	Leopoldia tabriziana Jafari	Asparagaceae	Irano-Turanian	Az	Geophyte	YES
1806	Lepechiniella fursei Riedl	Boraginaceae	Irano-Turanian	Al	Hemicryptophyte	YES
1807	Lepechiniella persica (Boiss.) Riedl	Boraginaceae	Irano-Turanian	Al	Hemicryptophyte	NO
1808	Lepechiniella wendelboi Riedl	Boraginaceae	Irano-Turanian	Al	Hemicryptophyte	NO
1809	Leucopoa pseudosclerophylla (Krivot.) Bor	Poaceae	Irano-Turanian	Ke, Ko	Hemicryptophyte	YES
1810	Leutea avicennae Mozaff.	Apiaceae	Irano-Turanian	Za	Hemicryptophyte	YES
1811	Leutea cupularis (Boiss.) Pimen.	Apiaceae	Irano-Turanian	Al, Za	Hemicryptophyte	NO
1812	Leutea elbursensis Mozaff.	Apiaceae	Irano-Turanian	Al	Hemicryptophyte	YES
1813	Leutea gracillima Pimenov	Apiaceae	Irano-Turanian	Al	Hemicryptophyte	YES
1814	Leutea kurdistanica Mozaff.	Apiaceae	Irano-Turanian	Za	Hemicryptophyte	YES
1815	Leutea nematoloba (Rech.f.) Pimenov	Apiaceae	Irano-Turanian, Euro-Siberian	Al	Hemicryptophyte	NO

1816	Leutea petiolaris (DC.) Pimenov	Apiaceae	Irano-Turanian	Az, Al, Za, Ko	Hemicryptophyte	NO
1817	Leutea polyscias (Boiss.) Pimenov	Apiaceae	Euro-Siberian		Hemicryptophyte	YES
1818	Leutea rechingeri (Leute) Pimenov	Apiaceae	Irano-Turanian	Az	Hemicryptophyte	YES
1819	Ligularia persica Boiss.	Asteraceae	Irano-Turanian	Al	Hemicryptophyte	NO
1820	Lilium ledebourii (Baker) Boiss.	Hamamelidaceae	Euro-Siberian	Al	Hemicryptophyte	YES
1821	Linaria azerbaijanensis Hamdi & Assadi	Scrophulariaceae	Irano-Turanian	Az	Hemicryptophyte	NO
1822	Linaria boushehrensis Hamdi & Assadi	Scrophulariaceae	Saharo-Sindian		Hemicryptophyte	NO
1823	Linaria elymaitica (Boiss.) Kuprian.	Scrophulariaceae	Irano-Turanian	Al, Za	Hemicryptophyte	YES
1824	Linaria farsensis Hamdi & Assadi	Scrophulariaceae	Irano-Turanian	Za	Hemicryptophyte	YES
1825	Linaria golestanensis Hamdi & Assadi	Scrophulariaceae	Euro-Siberian	Al, Ko	Hemicryptophyte	NO
1826	Linaria guilanensis Hamdi & Assadi	Scrophulariaceae	Irano-Turanian	Al	Hemicryptophyte	YES
1827	Linaria iranica Hamdi & Assadi	Scrophulariaceae	Irano-Turanian	Ke	Hemicryptophyte	YES
1828	Linaria karajensis Hamdi & Assadi	Scrophulariaceae	Irano-Turanian	Al	Hemicryptophyte	YES
1829	Linaria kavirensis Hamdi & Assadi	Scrophulariaceae	Irano-Turanian		Hemicryptophyte	YES
1830	Linaria khalkhalensis Hamdi & Assadi	Scrophulariaceae	Irano-Turanian, Euro-Siberian	Az, Al	Hemicryptophyte	YES
1831	Linaria khorasanensis Hamdi & Assadi	Scrophulariaceae	Irano-Turanian	Ko	Hemicryptophyte	NO
1832	Linaria lineolata Boiss.	Scrophulariaceae	Irano-Turanian	Al	Hemicryptophyte	NO
1833	Linaria mazandaranensis Hamdi & Assadi	Scrophulariaceae	Euro-Siberian	Al	Hemicryptophyte	YES
1834	Linaria michauxii Chav.	Scrophulariaceae	Irano-Turanian	Al, Za, Ke, Ko, Az, Lo	Hemicryptophyte	NO
1835	Linaria nurensis Boiss. & Hausskn.	Scrophulariaceae	Irano-Turanian	Za	Hemicryptophyte	NO
1836	Linaria orientalis Hamdi & Assadi	Scrophulariaceae	Irano-Turanian	Al	Hemicryptophyte	YES
1837	Linaria remotiflora Patzak	Scrophulariaceae	Irano-Turanian	Ke, Za	Hemicryptophyte	NO
1838	Linaria Shahroudensis Hamdi & Assadi	Scrophulariaceae	Irano-Turanian	Al	Hemicryptophyte	YES
1839	Lindelofia kandavanensis Bornm. & Gauba	Boraginaceae	Euro-Siberian	Al	Hemicryptophyte	YES
1840	Linum persicum Boiss.	Linaceae	Irano-Turanian	Za	Chamaephyte	NO
1841	Lomatopodium staurophyllum (Rech.f.) Rech.f.	Apiaceae	Irano-Turanian	Al, Ko	Hemicryptophyte	NO
1842	Malabaila isfahanica Alava	Apiaceae	Irano-Turanian	Za	Hemicryptophyte	YES
1843	Malabaila kotschyi Boiss.	Apiaceae	Irano-Turanian	Za, Ko	Hemicryptophyte	NO
1844	Malabaila porphyrodiscus Stapf & Wettst. ex Stapf	Apiaceae	Irano-Turanian	Za, Az	Hemicryptophyte	NO
1845	Marrubium cordatum Nábelek	Lamiaceae	Irano-Turanian	Az	Hemicryptophyte	YES

1846	Marrubium eriocephalum Seybold	Lamiaceae	Irano-Turanian	Az	Hemicryptophyte	YES
1847	Marrubium procerum Bunge	Lamiaceae	Irano-Turanian	Ko	Hemicryptophyte	YES
1848	Matthiola dumulosa Boiss. & Buhse	Brassicaceae	Irano-Turanian	Ko	Chamaephyte	NO
1849	Matthiola iranica Zeraatkar, Mahmoodi, F.Ghahrem. & Maassoumi	Brassicaceae	Irano-Turanian	Az	Hemicryptophyte	YES
1850	Matthiola ovatifolia Boiss.	Brassicaceae	Irano-Turanian	Az, Al, Ke, Za	Hemicryptophyte	NO
1851	Matthiola revoluta Bunge ex Boiss.	Brassicaceae	Irano-Turanian	Al, Za, Ke	Chamaephyte	NO
1852	Matthiola shehbazii Ranjbar & Karami	Brassicaceae	Irano-Turanian	Za	Hemicryptophyte	YES
1853	Matthiola subglabra Ponert	Brassicaceae	Irano-Turanian	Al	Chamaephyte	YES
1854	Mattiastrum pygmaeum Rech.f.	Boraginaceae	Irano-Turanian	Al	Hemicryptophyte	YES
1855	Mentha mozaffarianii Jamzad	Lamiaceae	Irano-Turanian	Ke	Hemicryptophyte	NO
1856	Michauxia koeieana Rech.f.	Campanulaceae	Saharo-Sindian		Hemicryptophyte	YES
1857	Michauxia stenophylla Boiss. & Hausskn.	Campanulaceae	Saharo-Sindian		Hemicryptophyte	NO
1858	Micrantha multicaulis (Boiss.) F. Dvořák	Brassicaceae	Irano-Turanian	Za, Ke	Hemicryptophyte	NO
1859	Micromeria hedgei Rech.f.	Lamiaceae	Saharo-Sindian		Chamaephyte	NO
1860	Milium atropatanum Maroofi	Poaceae	Irano-Turanian	Az	Hemicryptophyte	YES
1861	Minuartia acuminata Turrill	Caryophyllaceae	Irano-Turanian	Az	Hemicryptophyte	YES
1862	Minuartia aucheriana Bornm.	Caryophyllaceae	Irano-Turanian	Za	Chamaephyte	YES
1863	Minuartia glandulosa (Boiss. & A. Huet) Bornm.	Caryophyllaceae	Irano-Turanian	Az	Hemicryptophyte	NO
1864	Minuartia khorassanica Assadi & Mostafavi	Caryophyllaceae	Irano-Turanian		Hemicryptophyte	YES
1865	Minuartia lineata (Boiss.) Bornm.	Caryophyllaceae	Irano-Turanian	Al	Hemicryptophyte	NO
1866	Minuartia litwinowii Schischk.	Caryophyllaceae	Irano-Turanian	Al, Ko	Hemicryptophyte	NO
1867	Minuartia sabalanica Assadi & Mostafavi	Caryophyllaceae	Irano-Turanian	Az	Hemicryptophyte	YES
1868	Minuartia sublineata Rech.f.	Caryophyllaceae	Irano-Turanian	Az, Za	Hemicryptophyte	NO
1869	Mozaffariania insignis Pimenov & Maassoumi	Apiaceae	Saharo-Sindian		Hemicryptophyte	NO
1870	Muscari kurdicum Maroofi	Asparagaceae	Irano-Turanian	Za	Geophyte	YES
1871	Muscari pseudomuscari (Boiss. & Buhse) Wendelbo	Asparagaceae	Euro-Siberian	Al	Geophyte	YES
1872	Myopordon aucheri Boiss.	Asteraceae	Irano-Turanian	Za	Chamaephyte	YES
1873	Myopordon damavandica Mozaff.	Asteraceae	Irano-Turanian	Al	Chamaephyte	YES
1874	Myopordon hyrcanum (Bornm.) Wagenitz	Asteraceae	Irano-Turanian	Al	Chamaephyte	YES
1875	Myopordon persicum Boiss.	Asteraceae	Irano-Turanian	Za	Chamaephyte	YES
1876	Myosotis anomala Riedl	Boraginaceae	Irano-Turanian	Az	Hemicryptophyte	NO

1877	Myosotis koelzii Riedl	Boraginaceae	Irano-Turanian	Za	Therophyte	YES
1878	Nanorrhinum baluchestanicum Naanaie, Assadi & Tavassoli	Scrophulariaceae	Saharo-Sindian		Hemicryptophyte	YES
1879	Nanorrhinum campyloceras (Rech.f. & Esfand.) Naanaie, Assadi & Tavassoli	Scrophulariaceae	Saharo-Sindian		Hemicryptophyte	NO
1880	Nanorrhinum chasmophyticum (Wendelbo) Naanaie, Assadi & Tavassoli	Scrophulariaceae	Saharo-Sindian		Hemicryptophyte	YES
1881	Nanorrhinum khuzestanicum Naanaie, Assadi & Tavassoli	Scrophulariaceae	Saharo-Sindian		Hemicryptophyte	NO
1882	Nectaroscordum koelzii Wendelbo	Alliaceae	Irano-Turanian	Za	Geophyte	NO
1883	Neocryptodiscus persicus (Boiss.) Hedge & Lamond	Apiaceae	Irano-Turanian		Hemicryptophyte	NO
1884	Nepeta adenoclada Bornm.	Lamiaceae	Irano-Turanian	Za	Chamaephyte	YES
1885	Nepeta allotria Rech.f.	Lamiaceae	Irano-Turanian	Al	Hemicryptophyte	YES
1886	Nepeta archibaldii Rech.f.	Lamiaceae	Irano-Turanian	Za	Chamaephyte	YES
1887	Nepeta assadii Jamzad	Lamiaceae	Irano-Turanian	Al, Za	Hemicryptophyte	NO
1888	Nepeta assurgens Hausskn. & Bornm.	Lamiaceae	Irano-Turanian	Ko, Ke	Hemicryptophyte	NO
1889	Nepeta asterotricha Rech.f.	Lamiaceae	Irano-Turanian	Ke	Hemicryptophyte	YES
1890	Nepeta bakhtiarica Rech.f.	Lamiaceae	Irano-Turanian	Ke, Za	Therophyte	NO
1891	Nepeta balouchestanica Jamzad & Ingr.	Lamiaceae	Irano-Turanian		Hemicryptophyte	YES
1892	Nepeta bazoftica Jamzad	Lamiaceae	Irano-Turanian	Za	Hemicryptophyte	YES
1893	Nepeta binaloudensis Jamzad	Lamiaceae	Irano-Turanian	Ko	Hemicryptophyte	YES
1894	Nepeta bokhonica Jamzad	Lamiaceae	Irano-Turanian	Ke	Hemicryptophyte	YES
1895	Nepeta bornmuelleri Hausskn. ex Bornm.	Lamiaceae	Irano-Turanian	Ke	Therophyte	NO
1896	Nepeta cephalotes Boiss.	Lamiaceae	Irano-Turanian	Az, Al	Chamaephyte	NO
1897	Nepeta chionophila Boiss. & Hausskn.	Lamiaceae	Irano-Turanian	Za	Hemicryptophyte	YES
1898	Nepeta crassifolia Boiss. & Buhse	Lamiaceae	Irano-Turanian, Euro-Siberian	Al, Za, Ke, Ko	Hemicryptophyte	NO
1899	Nepeta crispa Willd.	Lamiaceae	Irano-Turanian	Za, Al	Hemicryptophyte	NO
1900	Nepeta denudata Benth.	Lamiaceae	Irano-Turanian	Az, Al	Hemicryptophyte	NO
1901	Nepeta depauperata Benth.	Lamiaceae	Irano-Turanian	Za, Ke	Chamaephyte	NO
1902	Nepeta dschuparensis Bornm.	Lamiaceae	Irano-Turanian, Saharo-Sindian	Ke	Chamaephyte	NO
1903	Nepeta elymaitica Bornm.	Lamiaceae	Irano-Turanian	Za	Hemicryptophyte	YES
1904	Nepeta eremokosmos Rech.f.	Lamiaceae	Irano-Turanian	Al	Chamaephyte	YES

1905	Nepeta gloeocephala Rech.f.	Lamiaceae	Irano-Turanian	Za, Ke	Hemicryptophyte	NO
1906	Nepeta hormozganica Jamzad	Lamiaceae	Saharo-Sindian		Therophyte	NO
1907	Nepeta hymenodonta Boiss.	Lamiaceae	Irano-Turanian	Ke	Therophyte	YES
1908	Nepeta iranshahrii Rech.f.	Lamiaceae	Irano-Turanian	Za	Hemicryptophyte	YES
1909	Nepeta koieana Rech.f.	Lamiaceae	Saharo-Sindian		Therophyte	YES
1910	Nepeta kotschyi Boiss.	Lamiaceae	Irano-Turanian	Za	Chamaephyte	NO
1911	Nepeta lasiocephala Benth.	Lamiaceae	Irano-Turanian	Za, Ke	Hemicryptophyte	NO
1912	Nepeta laxiflora Benth.	Lamiaceae	Irano-Turanian	Za	Hemicryptophyte	NO
1913	Nepeta macrosiphon Boiss.	Lamiaceae	Irano-Turanian	Az, Za	Hemicryptophyte	NO
1914	Nepeta mahanensis Jamzad & M.Simmonds	Lamiaceae	Irano-Turanian	Ke	Therophyte	YES
1915	Nepeta makuensis Jamzad & Mozaff.	Lamiaceae	Irano-Turanian	Az	Hemicryptophyte	YES
1916	Nepeta menthoides Boiss. & Buhse	Lamiaceae	Irano-Turanian	Az, Al	Hemicryptophyte	NO
1917	Nepeta minuticephala Jamzad	Lamiaceae	Irano-Turanian	Za	Hemicryptophyte	YES
1918	Nepeta monocephala Rech.f.	Lamiaceae	Irano-Turanian	Za	Hemicryptophyte	YES
1919	Nepeta natanzensis Jamzad	Lamiaceae	Irano-Turanian	Za, Ke	Hemicryptophyte	YES
1920	Nepeta oxyodonta Boiss.	Lamiaceae	Irano-Turanian	Za, Ke	Hemicryptophyte	NO
1921	Nepeta pogonosperma Jamzad & Assadi	Lamiaceae	Irano-Turanian	Az, Al	Hemicryptophyte	YES
1922	Nepeta prostrata Benth.	Lamiaceae	Irano-Turanian	Za	Hemicryptophyte	NO
1923	Nepeta racemosa Lam.	Lamiaceae	Irano-Turanian	Az, Al	Hemicryptophyte	NO
1924	Nepeta rivularis Bornm.	Lamiaceae	Irano-Turanian	Ke	Hemicryptophyte	YES
1925	Nepeta sahandica Noroozi & Ajani	Lamiaceae	Irano-Turanian	Az, Al	Chamaephyte	NO
1926	Nepeta schiraziana Boiss.	Lamiaceae	Irano-Turanian	Za, Ke, Al	Therophyte	NO
1927	Nepeta sessilifolia Bunge	Lamiaceae	Irano-Turanian	Za	Hemicryptophyte	NO
1928	Nepeta shahmirzadensis Assadi & Jamzad	Lamiaceae	Irano-Turanian	Al	Hemicryptophyte	YES
1929	Nepeta straussii Hausskn. & Bornm.	Lamiaceae	Irano-Turanian	Za	Therophyte	NO
1930	Noccaea apterocarpa (Rech.f. & Aellen) Al-Shehbaz & Menke	Brassicaceae	Irano-Turanian	Ko	Hemicryptophyte	YES
1931	Noccaea tenuis (Boiss. & Buhse) F.K. Mey.	Brassicaceae	Irano-Turanian	Az	Hemicryptophyte	YES
1932	Noccidium tuberculatum F.K. Mey.	Brassicaceae	Euro-Siberian	Al	Hemicryptophyte	YES
1933	Nonea anchusoides Boiss. & Buhse	Boraginaceae	Irano-Turanian	Az	Hemicryptophyte	NO
1934	Nonea hypoleia Bornm.	Boraginaceae	Irano-Turanian	Za	Hemicryptophyte	YES
1935	Nonea iranica Falat. & Pakravan	Boraginaceae	Irano-Turanian	Al, Az, Ke	Therophyte	NO

1936	Nonea persica Boiss.	Boraginaceae	Irano-Turanian	Az, Al, Za, Ke	Hemicryptophyte	NO
1937	Onobrychis alamutensis Amirahm., Kaz.Osaloo, Charkhch.	Fabaceae	Irano-Turanian	Al	Hemicryptophyte	YES
1938	Onobrychis alborzensis Ranjbar & Hajmoradi	Fabaceae	Irano-Turanian	Al	Hemicryptophyte	YES
1939	Onobrychis andalanica Bornm.	Fabaceae	Irano-Turanian	Za	Hemicryptophyte	YES
1940	Onobrychis assadii Ranjbar, Tolui & Amirab.	Fabaceae	Irano-Turanian	Ko	Chamaephyte	YES
1941	Onobrychis aucheri Boiss.	Fabaceae	Irano-Turanian	Az, Al	Therophyte	NO
1942	Onobrychis aurea Ranjbar, Amirab. & Ghahrem.	Fabaceae	Irano-Turanian	Az	Hemicryptophyte	YES
1943	Onobrychis depauperata Boiss.	Fabaceae	Irano-Turanian	Az	Hemicryptophyte	YES
1944	Onobrychis elymaitica Boiss. & Hausskn. ex Boiss.	Fabaceae	Irano-Turanian	Za	Chamaephyte	YES
1945	Onobrychis gaubae Bornm.	Fabaceae	Irano-Turanian	Az,Al	Hemicryptophyte	NO
1946	Onobrychis gypsicola Rech.f.	Fabaceae	Saharo-Sindian		Chamaephyte	NO
1947	Onobrychis heliocarpa Boiss.	Fabaceae	Irano-Turanian	Az, Al	Therophyte	NO
1948	Onobrychis iranensis Amirab. & Ghanavati	Fabaceae	Irano-Turanian	Ko, Lo	Therophyte	NO
1949	Onobrychis iranshahrii Rech.f.	Fabaceae	Saharo-Sindian		Chamaephyte	YES
1950	Onobrychis kermanensis (Sirj. & Rech.f.) Rech.f.	Fabaceae	Irano-Turanian	Ke	Chamaephyte	YES
1951	Onobrychis longipes Bunge	Fabaceae	Irano-Turanian	Ko	Hemicryptophyte	YES
1952	Onobrychis luristanica Rech.f.	Fabaceae	Irano-Turanian	Za	Hemicryptophyte	YES
1953	Onobrychis marandensis Amirab. & Ghanavati	Fabaceae	Irano-Turanian	Az	Hemicryptophyte	YES
1954	Onobrychis mazanderanica Rech.f.	Fabaceae	Euro-Siberian	Al	Hemicryptophyte	NO
1955	Onobrychis melanotricha Boiss.	Fabaceae	Irano-Turanian	Za, Al	Chamaephyte	NO
1956	Onobrychis mozaffarianii Amirab.	Fabaceae	Irano-Turanian	Za	Chamaephyte	YES
1957	Onobrychis oshnaviyehensis Ranjbar	Fabaceae	Irano-Turanian	Az	Hemicryptophyte	YES
1958	Onobrychis oxyptera Boiss.	Fabaceae	Irano-Turanian	Za	Hemicryptophyte	YES
1959	Onobrychis persica Sirj. & Rech.f.	Fabaceae	Irano-Turanian	Az, Za	Hemicryptophyte	NO
1960	Onobrychis plantago Bornm.	Fabaceae	Irano-Turanian	Ke	Chamaephyte	YES
1961	Onobrychis psoraleifolia Boiss.	Fabaceae	Irano-Turanian	Za	Hemicryptophyte	NO
1962	Onobrychis ptychophylla Sirj. & Rech.f.	Fabaceae	Irano-Turanian	Ko	Chamaephyte	YES
1963	Onobrychis rechingerorum Wendelbo	Fabaceae	Saharo-Sindian		Therophyte	YES
1964	Onobrychis scrobiculata Boiss.	Fabaceae	Irano-Turanian	Za, Az	Hemicryptophyte	NO
1965	Onobrychis sojakii Rech.f.	Fabaceae	Irano-Turanian	Za	Hemicryptophyte	YES
1966	Onobrychis subnitens Bornm.	Fabaceae	Irano-Turanian	Az	Chamaephyte	NO

1967	Onobrychis susiana Nabelek	Fabaceae	Saharo-Sindian		Hemicryptophyte	YES
1968	Onobrychis szovitsii Boiss.	Fabaceae	Irano-Turanian	Az	Chamaephyte	YES
1969	Onobrychis talagonica Rech.f.	Fabaceae	Irano-Turanian	Al	Chamaephyte	YES
1970	Onopordon carmanicum (Bornm.) Bornm.	Asteraceae	Irano-Turanian	Ke, Lo	Hemicryptophyte	NO
1971	Onosma azarbaidjanensis Mehrabian	Boraginaceae	Irano-Turanian	Az	Hemicryptophyte	YES
1972	Onosma bilabiata Boiss. & Buhse	Boraginaceae	Irano-Turanian	Az, Za	Hemicryptophyte	NO
1973	Onosma bisotunensis Attar & Hamzehee	Boraginaceae	Irano-Turanian	Za	Hemicryptophyte	YES
1974	Onosma chrysochaetum Bornm.	Boraginaceae	Irano-Turanian	Az, Al, Za	Hemicryptophyte	NO
1975	Onosma demavendica Riedl	Boraginaceae	Irano-Turanian	Al, Za	Hemicryptophyte	NO
1976	Onosma ghahremanii Attar & Naqinezhad	Boraginaceae	Irano-Turanian	Al	Hemicryptophyte	YES
1977	Onosma iranshahrii Ghahr. & Attar	Boraginaceae	Irano-Turanian	Za	Hemicryptophyte	YES
1978	Onosma khorassanica Attar & Joharchi	Boraginaceae	Irano-Turanian	Ko	Hemicryptophyte	YES
1979	Onosma kilouyense Boiss. & Hausskn.	Boraginaceae	Irano-Turanian	Al, Za	Hemicryptophyte	NO
1980	Onosma kotschyi Boiss.	Boraginaceae	Irano-Turanian	Az, Al, Za , Ke	Hemicryptophyte	NO
1981	Onosma maculata Ranjbar & Almasi	Boraginaceae	Irano-Turanian	Za	Hemicryptophyte	YES
1982	Onosma mozaffariani Mehrabian	Boraginaceae	Irano-Turanian	Za	Hemicryptophyte	YES
1983	Onosma pachypoda Boiss.	Boraginaceae	Irano-Turanian	Al, Az, Za	Hemicryptophyte	NO
1984	Onosma platyphylla Riedl	Boraginaceae	Irano-Turanian	Za	Hemicryptophyte	NO
1985	Onosma sabalanica Ponert	Boraginaceae	Irano-Turanian	Az	Hemicryptophyte	YES
1986	Onosma sheidaii Mehrabian	Boraginaceae	Irano-Turanian	Za	Hemicryptophyte	YES
1987	Onosma stenosiphon Boiss.	Boraginaceae	Irano-Turanian	Az, Al, Ke, Ko	Hemicryptophyte	NO
1988	Onosma straussii (Riedl) Khat.	Boraginaceae	Irano-Turanian	Za	Hemicryptophyte	NO
1989	Ophrys kurdistanica Renz	Orchidaceae	Irano-Turanian	Az	Geophyte	YES
1990	Ophrys turcomanica Renz	Orchidaceae	Euro-Siberian		Geophyte	YES
1991	Opoidia galbanifera Lindl.	Apiaceae	Irano-Turanian	Ko	Hemicryptophyte	YES
1992	Opsicarpium insignis Mozaff.	Apiaceae	Irano-Turanian	Az, Za	Hemicryptophyte	NO
1993	Oreophysa microphylla (Jaub. & Spach) Browicz	Fabaceae	Irano-Turanian	Al	Chamaephyte	NO
1994	Ornithogalum boissieri Bidarlord & F.Ghahrem.	Asparagaceae	Irano-Turanian	Al	Geophyte	YES
1995	Ornithogalum khuzestanicum Heidaryan, Hamdi & Assadi	Asparagaceae	Saharo-Sindian		Geophyte	YES
1996	Ornithogalum pycnanthum Wendelbo	Asparagaceae	Irano-Turanian	Za	Geophyte	YES
1997	Ornithogalum sanandajense Maroofi	Asparagaceae	Irano-Turanian	Za	Geophyte	YES

1998	Orobanche eriophora Bornm. & Gauba	Orobanchaceae	Euro-Siberian	Al	Geophyte	YES
1999	Orobanche longibracteata Schiman-Czeika	Orobanchaceae	Irano-Turanian	Za	Geophyte	YES
2000	Orobanche pulchra Gilli	Orobanchaceae	Irano-Turanian	Al, Za, Ko	Geophyte	NO
2001	Orobanche schwingenschussii Gilli	Orobanchaceae	Irano-Turanian	Al	Geophyte	YES
2002	Otostegia michauxii Briq.	Lamiaceae	Irano-Turanian	Za	Chamaephyte	YES
2003	Oxytropis aellenii Vassilcz.	Fabaceae	Irano-Turanian	Al	Hemicryptophyte	YES
2004	Oxytropis azerbaijanica Podlech	Fabaceae	Irano-Turanian	Az	Hemicryptophyte	YES
2005	Oxytropis bakhtiarica Maassoumi	Fabaceae	Irano-Turanian	Za	Hemicryptophyte	YES
2006	Oxytropis bicornis Vassilcz.	Fabaceae	Irano-Turanian	Al, Ko	Hemicryptophyte	NO
2007	Oxytropis binaludensis Vassilcz.	Fabaceae	Irano-Turanian	Al, Ko	Hemicryptophyte	NO
2008	Oxytropis chrysocarpa Boiss.	Fabaceae	Irano-Turanian	Al, Za, Lo	Hemicryptophyte	NO
2009	Oxytropis cinerea Vassilcz.	Fabaceae	Irano-Turanian	Al	Hemicryptophyte	YES
2010	Oxytropis compacta Maassoumi & Joharchi	Fabaceae	Irano-Turanian	Ko	Hemicryptophyte	YES
2011	Oxytropis gracillima Vassilcz.	Fabaceae	Irano-Turanian	Ko	Hemicryptophyte	YES
2012	Oxytropis guilanica Maassoumi & Moradi	Fabaceae	Irano-Turanian	Az	Hemicryptophyte	YES
2013	Oxytropis hypsophila Bunge ex Boiss.	Fabaceae	Irano-Turanian	Ko, Lo	Hemicryptophyte	NO
2014	Oxytropis indurata Maassoumi	Fabaceae	Irano-Turanian	Ko	Hemicryptophyte	YES
2015	Oxytropis Iranica Vassilcz.	Fabaceae	Irano-Turanian	Al	Hemicryptophyte	YES
2016	Oxytropis javaherdehi Maassoumi	Fabaceae	Euro-Siberian	Al	Hemicryptophyte	YES
2017	Oxytropis karjaginii Grossh.	Fabaceae	Irano-Turanian	Az, Al, Za	Hemicryptophyte	YES
2018	Oxytropis kermanica Freyn & Bornm.	Fabaceae	Irano-Turanian	Al, Za, Ke	Hemicryptophyte	NO
2019	Oxytropis kordkoyensis Maassoumi	Fabaceae	Euro-Siberian	Al	Hemicryptophyte	YES
2020	Oxytropis kuchanensis Vassilcz.	Fabaceae	Irano-Turanian	Al, Ko	Hemicryptophyte	NO
2021	Oxytropis mahneshanensis Maassoumi	Fabaceae	Irano-Turanian	Az	Hemicryptophyte	YES
2022	Oxytropis masanderanensis Vassilcz.	Fabaceae	Irano-Turanian	Al, Za	Hemicryptophyte	YES
2023	Oxytropis neorechingeriana Vassilcz.	Fabaceae	Irano-Turanian	Ko	Hemicryptophyte	YES
2024	Oxytropis persica Boiss.	Fabaceae	Irano-Turanian	Az, Al	Hemicryptophyte	NO
2025	Oxytropis pseudosuavis Maassoumi	Fabaceae	Irano-Turanian		Hemicryptophyte	YES
2026	Oxytropis rechingeri Vassilcz.	Fabaceae	Irano-Turanian	Ko	Hemicryptophyte	YES
2027	Oxytropis rhodontha Vassilcz.	Fabaceae	Irano-Turanian	Az	Hemicryptophyte	YES
2028	Oxytropis rudbariensis Vassilcz.	Fabaceae	Irano-Turanian	Al	Hemicryptophyte	YES

2029	Oxytropis sabzavarensis Maassoumi	Fabaceae	Irano-Turanian	Ko	Hemicryptophyte	YES
2030	Oxytropis salukensis Maassoumi	Fabaceae	Irano-Turanian	Ko	Hemicryptophyte	YES
2031	Oxytropis shahvarica Maassoumi	Fabaceae	Irano-Turanian	Al	Hemicryptophyte	YES
2032	Oxytropis shirkuhi Vassilcz.	Fabaceae	Irano-Turanian	Ke	Hemicryptophyte	YES
2033	Oxytropis sivehensis Maassoumi & Amini Rad	Fabaceae	Irano-Turanian	Az	Hemicryptophyte	YES
2034	Oxytropis surmandehi Vassilcz.	Fabaceae	Irano-Turanian	Za	Hemicryptophyte	YES
2035	Oxytropis szovitsii Boiss. & Buhse	Fabaceae	Irano-Turanian	Az, Ko	Hemicryptophyte	NO
2036	Oxytropis yazdi Vassilcz.	Fabaceae	Irano-Turanian	Ke	Hemicryptophyte	YES
2037	Oxytropis zangolehensis Vassilcz.	Fabaceae	Irano-Turanian	Al	Hemicryptophyte	YES
2038	Papaver armeniacum (L.) DC.	Papaveraceae	Irano-Turanian	Al, Lo	Therophyte	YES
2039	Papaver gaubae Cullen & Rech.f.	Papaveraceae	Irano-Turanian	Al, Lo	Therophyte	YES
2040	Papaver persicum Lindl.	Papaveraceae	Irano-Turanian	Az	Hemicryptophyte	NO
2041	Papaver tenuifolium Boiss. & Hohen.	Papaveraceae	Irano-Turanian	Al, Za	Hemicryptophyte	NO
2042	Paracaryum cyclhymenium (Boiss.) Riedl	Boraginaceae	Irano-Turanian	Al, Za, Ke	Hemicryptophyte	NO
2043	Paracaryum glandulosum Khat.	Boraginaceae	Irano-Turanian	Za	Hemicryptophyte	YES
2044	Paracaryum gracile Czerniak.	Boraginaceae	Irano-Turanian	Ko	Hemicryptophyte	YES
2045	Paracaryum khorassanicum Khat.	Boraginaceae	Irano-Turanian	Ko	Hemicryptophyte	YES
2046	Paracaryum luristanicum Nábělek	Boraginaceae	Irano-Turanian, Saharo-Sindian		Hemicryptophyte	NO
2047	Paracaryum modestum Boiss. & Hausskn.	Boraginaceae	Irano-Turanian	Za, Ke	Hemicryptophyte	NO
2048	Paracaryum persicum (Boiss.) Boiss.	Boraginaceae	Irano-Turanian	Za, Ke, Al	Hemicryptophyte	NO
2049	Paracaryum pygmaeum (Rech.f.) D.Heller	Boraginaceae	Irano-Turanian	Al	Hemicryptophyte	YES
2050	Paracaryum tenerum Bornm.	Boraginaceae	Irano-Turanian	Za	Hemicryptophyte	YES
2051	Paraquilegia caespitosa (Boiss. & Hohen.) J.R. Drumm. & Hutch.	Ranunculaceae	Irano-Turanian	Al	Hemicryptophyte	YES
2052	Parlatoria rostrata Boiss.	Brassicaceae	Irano-Turanian	Al, Za	Therophyte	NO
2053	Paronychia bungei Boiss.	Caryophyllaceae	Irano-Turanian, Saharo-Sindian		Chamaephyte	NO
2054	Paronychia caespitosa Stapf	Caryophyllaceae	Irano-Turanian	Al, Za	Hemicryptophyte	NO
2055	Paronychia lordeganica Dinarvand & Assadi	Caryophyllaceae	Irano-Turanian	Za	Hemicryptophyte	YES
2056	Parrotia persica C.A.Mey.	Hamamelidaceae	Euro-Siberian	Al	Phanerophyte	NO
2057	Pedicularis rechingeri Wendelbo	Scrophulariaceae	Irano-Turanian	Ko	Hemicryptophyte	YES
2058	Pedicularis straussii Hausskn.	Scrophulariaceae	Irano-Turanian	Za	Hemicryptophyte	YES

2059	Pentanema flexuosum (Boiss. & Hausskn.) Rech.f.	Asteraceae	Irano-Turanian	Az, Za	Hemicryptophyte	NO
2060	Pentanema kurdistanicum Maroofi & Ghaderi	Asteraceae	Irano-Turanian	Az	Hemicryptophyte	YES
2061	Pentanema multicaule Boiss.	Asteraceae	Irano-Turanian	Za	Chamaephyte	NO
2062	Pentanema pulicariiforme (DC.) Rech.f.	Asteraceae	Irano-Turanian	Al, Za	Chamaephyte	NO
2063	Petrorhagia macra (Boiss. & Hausskn.) P.W.Ball & Heywood	Caryophyllaceae	Irano-Turanian	Za	Hemicryptophyte	YES
2064	Peucedanum chenur Mozaff.	Apiaceae	Irano-Turanian	Za	Hemicryptophyte	YES
2065	Peucedanum glaucopruinosum Rech.f.	Apiaceae	Euro-Siberian	Al	Hemicryptophyte	YES
2066	Peucedanum knappii Bornm.	Apiaceae	Irano-Turanian	Az	Hemicryptophyte	YES
2067	Peucedanum pimenovii Mozaff.	Apiaceae	Euro-Siberian	Al	Hemicryptophyte	YES
2068	Peucedanum translucens Rech.f.	Apiaceae	Euro-Siberian	Al	Hemicryptophyte	YES
2069	Phagnalon persicum Boiss.	Asteraceae	Irano-Turanian	Za, Ke	Chamaephyte	NO
2070	Phleum iranicum Bornm. & Gauba	Poaceae	Irano-Turanian	Al	Hemicryptophyte	NO
2071	Phlomis anisodonta Boiss.	Lamiaceae	Irano-Turanian	Al, Za	Hemicryptophyte	NO
2072	Phlomis aucheri Boiss.	Lamiaceae	Irano-Turanian	Za, Ke	Hemicryptophyte	NO
2073	Phlomis chorassanica Bunge	Lamiaceae	Irano-Turanian	Ko	Hemicryptophyte	YES
2074	Phlomis elliptica Benth.	Lamiaceae	Irano-Turanian	Za	Chamaephyte	NO
2075	Phlomis ghilanensis K.Koch	Lamiaceae	Irano-Turanian		Hemicryptophyte	YES
2076	Phlomis lurestanica Jamzad	Lamiaceae	Irano-Turanian	Za	Hemicryptophyte	YES
2077	Phlomis mazandaranica Jamzad	Lamiaceae	Euro-Siberian	Al	Hemicryptophyte	YES
2078	Phlomis pachyphylla Rech.f.	Lamiaceae	Saharo-Sindian		Hemicryptophyte	NO
2079	Phlomis persica Boiss.	Lamiaceae	Irano-Turanian, Euro-Siberian, Saharo-Sindian	Al, Za	Hemicryptophyte	NO
2080	Phlomoides adenantha (Jaub. & Spach) Kamelin & Makhm.	Lamiaceae	Irano-Turanian, Saharo-Sindian	Za, Ke	Hemicryptophyte	NO
2081	Phlomoides azerbaijanica (Rech.f.) Kamelin & Makhm.	Lamiaceae	Irano-Turanian	Az	Hemicryptophyte	NO
2082	Phlomoides codonocalyx (Rech.f.) Kamelin & Makhm.	Lamiaceae	Irano-Turanian		Hemicryptophyte	YES
2083	Phlomoides hyoscyamoides (Boiss. & Buhse) Kamelin & Makhm.	Lamiaceae	Irano-Turanian	Ko, Lo	Hemicryptophyte	NO
2084	Phlomoides kermanica Ranjbar & C.Mahmoudi	Lamiaceae	Irano-Turanian	Ke	Hemicryptophyte	YES
2085	Phlomoides lanata (Jamzad) Salmaki	Lamiaceae	Irano-Turanian	Al	Hemicryptophyte	YES
2086	Phlomoides pulvinaris (Jaub. & Spach) Kamelin & Makhm.	Lamiaceae	Irano-Turanian	Za, Ko, Al	Hemicryptophyte	NO
2087	Phuopsis stylosa (Trin.) Hook.f. ex B.D.Jacks.	Rubiaceae	Euro-Siberian	Al	Hemicryptophyte	NO

2088	Physogeton acanthophyllus Jaub. & Spach	Chenopodiaceae	Irano-Turanian	Za, Az, Al	Therophyte	NO
2089	Physogeton occultus (Bunge) Assadi	Chenopodiaceae	Irano-Turanian		Therophyte	NO
2090	Physogeton pedunculatus (Assadi) Assadi	Chenopodiaceae	Saharo-Sindian		Therophyte	YES
2091	Physoptychis gnaphalodes Boiss.	Brassicaceae	Irano-Turanian	Az, Za, Al	Hemicryptophyte	NO
2092	Pimpinella anisactis Rech.f.	Apiaceae	Irano-Turanian	Az, Al ,Ko	Hemicryptophyte	NO
2093	Pimpinella deverroides Boiss.	Apiaceae	Irano-Turanian	Za	Hemicryptophyte	NO
2094	Pimpinella dichotoma (Boiss. & Hausskn.) H.Wolff	Apiaceae	Irano-Turanian	Za, Ke	Hemicryptophyte	NO
2095	Pimpinella gedrosiaca Bornm.	Apiaceae	Irano-Turanian		Hemicryptophyte	YES
2096	Pimpinella khayyamii Mozaff.	Apiaceae	Irano-Turanian	Ko	Hemicryptophyte	YES
2097	Pimpinella khorasanica Engstrand	Apiaceae	Irano-Turanian	Ko	Hemicryptophyte	NO
2098	Pimpinella tragioides Boiss.	Apiaceae	Irano-Turanian	Az, Al	Hemicryptophyte	NO
2099	Piptatherum denaense Hamzehee & Assadi	Poaceae	Irano-Turanian	Za	Hemicryptophyte	YES
2100	Piptatherum molinioides Boiss.	Poaceae	Irano-Turanian	Al, Za , Ke	Hemicryptophyte	NO
2101	Plantago podlechii Akhani	Plantaginaceae	Euro-Siberian		Chamaephyte	YES
2102	Platychaete aucheri (Boiss.) Boiss.	Asteraceae	Irano-Turanian, Saharo-Sindian		Chamaephyte	NO
2103	Platychaete mucronifolia (Boiss.) Boiss.	Asteraceae	Irano-Turanian, Saharo-Sindian		Chamaephyte	NO
2104	Platychaete velutina Boiss. & Hausskn. ex Boiss.	Asteraceae	Saharo-Sindian		Chamaephyte	YES
2105	Poa demavandica Assadi & Kavousi	Poaceae	Irano-Turanian	Al	Hemicryptophyte	YES
2106	Poa golestanensis H.Scholz & Akhani	Poaceae	Euro-Siberian		Hemicryptophyte	YES
2107	Polygala platyptera Bornm. & Gauba	Polygalaceae	Irano-Turanian, Euro-Siberian	Al	Hemicryptophyte	NO
2108	Polygonum aridum Boiss. & Hausskn.	Polygonaceae	Irano-Turanian	Za	Chamaephyte	NO
2109	Polygonum botuliforme Mozaff.	Polygonaceae	Irano-Turanian	Za	Hemicryptophyte	YES
2110	Polygonum dumosum Boiss.	Polygonaceae	Irano-Turanian	Za, Ke	Chamaephyte	NO
2111	Polygonum hyrcanicum Rech.f.	Polygonaceae	Irano-Turanian, Euro-Siberian	Al, Lo	Hemicryptophyte	NO
2112	Polygonum iranicum Mozaff.	Polygonaceae	Irano-Turanian	Za	Chamaephyte	YES
2113	Polygonum salicornioides Jaub. & Spach ex Boiss.	Polygonaceae	Irano-Turanian	Za, Ke	Chamaephyte	NO
2114	Polygonum spinosum H.Gross	Polygonaceae	Irano-Turanian	Ke	Chamaephyte	NO
2115	Polylophium involucratum Boiss.	Apiaceae	Euro-Siberian	Al	Hemicryptophyte	NO

2116	Postia bombycina Boiss. & Hausskn.	Asteraceae	Irano-Turanian, Saharo-Sindian		Chamaephyte	YES
2117	Postia puberula Boiss. & Hausskn.	Asteraceae	Saharo-Sindian		Chamaephyte	NO
2118	Potentilla argyroloma Boiss. & Hohen.	Rosaceae	Irano-Turanian	Al, Za	Hemicryptophyte	NO
2119	Potentilla aucheriana Th.Wolf ex Bornm.	Rosaceae	Irano-Turanian	Al, Az	Hemicryptophyte	NO
2120	Potentilla elvendensis Boiss.	Rosaceae	Irano-Turanian	Za	Hemicryptophyte	NO
2121	Potentilla farsistanica Browicz	Rosaceae	Irano-Turanian	Za	Hemicryptophyte	YES
2122	Potentilla flaccida Th.Wolf ex Bornm.	Rosaceae	Irano-Turanian	Al, Za	Hemicryptophyte	NO
2123	Potentilla gaubaeana Bornm.	Rosaceae	Irano-Turanian	Al	Hemicryptophyte	YES
2124	Potentilla gilanica (Th.Wolf) Th.Wolf	Rosaceae	Euro-Siberian	Al	Hemicryptophyte	YES
2125	Potentilla iranica (Rech.f.) Schiman-Czeika	Rosaceae	Irano-Turanian	Al, Az, Ko	Hemicryptophyte	NO
2126	Potentilla kandavanensis Bornm. & Gauba	Rosaceae	Irano-Turanian	Al	Hemicryptophyte	YES
2127	Potentilla lignosa Willd. ex Schltdl.	Rosaceae	Irano-Turanian	Al, Za	Hemicryptophyte	YES
2128	Potentilla mallota Boiss.	Rosaceae	Irano-Turanian	Al, Za	Hemicryptophyte	YES
2129	Potentilla nuda Boiss.	Rosaceae	Irano-Turanian	Az, Al, Za, Ke, Ko	Hemicryptophyte	NO
2130	Potentilla nurensis Boiss. & Hausskn. ex Boiss.	Rosaceae	Irano-Turanian	Az, Za	Hemicryptophyte	NO
2131	Potentilla pannosa Boiss. & Hausskn. ex Boiss.	Rosaceae	Irano-Turanian	Al, Za	Hemicryptophyte	NO
2132	Potentilla petraea Willd. ex Schltdl.	Rosaceae	Irano-Turanian	Al	Hemicryptophyte	YES
2133	Potentilla polyschista Boiss. & Hohen.	Rosaceae	Irano-Turanian	Al, Az	Hemicryptophyte	NO
2134	Potentilla porphyrantha Juz.	Rosaceae	Irano-Turanian	Az, Al	Hemicryptophyte	NO
2135	Potentilla poteriifolia Boiss.	Rosaceae	Irano-Turanian	Al, Za, Ke	Hemicryptophyte	NO
2136	Potentilla radiata Lehm.	Rosaceae	Euro-Siberian	Al	Hemicryptophyte	YES
2137	Potentilla schiraziana Khat.	Rosaceae	Irano-Turanian	Za	Hemicryptophyte	YES
2138	Prangos calligonoides Rech.f.	Apiaceae	Irano-Turanian	Za	Hemicryptophyte	YES
2139	Prangos cheilanthifolia Boiss.	Apiaceae	Irano-Turanian	Al, Za, Ke	Hemicryptophyte	NO
2140	Prangos crossoptera Herrnst. & Heyn	Apiaceae	Irano-Turanian	Za	Hemicryptophyte	YES
2141	Prangos gaubae (Bornm.) Herrnstadt & Heyn	Apiaceae	Irano-Turanian	Az, Al, Ko	Hemicryptophyte	NO
2142	Prangos tuberculata Boiss. & Hausskn.	Apiaceae	Irano-Turanian	Za, Ke	Hemicryptophyte	NO
2143	Primula gaubaeana Bornm.	Primulaceae	Irano-Turanian	Al, Za, Lo	Hemicryptophyte	NO
2144	Psephellus congestus (Wagenitz) Wagenitz	Asteraceae	Irano-Turanian	Az	Hemicryptophyte	YES
2145	Psephellus khalkhalensis Ranjbar & Negaresh	Asteraceae	Euro-Siberian		Hemicryptophyte	YES

2146	Pseudocamelina aphragmodes (Boiss.) N. Busch	Brassicaceae	Irano-Turanian	Za	Hemicryptophyte	YES
2147	Pseudocamelina camelinae N.Busch	Brassicaceae	Irano-Turanian	Za, Ke	Hemicryptophyte	YES
2148	Pseudocamelina campylocarpa (Boiss.) N. Busch	Brassicaceae	Irano-Turanian	Za	Hemicryptophyte	YES
2149	Pseudocamelina glaucophylla N. Busch	Brassicaceae	Irano-Turanian	Al, Az, Za	Hemicryptophyte	NO
2150	Pseudocamelina szowitsii (Boiss.) N. Busch	Brassicaceae	Irano-Turanian	Az	Hemicryptophyte	YES
2151	Pseudocamelina violacea (Boiss.) N. Busch	Brassicaceae	Irano-Turanian	Za	Chamaephyte	YES
2152	Pseudofortuynia esfandiarii Hedge	Brassicaceae	Irano-Turanian	Za	Hemicryptophyte	YES
2153	Pseudofortuynia leucoclada (Boiss.) Khosravi	Brassicaceae	Irano-Turanian	Za, Ke	Hemicryptophyte	NO
2154	Pseudotrachydium kotschyi (Boiss.) Pimenov & Kljuykov	Apiaceae	Irano-Turanian	Za	Hemicryptophyte	NO
2155	Pseudotrachydium pauciradiatum (Boiss. & Hohen.) Pimenov & Kljuykov	Apiaceae	Irano-Turanian	Al	Hemicryptophyte	NO
2156	Psychrogeton aellenii (Rech.f.) Grierson	Asteraceae	Irano-Turanian	Al, Ko	Hemicryptophyte	YES
2157	Psychrogeton chionophilus (Boiss.) Krasch.	Asteraceae	Irano-Turanian	Za	Hemicryptophyte	YES
2158	Psychrogeton persicus (Boiss.) Grierson	Asteraceae	Irano-Turanian	Za, Ke, Ko	Hemicryptophyte	NO
2159	Pterocephalus ghahremanii Jamzad	Dipsacaceae	Irano-Turanian	Za	Chamaephyte	YES
2160	Pterocephalus lignosus Freyn & Bornm.	Dipsacaceae	Irano-Turanian	Za	Chamaephyte	NO
2161	Pterocephalus melanobasis Pau	Dipsacaceae	Irano-Turanian	Za	Chamaephyte	YES
2162	Pterocephalus persicus Boiss.	Dipsacaceae	Irano-Turanian	Za, Ke	Chamaephyte	NO
2163	Pterocephalus wendelboi Rech.f.	Dipsacaceae	Irano-Turanian	Ke	Chamaephyte	YES
2164	Pycnocycla acanthorhipsis Rech.f., Aellen & Esfand.	Apiaceae	Saharo-Sindian		Chamaephyte	YES
2165	Pycnocycla bashagardiana Mozaff.	Apiaceae	Saharo-Sindian		Chamaephyte	YES
2166	Pycnocycla musiformis Hedge & Lamond	Apiaceae	Irano-Turanian		Chamaephyte	YES
2167	Pyrus ghahremanii Attar & Zamani	Rosaceae	Euro-Siberian	Al	Phanerophyte	YES
2168	Pyrus giffanica Zamani & Attar	Rosaceae	Irano-Turanian	Ko	Phanerophyte	YES
2169	Pyrus glabra Boiss.	Rosaceae	Irano-Turanian	Za	Phanerophyte	NO
2170	Pyrus kandevanica Ghahr., Khat. & Mozaff.	Rosaceae	Irano-Turanian, Euro-Siberian	Al	Phanerophyte	YES
2171	Pyrus longipedicellata Zamani & Attar	Rosaceae	Euro-Siberian	Al	Phanerophyte	YES
2172	Pyrus mazanderanica Schönb.-Tem.	Rosaceae	Irano-Turanian, Euro-Siberian	Al	Phanerophyte	YES
2173	Quercus brantii Lindl.	Fagaceae	Irano-Turanian	Za	Phanerophyte	NO
2174	Ranunculus amblyolobus Boiss. & Hohen.	Ranunculaceae	Irano-Turanian,	Az, Al	Hemicryptophyte	NO

			Euro-Siberian			
2175	Ranunculus aucheri Boiss.	Ranunculaceae	Irano-Turanian	Za, Ke, Al	Hemicryptophyte	NO
2176	Ranunculus bulbilliferus Boiss. & Hohen.	Ranunculaceae	Irano-Turanian	Al	Hemicryptophyte	YES
2177	Ranunculus crymophilus Boiss. & Hohen.	Ranunculaceae	Irano-Turanian	Az, Al, Za	Hemicryptophyte	NO
2178	Ranunculus dalechanensis Iranshahr & Rech.f.	Ranunculaceae	Irano-Turanian	Za	Hemicryptophyte	YES
2179	Ranunculus diversifolius Boiss.& Kotschy	Ranunculaceae	Irano-Turanian	Az, Za	Hemicryptophyte	YES
2180	Ranunculus elbursensis Boiss.	Ranunculaceae	Irano-Turanian	Al, Za	Hemicryptophyte	NO
2181	Ranunculus elymaiticus Boiss. & Hausskn.	Ranunculaceae	Irano-Turanian	Za	Hemicryptophyte	NO
2182	Ranunculus eriorrhizus Boiss. & Buhse	Ranunculaceae	Irano-Turanian	Ke	Hemicryptophyte	NO
2183	Ranunculus farsicus Rech.f.	Ranunculaceae	Irano-Turanian	Za	Hemicryptophyte	YES
2184	Ranunculus koeiei Rech.f.	Ranunculaceae	Irano-Turanian		Hemicryptophyte	YES
2185	Ranunculus microflorus Pakravan	Ranunculaceae	Irano-Turanian	Za	Hemicryptophyte	YES
2186	Ranunculus papyrocarpus Rech.f., Aellen & Esfand.	Ranunculaceae	Irano-Turanian	Ke	Hemicryptophyte	NO
2187	Ranunculus pichleri Freyn ex Stapf	Ranunculaceae	Irano-Turanian	Za	Hemicryptophyte	NO
2188	Ranunculus renzii Iranshahr & Rech.f.	Ranunculaceae	Irano-Turanian	Az	Hemicryptophyte	YES
2189	Ranunculus sahendicus Boiss. & Buhse	Ranunculaceae	Irano-Turanian	Az	Hemicryptophyte	YES
2190	Ranunculus sojakii Iranshahr & Rech.f.	Ranunculaceae	Irano-Turanian	Al	Hemicryptophyte	YES
2191	Ranunculus straussii Bornm.	Ranunculaceae	Irano-Turanian	Za	Hemicryptophyte	YES
2192	Ranunculus termei Iranshahr & Rech.f.	Ranunculaceae	Irano-Turanian	Za	Hemicryptophyte	YES
2193	Ranunculus trichocarpus Boiss. & Kotschy	Ranunculaceae	Irano-Turanian	Az, Al	Hemicryptophyte	NO
2194	Ranunculus zenjanensis Iranshahr & Rech.f.	Ranunculaceae	Irano-Turanian	Az, Za	Hemicryptophyte	YES
2195	Reseda buhseana Müll.Arg.	Resedaceae	Irano-Turanian	Za, Ke	Hemicryptophyte	NO
2196	Reseda bungei Boiss.	Resedaceae	Irano-Turanian		Hemicryptophyte	NO
2197	Reseda macrobotrys Boiss.	Resedaceae	Irano-Turanian	Za, Ke	Hemicryptophyte	NO
2198	Rhabdosciadium aucheri Boiss.	Apiaceae	Irano-Turanian	Za	Hemicryptophyte	NO
2199	Rhabdosciadium petiolare Boiss. & Hausskn.	Apiaceae	Irano-Turanian	Za, Al	Hemicryptophyte	YES
2200	Rhabdosciadium straussii Hausskn. ex Bornm.	Apiaceae	Irano-Turanian	Za	Hemicryptophyte	YES
2201	Rhamnus cornifolia Boiss. & Hohen.	Rhamnaceae	Irano-Turanian	Az, Za	Phanerophyte	NO
2202	Rhaponticoides bachtiarica (Boiss. & Hausskn.) L.Martins	Asteraceae	Irano-Turanian	Za	Hemicryptophyte	YES
2203	Rhaponticum insigne (Boiss.) Wagenitz	Asteraceae	Irano-Turanian	Az	Hemicryptophyte	NO
2204	Rheum austroiranicum Taheri & Assadi	Polygonaceae	Saharo-Sindian		Hemicryptophyte	YES

2205	Rheum iranshahrii Taheri & Assadi	Polygonaceae	Irano-Turanian		Hemicryptophyte	YES
2206	Rheum kordestanicum Taheri & Assadi	Polygonaceae	Irano-Turanian	Az, Za, Ko	Hemicryptophyte	NO
2207	Rheum neyshabourense Baradaran & Jafari	Polygonaceae	Irano-Turanian	Ko	Hemicryptophyte	YES
2208	Rheum persicum Losinsk.	Polygonaceae	Irano-Turanian	Za	Hemicryptophyte	YES
2209	Rhopalosciadium stereocalyx Rech.f.	Apiaceae	Irano-Turanian	Za	Therophyte	YES
2210	Ribes khorasanicum Saghafi & Assadi	Grossulariaceae	Irano-Turanian	Ko	Phanerophyte	YES
2211	Rindera bungei Gürke	Boraginaceae	Irano-Turanian	Al, Ko	Hemicryptophyte	NO
2212	Rindera media (Turrill) Riedl	Boraginaceae	Irano-Turanian	Az	Hemicryptophyte	YES
2213	Rindera regia Kusn.	Boraginaceae	Irano-Turanian	Al	Hemicryptophyte	YES
2214	Rochelia mirheydari Riedl & Esfand.	Boraginaceae	Irano-Turanian	Ko	Therophyte	NO
2215	Rochelia retrosepala Khat.	Boraginaceae	Irano-Turanian	Za	Therophyte	YES
2216	Rorippa kurdica (Boiss. & Hausskn.) Hedge	Brassicaceae	Irano-Turanian	Za	Hemicryptophyte	YES
2217	Rosa abrica Khat. & Koobaz	Rosaceae	Irano-Turanian	Za	Phanerophyte	YES
2218	Rubia albicaulis Boiss.	Rubiaceae	Irano-Turanian	Za, Ke	Chamaephyte	NO
2219	Rubia caramanica Bornm.	Rubiaceae	Irano-Turanian	Ke	Chamaephyte	YES
2220	Rubia pauciflora Boiss.	Rubiaceae	Irano-Turanian	Za	Chamaephyte	NO
2221	Rumex elbrusensis Boiss.	Polygonaceae	Irano-Turanian	Az, Za, Al	Hemicryptophyte	NO
2222	Rumex ephedroides Bornm.	Polygonaceae	Saharo-Sindian		Chamaephyte	NO
2223	Rumex kandavanicus (Rech.f.) Rech.f.	Polygonaceae	Irano-Turanian	Al	Hemicryptophyte	YES
2224	Salicornia iranica Akhani	Chenopodiaceae	Irano-Turanian	Al, Za	Therophyte	YES
2225	Salicornia persica Akhani	Chenopodiaceae	Irano-Turanian	Al, Za	Therophyte	NO
2226	Salicornia perspolitana Akhani	Chenopodiaceae	Irano-Turanian	Za	Therophyte	YES
2227	Salicornia sinus-persica Akhani	Chenopodiaceae	Saharo-Sindian		Therophyte	NO
2228	Salix baladehensis Maassoumi, Moeeni & Rahimin.	Salicaceae	Euro-Siberian	Al	Phanerophyte	YES
2229	Salix elymaitica Maassoumi	Salicaceae	Irano-Turanian	Za	Phanerophyte	YES
2230	Salix firouzkuhensis Maassoumi	Salicaceae	Irano-Turanian	Al	Phanerophyte	YES
2231	Salix issatissensis Maassoumi, Moeeni & Rahimin.	Salicaceae	Irano-Turanian	Za, Ke	Phanerophyte	NO
2232	Salix lacus-tari Maassoumi & Kazempour	Salicaceae	Irano-Turanian	Al	Phanerophyte	YES
2233	Salix viridiformis Maassoumi	Salicaceae	Irano-Turanian	Az	Phanerophyte	YES
2234	Salsola abarghuensis Assadi	Chenopodiaceae	Irano-Turanian		Phanerophyte	YES
2235	Salsola austro-iranica Akhani	Chenopodiaceae	Saharo-Sindian		Therophyte	YES

2236	Salsola persica Bunge ex Boiss.	Chenopodiaceae	Irano-Turanian	Az	Chamaephyte	NO
2237	Salsola yazdiana Assadi	Chenopodiaceae	Irano-Turanian	Ke, Lo	Phanerophyte	NO
2238	Salvia aristata Aucher ex Benth.	Lamiaceae	Irano-Turanian	Al, Az, Za	Hemicryptophyte	NO
2239	Salvia bazmanica Rech.f. & Esfand.	Lamiaceae	Saharo-Sindian		Chamaephyte	NO
2240	Salvia chorassanica Bunge	Lamiaceae	Irano-Turanian	Ko	Hemicryptophyte	NO
2241	Salvia eremophila Boiss.	Lamiaceae	Irano-Turanian	Za, Ke	Chamaephyte	NO
2242	Salvia hypochionaea Boiss.	Lamiaceae	Irano-Turanian	Az	Hemicryptophyte	YES
2243	Salvia hypoleuca Benth.	Lamiaceae	Irano-Turanian	Al	Hemicryptophyte	NO
2244	Salvia jamzadii Mozaff.	Lamiaceae	Irano-Turanian	Za	Hemicryptophyte	YES
2245	Salvia kermanshahensis Rech.f.	Lamiaceae	Irano-Turanian	Za	Chamaephyte	NO
2246	Salvia lachnocalyx Hedge	Lamiaceae	Irano-Turanian	Za	Hemicryptophyte	YES
2247	Salvia oligophylla Aucher ex Benth.	Lamiaceae	Irano-Turanian, Euro-Siberian	Az, Al	Hemicryptophyte	NO
2248	Salvia persepolitana Boiss.	Lamiaceae	Irano-Turanian, Saharo-Sindian	Za, Lo	Hemicryptophyte	NO
2249	Salvia sahendica Boiss. & Buhse	Lamiaceae	Irano-Turanian	Az	Hemicryptophyte	NO
2250	Salvia sclareopsis Bornm. ex Hedge	Lamiaceae	Irano-Turanian	Az, Za	Hemicryptophyte	NO
2251	Salvia shahkuhmahalei Akhani	Lamiaceae	Irano-Turanian	Al	Chamaephyte	YES
2252	Salvia sharifii Rech.f. & Esfand.	Lamiaceae	Irano-Turanian, Saharo-Sindian		Hemicryptophyte	NO
2253	Salvia urmiensis Bunge	Lamiaceae	Irano-Turanian	Az, Al, Za	Hemicryptophyte	NO
2254	Salvia wendelboi Hedge	Lamiaceae	Irano-Turanian	Za	Chamaephyte	YES
2255	Saponaria bodeana Boiss.	Caryophyllaceae	Irano-Turanian	Al	Hemicryptophyte	NO
2256	Saponaria esfandiarii Assadi	Caryophyllaceae	Saharo-Sindian		Therophyte	YES
2257	Saponaria iranica Dashti, Assadi & Sharifnia	Caryophyllaceae	Irano-Turanian	Ke	Therophyte	YES
2258	Satureja atropatana Bunge	Lamiaceae	Irano-Turanian	Az	Chamaephyte	YES
2259	Satureja avromanica Maroofi	Lamiaceae	Irano-Turanian	Za	Chamaephyte	YES
2260	Satureja bachtiarica Bunge	Lamiaceae	Irano-Turanian	Za, Ke	Chamaephyte	NO
2261	Satureja edmondi Briq.	Lamiaceae	Irano-Turanian	Za	Chamaephyte	NO
2262	Satureja isophylla Rech.f.	Lamiaceae	Irano-Turanian	Al	Chamaephyte	NO
2263	Satureja kallarica Jamzad	Lamiaceae	Irano-Turanian	Za	Chamaephyte	YES
2264	Satureja kermanshahensis Jamzad	Lamiaceae	Saharo-Sindian		Chamaephyte	YES

2265	Satureja khuzistanica Jamzad	Lamiaceae	Saharo-Sindian		Chamaephyte	YES
2266	Satureja rechingeri Jamzad	Lamiaceae	Saharo-Sindian		Chamaephyte	YES
2267	Satureja sahendica Bornm.	Lamiaceae	Irano-Turanian	Al, Za	Chamaephyte	NO
2268	Saxifraga iranica Bornm.	Saxifragaceae	Irano-Turanian	Al	Chamaephyte	YES
2269	Saxifraga koelzii Schönb.-Tem.	Saxifragaceae	Irano-Turanian	Al	Chamaephyte	YES
2270	Saxifraga mazanderanica Rech.f.	Saxifragaceae	Euro-Siberian	Al	Chamaephyte	YES
2271	Saxifraga ramsarica Jamzad	Saxifragaceae	Irano-Turanian	Al	Chamaephyte	YES
2272	Saxifraga wendelboi Schönb.-Tem.	Saxifragaceae	Irano-Turanian	Al	Chamaephyte	YES
2273	Scabiosa esfandiarii Jamzad	Dipsacaceae	Irano-Turanian	Za	Therophyte	YES
2274	Scabiosa schimperiana Boiss. & Buhse	Dipsacaceae	Euro-Siberian		Hemicryptophyte	YES
2275	Scilla bisotunensis Speta	Asparagaceae	Irano-Turanian	Za	Geophyte	YES
2276	Scilla gorganica Speta	Asparagaceae	Euro-Siberian	Al	Geophyte	YES
2277	Scilla greilhuberi Speta	Asparagaceae	Euro-Siberian	Al	Geophyte	NO
2278	Scilla khorassanica Meikle	Asparagaceae	Irano-Turanian	Ko, Al	Geophyte	NO
2279	Sclerochorton haussknechtii Boiss.	Apiaceae	Irano-Turanian	Za	Hemicryptophyte	YES
2280	Sclerorhachis leptoclada Rech.f.	Asteraceae	Irano-Turanian		Hemicryptophyte	YES
2281	Sclerorhachis platyrachis (Boiss.) Podlech ex Rech.f.	Asteraceae	Irano-Turanian	Ko, Lo	Hemicryptophyte	NO
2282	Scorzonera calyculata Boiss.	Asteraceae	Irano-Turanian	Az, Al, Za	Hemicryptophyte	NO
2283	Scorzonera flaccida Rech.f.	Asteraceae	Irano-Turanian	Za	Geophyte	YES
2284	Scorzonera grossheimii Lipsch. & Vassilcz.	Asteraceae	Irano-Turanian	Al, Za	Hemicryptophyte	NO
2285	Scorzonera helodes Rech.f.	Asteraceae	Irano-Turanian	Za	Geophyte	YES
2286	Scorzonera intricata Boiss.	Asteraceae	Irano-Turanian	Za, Ke, Bl	Chamaephyte	NO
2287	Scorzonera ispahanica Boiss.	Asteraceae	Irano-Turanian	Za	Hemicryptophyte	NO
2288	Scorzonera joharchii S.R.Safavi	Asteraceae	Irano-Turanian		Geophyte	YES
2289	Scorzonera kandavanica Rech.f.	Asteraceae	Euro-Siberian	Al	Hemicryptophyte	YES
2290	Scorzonera kirpicznikovii Lipsch.	Asteraceae	Euro-Siberian	Al	Hemicryptophyte	YES
2291	Scorzonera limnophila Boiss.	Asteraceae	Irano-Turanian	Za	Geophyte	YES
2292	Scorzonera luristanica Rech.f.	Asteraceae	Irano-Turanian	Al, Za	Hemicryptophyte	NO
2293	Scorzonera microcalathia (Rech.f.) Rech.f.	Asteraceae	Irano-Turanian	Ke, Ko, Lo	Hemicryptophyte	NO
2294	Scorzonera mucida Rech.f., Aellen & Esfand.	Asteraceae	Irano-Turanian	Al, Za ,Ke, Ko	Geophyte	NO
2295	Scorzonera nivalis Boiss. & Hausskn. ex Boiss.	Asteraceae	Irano-Turanian	Za	Hemicryptophyte	YES

2296	Scorzonera persica Boiss. & Buhse	Asteraceae	Irano-Turanian	Al	Hemicryptophyte	YES
2297	Scorzonera persepolitana Boiss.	Asteraceae	Irano-Turanian	Za	Hemicryptophyte	NO
2298	Scorzonera psychrophila Boiss. & Hausskn. ex Boiss. & Hausskn.	Asteraceae	Irano-Turanian	Za	Geophyte	YES
2299	Scorzonera renzii Rech.f.	Asteraceae	Irano-Turanian	Az	Hemicryptophyte	NO
2300	Scorzonera rupicola Hausskn.	Asteraceae	Irano-Turanian	Az, Za	Chamaephyte	NO
2301	Scorzonera stenocephala Boiss.	Asteraceae	Irano-Turanian	Al, Za	Geophyte	NO
2302	Scorzonera subaphylla Boiss.	Asteraceae	Irano-Turanian	Za	Hemicryptophyte	YES
2303	Scorzonera wendelboi Rech.f.	Asteraceae	Irano-Turanian	Al	Hemicryptophyte	YES
2304	Scorzonera xylobasis Rech.f.	Asteraceae	Irano-Turanian	Al	Hemicryptophyte	YES
2305	Scrophularia alhagioides Attar & Joharchi	Scrophulariaceae	Irano-Turanian		Chamaephyte	YES
2306	Scrophularia atroglandulosa Grau	Scrophulariaceae	Irano-Turanian	Az	Hemicryptophyte	YES
2307	Scrophularia crassicaulis Boiss.	Scrophulariaceae	Irano-Turanian	Al	Chamaephyte	NO
2308	Scrophularia crassipedunculata Attar & Joharchi	Scrophulariaceae	Irano-Turanian	Ko	Chamaephyte	YES
2309	Scrophularia crassiuscula Grau	Scrophulariaceae	Irano-Turanian	Za	Hemicryptophyte	NO
2310	Scrophularia denaensis Attar	Scrophulariaceae	Irano-Turanian	Za	Chamaephyte	YES
2311	Scrophularia elbursensis Bornm.	Scrophulariaceae	Irano-Turanian	Az, Za, Al	Hemicryptophyte	NO
2312	Scrophularia elymaitica Mozaff.	Scrophulariaceae	Saharo-Sindian		Hemicryptophyte	YES
2313	Scrophularia farinosa Boiss.	Scrophulariaceae	Irano-Turanian	Za	Hemicryptophyte	NO
2314	Scrophularia flava Grau	Scrophulariaceae	Irano-Turanian	Za	Hemicryptophyte	YES
2315	Scrophularia frigida Boiss.	Scrophulariaceae	Irano-Turanian	Az, Al, Ko, Za ,Ke	Chamaephyte	NO
2316	Scrophularia gaubae Bornm.	Scrophulariaceae	Irano-Turanian, Saharo-Sindian	Al	Hemicryptophyte	NO
2317	Scrophularia ghahremanii Attar & Hamzehee	Scrophulariaceae	Saharo-Sindian		Hemicryptophyte	YES
2318	Scrophularia glauca Decne. ex Benth.	Scrophulariaceae	Irano-Turanian	Za	Hemicryptophyte	NO
2319	Scrophularia gorganica Rech.f.	Scrophulariaceae	Irano-Turanian	Al, Ko	Hemicryptophyte	YES
2320	Scrophularia iranica Attar	Scrophulariaceae	Irano-Turanian	Za	Hemicryptophyte	YES
2321	Scrophularia ispahanica Attar & Nowrouzi	Scrophulariaceae	Irano-Turanian	Za	Hemicryptophyte	YES
2322	Scrophularia kermanica Ghahr. & Mirtadz.	Scrophulariaceae	Irano-Turanian	Ke	Hemicryptophyte	YES
2323	Scrophularia khorassanica Attar & Joharchi	Scrophulariaceae	Irano-Turanian	Ko	Hemicryptophyte	YES
2324	Scrophularia megalantha Rech.f.	Scrophulariaceae	Euro-Siberian	Al	Hemicryptophyte	NO
2325	Scrophularia oxysepala Boiss.	Scrophulariaceae	Irano-Turanian	Az	Hemicryptophyte	YES

2326	Scrophularia papyracea Attar	Scrophulariaceae	Irano-Turanian	Za	Chamaephyte	YES
2327	Scrophularia rechingeri Grau	Scrophulariaceae	Irano-Turanian	Az	Hemicryptophyte	YES
2328	Scrophularia rostrata Boiss. & Buhse	Scrophulariaceae	Irano-Turanian, Euro-Siberian	Al, Az	Hemicryptophyte	NO
2329	Scrophularia schiraziana Attar & Hatami	Scrophulariaceae	Irano-Turanian	Za	Hemicryptophyte	YES
2330	Scrophularia shulabadensis Attar & Hamzehee	Scrophulariaceae	Irano-Turanian	Za	Hemicryptophyte	YES
2331	Scrophularia subaphylla Boiss.	Scrophulariaceae	Irano-Turanian	Az, Za, Ke, Al	Hemicryptophyte	NO
2332	Scrophularia tortuosissima Attar & Joharchi	Scrophulariaceae	Irano-Turanian	Ko	Hemicryptophyte	YES
2333	Scrophularia valida Grau	Scrophulariaceae	Irano-Turanian	Ko	Hemicryptophyte	YES
2334	Scrophularia xylobasis Rech.f.	Scrophulariaceae	Irano-Turanian	Za	Hemicryptophyte	YES
2335	Scutellaria bornmuelleri Hausskn. ex Bornm.	Lamiaceae	Irano-Turanian	Az	Chamaephyte	NO
2336	Scutellaria farsistanica Rech.f.	Lamiaceae	Irano-Turanian	Za	Chamaephyte	NO
2337	Scutellaria fragillima Rech.f.	Lamiaceae	Irano-Turanian	Za	Chamaephyte	YES
2338	Scutellaria glechomoides Boiss.	Lamiaceae	Irano-Turanian	Al, Za	Chamaephyte	NO
2339	Scutellaria multicaulis Boiss.	Lamiaceae	Irano-Turanian	Az, Za, Ke	Chamaephyte	NO
2340	Scutellaria nepetifolia Benth.	Lamiaceae	Irano-Turanian	Az, Za, Al	Chamaephyte	NO
2341	Scutellaria persica Bornm.	Lamiaceae	Irano-Turanian	Za	Chamaephyte	NO
2342	Scutellaria pinnatifida A.Ham.	Lamiaceae	Irano-Turanian	Az, Za, Al, Ko	Chamaephyte	NO
2343	Scutellaria ramazanica Parsa	Lamiaceae	Irano-Turanian	Az	Chamaephyte	YES
2344	Scutellaria szovitziana Bunge	Lamiaceae	Irano-Turanian	Az	Chamaephyte	YES
2345	Scutellaria theobromina Rech.f.	Lamiaceae	Irano-Turanian	Az	Chamaephyte	NO
2346	Scutellaria xylorrhiza Bornm.	Lamiaceae	Irano-Turanian	Za	Chamaephyte	YES
2347	Secale ciliatiglume (Boiss.) Grossh.	Poaceae	Irano-Turanian	Za	Hemicryptophyte	YES
2348	Sedum callichroum Boiss.	Crassulaceae	Irano-Turanian	Za	Therophyte	YES
2349	Sedum elburzense Akhiani & Assadi	Crassulaceae	Irano-Turanian	Az, Al	Therophyte	YES
2350	Sedum kotschyanum Boiss.	Crassulaceae	Irano-Turanian	Za, Ke	Therophyte	NO
2351	Semenovia dichotoma (Boiss.) Manden.	Apiaceae	Irano-Turanian	Za	Hemicryptophyte	NO
2352	Semenovia frigida (Boiss. & Hausskn.) Manden.	Apiaceae	Irano-Turanian	Za, Ke	Hemicryptophyte	NO
2353	Semenovia subscaposa (Rech.f.) Alava	Apiaceae	Irano-Turanian	Al, Ko	Chamaephyte	NO
2354	Semenovia suffruticosa (Freyn & Bornm.) Manden.	Apiaceae	Irano-Turanian	Ke, Lo	Chamaephyte	YES
2355	Semenovia tragioides (Boiss.) Manden.	Apiaceae	Irano-Turanian	Az, Za, Al, Ko	Hemicryptophyte	NO

2356	Sempervivum atropatanum J.Parn.	Crassulaceae	Irano-Turanian	Az	Chamaephyte	YES
2357	Sempervivum iranicum Bornm. & Gauba	Crassulaceae	Irano-Turanian	Az, Al	Chamaephyte	NO
2358	Senecio eligulatus B.Nord., Moussavi & Djavadi	Asteraceae	Irano-Turanian	Ke	Therophyte	YES
2359	Senecio iranicus B.Nord.	Asteraceae	Irano-Turanian	Al	Therophyte	YES
2360	Senecio joharchii F.Ghahrem., Ezazi, Rahch. & Attar	Asteraceae	Irano-Turanian	Ko	Hemicryptophyte	YES
2361	Senecio kotschyanus Boiss.	Asteraceae	Irano-Turanian	Za	Therophyte	YES
2362	Senecio lipskyi Lomakin	Asteraceae	Irano-Turanian	Az	Hemicryptophyte	YES
2363	Senecio subnivalis Ajani, Noroozi & Nord.	Asteraceae	Irano-Turanian	Ke	Therophyte	YES
2364	Senecio vulcanicus Boiss.	Asteraceae	Irano-Turanian	Al	Hemicryptophyte	NO
2365	Serratula bachtiarica Boiss. & Hausskn. ex Boiss.	Asteraceae	Irano-Turanian	Za	Hemicryptophyte	YES
2366	Serratula calcarea Mozaff.	Asteraceae	Irano-Turanian	Az	Hemicryptophyte	YES
2367	Serratula gracillima Rech.f.	Asteraceae	Irano-Turanian	Al	Hemicryptophyte	YES
2368	Serratula grandifolia P.H.Davis	Asteraceae	Irano-Turanian	Za	Hemicryptophyte	YES
2369	Serratula haussknechtii Boiss.	Asteraceae	Irano-Turanian	Al, Za	Hemicryptophyte	NO
2370	Serratula melanocheila Boiss. & Hausskn. ex Boiss.	Asteraceae	Irano-Turanian	Za	Hemicryptophyte	YES
2371	Serratula suffulta Rech.f.	Asteraceae	Irano-Turanian	Za	Hemicryptophyte	YES
2372	Serratula viciifolia Boiss. & Hausskn.	Asteraceae	Irano-Turanian	Az, Za	Hemicryptophyte	YES
2373	Silene albescens Boiss.	Caryophyllaceae	Irano-Turanian	Za	Chamaephyte	NO
2374	Silene avromana Boiss. & Hausskn.	Caryophyllaceae	Irano-Turanian	Az, Za, Al, Ko	Chamaephyte	NO
2375	Silene caroli-henrici Melzh.	Caryophyllaceae	Irano-Turanian	Za	Hemicryptophyte	YES
2376	Silene daenensis Melzh.	Caryophyllaceae	Irano-Turanian	Za, Ke	Hemicryptophyte	YES
2377	Silene demawendica Bornm.	Caryophyllaceae	Irano-Turanian	Al	Hemicryptophyte	YES
2378	Silene dschuparensis Bornm.	Caryophyllaceae	Irano-Turanian	Ke	Chamaephyte	YES
2379	Silene elymaitica Bornm.	Caryophyllaceae	Irano-Turanian	Za	Chamaephyte	NO
2380	Silene erysimifolia Stapf	Caryophyllaceae	Irano-Turanian	Az, Za, Al	Chamaephyte	NO
2381	Silene farsistanica Melzh.	Caryophyllaceae	Irano-Turanian	Za	Chamaephyte	NO
2382	Silene ferdowsii Joharchi, Nejati & F.Ghahrem.	Caryophyllaceae	Irano-Turanian	Ko	Chamaephyte	YES
2383	Silene gaubae Bornm. & Gauba	Caryophyllaceae	Irano-Turanian	Al	Chamaephyte	YES
2384	Silene gertraudiae Melzh.	Caryophyllaceae	Irano-Turanian	Ko, Za	Chamaephyte	NO
2385	Silene goniocaula Boiss.	Caryophyllaceae	Irano-Turanian	Az, Za	Hemicryptophyte	NO
2386	Silene gynodioica Ghaz.	Caryophyllaceae	Irano-Turanian	Az, Za, Al, Ke,	Chamaephyte	NO

				Ko		
2387	Silene hirticalyx Boiss. & Hausskn.	Caryophyllaceae	Irano-Turanian	Za	Chamaephyte	YES
2388	Silene lineata Boiss. & Buhse	Caryophyllaceae	Irano-Turanian		Chamaephyte	YES
2389	Silene lucida Chowdhuri	Caryophyllaceae	Irano-Turanian	Az	Hemicryptophyte	YES
2390	Silene meyeri Fenzl ex Boiss. & Buhse	Caryophyllaceae	Irano-Turanian	Az, Al	Hemicryptophyte	NO
2391	Silene mishudaghensis Gholipour & Parsa Khanghah	Caryophyllaceae	Irano-Turanian	Za	Hemicryptophyte	YES
2392	Silene nizvana Melzh.	Caryophyllaceae	Irano-Turanian	Al	Chamaephyte	YES
2393	Silene nurensis Boiss. & Hausskn.	Caryophyllaceae	Irano-Turanian	Za, Ke	Hemicryptophyte	NO
2394	Silene oligophylla Melzh.	Caryophyllaceae	Irano-Turanian	Az	Chamaephyte	YES
2395	Silene palinotricha Fenzl ex Boiss.	Caryophyllaceae	Irano-Turanian	Al	Chamaephyte	YES
2396	Silene parrowiana Boiss. & Hausskn.	Caryophyllaceae	Irano-Turanian	Za	Chamaephyte	YES
2397	Silene persepolitana Melzh.	Caryophyllaceae	Irano-Turanian	Za	Hemicryptophyte	YES
2398	Silene persica Boiss.	Caryophyllaceae	Irano-Turanian	Za	Hemicryptophyte	NO
2399	Silene pravitziana Rech.f.	Caryophyllaceae	Irano-Turanian	Za	Chamaephyte	YES
2400	Silene pseudaucheriana Melzh.	Caryophyllaceae	Irano-Turanian	Az, Za	Chamaephyte	YES
2401	Silene pseudonurensis Melzh.	Caryophyllaceae	Irano-Turanian	Za	Chamaephyte	YES
2402	Silene rasvandica Melzh.	Caryophyllaceae	Irano-Turanian	Za	Chamaephyte	YES
2403	Silene renzii Melzh.	Caryophyllaceae	Irano-Turanian		Chamaephyte	YES
2404	Silene sciaphila Melzh. & Rech.f.	Caryophyllaceae	Saharo-Sindian	Za	Chamaephyte	YES
2405	Silene shahrudensis Rech.f.	Caryophyllaceae	Irano-Turanian	Al	Chamaephyte	YES
2406	Silene sojakii Melzh.	Caryophyllaceae	Irano-Turanian	Al	Chamaephyte	YES
2407	Silene stapfii Melzh.	Caryophyllaceae	Irano-Turanian	Ke	Hemicryptophyte	YES
2408	Silene tragacantha Fenzl ex Boiss.	Caryophyllaceae	Irano-Turanian	Za	Chamaephyte	YES
2409	Sisymbrium gaubae Rech. f. & Bornm.	Brassicaceae	Irano-Turanian	Al	Hemicryptophyte	YES
2410	Sisymbrium kermanicum Khodash. & Mirtadz.	Brassicaceae	Irano-Turanian	Ke	Therophyte	YES
2411	Solenanthus bakhtiaricus Khat.	Boraginaceae	Irano-Turanian	Za	Hemicryptophyte	YES
2412	Sorbus luristanica (Bornm.) Schönb.-Tem.	Rosaceae	Irano-Turanian	Za	Phanerophyte	YES
2413	Sorbus tiliifolia H.Zare, Amini & Assadi	Rosaceae	Irano-Turanian	Al	Phanerophyte	YES
2414	Spiraea sheikhii H.Zare	Rosaceae	Euro-Siberian		Phanerophyte	YES
2415	Stachys acerosa Boiss.	Lamiaceae	Irano-Turanian	Za, Ke	Chamaephyte	NO
2416	Stachys asterocalyx Rech.f.	Lamiaceae	Irano-Turanian	Za, Ke	Chamaephyte	NO

2417	Stachys aucheri Benth.	Lamiaceae	Irano-Turanian	Za	Chamaephyte	NO
2418	Stachys ixodes Boiss. & Hausskn.	Lamiaceae	Irano-Turanian	Za	Chamaephyte	NO
2419	Stachys kermanshahensis Rech.f.	Lamiaceae	Irano-Turanian	Za	Chamaephyte	NO
2420	Stachys koelzii Rech.f.	Lamiaceae	Irano-Turanian	Za	Chamaephyte	YES
2421	Stachys lanigera (Bornm.) Rech.f.	Lamiaceae	Irano-Turanian	Az, Za	Hemicryptophyte	YES
2422	Stachys laxa Boiss. & Buhse	Lamiaceae	Irano-Turanian, Euro-Siberian	Al	Chamaephyte	NO
2423	Stachys lurestanica Jamzad	Lamiaceae	Irano-Turanian	Za	Chamaephyte	YES
2424	Stachys obtusicrena Boiss.	Lamiaceae	Irano-Turanian	Za, Ke	Chamaephyte	NO
2425	Stachys persepolitana Boiss.	Lamiaceae	Irano-Turanian	Za, Ke	Therophyte	NO
2426	Stachys pilifera Benth.	Lamiaceae	Irano-Turanian	Za	Chamaephyte	NO
2427	Stachys subaphylla Rech.f.	Lamiaceae	Irano-Turanian, Euro-Siberian	Al	Chamaephyte	YES
2428	Stachys veroniciformis Rech.f.	Lamiaceae	Irano-Turanian	Za	Chamaephyte	NO
2429	Stellaria scaturiginella Rech.f.	Caryophyllaceae	Irano-Turanian	Al	Therophyte	YES
2430	Stelleropsis iranica Pobed.	Thymelaeaceae	Irano-Turanian	Al, Ko	Chamaephyte	YES
2431	Stenotaenia elbursensis Bornm.	Apiaceae	Irano-Turanian	Al	Hemicryptophyte	YES
2432	Stenotaenia haussknechtii Boiss.	Apiaceae	Irano-Turanian	Za	Hemicryptophyte	YES
2433	Stenotaenia nudicaulis Boiss.	Apiaceae	Irano-Turanian	Az, Al	Hemicryptophyte	NO
2434	Sterigmostemum longistylum (Boiss.) Kuntze	Brassicaceae	Irano-Turanian	Al, Za, Ke	Therophyte	NO
2435	Stipa atriseta Stapf ex Bor	Poaceae	Irano-Turanian	Za	Hemicryptophyte	NO
2436	Stipa gaubae Bor	Poaceae	Irano-Turanian	Al	Hemicryptophyte	YES
2437	Stipa haussknechtii Boiss.	Poaceae	Irano-Turanian	Za, Ke	Hemicryptophyte	NO
2438	Straussiella purpurea (Bunge ex Boiss.) Hausskn.	Brassicaceae	Irano-Turanian	Za	Chamaephyte	NO
2439	Stroganowia persica N.Busch	Brassicaceae	Irano-Turanian	Za	Hemicryptophyte	YES
2440	Suaeda iranshahrii Akhani & Freitag	Chenopodiaceae	Saharo-Sindian		Therophyte	YES
2441	Suaeda khalijefarsica Akhani	Chenopodiaceae	Saharo-Sindian		Therophyte	YES
2442	Tamarix serotina Bunge ex Boiss.	Tamaricaceae	Irano-Turanian	Al, Lo	Phanerophyte	YES
2443	Tanacetum archibaldii Podlech	Asteraceae	Irano-Turanian	Al	Chamaephyte	YES
2444	Tanacetum bachtiaricum Mozaff.	Asteraceae	Irano-Turanian	Za	Chamaephyte	YES
2445	Tanacetum budjnurdense (Rech.f.) Tzvelev	Asteraceae	Irano-Turanian		Hemicryptophyte	YES
2446	Tanacetum dumosum Boiss.	Asteraceae	Irano-Turanian	Za	Chamaephyte	NO

2447	Tanacetum elbursense Mozaff.	Asteraceae	Irano-Turanian	Al	Chamaephyte	YES
2448	Tanacetum hololeucum (Bornm.) Podlech	Asteraceae	Irano-Turanian	Al	Chamaephyte	NO
2449	Tanacetum joharchii Sonboli & Kaz.Osaloo	Asteraceae	Irano-Turanian	Ko	Chamaephyte	YES
2450	Tanacetum khorassanicum (Krasch.) Parsa	Asteraceae	Irano-Turanian	Ko	Chamaephyte	NO
2451	Tanacetum lingulatum (Boiss.) Bornm.	Asteraceae	Irano-Turanian	Al, Za, Ke	Chamaephyte	NO
2452	Tanacetum paradoxum Bornm.	Asteraceae	Irano-Turanian	Za	Chamaephyte	YES
2453	Tanacetum persicum (Boiss.) Mozaff.	Asteraceae	Irano-Turanian	Az, Za, Ke, Ko, Al	Chamaephyte	NO
2454	Tanacetum salsugineum Podlech	Asteraceae	Irano-Turanian	Az	Chamaephyte	YES
2455	Tanacetum sonbolii Mozaff.	Asteraceae	Irano-Turanian	Az	Chamaephyte	YES
2456	Tanacetum stapfianum (Rech.f.) Podlech	Asteraceae	Irano-Turanian	Za	Chamaephyte	YES
2457	Tanacetum tarighii Sonboli	Asteraceae	Irano-Turanian	Az	Chamaephyte	YES
2458	Tanacetum tenuisectum (Boiss.) Podlech	Asteraceae	Irano-Turanian	Al	Chamaephyte	NO
2459	Tanacetum trifoliolatum Podlech	Asteraceae	Irano-Turanian	Az	Hemicryptophyte	YES
2460	Tanacetum turcomanicum (Krasch.) Tzvelev	Asteraceae	Irano-Turanian	Ko	Chamaephyte	YES
2461	Tanacetum walteri (C.Winkl.) Tzvelev	Asteraceae	Irano-Turanian	Ko	Chamaephyte	YES
2462	Taraxacum azerbaijanicum Soest	Asteraceae	Irano-Turanian	Az, Al	Hemicryptophyte	NO
2463	Taraxacum baluchistanicum Soest	Asteraceae	Saharo-Sindian		Hemicryptophyte	YES
2464	Taraxacum darbandense Soest	Asteraceae	Irano-Turanian	Al	Hemicryptophyte	YES
2465	Taraxacum hepaticolor Soest	Asteraceae	Irano-Turanian	Za	Hemicryptophyte	YES
2466	Taraxacum hydrophilum Soest	Asteraceae	Irano-Turanian	Ko	Hemicryptophyte	YES
2467	Taraxacum iranicum Soest	Asteraceae	Irano-Turanian	Al, Az, Ko	Hemicryptophyte	NO
2468	Taraxacum kalchanicum Soest	Asteraceae	Irano-Turanian	Az	Hemicryptophyte	YES
2469	Taraxacum koelzii Soest	Asteraceae	Irano-Turanian	Az, Al	Hemicryptophyte	YES
2470	Taraxacum kotschyi Soest	Asteraceae	Irano-Turanian	Za	Hemicryptophyte	YES
2471	Taraxacum leonardii Soest	Asteraceae	Irano-Turanian	Ko	Hemicryptophyte	YES
2472	Taraxacum neospurium Soest	Asteraceae	Irano-Turanian	Al, Za	Hemicryptophyte	YES
2473	Taraxacum plicatulum Soest	Asteraceae	Irano-Turanian	Za	Hemicryptophyte	YES
2474	Taraxacum primigenium Hand.-Mazz.	Asteraceae	Irano-Turanian	Az, Ke	Hemicryptophyte	YES
2475	Taraxacum rechingeri Soest	Asteraceae	Irano-Turanian	Al, Za	Hemicryptophyte	NO
2476	Taraxacum roseum Bornm.	Asteraceae	Irano-Turanian	Al, Za, Ko, Ke	Hemicryptophyte	NO

2477	Taraxacum ruberuliforme Soest	Asteraceae	Irano-Turanian	Az, Al	Hemicryptophyte	YES
2478	Taraxacum subecorniculatum Gilli	Asteraceae	Irano-Turanian	Az	Hemicryptophyte	YES
2479	Taraxacum vagum Soest	Asteraceae	Irano-Turanian	Al	Hemicryptophyte	YES
2480	Taverniera echinata Mozaff.	Fabaceae	Saharo-Sindian		Chamaephyte	YES
2481	Telephium eriglaucum F.N.Williams	Caryophyllaceae	Irano-Turanian	Za, Ke	Chamaephyte	NO
2482	Tetrataenium lasiopetalum (Boiss.) Manden.	Apiaceae	Irano-Turanian	Az, Za	Hemicryptophyte	NO
2483	Tetrataenium nephrophyllum (Leute) Manden.	Apiaceae	Irano-Turanian	Za	Hemicryptophyte	YES
2484	Teucrium macrum Boiss. & Hausskn.	Lamiaceae	Irano-Turanian	Za	Chamaephyte	YES
2485	Teucrium persicum Boiss.	Lamiaceae	Saharo-Sindian		Chamaephyte	NO
2486	Thalictrum mazandaranicum Pakravan & Assadi	Ranunculaceae	Irano-Turanian	Al	Hemicryptophyte	YES
2487	Thalictrum tacabicum Pakravan & Assadi	Ranunculaceae	Irano-Turanian	Az	Hemicryptophyte	YES
2488	Thecocarpus meifolius Boiss.	Apiaceae	Irano-Turanian	Za, Ke	Hemicryptophyte	NO
2489	Thlaspi inhumile Ponert	Brassicaceae	Irano-Turanian	Al	Hemicryptophyte	YES
2490	Thlaspi kurdicum Hedge	Brassicaceae	Irano-Turanian	Az	Hemicryptophyte	YES
2491	Thlaspi maassoumii Mozaff.	Brassicaceae	Irano-Turanian	Al	Hemicryptophyte	YES
2492	Thlaspi pulvinata Mozaff.	Brassicaceae	Irano-Turanian	Az, Ke	Hemicryptophyte	YES
2493	Thlaspi stenocarpum (Boiss.) Hedge	Brassicaceae	Irano-Turanian	Al	Hemicryptophyte	NO
2494	Thlaspi tenue (Boiss. & Buhse) Hedge	Brassicaceae	Irano-Turanian	Az	Hemicryptophyte	YES
2495	Thymus carmanicus Jalas	Lamiaceae	Irano-Turanian	Az, Al, Za, Ke	Hemicryptophyte	NO
2496	Thymus daenensis Celak.	Lamiaceae	Irano-Turanian	Az, Za, Al, Ke	Chamaephyte	NO
2497	Thymus marandensis Jamzad	Lamiaceae	Irano-Turanian	Az	Chamaephyte	YES
2498	Thymus persicus (Ronniger ex Rech.f.) Jalas	Lamiaceae	Irano-Turanian	Az	Chamaephyte	YES
2499	Thymus pubescens Boiss. & Kotschy ex Celak.	Lamiaceae	Irano-Turanian	Az, Al, Za, Ko	Hemicryptophyte	NO
2500	Tilia sabetii H.Zare	Tiliaceae	Euro-Siberian	Al	Phanerophyte	YES
2501	Tilia stellatopilosa H.Zare, Amini & Assadi	Tiliaceae	Euro-Siberian	Al	Phanerophyte	YES
2502	Trachydium depressum Boiss.	Apiaceae	Irano-Turanian	Az, Za, Al, Ke	Hemicryptophyte	NO
2503	Trachydium eriocarpum Bornm. & Gauba	Apiaceae	Irano-Turanian	Al	Hemicryptophyte	YES
2504	Trachydium kotschyi Boiss.	Apiaceae	Irano-Turanian	Za	Hemicryptophyte	NO
2505	Trachydium pauciradiatum (Boiss. & Hohen.) Rech.f.	Apiaceae	Irano-Turanian	Al	Hemicryptophyte	NO
2506	Tragopogon acanthocarpus Boiss.	Asteraceae	Irano-Turanian	Al, Za, Az	Hemicryptophyte	NO
2507	Tragopogon bakhtiaricus Rech.f.	Asteraceae	Irano-Turanian	Za	Hemicryptophyte	YES

2508	Tragopogon caricifolius Boiss.	Asteraceae	Irano-Turanian	Az, Al, Za, Ke	Hemicryptophyte	NO
2509	Tragopogon erostris Boiss. & Hausskn.	Asteraceae	Irano-Turanian	Za	Hemicryptophyte	YES
2510	Tragopogon gongylorrhizus Rech.f.	Asteraceae	Euro-Siberian		Hemicryptophyte	NO
2511	Tragopogon jesdianus Boiss. & Buhse	Asteraceae	Irano-Turanian	Al, Za, Ke, Ko	Hemicryptophyte	NO
2512	Tragopogon kotschyi Boiss.	Asteraceae	Irano-Turanian	Az, Al	Hemicryptophyte	NO
2513	Tragopogon kurdicus Safavi & Maroofi	Asteraceae	Irano-Turanian	Za	Hemicryptophyte	YES
2514	Tragopogon maturatus Boriss.	Asteraceae	Irano-Turanian	Al	Therophyte	YES
2515	Tragopogon porphyrocephalus Rech.f.	Asteraceae	Irano-Turanian	Az, Za, Al	Hemicryptophyte	NO
2516	Tragopogon rezaiyensis Rech.f.	Asteraceae	Irano-Turanian	Az, Za	Hemicryptophyte	NO
2517	Tragopogon stroterocarpus Rech.f.	Asteraceae	Irano-Turanian	Az, Za	Hemicryptophyte	YES
2518	Trichodesma aellenii Riedl	Boraginaceae	Irano-Turanian	Al, Ko	Hemicryptophyte	YES
2519	Trichodesma aucheri DC.	Boraginaceae	Irano-Turanian	Za, Ke	Hemicryptophyte	NO
2520	Trichodesma elymaiticum Mozaff.	Boraginaceae	Saharo-Sindian		Hemicryptophyte	YES
2521	Trichodesma macrocarpum Rech.f., Aellen & Esfand.	Boraginaceae	Saharo-Sindian		Hemicryptophyte	YES
2522	Tricholepis edmondsonii Rech.f.	Asteraceae	Irano-Turanian	Ke	Chamaephyte	YES
2523	Trifolium mazanderanicum Rech.f.	Fabaceae	Euro-Siberian		Hemicryptophyte	YES
2524	Trifolium radicosum Boiss. & Hohen.	Fabaceae	Irano-Turanian	Al, Az	Hemicryptophyte	NO
2525	Trigonella aphanoneura Rech.f.	Fabaceae	Irano-Turanian	Za	Chamaephyte	YES
2526	Trigonella bakhtiarica Ranjbar & Z.Hajmoradi	Fabaceae	Irano-Turanian	Za	Chamaephyte	YES
2527	Trigonella disperma Bornm.	Fabaceae	Irano-Turanian	Az, Za	Chamaephyte	NO
2528	Trigonella elliptica Boiss.	Fabaceae	Irano-Turanian	Al, Za, Ko	Chamaephyte	NO
2529	Trigonella latialata (Bornm.) Vassilcz.	Fabaceae	Irano-Turanian	Ke	Chamaephyte	YES
2530	Trigonella persica Boiss.	Fabaceae	Irano-Turanian, Saharo-Sindian	Za	Therophyte	NO
2531	Trigonella stenocarpa Rech.f.	Fabaceae	Irano-Turanian		Chamaephyte	YES
2532	Trigonella subenervis Rech.f.	Fabaceae	Irano-Turanian	Ko	Chamaephyte	YES
2533	Trigonella teheranica (Bornm.) Grossh.	Fabaceae	Irano-Turanian	Al	Chamaephyte	YES
2534	Trigonella yasujensis Ranjbar,Z.Hajmoradi & Karamain	Fabaceae	Irano-Turanian	Za	Chamaephyte	YES
2535	Trigonosciadium brachytaenium (Boiss.) Alava	Apiaceae	Irano-Turanian	Az, Za	Hemicryptophyte	NO
2536	Trisetum bungei Boiss.	Poaceae	Irano-Turanian	Al, Za	Hemicryptophyte	YES
2537	Tulipa harazensis Rech.f.	Liliaceae	Irano-Turanian	Al	Geophyte	YES

2538	Tulipa montana Lindl.	Liliaceae	Irano-Turanian	Al, Za	Geophyte	NO
2539	Tulipa ulophylla Wendelbo	Liliaceae	Irano-Turanian	Al	Geophyte	YES
2540	Tulipa urumiensis Stapf	Liliaceae	Irano-Turanian	Az	Geophyte	YES
2541	Tulipa wendelboi Matin & Iranshahr	Liliaceae	Euro-Siberian	Al	Geophyte	YES
2542	Typha azerbaijanensis Hamdi & Assadi	Typhaceae	Irano-Turanian	Az	Geophyte	YES
2543	Typha kalatensis Assadi & Hamdi	Typhaceae	Irano-Turanian	Ko	Geophyte	YES
2544	Ulmus boissieri Grudz.	Ulmaceae	Irano-Turanian	Za, Ke	Phanerophyte	NO
2545	Ungernia flava Boiss. & Hausskn.	Amaryllidaceae	Irano-Turanian	Az, Za	Geophyte	NO
2546	Verbascum albidiflorum Ranjbar & Nouri	Scrophulariaceae	Irano-Turanian	Za	Hemicryptophyte	YES
2547	Verbascum aucheri (Boiss.) Hub.-Mor.	Scrophulariaceae	Irano-Turanian	Az, Al	Hemicryptophyte	NO
2548	Verbascum austroiranicum Hub.-Mor.	Scrophulariaceae	Irano-Turanian	Za	Hemicryptophyte	YES
2549	Verbascum azerbaijanense Sharifnia & Assadi	Scrophulariaceae	Irano-Turanian	Az	Hemicryptophyte	YES
2550	Verbascum bornmuellerianum Hub.-Mor.	Scrophulariaceae	Irano-Turanian	Az, Za	Hemicryptophyte	YES
2551	Verbascum carmanicum (Bornm.) Hub.-Mor.	Scrophulariaceae	Irano-Turanian	Ke	Hemicryptophyte	NO
2552	Verbascum disjectum (Murb.) Hub.-Mor.	Scrophulariaceae	Irano-Turanian		Hemicryptophyte	NO
2553	Verbascum farsistanicum (Murb.) Hub.-Mor.	Scrophulariaceae	Irano-Turanian, Saharo-Sindian		Hemicryptophyte	NO
2554	Verbascum gabrieliae (Bornm.) Hub.-Mor.	Scrophulariaceae	Irano-Turanian	Ke, Lo	Hemicryptophyte	YES
2555	Verbascum hasarense Freyn & Bornm.	Scrophulariaceae	Irano-Turanian	Za, Ke	Hemicryptophyte	YES
2556	Verbascum haussknechtianum Hub.-Mor.	Scrophulariaceae	Irano-Turanian	Za	Hemicryptophyte	NO
2557	Verbascum intricatum Kuntze	Scrophulariaceae	Irano-Turanian	Za	Hemicryptophyte	NO
2558	Verbascum kermanense Hub.-Mor.	Scrophulariaceae	Irano-Turanian	Ke, Lo	Hemicryptophyte	YES
2559	Verbascum kochiiforme Boiss. & Hausskn.	Scrophulariaceae	Saharo-Sindian		Hemicryptophyte	NO
2560	Verbascum lyprocarpum (Murb.) Hub.-Mor.	Scrophulariaceae	Irano-Turanian	Za, Ke	Hemicryptophyte	YES
2561	Verbascum scoparium Mozaff.	Scrophulariaceae	Irano-Turanian	Ke	Hemicryptophyte	YES
2562	Verbascum shahsavarensis Sotoodeh, Attar & Civeyrel	Scrophulariaceae	Euro-Siberian	Al	Hemicryptophyte	YES
2563	Verbascum straussii (Bornm.) Hub.-Mor.	Scrophulariaceae	Irano-Turanian	Za	Hemicryptophyte	NO
2564	Verbascum sublobatum Murb.	Scrophulariaceae	Irano-Turanian, Euro-Siberian	Al	Hemicryptophyte	NO
2565	Veronica acrotheca Bornm. & Gauba	Scrophulariaceae	Irano-Turanian	Al, Za	Hemicryptophyte	NO
2566	Veronica aucheri Boiss.	Scrophulariaceae	Irano-Turanian	Al	Hemicryptophyte	NO
2567	Veronica bungei Boiss.	Scrophulariaceae	Irano-Turanian	Al	Therophyte	YES

2568	Veronica chionantha Bornm.	Scrophulariaceae	Irano-Turanian	Al	Chamaephyte	YES
2569	Veronica daranica Saeidi & Ghahr.	Scrophulariaceae	Irano-Turanian	Za	Hemicryptophyte	YES
2570	Veronica euphrasiifolia Link	Scrophulariaceae	Irano-Turanian	Al	Hemicryptophyte	YES
2571	Veronica farinosa Hausskn.	Scrophulariaceae	Irano-Turanian	Za	Hemicryptophyte	NO
2572	Veronica fragilis Boiss. & Hausskn.	Scrophulariaceae	Irano-Turanian	Za	Chamaephyte	NO
2573	Veronica francispetae M.A.Fisch.	Scrophulariaceae	Euro-Siberian	Al	Therophyte	NO
2574	Veronica kopetdaghensis B.Fedtsch. ex Boriss.	Scrophulariaceae	Irano-Turanian	Ko	Hemicryptophyte	YES
2575	Veronica kurdica Benth.	Scrophulariaceae	Irano-Turanian	Za, Al, Ke	Hemicryptophyte	NO
2576	Veronica longipedicellata Saeidi	Scrophulariaceae	Irano-Turanian	Al	Therophyte	YES
2577	Veronica mazanderanae Wendelbo	Scrophulariaceae	Irano-Turanian, Euro-Siberian	Al, Az	Therophyte	NO
2578	Veronica mirabilis Wendelbo	Scrophulariaceae	Irano-Turanian	Al	Hemicryptophyte	YES
2579	Veronica paederotae Boiss.	Scrophulariaceae	Irano-Turanian	Al	Hemicryptophyte	NO
2580	Veronica rechingeri M.A.Fisch.	Scrophulariaceae	Irano-Turanian	Al	Hemicryptophyte	NO
2581	Veronica rubrifolia Boiss.	Scrophulariaceae	Irano-Turanian	Al, Za, Ke	Therophyte	NO
2582	Vicia aucheri Jaub. & Spach	Fabaceae	Irano-Turanian	Al	Hemicryptophyte	YES
2583	Vicia ciceroidea Boiss.	Fabaceae	Irano-Turanian	Al, Za, Az	Hemicryptophyte	NO
2584	Vicia kotschyana Boiss.	Fabaceae	Irano-Turanian	Za	Hemicryptophyte	YES
2585	Vicia kurdica Jalilian	Fabaceae	Irano-Turanian	Za	Therophyte	YES
2586	Vicia persica Boiss.	Fabaceae	Irano-Turanian	Az, Al	Hemicryptophyte	NO
2587	Vicia sojakii Chrtkova	Fabaceae	Irano-Turanian	Al	Hemicryptophyte	YES
2588	Vincetoxicum assadii Zaeifi	Asclepiadaceae	Irano-Turanian	Za	Chamaephyte	YES
2589	Vincetoxicum mozaffarianii Zaeifi	Asclepiadaceae	Irano-Turanian	Ke	Chamaephyte	YES
2590	Viola pachyrrhiza Boiss. & Hohen.	Violaceae	Irano-Turanian	Az	Hemicryptophyte	YES
2591	Viola spathulata Willd. ex Schult.	Violaceae	Irano-Turanian	Al	Hemicryptophyte	NO
2592	Zeravschania aucheri (Boiss.) Pimenov	Apiaceae	Irano-Turanian	Az, Al, Za, Ko	Hemicryptophyte	NO
2593	Zeravschania membranacea (Boiss.) Pimenov	Apiaceae	Irano-Turanian	Az, Al, Za	Hemicryptophyte	NO
2594	Zerdana anchonioides Boiss.	Brassicaceae	Irano-Turanian	Za, Ke	Hemicryptophyte	NO
2595	Zeugandra iranica P.H.Davis	Campanulaceae	Irano-Turanian	Za	Chamaephyte	NO
2596	Zhumeria majdae Rech.f. & Wendelbo	Lamiaceae	Saharo-Sindian		Chamaephyte	NO
2597	Zosima radians Boiss. & Hohen.	Apiaceae	Irano-Turanian	Al, Za	Hemicryptophyte	NO

Table S2. The list of new vascular plant species published after Flora Iranica[83] and Flora of Iran[84].

Nr	Species	Family	Reference
1	Acantholimon albocalycinum Assadi & Mirtadz.	Plumbaginaceae	Assadi & Mirtadzadini (2006) Iran. J. Bot. 11(2) 129-136.
2	Acantholimon bakhtiaricum Assadi	Plumbaginaceae	Assadi (2003) Iran. J. Bot. 10(1) 25-29.
3	Acantholimon densiflorum Assadi	Plumbaginaceae	Assadi (2005) Iran. J. Bot. 11(1) 31-39.
4	Acantholimon flabellum Assadi	Plumbaginaceae	Assadi (2005) Iran. J. Bot. 11(1) 31-39.
5	Acantholimon glabratum Assadi	Plumbaginaceae	Assadi (2005) Iran. J. Bot. 11(1) 31-39.
6	Acantholimon hormozganense Assadi	Plumbaginaceae	Assadi (2004) Iran. J. Bot. 10(2) 153-157.
7	Acantholimon kermanense Assadi & Mirtadz.	Plumbaginaceae	Assadi & Mirtadzadini (2006) Iran. J. Bot. 11(2) 129-136.
8	Acantholimon mirtadzadinii Assadi	Plumbaginaceae	Assadi (2003) Iran. J. Bot. 10(1) 25-29.
9	Acantholimon moradii Assadi	Plumbaginaceae	Assadi (2005) Iran. J. Bot. 11(1) 31-39.
10	Acantholimon sirchense Assadi & Mirtadz.	Plumbaginaceae	Assadi & Mirtadzadini (2006) Iran. J. Bot. 11(2) 129-136.
11	Acantholimon zaeifii Assadi	Plumbaginaceae	Assadi (2004) Iran. J. Bot. 10(2) 153-157.
12	Acanthophyllum ejtehadii Mahmoudi & Vaezi	Caryophyllaceae	Mahmoudi Shamsabad et al. (2012) Iran. J. Bot. 18(1) 59-63.
13	Acanthophyllum yasamin-nassehiae Joharchi & Pirani	Caryophyllaceae	Pirani et al. (2013) Phytotaxa 92(2) 20-24
14	Acer mazandaranicum Amini, H.Zare & Assadi	Aceraceae	Amini et al. (2008) Iran. J. Bot. 14(2) 81-86.
15	Aeluropus laciniatus Khodash.	Poaceae	Khodashenas & Joharchi (2012) Iran. J. Bot. 18(1) 64-66.
16	Aeluropus peterganicus Khodash.	Poaceae	Khodashenas (2008) Iran. J. Bot. 14(1) 13- 15.
17	Aethionema sabzevaricum Khosravi & Joharchi	Brassicaceae	Khosravi et al. (2011) Iran. J. Bot. 17(1) 119-124.
18	Aethionema semnanensis Mozaff.	Brassicaceae	Mozaffarian (1996) Iran. J. Bot. 7(1) 127-142.
19	Agrostis gariana Taheri	Poaceae	Taheri (2013) Iran. J. Bot. 19(1) 40-43.
20	Ajuga saxicola Assadi & Jamzad	Lamiaceae	Jamzad & Assadi (1984) Iran. J. Bot. 2(2) 95-102.
21	Alcea assadii Pakravan	Malvaceae	Pakravan (2006) Iran. J. Bot. 12(2) 183-186.
22	Alcea ghahremanii Pakravan & Assadi	Malvaceae	Pakravan & Ghahreman (2002) Iran. J. Bot. 9(2) 245-247.
23	Alcea ilamica Pakravan	Malvaceae	Pakravan (2003) Rostaniha. 4(3-4) 93-98.
24	Alcea iranshahrii Pakravan, Ghahr. & Assadi	Malvaceae	Pakravan & Ghahreman (2005) Rostaniha. 6 51-59.
25	Alcea mozaffarianii Ghahr., Pakravan & Assadi	Malvaceae	Pakravan & Ghahreman (2005) Rostaniha. 6 51-59.

26	Alcea semnanica Pakravan	Malvaceae	Pakravan (2006) Iran. J. Bot. 12(2) 183-186.
27	Allium alamutense Razyfard, Zarre & R.M.Fritsch	Alliaceae	Razyfard et al. (2011) Ann. Bot. Fennici 48, 352-360
28	Allium assadii Seisums	Alliaceae	Seisums (2000) Iran. J. Bot. 8(2).223-232.
29	Allium autumniflorum F.O.Khass. & Akhani	Alliaceae	Khassanov et al. (2006) Rostaniha 7(suppl.2) 119-129.
30	Allium chlorotepalum R.M.Fritsch & M.Jaeger	Alliaceae	Fritsch & Maroofi (2010) Phyton (Horn, Austria) 50(1) 1-32.
31	Allium esfandiarii Matin	Alliaceae	Matin (1989) Iran. J. Bot. 4(2) 165-170.
32	Allium jaegeri R.M.Fritsch	Alliaceae	Fritsch & Maroofi (2010) Phyton (Horn, Austria) 50(1) 1-32.
33	Allium joharchii F.O.Khass. & Memariani	Alliaceae	Khassanov & Memariani (2006) Rostaniha. Vol. 7(suppl. 2) 65-69.
34	Allium kuhrangense Akhavan, Saeidi & R.M.Fritsch	Alliaceae	Akhavan et al. (2014) Phytotaxa 170(3) 213-218.
35	Allium kurdistanicum Maroofi & R.M.Fritsch	Alliaceae	Razyfard et al. (2011) Ann. Bot. Fennici 48 352-360.
36	Allium longipapillatum R.M.Fritsch & Matin	Alliaceae	Fritsch et al. (2006) Rostaniha. 7(suppl.2) 255-281.
37	Allium mahneshanense Razyfard, Zarre & R.M.Fritsch	Alliaceae	Razyfard et al. (2011) Ann. Bot. Fennici 48 352-360.
38	Allium montelburzense R.M.Fritsch, Salmaki & Zarre	Alliaceae	Fritsch et al. (2006) Rostaniha. 7(suppl.2) 255-281.
39	Allium mozaffarianii Maroofi & R.M.Fritsch	Alliaceae	Fritsch & Maroofi (2010) Phyton (Horn, Austria) 50(1) 1-32.
40	Allium najafdaricum R.M.Fritsch	Alliaceae	Fritsch & Maroofi (2010) Phyton (Horn, Austria) 50(1) 1-32.
41	Allium subakaka Razyfard & Zarre	Alliaceae	Razyfard et al. (2011) Ann. Bot. Fennici 48 352-360.
42	Allium tuchalense F.O.Khass. & Noroozi	Alliaceae	Khassanov et al. (2006) Rostaniha 7(suppl.2) 119-129.
43	Allium wendelboi Matin	Alliaceae	Matin (1989) Iran. J. Bot. 4(2) 165-170.
44	Allochrusa lutea Falat. & Mahmoodi	Caryophyllaceae	Mahmoodi & Nejad Falatoury (2016) 277(2) 191-198.
45	Alyssum hezarmasjedense Kavousi & Nazary	Brassicaceae	Kavousi et al. (2014) Novon 23(1) 59-61.
46	Alyssum mozaffarianii Kavousi	Brassicaceae	Kavousi (2001) Iran. J. Bot. 9(1) 47-54.
47	Alyssum stipitatum Kavousi & T.R.Dudley	Brassicaceae	Kavousi (2001) Iran. J. Bot. 9(1) 47-54.
48	Amberboa lippii (L.) DC.	Asteraceae	Mozaffarian (1988) Iran. J. Bot. 4(1) 61-70.
49	Amberboa maroofii Negaresh	Asteraceae	Negaresh (2015) Phytotaxa 195(2) 171-177.
50	Amygdalus reticulata Runemark ex Khat.	Rosaceae	Khatamsaz (1985) Iran. J. Bot. 3(1) 77-80.
51	Amygdalus wendelboi Freitag	Rosaceae	Freitag (1977) Iran. J. Bot. 1(2) 117-120.
52	Anabasis firouzii Akhani	Chenopodiaceae	Akhani et al. (2016) Phytotaxa 249(1) 159-180.
53	Angelica urumiensis Mozaff.	Apiaceae	Mozaffarian (2013) Rostaniha 14(1) 36-42.

54	Arenaria assadii Fadaie	Caryophyllaceae	Fadaie (2013) Iran. J. Bot. 19(1) 32-39.
55	Arenaria kandavanensis Fadaie, Sheidai & Assadi	Caryophyllaceae	Fadaie et al. (201) Iran. J. Bot. 16(2) 218-220.
56	Arenaria longibracteata Fadaie	Caryophyllaceae	Fadaie (2013) Iran. J. Bot. 19(1) 32-39.
57	Arenaria semiromica Fadaie	Caryophyllaceae	Fadaie (2013) Iran. J. Bot. 19(1) 32-39.
58	Arum giganteum Ghahr.	Araceae	Ghahreman (1983) Iran. J. Bot. 2(1) 79-81.
59	Asparagus khorasanensis Hamdi & Assadi	Asparagaceae	Hamdi & Assadi (2009) Feddes Repertorium 120 (7-8) 419-425.
60	Asparagus touranensis Hamdi & Assadi	Asparagaceae	Hamdi & Assadi (2013) Iran. J. Bot. 19 (1) 44-46.
61	Asperula oppositifolia Regel & Schmalh.	Rubiaceae	Ghahremaninejad et al. (2006) Rostaniha. 7(suppl.2) 207-212.
62	Aster bachtiaricus Mozaff.	Asteraceae	Mozaffarian (2006) Iran. J. Bot. 7(1) 127-142.
63	Astragalus abharensis Maassoumi & Podlech	Fabaceae	Podlech & Maassoumi (2003) Feddes Repertorium 114(5-6) 320-351.
64	Astragalus absconditus Zarre & Podlech	Fabaceae	Zarre et al. (2005) Feddes Repertorium 116(1-2) 54- 79.
65	Astragalus affinis Podlech & Zarre	Fabaceae	Zarre et al. (2005) Feddes Repertorium 116(1-2) 54- 79.
66	Astragalus ahangarensis Zarre & Podlech	Fabaceae	Zarre et al. (2005) Feddes Repertorium 116(1-2) 54- 79.
67	Astragalus aharicus Maassoumi & Podlech	Fabaceae	Podlech & Maassoumi (1987) Iran. J. Bot. 3(2) 95-110.
68	Astragalus aliomranii Maassoumi	Fabaceae	Maassoumi (2002) Iran. J. Bot. 9(2) 121-125.
69	Astragalus altimontanus Podlech & Maassoumi	Fabaceae	Podlech & Maassoumi (2003) Feddes Repertorium 114 (5-6) 320-351.
70	Astragalus altiusculus Maassoumi & Ghahrem.	Fabaceae	Maassoumi & Ghahremaninejad (1999) Iran. J. Bot. 8(1) 35-41.
71	Astragalus ammodendroides Bornm.	Fabaceae	Podlech & Zarre (2003) Willdenowia 33 341-352.
72	Astragalus andabaddensis Maassoumi, Bagheri & F.Ghahrem.	Fabaceae	Ghahremaninejad & Bagheri (2009) Iran. J. Bot. 15(2) 175-177.
73	Astragalus anodiophilus Zarre & Podlech	Fabaceae	Zarre et al. (2005) Feddes Repertorium 116(1-2) 54- 79.
74	Astragalus ashtianensis Podlech & Maassoumi	Fabaceae	Podlech & Maassoumi (2003) Feddes Repertorium 114 (5-6) 320-351.
75	Astragalus assadii Maassoumi & Podlech	Fabaceae	Podlech & Maassoumi (1987) Iran. J. Bot. 3(2) 95-110.
76	Astragalus austromahneshanensis F.Ghahrem., Maassoumi & Bagheri	Fabaceae	Bagheri et al. (2011) Iran. J. Bot.17(1) 15-19.
77	Astragalus aznaicus Podlech & Maassoumi	Fabaceae	Podlech & Maassoumi (2003) Feddes Repertorium 114 (5-6) 320-351.
78	Astragalus babakhanloui Maassoumi & Podlech	Fabaceae	Podlech & Maassoumi (1987) Iran. J. Bot. 4(1) 71-90.

79	Astragalus baftensis Ranjbar & Maassoumi	Fabaceae	Ranjbar (2011) Iran. J. Bot. 17(1) 1-5.
80	Astragalus bavanatensis Maassoumi, Nowroozi & Podlech	Fabaceae	Maassoumi (2005) Iran. J. Bot. 11(1) 101-109.
81	Astragalus bazarganii Podlech & Zarre	Fabaceae	Podlech & Zarre (2003) Willdenowia 33 341-352.
82	Astragalus bejourensis Podlech & Maassoumi	Fabaceae	Podlech & Maassoumi (2003) Feddes Repertorium 114(5-6) 320-351.
83	Astragalus belgheisicoides Podlech & Maassoumi	Fabaceae	Podlech & Maassoumi (2003) Feddes Repertorium 114(5-6) 320-351.
84	Astragalus biarjmandicus Podlech & Zarre	Fabaceae	Podlech & Zarre (2003) Willdenowia 33 341-352.
85	Astragalus borujenensis Ranjbar & Maassoumi	Fabaceae	Ranjbar et al. (2007) Iran. J. Bot. 13(2) 82-86.
86	Astragalus bozghoushensis Maassoumi, Mozaff. & Ramezani	Fabaceae	Maassoumi et al. (2015) Iran. J. Bot. 21(2) 94-99.
87	Astragalus bukanensis Maassoumi & Podlech	Fabaceae	Podlech & Maassoumi (2003) Feddes Repertorium 114(5-6) 320-351.
88	Astragalus chaldoranicus Podlech & Maassoumi	Fabaceae	Podlech & Maassoumi (2003) Feddes Repertorium 114(5-6) 320-351.
89	Astragalus chichesticus Podlech & Maassoumi	Fabaceae	Podlech & Maassoumi (2003) Feddes Repertorium 114(5-6) 320-351.
90	Astragalus ciceropsis Hamzehee & Maassoumi	Fabaceae	Maassoumi & Hamzehee (2003) Iran. J. Bot. 10(1) 59-61.
91	Astragalus clivicola Podlech & Maassoumi	Fabaceae	Podlech & Maassoumi (2003) Feddes Repertorium 114(5-6) 320-351.
92	Astragalus controversus Maassoumi & Podlech	Fabaceae	Podlech & Maassoumi (1987) Iran. J. Bot. 3(2) 95-110.
93	Astragalus culminatus Maassoumi, Kaz.Osaloo & Joharchi	Fabaceae	Maassoumi & Kazempour osaloo (2006) Iran. Jour. Bot. 11(2) 137-142.
94	Astragalus damghanensis Podlech	Fabaceae	Zarre et al. (2005) Feddes Repertorium 116(1-2) 54- 79.
95	Astragalus darrehbidensis Podlech & Zarre	Fabaceae	Podlech & Zarre (2003) Willdenowia 33 341-352.
96	Astragalus dorudensis Zarre & Podlech	Fabaceae	Zarre et al. (2005) Feddes Repertorium 116(1-2) 54-79.
97	Astragalus doshman-ziariensis Maassoumi & Podlech	Fabaceae	Podlech & Maassoumi (1987) Iran. J. Bot. 4(1) 71-90.
98	Astragalus dysbatophilus Zarre & Podlech	Fabaceae	Zarre et al. (2005) Feddes Repertorium 116(1-2) 54-79.
99	Astragalus ebrahimabadensis Zarre & Podlech	Fabaceae	Zarre et al. (2005) Feddes Repertorium 116(1-2) 54-79.
100	Astragalus elezgensis Maassoumi & Kaz.Osaloo	Fabaceae	Maassoumi & Kazempour osaloo (2006) Iran. Jour. Bot. 11(2) 137-142.
101	Astragalus estahbanensis Maassoumi & Podlech	Fabaceae	Podlech & Maassoumi (2003) Feddes Repertorium 114(5-6) 320-351.

102	Astragalus facetus Maassoumi & Podlech	Fabaceae	Podlech & Maassoumi (1987) Iran. J. Bot. 4(1) 71-90.
103	Astragalus fagh-soleimanensis Maassoumi & Podlech	Fabaceae	Podlech & Maassoumi (2003) Feddes Repertorium 114(5-6) 320-351.
104	Astragalus gandomanicus Podlech	Fabaceae	Podlech (2009) Feddes Repertorium 120 48ï 58.
105	Astragalus ghamishluensis Dastpak, Maassoumi & Kaz.Osaloo	Fabaceae	Dastpak et al. (2011) Iran. J. Bot. 17(2) 175-177.
106	Astragalus gigantirostratus Maassoumi, Ghahr., Ghahrem. & F.Matin.	Fabaceae	Maassoumi et al. (1999) Willdenowia 29 221-225.
107	Astragalus glaucopsiformis Maassoumi	Fabaceae	Maassoumi (2000) Iran. J. Bot. 8(2) 309-326.
108	Astragalus hajiabadensis Podlech & Maassoumi	Fabaceae	Podlech & Maassoumi (2003) Feddes Repertorium 114(5-6) 320-351.
109	Astragalus hamadryadis Podlech	Fabaceae	Zarre et al. (2005) Feddes Repertorium 116(1-2) 54-79.
110	Astragalus harazensis Zarre & Podlech	Fabaceae	Zarre et al. (2005) Feddes Repertorium 116(1-2) 54-79.
111	Astragalus hecatae Zarre & Podlech	Fabaceae	Zarre et al. (2005) Feddes Repertorium 116(1-2) 54-79.
112	Astragalus homandicus Maassoumi & Podlech	Fabaceae	Podlech & Maassoumi (2003) Feddes Repertorium 114(5-6) 320-351.
113	Astragalus hotkanensis Maassoumi & Mirtadz.	Fabaceae	Maassoumi & Mirtadzadini (2006) Iran. J. Bot. 12(1) 90-91.
114	Astragalus ignotus Podlech	Fabaceae	Zarre et al. (2005) Feddes Repertorium 116(1-2) 54-79.
115	Astragalus innotabilis Podlech	Fabaceae	Zarre et al. (2005) Feddes Repertorium 116(1-2) 54-79.
116	Astragalus insularis-ashkii Maassoumi & Podlech	Fabaceae	Massoumi (2015) Iran. J. Bot. 21(2) 123-125; originally described as *A. insularis* (nomen illegit.) in Podlech & Maassoumi (2003) Feddes Repert. 114(5-6) 320-351.
117	Astragalus iranshahrii Maassoumi & Podlech	Fabaceae	Podlech & Maassoumi (1987) Iran. J. Bot. 4(1) 71-90.
118	Astragalus issatissensis Maassoumi & Mahmoodi	Fabaceae	Mahmoodi et al. (2013) Willdenowia 43 263ï 270.
119	Astragalus jamzadiae Maassoumi	Fabaceae	Maassoumi (2000) Iran. J. Bot. 8(2) 309-326.
120	Astragalus juladakensis Maassoumi	Fabaceae	Maassoumi (2007) Iran. J. Bot. 13(2) 78-81.
121	Astragalus kabutarlanensis Dehshiri & Maassoumi	Fabaceae	Maassoumi & Dehshiri (2006) Iran. J. Bot. 11(2) 193-195.
122	Astragalus kadschoroides Ranjbar	Fabaceae	Ranjbar et al. (2007) Willdenowia 37 35-312.
123	Astragalus kalatehensis Maassoumi & Kaz.Osaloo	Fabaceae	Maassoumi & Kazempour Osaloo (2006) Iran. Jour. Bot. 11(2) 137-142.
124	Astragalus karl-heinzii Maassoumi	Fabaceae	Maassoumi (2007) Iran. J. Bot. 13(2) 78-81.
125	Astragalus kashmarensis Maassoumi & Podlech	Fabaceae	Podlech & Maassoumi (1987) Iran. J. Bot. 3(2) 95-110.

126	Astragalus khadem-kandicus Maassoumi & Podlech	Fabaceae	Podlech & Maassoumi (2003) Feddes Repertorium 114(5-6) 320-351.
127	Astragalus khatamsaziae Maassoumi	Fabaceae	Maassoumi (2000) Iran. J. Bot. 8(2) 309-326.
128	Astragalus khongensis Maassoumi, Joharchi & Podlech	Fabaceae	Maassoumi (2005) Iran. J. Bot. 11(1) 101-109.
129	Astragalus khonikensis Nasseh & Joharchi	Fabaceae	Nasseh et al (2010) Iran. J. Bot. 16(2) 221-224.
130	Astragalus khunsarensis Zarre & Podlech	Fabaceae	Zarre et al. (2005) Feddes Repertorium 116(1-2) 54-79.
131	Astragalus kiamaky-daghensis Maassoumi & Podlech	Fabaceae	Podlech & Maassoumi (2003) Feddes Repertorium 114(5-6) 320-351.
132	Astragalus kiviensis Ranjbar & Rahimin.	Fabaceae	Ranjbar et al. (2007) Willdenowia 37 35-312.
133	Astragalus lacus-valashti Maassoumi, Podlech & Jalili	Fabaceae	Podlech & Maassoumi (2003) Feddes Repertorium 114(5-6) 320-351.
134	Astragalus lasiocalycinus Podlech & Maassoumi	Fabaceae	Podlech & Maassoumi (2003) Feddes Repertorium 114(5-6) 320-351.
135	Astragalus maarofii Podlech & Maassoumi	Fabaceae	Podlech & Maassoumi (2003) Feddes Repertorium 114(5-6) 320-351.
136	Astragalus magnibracteatus Maassoumi & Maroofi	Fabaceae	Maassoumi (2005) Iran. J. Bot. 11(1) 101-109.
137	Astragalus mahneshanensis Maassoumi & Moussavi	Fabaceae	Maassoumi (2005) Iran. J. Bot. 11(1) 101-109.
138	Astragalus makuensis Maassoumi, Bagheri & Rahimin.	Fabaceae	Bagheri et al. (2014) Phytotaxa 178(1) 38-42.
139	Astragalus markasicus Podlech & Maassoumi	Fabaceae	Podlech & Maassoumi (2003) Feddes Repertorium 114(5-6) 320-351.
140	Astragalus masulehensis Ranjbar & Assadi	Fabaceae	Ranjbar etal. (2005) Willdenowia 35 (1) 117-124.
141	Astragalus mehranensis Maassoumi & Mozaff.	Fabaceae	Maassoumi & Mozaffarian (2007) Iran. J. Bot. 13(2) 87-88.
142	Astragalus mehrizianus Podlech & Maassoumi	Fabaceae	Podlech & Maassoumi (2003) Feddes Repertorium 114(5-6) 320-351.
143	Astragalus meimandicus Maassoumi & Vakili	Fabaceae	Maassoumi & Vakili Shahrebabaki (2001) Iran. J. Bot. 9(1) 69-70.
144	Astragalus membranostipulus Maassoumi	Fabaceae	Maassoumi (2000) Iran. J. Bot. 8(2) 309-326.
145	Astragalus memnonius Maassoumi & Podlech	Fabaceae	Podlech & Maassoumi (1987) Iran. J. Bot. 4(1) 71-90.
146	Astragalus meshkinensis Podlech	Fabaceae	Zarre et al. (2005) Feddes Repertorium 116(1-2) 54-79.
147	Astragalus montismishoudaghi Sheikh Akbari Mehr, Ghorbani & Maassoumi	Fabaceae	Sheikh Akbari Mehr et al. (2011) Iran. J. Bot. 17(2) 178-180.
148	Astragalus montis-nacarouzii Maassoumi & Maroofi	Fabaceae	Maassoumi & Maroofi (2006) Iran. J. Bot. 12(1) 92-93.

149	Astragalus montis-parrowii Maassoumi & Nemati	Fabaceae	Maassoumi (2005) Iran. J. Bot. 11(1) 101-109.
150	Astragalus montosus Maassoumi	Fabaceae	Maassoumi (2000) Iran. J. Bot. 8(2) 309-326.
151	Astragalus musaianus Maassoumi & Joharchi	Fabaceae	Maassoumi et al. (2013) Rostaniha 14(1) 14-21.
152	Astragalus neoiranshahrii Maassoumi & Amini Rad	Fabaceae	Maassoumi et al. Rostaniha 14(1) 14-21.
153	Astragalus neomozaffarianii Maassoumi	Fabaceae	Maassoumi (2000) Iran. J. Bot. 8(2) 309-326.
154	Astragalus nezva-montis Podlech & Zarre	Fabaceae	Zarre et al. (2005) Feddes Repertorium 116(1-2) 54-79.
155	Astragalus nowroozii Podlech & Zarre	Fabaceae	Podlech & Zarre (2003) Willdenowia 33 341-352.
156	Astragalus orientopersicus F.Ghahrem., Joharchi, Fereid. & Hoseini	Fabaceae	Ghahremaninejad et al. (2016) 38(1) 29-33.
157	Astragalus pakravaniae Podlech & Maassoumi	Fabaceae	Podlech & Maassoumi (2003) Feddes Repertorium 114(5-6) 320-351.
158	Astragalus passargadensis Maassoumi	Fabaceae	Maassoumi (2000) Iran. J. Bot. 8(2) 309-326.
159	Astragalus pauxillis Maassoumi & Ghahrem.	Fabaceae	Maassoumi & Ghahremaninejad (1999) Iran. J. Bot. 8(1) 35-41.
160	Astragalus pereshkhoranicus Maassoumi & F.Ghahrem.	Fabaceae	Maassoumi & Ghahremaninejad (1999) Iran. J. Bot. 8(1) 35-41.
161	Astragalus peymanii Maassoumi	Fabaceae	Maassoumi (2000) Iran. J. Bot. 8(2) 309-326.
162	Astragalus pileh-khasehensis Podlech & Maassoumi	Fabaceae	Podlech & Maassoumi (2003) Feddes Repertorium 114(5-6) 320-351.
163	Astragalus plagiophacos Maassoumi & Podlech	Fabaceae	Podlech & Maassoumi (1987) Iran. J. Bot. 4(1) 71-90.
164	Astragalus polystachys Maassoumi	Fabaceae	Maassoumi (2000) Iran. J. Bot. 8(2) 309-326.
165	Astragalus pseudocomosus Maassoumi, F.Ghahrem. & Bagheri	Fabaceae	Maassoumi et al. (2013) Rostaniha 14(1) 14-21.
166	Astragalus pseudojohannis Maassoumi & Podlech	Fabaceae	Podlech & Maassoumi (1987) Iran. J. Bot. 3(2) 95-110.
167	Astragalus pseudorobustus Podlech & Maassoumi	Fabaceae	Podlech & Maassoumi (2003) Feddes Repertorium 114(5-6) 320-351.
168	Astragalus qaratchaicus Maassoumi, Ghahrem. & Javadi	Fabaceae	Maassoumi (2005) Iran. J. Bot. 11(1) 101-109.
169	Astragalus qeydarnabiensis Bagheri, F.Ghahrem. & Maassoumi	Fabaceae	Bagheri et al. (2011) Iran. J. Bot.17(1) 15-19.
170	Astragalus qohestanicus Nasseh & Maassoumi	Fabaceae	Nasseh et al (2010) Iran. J. Bot. 16(2) 221-224.
171	Astragalus qorvehensis Podlech	Fabaceae	Zarre et al. (2005) Feddes Repertorium 116(1-2) 54-79.
172	Astragalus razensis Nasseh & Joharchi	Fabaceae	Nasseh & Joharchi (2009) Iran. J. Bot. 15(2) 154-156.
173	Astragalus reconditus Podlech & Maassoumi	Fabaceae	Podlech & Maassoumi (2003) Feddes Repertorium 114(5-6) 320-351.

174	Astragalus remotispicatus Bagheri & Maassoumi	Fabaceae	Bagheri et al. (2016) 11(3) e0149726
175	Astragalus repentinus Ekici & Podlech	Fabaceae	Zarre et al. (2005) Feddes Repertorium 116 (1-2) 54- 79.
176	Astragalus reticulato-venosus Maassoumi & Podlech	Fabaceae	Podlech & Maassoumi (1987) Iran. J. Bot. 3(2) 95-110.
177	Astragalus rubriphysa Maassoumi & Khorrami	Fabaceae	Maassoumi (2005) Iran. J. Bot. 11(1) 101-109.
178	Astragalus rubrocalycinus Maassoumi & Podlech	Fabaceae	Podlech & Maassoumi (1987) Iran. J. Bot. 4(1) 71-90.
179	Astragalus runemarkii Maassoumi & Podlech	Fabaceae	Podlech & Maassoumi (2003) Feddes Repertorium 114(5-6) 320-351.
180	Astragalus sabetii Podlech & Maassoumi	Fabaceae	Podlech & Maassoumi (2003) Feddes Repertorium 114(5-6) 320-351.
181	Astragalus safavii Podlech & Maassoumi	Fabaceae	Podlech & Maassoumi (2003) Feddes Repertorium 114(5-6)320-351.
182	Astragalus salavatabadensis Podlech	Fabaceae	Podlech (2009) Feddes Repertorium 120 48ï 58.
183	Astragalus sarabensis Maassoumi & Podlech	Fabaceae	Podlech & Maassoumi (2003) Feddes Repertorium 114(5-6)320-351.
184	Astragalus saremii Maassoumi	Fabaceae	Maassoumi (2002) Iran. J. Bot. 9(2) 121-125.
185	Astragalus segregatus Zarre & Podlech	Fabaceae	Zarre et al. (2005) Feddes Repertorium 116 (1-2) 54- 79.
186	Astragalus semiromensis Podlech & Maassoumi	Fabaceae	Podlech & Maassoumi (2003) Feddes Repertorium 114(5-6) 320-351.
187	Astragalus semitarius Zarre & Podlech	Fabaceae	Zarre et al. (2005) Feddes Repertorium 116(1-2) 54-79.
188	Astragalus shabilensis Podlech & Maassoumi	Fabaceae	Podlech & Maassoumi (2003) Feddes Repertorium 114(5-6) 320-351.
189	Astragalus shahinii Podlech & Maassoumi	Fabaceae	Podlech & Maassoumi (2003) Feddes Repertorium 114(5-6)320-351.
190	Astragalus shahsavarii Maassoumi & Podlech	Fabaceae	Maassoumi & Podlech (2002) Iran. J. Bot. 9(2) 203-205.
191	Astragalus shehbazii Zarre & Podlech	Fabaceae	Zarre et al. (2005) Feddes Repertorium 116(1-2) 54-79.
192	Astragalus siahbishehensis Rahimin. & Ranjbar	Fabaceae	Ranjbar et al. (2005) Willdenowia 35(1) 117-124.
193	Astragalus siahcheshmehensis Maassoumi & Podlech	Fabaceae	Podlech & Maassoumi (2003) Feddes Repertorium 114(5-6) 320-351.
194	Astragalus simakanensis Maassoumi & Hatami	Fabaceae	Maassoumi (2005) Iran. J. Bot. 11(1) 101-109.
195	Astragalus sisakhtianus Podlech & Maassoumi	Fabaceae	Podlech & Maassoumi (2003) Feddes Repertorium 114(5-6) 320-351.
196	Astragalus sivendicus Podlech & Maassoumi	Fabaceae	Podlech & Maassoumi (2003) Feddes Repertorium 114(5-6) 320-351.

197	Astragalus spachianiformis Podlech & Maassoumi	Fabaceae	Podlech & Maassoumi (2003) Feddes Repertorium 114(5-6) 320-351.
198	Astragalus strictissimus Podlech & Zarre	Fabaceae	Podlech & Zarre (2003) Willdenowia 33 341-352.
199	Astragalus subaspadanus Maassoumi, F.Ghahrem. & Bagheri	Fabaceae	Maassoumi et al. (2013) Rostaniha 14(1) 14-21.
200	Astragalus subglaberrimus Podlech & Maassoumi	Fabaceae	Podlech & Maassoumi (2003) Feddes Repertorium 114(5-6) 320-351.
201	Astragalus subkohrudicus Maassoumi, F.Ghahrem. & Bagheri	Fabaceae	Bagheri et al. (2011) Iran. J. Bot.17(1) 15-19.
202	Astragalus subrecognitus Bagheri, Maassoumi & F.Ghahrem.	Fabaceae	Bagheri et al. (2011) Iran. J. Bot.17(1) 15-19.
203	Astragalus sumarensis Maassoumi	Fabaceae	Maassoumi (2000) Iran. J. Bot. 8(2) 309-326.
204	Astragalus taebiae Zarre & Podlech	Fabaceae	Zarre et al. (2005) Feddes Repertorium 116(1-2) 54-79.
205	Astragalus tahbaziae Zarre & Podlech	Fabaceae	Zarre et al. (2005) Feddes Repertorium 116(1-2) 54-79.
206	Astragalus taleshensis Bidarlord, F.Ghahrem. & Maassoumi	Fabaceae	Bidarlord et al. (2016) Phytotaxa 252(4) 280-284.
207	Astragalus tenuiramosus Podlech & Zarre	Fabaceae	Podlech & Zarre (2003) Willdenowia 33 341-352.
208	Astragalus tenuissimus Zarre & Podlech	Fabaceae	Zarre et al. (2005) Feddes Repertorium 116(1-2) 54-79.
209	Astragalus veiskaramii Zarre, Podlech & Sabaii	Fabaceae	Sabaii et al. (2007) Willdenowia 37 297-304.
210	Astragalus vessalae Maassoumi & Podlech	Fabaceae	Podlech & Maassoumi (1987) Iran. J. Bot. 4(1) 71-90.
211	Astragalus vicinalis Zarre & Podlech	Fabaceae	Zarre et al. (2005) Feddes Repertorium 116(1-2) 54-79.
212	Astragalus yazdii (Vassilcz.) Podlech & Maassoumi	Fabaceae	Maassoumi (2013) Iran. J. Bot. 19(1) 1-28.
213	Astragalus zangooeianus Maassoumi, Safavi & Nasseh	Fabaceae	Maassoumi et al. (2001) Iran. J. Bot. 9(1) 63-64.
214	Astragalus zanjanensis Podlech & Maassoumi	Fabaceae	Podlech & Maassoumi (2003) Feddes Repertorium 114(5-6) 320-351.
215	Astragalus zourabadensis Zarre & Podlech	Fabaceae	Zarre et al. (2005) Feddes Repertorium 116(1-2) 54-79.
216	Atractylis delvarii Mozaff.	Asteraceae	Mozaffarian (1996) Iran. J. Bot. 7(1) 127-142.
217	Atraphaxis binaludensis S. Tavakkoli, Mozaff. & Kaz.Osaloo	Polygonaceae	Tavakkoli et al. (2014) Iran. J. Bot. 20(1) 01-04.
218	Atraphaxis intricata Mozaff.	Rubiaceae	Mozaffarian (2006) Iran. J. Bot. 12(2) 107-113.
219	Bellevalia wendelboi Maassoumi & Jafari	Asparagaceae	Maassoumi & Jafari (2008) Edinb. J. Bot. 65 469-473.
220	Bienertia kavirense Akhani	Chenopodiaceae	Akhani et al. (2012) Plant Biosystems 146(3) 550-559.
221	Bupleurum ghahremanii Mozaff.	Apiaceae	Mozaffarian (1991) Iran. J. Bot. 5(1) 29-39.
222	Bupleurum gilanicum Mozaff.	Apiaceae	Mozaffarian (2015) Iran. J. Bot. 21(1) 83-85.
223	Calligonum alatosetosum Maassoumi & Kazempour	Polygonaceae	Maassoumi (2011) Iran. J. Bot. 17(1) 43-54.

224	Calligonum spinosetosum Maassoumi & Batooli	Polygonaceae	Maassoumi & Batooli (2009) Iran . J. Bot. 15(2) 153-153.
225	Campanula kurdistanica Advay & Maroofi	Campanulaceae	Advay & Marofi (2015) Iran. J. Bot. 21(1) 35-38.
226	Centaurea ardabilica Ranjbar & Heydari	Asteraceae	Ranjbar & Heydari (2015) Phytotaxa 201(1) 79-86.
227	Centaurea elymaitica Mozaff.	Rubiaceae	Mozaffarian (2006) Iran. J. Bot. 12(2) 107-113.
228	Centaurea kabirkuhensis Mozaff., F.Ghahrem. & Fereid.	Asteraceae	Mozaffarian (2012) Ann. Naturhist. Mus. Wien, B 114: 133-134; originally described as *C. procera* (nomen illegit.) in Mozaffarian (2010) Iran. J. Bot. 16(2) 204-212.
229	Centaurea khuzistanica Mozaff.	Asteraceae	Mozaffarian (1992) Iran. J. Bot. 5(2) 83-90.
230	Centaurea paradoxa Mozaff.	Asteraceae	Mozaffarian (1991) Iran. J. Bot. 5(1) 29-39.
231	Centaurea rahiminejadii Negaresh	Asteraceae	Negaresh & Rahiminejad (2015) Nordic J. Bot. 34(1) 15-22.
232	Centaurea ravansarensis Ranjbar & Negaresh	Asteraceae	Ranjbar et al. (2013) Nordic J. Bot. 31(4) 430-436.
233	Centaurea salmasensis Ranjbar & Heydari	Asteraceae	Ranjbar & Heydari (2016) Phytotaxa 277(2) 182-190.
234	Centaurea shahuensis Ranjbar & Negaresh	Asteraceae	Ranjbar et al. (2013) Nordic J. Bot. 31(4) 430-436.
235	Centaurea tabriziana Ranjbar & Heydari	Asteraceae	Ranjbar & Heydari (2015) Phytotaxa 201(1) 79-86.
236	Centaurea zagrosmontana Ranjbar & Heydari	Asteraceae	Ranjbar & Heydari (2016) Phytotaxa 277(2) 182-190.
237	Cephalaria bojnordensis Ranjbar & Z.Ranjbar	Dipsacaceae	Ranjbar et al. (2014) Feddes Repertorium 124 116-121.
238	Cerasus paradoxa Dehshiri & Mozaff.	Rosaceae	Dehshiri & Mozaffarian (2012) Iran. J. Bot. 18(2) 220-223.
239	Clematis iranica Habibi, Ghorbani & Azizian	Ranunculaceae	Habibi et al. (2014) Phytotaxa 16(2) 99-106.
240	Colchicum bakhtiaricum Matin & Iranshahr	Liliaceae	Matin & Iranshahr (2000) Iran. J. Bot. 8(2) 251-254.
241	Convolvulus elymaiticus Mozaff.	Convolvulaceae	Mozaffarian (2010) Iran. J. Bot. 16(2) 204-212.
242	Convolvulus iranicus J.R.I.Wood & Scotland	Convolvulaceae	Wood et al. (2015) Phytokeys 51 1-282.
243	Corydalis firouzii Wendelbo	Fumariaceae	Wendelbo (1976) Iran. J. Bot. 1 61-62.
244	Cotoneaster assadii Khat.	Rosaceae	Khatamsaz (1988) Iran. J. Bot. 4(1) 111-125.
245	Cotoneaster esfandiarii Khat.	Rosaceae	Khatamsaz (1991) Iran. J. Bot. 5(1) 1-5.
246	Cousinia argentea Mehregan & Assadi	Asteraceae	Mehregan & Assadi (2009) Willdenowia 39(2) 265-271.
247	Cousinia assadii Attar	Asteraceae	Attar (2011) Iran. J. Bot. 17(2) 150-157.
248	Cousinia atrobracteata Attar	Asteraceae	Attar (2011) Iran. J. Bot. 17(2) 150-157.
249	Cousinia attariae Assadi & Joharchi	Asteraceae	Assadi (2010) Iran. J. Bot. 16(2) 191-196.
250	Cousinia azerbaidjanica Djavadi, Attar & Najafi	Asteraceae	Djavadi et al. (2007) Iran. J. Bot. 13(1) 43-46.

251	Cousinia barezica Assadi	Asteraceae	Assadi (2009) Iran. J. Bot. 15(1) 36-44.
252	Cousinia bazoftensis Attar	Asteraceae	Attar (2011) Iran. J. Bot. 17(2) 150-157.
253	Cousinia brevicaulis Attar, Mozaff. & Mirtadz.	Asteraceae	Attar et al. (2016) Nordic. J. Bot. 34(2) 174-177.
254	Cousinia cordifolia Djavadi & Attar	Asteraceae	Djavadi & Attar. (2006) Feddes Repertorium 117 453ï 458.
255	Cousinia curvibracteata Mehregan	Asteraceae	Mehregan (2011) Iran J. Bot. 17(2) 137-149.
256	Cousinia dalahuensis Attar & Ghahr.	Asteraceae	Attar & Ghahreman (2011) Iran. J. Bot. 8(2) 259-269.
257	Cousinia farimanensis Assadi	Asteraceae	Assadi (2011) Iran. J. Bot. 17(1) 6-9.
258	Cousinia gatchsaranica Mehregan, Assadi & Attar	Asteraceae	Mehregan et al. (2003) Willdenowia 33 107-111.
259	Cousinia ghahremanii Mirtadz. & Attar	Asteraceae	Attar & Mirtadzadini (2009) Iran. J. Bot. 15(2) 146-152.
260	Cousinia hazarensis Mirtadz. & Attar	Asteraceae	Mirtadzadini & Attar (2004) Willdenowia 34 191-194.
261	Cousinia iranshahriana Attar & Maroofi	Asteraceae	Attar & Maroofi (2010) Iran. J. Bot. 16(1) 197-199.
262	Cousinia isfahanica Assadi	Asteraceae	Assadi (2009) Iran. J. Bot. 15 (1) 36-44.
263	Cousinia joharchii Assadi & Mehregan	Asteraceae	Mehregan & Assadi (2016) Phytotaxa 257(3) 271-279.
264	Cousinia karkasensis Mehregan & Djavadi	Asteraceae	Mehregan et al. (2010) Iran. J. Bot. 16(2) 200-203.
265	Cousinia kermanshahensis Attar, Ghahr. & Assadi	Asteraceae	Attar et al. (2001) Iran. J. Bot. 9(1) 55-62.
266	Cousinia khorasanica Djavadi & Attar	Asteraceae	Attar et al. (2006) Rostaniha. 7(suppl.2) 165-175.
267	Cousinia komidjanensis Mehregan	Asteraceae	Mehregan (2011) Iran J. Bot. 17(2) 137-149.
268	Cousinia longibracteata Attar & Mirtadz.	Asteraceae	Attar & Mirtadzadini (2009) Iran. J. Bot. 15(2) 146-152.
269	Cousinia maassoumii Assadi	Asteraceae	Assadi (2009) Iran. J. Bot. 15 (1) 36-44.
270	Cousinia mehreganii Assadi	Asteraceae	Assadi (2011) Iran. J. Bot. 17(1) 6-9.
271	Cousinia mobayenii Ghahr. & Attar	Asteraceae	Attar & Ghahreman (2011) Iran. J. Bot. 8(2) 259-269.
272	Cousinia mozdouranensis Djavadi & Attar	Asteraceae	Djavadi & Attar (2005) Feddes Repertorium 116(5-6) 285ï 289.
273	Cousinia nujianensis Attar, Ghahr., Saber & Zarre	Asteraceae	Attar et al. (2005) Iran. J. Bot. 11(1) 65-69.
274	Cousinia oshtorankuhensis Attar	Asteraceae	Attar (2011) Iran. J. Bot. 17(2) 150-157.
275	Cousinia papillosa Djavadi & Attar	Asteraceae	Djavadi et al. (2007) Rostaniha 8(2) 65-73.
276	Cousinia parsana Ghahr., Iranshahr & Attar	Asteraceae	Ghahreman et al. (1999) Iran. J. Bot. 8(1) 15-22.
277	Cousinia persica Djavadi & Attar	Asteraceae	Djavadi & Attar. (2006) Feddes Repertorium 117 453ï 458.
278	Cousinia pseudocandolleana Assadi	Asteraceae	Assadi (2009) Iran. J. Bot. 15 (1) 36-44.
279	Cousinia saloukensis Mehregan	Asteraceae	Mehregan (2011) Willdenowia 41 261ï 265.

280	Cousinia shebliensis Ghahr., Iranshahr & Attar	Asteraceae	Ghahreman et al. (1999) Iran. J. Bot. 8(1) 15-22.
281	Cousinia shulabadensis Attar & Ghahr.	Asteraceae	Attar & Ghahreman (2002) Iran. J. Bot. 9(2) 161-169.
282	Cousinia subpectinata Mirtadz., Attar & Assadi	Asteraceae	Mirtadzadini et al. (2004) Iran. J. Bot. 10(2) 143-146.
283	Cousinia taybadensis Djavadi & Attar	Asteraceae	Djavadi et al. (2006) Rostaniha 7(suppl.2) 165-175.
284	Cousinia yasujensis Attar	Asteraceae	Attar (2011) Iran. J. Bot. 17(2) 150-157.
285	Cousinia zardkuhensis Attar & Ghahr.	Asteraceae	Attar & Ghahreman (2002) Iran. J. Bot. 9(2) 161-169.
286	Crataegus aminii Khat.	Rosaceae	Khatamsaz (1991) Iran. J. Bot. 5(1) 47-56.
287	Crataegus assadii Khat.	Rosaceae	Khatamsaz (1991) Iran. J. Bot. 5(1) 47-56.
288	Crataegus babakhanloui Khat.	Rosaceae	Khatamsaz (1991) Iran. J. Bot. 5(1) 47-56.
289	Crataegus zagrica Khat.	Rosaceae	Khatamsaz (2000) Iran. J. Bot. 8(2) 181-186.
290	Crocus gunae RukġǕns	Iridaceae	Ruksans (2015) Int. Rock Gard. 61 1-26.
291	Crocus iranicus RukġǕns	Iridaceae	Ruksans (2015) Int. Rock Gard. 61 1-26.
292	Crocus reinhardii RukġǕns	Iridaceae	Ruksans (2015) Int. Rock Gard. 61 1-26.
293	Crotalaria assadii Zaeifi	Fabaceae	Zaeifi (2015) Iran. J. Bot. 21(2) 91-93.
294	Cymbalaria bakhtiarica Podlech & Iranshahr	Scrophulariaceae	Podlech & Iranshahr (2015) Fl. Iranica 180 1-125.
295	Cynoglossum semnanicum Khat.	Boraginaceae	Khatamsaz (1999) Iran. J. Bot. 8(1) 1-8.
296	Dianthus denaicus Assadi	Caryophyllaceae	Assadi (1985) Iran. J. Bot. 3(1) 9-45.
297	Dianthus diversifolius Assadi	Caryophyllaceae	Assadi (1985) Iran. J. Bot. 3(1) 9-45.
298	Dianthus hafezii Assadi	Caryophyllaceae	Assadi (1985) Iran. J. Bot. 3(1) 9-45.
299	Dianthus pseudocrinitus Behrooz. & Joharchi	Caryophyllaceae	Vaezi et al. (2014) Phytotaxa 156(2) 59-73.
300	Dianthus rudbaricus Assadi	Caryophyllaceae	Assadi (1985) Iran. J. Bot. 3(1) 9-45.
301	Dianthus sahandicus Assadi	Caryophyllaceae	Assadi (1985) Iran. J. Bot. 3(1) 9-45.
302	Dionysia archibaldii Wendelbo	Primulaceae	Wendelbo (1976) Bot. Bot. 120(2) 144-148.
303	Dionysia assadii Borjian	Primulaceae	Borjian et al. (2014) Nordic J. Bot. 32 717ï 722.
304	Dionysia aubrietioides Jamzad & Mozaff.	Primulaceae	Jamzad (1996) Iran. J. Bot. 7(1) 15-30.
305	Dionysia bazoftica Jamzad	Primulaceae	Jamzad (1996) Iran. J. Bot. 7(1) 15-30.
306	Dionysia cristagalli Lidén	Primulaceae	Liden (2007) Willdenowia 37(1) 37-61.
307	Dionysia esfandiarii Wendelbo	Primulaceae	Wendelbo (1970) Not. Bot. 123(2) 300-309.
308	Dionysia iranica Jamzad	Primulaceae	Jamzad (1996) Iran. J. Bot. 7(1) 15-30.

309	Dionysia iranshahrii Wendelbo	Primulaceae	Wendelbo (1976) Iran. J. Bot. 1 71-73.
310	Dionysia khatamii Mozaff.	Primulaceae	Mozaffarian (2002) Pakistan J. Bot. 34(3) 391-396.
311	Dionysia khuzistanica Jamzad	Primulaceae	Jamzad (1996) Iran. J. Bot. 7(1) 15-30.
312	Dionysia mozaffarianii Lidén	Primulaceae	Liden (2000) Iran. J. Bot. 8(2) 303-308.
313	Dionysia robusta Younesi	Primulaceae	Younesi et al. (2015) Willdenowia 46(1) 105-112.
314	Dionysia tacamahaca Lidén	Primulaceae	Liden (2007) Willdenowia 37(1) 37-61.
315	Dionysia termeana Wendelbo	Primulaceae	Wendelbo (1970) Not. Bot. 123(2) 300-309.
316	Dionysia viva Lidén & Zetterl.	Primulaceae	Liden (2007) Willdenowia 37(1) 37-61.
317	Dionysia zagrica Grey-Wilson	Primulaceae	Grey-Wilson (1974) Kew Bulletin 29(4) 687-694.
318	Dionysia zetterlundii Lidén	Primulaceae	Liden (2007) Willdenowia 37(1) 37-61.
319	Dionysia zschummelii Lidén	Primulaceae	Liden (2007) Willdenowia 37(1) 37-61.
320	Dracocephalum ghahremanii Jamzad	Lamiaceae	Jamzad (2012) Lamiaceae (in) Assadi et al. Fl. Iran 76 1-1068.
321	Echinops abazariae Mozaff.	Asteraceae	Mozaffarian (2006) Iran. J. Bot. 11(2) 197-239.
322	Echinops austro-iranicus Mozaff.	Asteraceae	Mozaffarian (2006) Iran. J. Bot. 11(2) 197-239.
323	Echinops avajensis Mozaff.	Asteraceae	Mozaffarian (2006) Iran. J. Bot. 11(2) 197-239.
324	Echinops barezicus Montaz. & Mozaff.	Asteraceae	montazerolghaem et al. (2016) Phytotaxa 263(2) 81-97.
325	Echinops delicatus Mozaff.	Asteraceae	Mozaffarian (2006) Iran. J. Bot. 11(2) 197-239.
326	Echinops kazerunensis Mozaff.	Asteraceae	Mozaffarian (2006) Iran. J. Bot. 11(2) 197-239.
327	Echinops kermanshahanicus Mozaff.	Asteraceae	Mozaffarian (2006) Iran. J. Bot. 11(2) 197-239.
328	Echinops khansaricus Mozaff.	Asteraceae	Mozaffarian (2006) Iran. J. Bot. 11(2) 197-239.
329	Echinops khuzistanicus Mozaff.	Asteraceae	Mozaffarian (2006) Iran. J. Bot. 11(2) 197-239.
330	Echinops laricus Mozaff.	Asteraceae	Mozaffarian (2006) Iran. J. Bot. 11(2) 197-239.
331	Echinops leiopolyceroides Mozaff.	Asteraceae	Mozaffarian (2006) Iran. J. Bot. 11(2) 197-239.
332	Echinops psammophilus Mozaff.	Asteraceae	Mozaffarian (2006) Iran. J. Bot. 11(2) 197-239.
333	Echinops quercetorum Mozaff.	Asteraceae	Mozaffarian (2008) Iran. J. Bot. 14(2) 87-94.
334	Echinops sabzevarensis Mozaff.	Asteraceae	Mozaffarian (2006) Iran. J. Bot. 11(2) 197-239.
335	Echinops shulabadensis Mozaff.	Asteraceae	Mozaffarian (2006) Iran. J. Bot. 11(2) 197-239.
336	Echinops viscidulus Mozaff.	Asteraceae	Mozaffarian (2006) Iran. J. Bot. 11(2) 197-239.
337	Echium khuzistanicum Mozaff.	Boraginaceae	Mozaffarian (1994) Iran. J. Bot. 6(2) 235-243.

338	Elymus zagricus Assadi	Poaceae	Assadi (1994) Iran. J. Bot. 6(2) 185-195.
339	Eminium jaegeri Bogner & P.C.Boyce	Araceae	Bonger & Boyce (2008) Willdenowia 38 149-153.
340	Ephedra laristanica Assadi	Ephedraceae	Assadi (1996) Iran. J. Bot. 7(1) 1-6.
341	Eremostachys lanata Jamzad	Lamiaceae	Jamzad (1987) Iran. J. Bot. 3(2) 111-116.
342	Eryngium iranicum Mozaff.	Apiaceae	Mozaffarian (2013) Rostaniha 14(1) 36-42.
343	Erysimum elymaiticum Mozaff.	Asteraceae	Mozaffarian (2008) Iran. J. Bot.14 (2) 87-94.
344	Erysimum hezarense Moazzeni	Brassicaceae	Moazzeni et al. (2014) Phytotaxa 175(5) 241ï 248.
345	Erysimum polatschekii Moazzeni, Assadi & Al-Shehbaz	Brassicaceae	Moazzeni et al. (2016) Phytotaxa 269(1) 047ï 053.
346	Euphorbia acanthodes Akhani	Euphorbiaceae	Akhani (2004) Bot. j. Linn. Soc. 146 107ï 121.
347	Euphorbia ferdowsiana Pahlevani	Euphorbiaceae	Pahlevani (2015) Bot. J. Linn. Soc. 177(3) 335ï 377.
348	Euphorbia gorenflotii Mobayen	Euphorbiaceae	Mobayen (1984) Iran. J. Bot. 2(2) 159-166.
349	Euphorbia iranshahrii Pahlevani	Euphorbiaceae	Pahlevani & Mozaffarian (2011) Adansonia 33(1) 93-99.
350	Euphorbia khorasanica Saeidi & Ghayorm.	Euphorbiaceae	Mehrvarz & Ghayormand (2015) Ann. Bot. Fenn. 52(1-2) 38-42.
351	Euphorbia mazandaranica Pahlevani	Euphorbiaceae	Pahlevani & Riina (2014) Nordic J. Bot. 32(3) 257ï 278.
352	Euphorbia spartiformis Mobayen	Euphorbiaceae	Mobayen (1984) Iran. J. Bot. 2(2) 159-166.
353	Euphorbia sulphurea Pahlevani	Euphorbiaceae	Pahlevani (2015) Bot. J. Linn. Soc. 177(3) 335ï 377.
354	Farsetia assadii Kavousi	Brassicaceae	Kavousi (2001) Iran. J. Bot. 9(1) 47-54.
355	Ferula hezarlalehzarica Ajani	Apiaceae	Ajani & Ajani (2008) Edinb. J. Bot. 65(3) 425-431.
356	Fritillaria avromanica Advay & Tekкen	Liliaceae	Advay et al. (2015) nordic. J. Bot. 33(5) 526ï 531.
357	Gagea calcicola Zarrei & Wilkin	Liliaceae	Zarrei et al. (2010) Kew Bulletin 65 89-96.
358	Gagea robusta Zarrei & Wilkin	Liliaceae	Zarrei et al. (2010) Kew Bulletin 65 327-336.
359	Gaillonia dezfulensis Naanaie & Assadi	Rubiaceae	Yusef Naanaie & Assadi (2011) Iran. J. Bot. 17(2) 227-229.
360	Galium tehranicum Moussavi, Ghahr. & Attar	Rubiaceae	Moussavi et al. (1993) Iran. J. Bot. 6(1) 145-147.
361	Geum iranicum Khat.	Rosaceae	Khatamsaz (1987) Iran. J. Bot. 3(2) 89-94.
362	Glaucium elegantissimum Mobayen	Papaveraceae	Mobayen (1984) Iran. J. Bot. 2(2) 159-166.
363	Glaucium golestanicum A.Gran & Sharifnia	Papaveraceae	Gran & Sharifnia (2008) Iran. J. Bot. 14(1) 23-38.
364	Glaucium mathiolifolium Mobayen	Papaveraceae	Mobayen (1984) Iran. J. Bot. 2(2) 159-166.
365	Gypsophila alvandica Falat., F.Ghahrem. & Assadi	Caryophyllaceae	Falatoury et al. (2015) Phytotaxa 222(4) 276-282.

366	Gypsophila elymaitica Mozaff.	Caryophyllaceae	Mozaffarian (2008) Iran. J. Bot.14(2) 87-94.
367	Gypsophila pseudopallida Falat., Assadi & F.Ghahrem.	Caryophyllaceae	Falatoury et al. (2016) Phyton 56(1) 1-13.
368	Gypsophila rupestris Mozaff.	Caryophyllaceae	Mozaffarian (1991) Iran. J. Bot. 5(1) 29-39.
369	Gypsophila yazdiana Falat., F.Ghahrem. & Assadi	Caryophyllaceae	Falatoury (2016) Novon 24(4) 347-351.
370	Halanthium alaeflavum Assadi	Amaranthaceae	Assadi (1992) Iran. J. Bot. 5(2) 57-63.
371	Halotis pedunculata Assadi	Amaranthaceae	Assadi (1992) Iran. J. Bot. 5(2) 57-63.
372	Haplophyllum bakhteganicum Soltani & Khosravi	Rutaceae	Soltani & Khosravi (2005) Willdenowia 35 293-298.
373	Hedysarum al-shehbazii Ranjbar	Fabaceae	Ranjbar et al. (2007) Bot. J. Linn. Soc. 155 505-512.
374	Hedysarum gypsophilum Dehshiri	Fabaceae	Dehshiri & Goodarzi (2016) Ann. Bot. Fenn. 53(1ï 2) 21-26.
375	Hedysarum marandense Mozaff.	Fabaceae	Akrami et al. (2011) Iran. J. Bot. 17(1) 20-23.
376	Hedysarum neyshaboricum Ranjbar	Fabaceae	Ranjbar (2010) Novon 20(3) 329-333.
377	Hedysarum orumiehense Ranjbar	Fabaceae	Ranjbar (2010) Novon 20(3) 329-333.
378	Hedysarum persicum Bidarlord, F.Ghahrem. & Mozaff.	Fabaceae	Bidarlord et al. (2015) Phytotaxa 234(3) 294-298.
379	Heliotropium esfahanicum Khat.	Boraginaceae	Khatamsaz (2000) Iran. J. Bot. 8(2) 181-186.
380	Heliotropium khayyamii Akhani	Boraginaceae	Akhani et al. (2016) Phytotaxa 249(1) 159-180.
381	Heliotropium shirazicum Mozaff.	Rubiaceae	Mozaffarian (2006) Iran. J. Bot. 12(2) 107-113.
382	Heliotropium ziegleri Akhani	Boraginaceae	Akhani (2007) Bot. j. Linn. Soc. 155(3) 401ï 425.
383	Heteroderis pusilla (Boiss.) Boiss.	Asteraceae	Nasseh (2010) Iran. J. Bot. 16(1) 91-95.
384	Hieracium piranshahricum Tavakkoli & Assadi	Asteraceae	Tavakkoli & Assadi (2005) Iran. J. Bot. 11(1) 59-63.
385	Hyoscyamus bornmulleri Khat.	Solanaceae	Ghahreman & khatamsaz (1996) Iran. J. Bot. 7(1) 31-37.
386	Hypericum dogonbadanicum Assadi	Acanthaceae	Assadi (1984) Iran. J. Bot. 2(2) 83-93.
387	Indigofera sinuspersica Mozaff.	Fabaceae	Mozaffarian (1996) Iran. J. Bot. 7(1) 127-142.
388	Jurinea cartilaginea Mozaff.	Asteraceae	Mozaffarian (1988) Iran. J. Bot. 4(1) 61-70.
389	Jurinea mobayenii Ghahr. & Mirtadz.	Asteraceae	Ghahreman & Mirtadzadini (2000) Iran. J. Bot. 8(2) 245-250.
390	Klasea nana Ranjbar & Negaresh	Asteraceae	Ranjbar et al. (2012) Ann. Bot. Fenn. 49 402-406.
391	Lactuca birjandica Mozaff.	Asteraceae	Mozaffarian (2015) Iran. J. Bot. 21(1) 24-29.
392	Lactuca denaensis N. Kilian & Djavadi	Asteraceae	kilian et al. (2012) Phytokeys 11 61ï 77.
393	Lactuca gilanica Mozaff.	Asteraceae	Mozaffarian (2015) Iran. J. Bot. 21(1) 24-29.
394	Lactuca hazaranensis Djavadi & N. Kilian	Asteraceae	kilian et al. (2012) Phytokeys 11 61ï 77.

395	Lagochilus lorestanicus Dehshiri & Mozaff.	Lamiaceae	Dehshiri & Mozaffarian (2013) Nordic J. Bot. 31 278-281.
396	Lagochilus quadridentatus Jamzad	Lamiaceae	Jamzad (1988) Iran. J. Bot. 4(1) 91-103.
397	Laser rechingeri Akhani	Apiaceae	Akhani (1996) Ann. Naturhist. Mus. Wien 98(B) 97-105.
398	Lathyrus alamutensis Mozaff., Ahavazi & Charkhch.	Fabaceae	Mozaffarian et al. (2008) Iran. J. Bot. 14(1) 7-9.
399	Linaria azerbaijanensis Hamdi & Assadi	Scrophulariaceae	Hamdi et al. (2008) Bot. J. Linn. Soc. 158 734ï 742.
400	Linaria boushehrensis Hamdi & Assadi	Scrophulariaceae	Hamdi et al. (2008) Iran. J. Bot. 14 (1) 16-22.
401	Linaria golestanensis Hamdi & Assadi	Scrophulariaceae	Hamdi et al. (2006) Iran. J. Bot. 11(2) 251-258.
402	Linaria guilanensis Hamdi & Assadi	Scrophulariaceae	Hamdi & Assadi (2006) Feddes Repertorium 117 501-507.
403	Linaria iranica Hamdi & Assadi	Scrophulariaceae	Hamdi & Assadi (2008) Novon 18(3) 340-343.
404	Linaria karajensis Hamdi & Assadi	Scrophulariaceae	Hamdi et al. (2005)Iran. J. Bot. 11(1) 79-83.
405	Linaria khorasanensis Hamdi & Assadi	Scrophulariaceae	Hamdi et al. (2005) Willdenowia 35(2) 299-304.
406	Linaria mazandaranensis Hamdi & Assadi	Scrophulariaceae	Hamdi et al. (2006) Iran. J. Bot. 11(2) 251-258.
407	Linaria Shahroudensis Hamdi & Assadi	Scrophulariaceae	Hamdi et al. (2008) Bot. J. Linn. Soc. 158 734ï 742.
408	Matthiola iranica Zeraatkar, Mahmoodi, F.Ghahrem. & Maassoumi	Brassicaceae	Mahmoodi et al. (2016) Phytotaxa 261(2) 194-198.
409	Matthiola shehbazii Ranjbar & Karami	Brassicaceae	Ranjbar & Karami (2014) Nordic J. Bot. 32(6) 713-716.
410	Mentha mozaffarianii Jamzad	Lamiaceae	Jamzad (1987) Iran. J. Bot. 3(2) 111-116.
411	Milium atropatanum Maroofi	Poaceae	Maroofi (2011) Iran. J. Bot. 17 (1) 10-14.
412	Minuartia khorassanica Assadi & Mostafavi	Caryophyllaceae	Mostafavi et al. (2012) Feddes Repertorium 122(3-4) 309-318.
413	Minuartia sabalanica Assadi & Mostafavi	Caryophyllaceae	Mostafavi et al. (2011) Iran. J. Bot. 17(2) 220-226.
414	Muscari kurdicum Maroofi	Liliaceae	Maroofi (2007) Iran. J. Bot. 13(2) 75-77.
415	Myopordon damavandica Mozaff.	Asteraceae	Mozaffarian (1991) Iran. J. Bot. 5(1) 29-39.
416	Nanorrhinum baluchestanicum Naanaie, Assadi & Tavassoli	Scrophulariaceae	Yusef Naanaie & Tavassoli (2010) Iran. J. Bot. 16(1) 114-124.
417	Nanorrhinum khuzestanicum Naanaie, Assadi & Tavassoli	Scrophulariaceae	Yusef Naanaie & Tavassoli (2010) Iran. J. Bot. 16(1) 114-124.
418	Nepeta assadii Jamzad	Lamiaceae	Jamzad (1991) Iran. J. Bot. 5(2) 69-74.
419	Nepeta bazoftica Jamzad	Lamiaceae	Jamzad (2009) Iran. J. Bot. 15(2) 141-145.
420	Nepeta binaloudensis Jamzad	Lamiaceae	Jamzad (1991) Iran. J. Bot. 5(1) 17-27.
421	Nepeta bokhonica Jamzad	Lamiaceae	Jamzad (1999) Iran. J. Bot. 8(1) 43-48.
422	Nepeta makuensis Jamzad & Mozaff.	Lamiaceae	Jamzad (1998) Iran. J. Bot. 7(2) 249-253.

423	Nepeta minuticephala Jamzad	Lamiaceae	Jamzad (1999) Iran. J. Bot. 8(1) 43-48.
424	Nepeta natanzensis Jamzad	Lamiaceae	Jamzad (2006) Iran. J. Bot. 11(2) 143-148.
425	Nepeta pogonosperma Jamzad & Assadi	Lamiaceae	Jamzad & Assadi (1984) Iran. J. Bot. 2(2) 95-102.
426	Nepeta sahandica Noroozi & Ajani	Lamiaceae	Noroozi & Ajani (2013) Novon 22(3) 297-303.
427	Nepeta shahmirzadensis Assadi & Jamzad	Lamiaceae	Jamzad & Assadi (1984) Iran. J. Bot. 2(2) 95-102.
428	Nonea iranica Falat. & Pakravan	Boraginaceae	Falatoury et al. (2011) Feddes Repertorium 122 1-8.
429	Onobrychis alamutensis Amirahm., Kaz.Osaloo, Charkhch.	Fabaceae	Amirahmadi et al. (2014) Turk. J. Bot. 38 658-664.
430	Onobrychis aurea Ranjbar, Amirab. & Ghahrem.	Fabaceae	Ranjbar et al. (2004) Willdenowia. 34 187-190.
431	Onobrychis iranensis Amirab. & Ghanavati	Fabaceae	Amirabadizadeh et al. (2009) Iran. J. Bot. 15(1) 45-50.
432	Onobrychis marandensis Amirab. & Ghanavati	Fabaceae	Amirabadizadeh et al. (2012) Iran. J. Bot. 18(1) 55-58.
433	Onobrychis mozaffarianii Amirab.	Fabaceae	Amirabadizadeh et al. (2007) Iran. J. Bot. 13(1) 53-56.
434	Onosma azarbaidjanensis Mehrabian	Boraginaceae	Mehrabian et al. (2014) Feddes Repertorium 124 69ï 79.
435	Onosma ghahremanii Attar & Naqinezhad	Boraginaceae	Naqinezhad & Attar (2016) Phytotaxa 268(4) 286-290.
436	Onosma iranshahrii Ghahr. & Attar	Boraginaceae	Ghahreman & Attar (1996) Iran. J. Bot. 7(1) 51-55.
437	Onosma khorassanica Attar & Joharchi	Boraginaceae	Attar & Joharchi (2006) Rostaniha 7(suppl.2) 112-118.
438	Onosma maculata Ranjbar & Almasi	Boraginaceae	Almasi & Ranjbar (2015) Nordic J. Bot. 33(5) 522-525.
439	Onosma mozaffariani Mehrabian	Boraginaceae	Mehrabian et al. (2014) Feddes Repertorium 124 69ï 79.
440	Onosma sabalanica Ponert	Boraginaceae	Ponert (1975) Feddes Repertorium 86(9-10) 503-505.
441	Onosma sheidaii Mehrabian	Boraginaceae	Mehrabian et al. (2014) Feddes Repertorium 124 69ï 79.
442	Opsicarpium insignis Mozaff.	Apiaceae	Mozaffarian (2003) Bot. Zhurn. (Moscow & Leningrad) 88(2) 93-94.
443	Ornithogalum boissieri Bidarlord & F.Ghahrem.	Liliaceae	Bidarlord & Ghahremaninejad (2016) Ann. Bot. Fenn. 53(1-2) 69-72.
444	Ornithogalum khuzestanicum Heidaryan, Hamdi & Assadi	Hyacinthaceae	Heidarnya et al. (2012) Iran J. Bot. 18(1) 47-54.
445	Ornithogalum sanandajense Maroofi	Liliaceae	Maroofi (2010) Iran. J. Bot. 16(1) 76-80.
446	Oxytropis bakhtiarica Maassoumi	Fabaceae	Maassoumi (2013) Iran. J. Bot. 19(1) 1-28.
447	Oxytropis compacta Maassoumi & Joharchi	Fabaceae	Maassoumi (2013) Iran. J. Bot. 19(1) 1-28.
448	Oxytropis guilanica Maassoumi & Moradi	Fabaceae	Moradi et al. (2015) Iran. J. Bot. 21(1) 19-23.
449	Oxytropis indurata Maassoumi	Fabaceae	Maassoumi (2013) Iran. J. Bot. 19(1) 1-28.

450	Oxytropis javaherdehi Maassoumi	Fabaceae	Maassoumi (2013) Iran. J. Bot. 19(1) 1-28.
451	Oxytropis kordkoyensis Maassoumi	Fabaceae	Maassoumi (2013) Iran. J. Bot. 19(1) 1-28.
452	Oxytropis mahneshanensis Maassoumi	Fabaceae	Maassoumi (2013) Iran. J. Bot. 19(1) 1-28.
453	Oxytropis pseudosuavis Maassoumi	Fabaceae	Maassoumi (2013) Iran. J. Bot. 19(1) 1-28.
454	Oxytropis rhodontha Vassilcz.	Fabaceae	Vassilczenko (1988) Byull. Moskovsk. Obshch. Isp. Prir., Otd. Biol. 93(3) 97-102.
455	Oxytropis rudbariensis Vassilcz.	Fabaceae	Vassilczenko (1988) Byull. Moskovsk. Obshch. Isp. Prir., Otd. Biol. 93(3) 97-102.
456	Oxytropis sabzavarensis Maassoumi	Fabaceae	Maassoumi (2013) Iran. J. Bot. 19(1) 1-28.
457	Oxytropis salukensis Maassoumi	Fabaceae	Maassoumi (2013) Iran. J. Bot. 19(1) 1-28.
458	Oxytropis shahvarica Maassoumi	Fabaceae	Maassoumi (2013) Iran. J. Bot. 19(1) 1-28.
459	Oxytropis shirkuhi Vassilcz.	Fabaceae	Vassilczenko (1988) Byull. Moskovsk. Obshch. Isp. Prir., Otd. Biol. 93(3) 97-102.
460	Oxytropis sivehensis Maassoumi & Amini Rad	Fabaceae	Maassoumi (2013) Iran. J. Bot. 19(1) 1-28.
461	Oxytropis surmandehi Vassilcz.	Fabaceae	Vassilczenko (1988) Byull. Moskovsk. Obshch. Isp. Prir., Otd. Biol. 93(3) 97-102.
462	Oxytropis yazdi Vassilcz.	Fabaceae	Vassilczenko (1988) Byull. Moskovsk. Obshch. Isp. Prir., Otd. Biol. 93(3) 97-102.
463	Oxytropis zangolehensis Vassilcz.	Fabaceae	Vassilczenko (1988) Byull. Moskovsk. Obshch. Isp. Prir., Otd. Biol. 93(3) 97-102.
464	Paracaryum glandulosum Khat.	Boraginaceae	Khatamsaz (1994) Iran. J. Bot. 6(2) 227-233.
465	Paracaryum khorassanicum Khat.	Boraginaceae	Khatamsaz (1999) Iran. J. Bot. 8(1) 1-8.
466	Paronychia lordeganica Dinarvand & Assadi	Caryophyllaceae	Dinarvand (2008) Iran. J. Bot. 14(1) 10-12.
467	Pentanema kurdistanicum Maroofi & Ghaderi	Asteraceae	Marrofi & Ghaderi (2016) Phytotaxa 253(3) 223ï 226.
468	Phlomoides kermanica Ranjbar & C.Mahmoudi	Lamiaceae	Ranjbar & Mohammadi (2015) Webbia 70(2) 237-245.
469	Piptatherum denaense Hamzehee & Assadi	Poaceae	Hamzehee & Assadi (2015) Iran. J. Bot. 21(1) 01-09.
470	Poa demavandica Assadi & Kavousi	Poaceae	Kavousi et al. (2009) Iran. J. Bot. 15(1) 57-60.
471	Polygonum botuliforme Mozaff.	Polygonaceae	Mozaffarian (1988) Iran. J. Bot. 4(1) 61-70.
472	Polygonum iranicum Mozaff.	Polygonaceae	mozaffarian (2012) Iran.J.Bot.18(2) 159-174.
473	Potentilla schiraziana Khat.	Rosaceae	Khatansaz (1987) Iran. J. Bot. 3(2) 89-94.
474	Psephellus khalkhalensis Ranjbar & Negaresh	Asteraceae	Ranjbar & Negaresh (2014) Phytotaxa 170(3) 199-206.

475	Pycnocycla bashagardiana Mozaff.	Apiaceae	Mozaffarian (1985) Iran. J. Bot. 3(1) 81-86.
476	Pyrus kandevanica Ghahr., Khat. & Mozaff.	Rosaceae	Khatamsaz (1989) Iran. J. Bot. 5(1) 1-5.
477	Ranunculus microflorus Pakravan	Ranunculaceae	Pakravan (2012 Scienceasia 38 419-421.
478	Rheum austroiranicum Taheri & Assadi	Polygonaceae	Taheri & Assadi (2013) Rostaniha 14(1) 85-93.
479	Rheum iranshahrii Taheri & Assadi	Polygonaceae	Taheri & Assadi (2013) Rostaniha 14(1) 85-93.
480	Rheum kordestanicum Taheri & Assadi	Polygonaceae	Taheri & Assadi (2013) Rostaniha 14(1) 85-93.
481	Rheum neyshabourense Baradaran & Jafari	Polygonaceae	Baradaran & Jafari (2014) Nordic J. Bot. 32 723ï 725.
482	Ribes khorasanicum Saghafi & Assadi	Grossulariaceae	Saghafi & Assadi (1996) Iran. J. Bot. 7(1) 11-14.
483	Rochelia retrosepala Khat.	Boraginaceae	Khatamsaz (2001) Iran. J. Bot. 9(1) 73-77.
484	Rosa abrica Khat. & Koobaz	Rosaceae	Koobaz et al. (2011) Rostaniha 12(1) 51-62.
485	Salicornia iranica Akhani	Chenopodiaceae	Akhani (2008) Pak. J. Bot. 40(4) 1635-1655.
486	Salicornia persica Akhani	Chenopodiaceae	Akhani (2008) Pak. J. Bot. 40(4) 1635-1655.
487	Salicornia perspolitana Akhani	Chenopodiaceae	Akhani (2008) Pak. J. Bot. 40(4) 1635-1655.
488	Salicornia sinus-persica Akhani	Chenopodiaceae	Akhani (2008) Pak. J. Bot. 40(4) 1635-1655.
489	Salix baladehensis Maassoumi, Moeeni & Rahimin.	Salicaceae	Maassoumi et al. (2008) Iran. J. Bot. 14(1) 1-6.
490	Salix elymaitica Maassoumi	Salicaceae	Maassoumi (2009) Iran. J. Bot. 15(1) 3-20.
491	Salix firouzkuhensis Maassoumi	Salicaceae	Maassoumi (2009) Iran. J. Bot. 15(1) 3-20.
492	Salix issatissensis Maassoumi, Moeeni & Rahimin.	Salicaceae	Maassoumi et al. (2008) Iran. J. Bot. 14(1) 1-6.
493	Salix lacus-tari Maassoumi & Kazempour	Salicaceae	Maassoumi (2009) Iran. J. Bot. 15(1) 3-20.
494	Salix viridiformis Maassoumi	Salicaceae	Maassoumi (2009) Iran. J. Bot. 15(1) 3-20.
495	Salsola abarghuensis Assadi	Chenopodiaceae	Assadi (1984) Iran. J. Bot. 2(2) 125-148.
496	Salsola austro-iranica Akhani	Chenopodiaceae	Akhani (2015) Pl. Veg. N. W. Persian Gulf 286.
497	Salsola yazdiana Assadi	Chenopodiaceae	Assadi (1984) Iran. J. Bot. 2(2) 125-148.
498	Salvia jamzadii Mozaff.	Lamiaceae	Mozaffarian (1991) Iran. J. Bot. 5(1) 29-39.
499	Salvia shahkuhmahalei Akhani	Lamiaceae	Akhani et al. (2016) Phytotaxa 249(1) 159-180.
500	Saponaria esfandiarii Assadi	Caryophyllaceae	Assadi (1989) Iran. J. Bot. 4(2) 197-204.
501	Saponaria iranica Dashti, Assadi & Sharifnia	Caryophyllaceae	Dashti et al. (2014) Iran. J. Bot. 20(2) 146-151.
502	Satureja avromanica Maroofi	Lamiaceae	Maroofi (2010) Iran. J. Bot. 16(1) 76-80.
503	Satureja kallarica Jamzad	Lamiaceae	Jamzad (1991) Iran. J. Bot. 5(2) 69-74.

504	Satureja kermanshahensis Jamzad	Lamiaceae	Jamzad (2010) Iran. J. Bot. 16(2) 213-217.
505	Satureja khuzistanica Jamzad	Lamiaceae	Jamzad (1994) Iran. J. Bot. 6(2) 215-218.
506	Saxifraga ramsarica Jamzad	Saxifragaceae	Jamzad (1993) Iran. J. Bot. 6(1) 137-141.
507	Scabiosa esfandiarii Jamzad	Dipsacaceae	Jamzad (1989) Iran. J. Bot. 4(2) 183-188.
508	Scrophularia alhagioides Attar & Joharchi	Scrophulariaceae	Attar et al. (2006) Iran. J. Bot. 12(2) 193-202.
509	Scrophularia crassipedunculata Attar & Joharchi	Scrophulariaceae	Attar et al. (2006) Iran. J. Bot. 12(2) 193-202.
510	Scrophularia denaensis Attar	Scrophulariaceae	Attar (2006) Iran. J. Bot. 12(2) 136-143.
511	Scrophularia elymaitica Mozaff.	Scrophulariaceae	Mozaffarian (2010) Iran. J. Bot. 16(2) 204-212.
512	Scrophularia ghahremanii Attar & Hamzehee	Scrophulariaceae	Attar & Hamzehee (2006) Frddes Repertorium 117 508ï 511.
513	Scrophularia iranica Attar	Scrophulariaceae	Attar (2006) Iran. J. Bot. 12(2) 136-143.
514	Scrophularia ispahanica Attar & Nowrouzi	Scrophulariaceae	Attar et al. (2006) Iran. J. Bot. 12(2) 193-202.
515	Scrophularia kermanica Ghahr. & Mirtadz.	Scrophulariaceae	Ghahreman & Mirtadzadini (2000) Iran. J. Bot. 8(2) 245-250.
516	Scrophularia khorassanica Attar & Joharchi	Scrophulariaceae	Attar et al. (2006) Iran. J. Bot. 12(2) 193-202.
517	Scrophularia papyracea Attar	Scrophulariaceae	Attar (2006) Iran. J. Bot. 12(2) 136-143.
518	Scrophularia schiraziana Attar & Hatami	Scrophulariaceae	Attar et al. (2006) Iran. J. Bot. 12(2) 193-202.
519	Scrophularia shulabadensis Attar & Hamzehee	Scrophulariaceae	Attar & Hamzehee (2006) Frddes Repertorium 117 508ï 511.
520	Scrophularia tortuosissima Attar & Joharchi	Scrophulariaceae	Attar et al. (2006) Iran. J. Bot. 12(2) 193-202.
521	Sedum elburzense Akhiani & Assadi	Crassulaceae	Akhiani & Assadi (2000) Iran. J. Bot. 8(2) 163-166.
522	Senecio eligulatus B.Nord., Moussavi & Djavadi	Asteraceae	Nordestam et al. (2002) Compositae Newsl 38 42-46.
523	Senecio subnivalis Ajani, Noroozi & Nord.	Asteraceae	Noroozi et al. (2010) Compositae Newsl 48 43-62.
524	Serratula calcarea Mozaff.	Rubiaceae	Mozaffarian (2006) Iran. J. Bot. 107-113.
525	Silene mishudaghensis Gholipour & Parsa Khanghah	Caryophyllaceae	Gholipour & Parsa Khanghah (2015) Iran. J. Bot. 21(2) 118-122.
526	Sisymbrium kermanicum Khodash. & Mirtadz.	Brassicaceae	Khodashenas et al. (2006) Iran. J. Bot. 12(1) 94-96.
527	Solenanthus bakhtiaricus Khat.	Boraginaceae	Khatamsaz (1999) Iran. J. Bot. 8(1) 1-8.
528	Sorbus tiliifolia H.Zare, Amini & Assadi	Rosaceae	Zare et al. (2004) Iran. J. Bot. 10(2) 147-151.
529	Spiraea sheikhii H.Zare	Rosaceae	Zare (2002) Iran. J. Bot. 9(2) 253-255.
530	Suaeda iranshahrii Akhani & Freitag	Chenopodiaceae	Freitag et al. (2013) Rostaniha 14(1) 68-80 .
531	Suaeda khalijefarsica Akhani	Chenopodiaceae	Akhani (2015) Pl. Veg. N. W. Persian Gulf 292.

532	Tanacetum bachtiaricum Mozaff.	Asteraceae	Mozaffarian (2005) Iran. J. Bot. 11(1) 115-127.
533	Tanacetum elbursense Mozaff.	Asteraceae	Mozaffarian (2005) Iran. J. Bot. 11(1) 115-127.
534	Tanacetum sonbolii Mozaff.	Asteraceae	Mozaffarian (2005) Iran. J. Bot. 11(1) 115-127.
535	Taverniera echinata Mozaff.	Fabaceae	Mozaffarian (1988) Iran. J. Bot. 4(1) 61-70.
536	Thlaspi maassoumii Mozaff.	Brassicaceae	Mozaffarian (1996) Iran. J. Bot. 7(1) 127-142.
537	Thlaspi pulvinata Mozaff.	Brassicaceae	Mozaffarian (1996) Iran. J. Bot. 7(1) 127-142.
538	Thymus marandensis Jamzad	Lamiaceae	Jamzad (2009) Iran. J. Bot. 15 (1) 51-56.
539	Tilia sabetii H.Zare	Tiliaceae	Zare et al. (2012) Iran. J. Bot. 18(2) 175-190.
540	Tilia stellatopilosa H.Zare, Amini & Assadi	Tiliaceae	Zare et al. (2012) Iran. J. Bot. 18(2) 175-190.
541	Tragopogon kurdicus Safavi & Maroofi	Asteraceae	Safavi & Maroofi (2014) Iran. J. Bot. 20(1) 05-07.
542	Trichodesma elymaiticum Mozaff.	Asteraceae	Mozaffarian (2008) Iran. J. Bot. 14(2) 87-94.
543	Trigonella bakhtiarica Ranjbar & Z.Hajmoradi	Fabaceae	Ranjbar & Hajmoradi (2015) Phytotaxa 202(1) 26-34.
544	Tulipa wendelboi Matin & Iranshahr	Liliaceae	Matin & Iranshahr (1998) Iran. J. Bot. 7(2) 227-229.
545	Verbascum albidiflorum Ranjbar & Nouri	Scrophulariaceae	Ranjbar & Nouri (2015) Willdenowia 45(1) 147-155.
546	Verbascum scoparium Mozaff.	Scrophulariaceae	Mozaffarian (1988) Iran. J. Bot. 4(1) 61-70.
547	Verbascum shahsavarensis Sotoodeh, Attar & Civeyrel	Scrophulariaceae	Sotoodeh et al. (2015) Phytotaxa 203(1) 76-80.
548	Vicia kurdica Jalilian	Fabaceae	Jalilian et al. (2010) Feddes Repertorium 121 81ï 84.
549	Vincetoxicum assadii Zaeifi	Asclepiadaceae	Zaeifi (1999) Iran. J. Bot. 8(1) 105-110.
550	Vincetoxicum mozaffarianii Zaeifi	Asclepiadaceae	Zaeifi (1999) Iran. J. Bot. 8(1) 105-110.
